# Modulator Driven Formation of a Very Complex Self-Catenated Zinc Metal–Organic Framework

**DOI:** 10.1021/acs.cgd.5c00992

**Published:** 2025-09-20

**Authors:** Alan Braschinsky, Davide M. Proserpio, Toby J. Blundell, Eduardo Rezende Triboni, Jonathan W. Steed

**Affiliations:** † Department of Chemistry, 3057 Durham University, South Road, Durham DH1 3LE, U.K.; ‡ Dipartimento di Chimica, Università Degli Studi di Milano, Via Golgi, 19, 20133 Milano, Italy; § Escola de Engenhara de Lorena, Universidade de São Paulo, Estrada Municipal do Campinho, s/n − Pte. Nova, Lorena SP 12602-810, Brazil

## Abstract

Solvothermal reaction of *N*,*N*′-bis­(5-isophthalic acid)­naphthalenediimide (H_4_BINDI) with zinc­(II) nitrate hexahydrate in dimethylformamide (DMF) in the presence of trifluoroacetic acid as a modulator gives rise to a self-catenated Metal–organic framework (MOF) termed BINDI-ZnSC of unprecedented topological complexity. Using the “all node” method, the topology is assigned as a six-nodal net with point symbol {4.10^2^}_2_{4.12^2^}­{4.6.8}­{4^2^.6.8.10^6^}­{6.10^2^}. In contrast to interpenetrated and other self-catenated MOFs that often exhibit limited pore volume, BINDI-ZnSC exhibits a total of 3730 Å^3^ (62.5% of the unit cell) of solvent-filled channels per unit cell, suggesting that the material is potentially capable of encapsulating not only single molecules but also molecular clusters of some small molecules.

## Graphical abstract

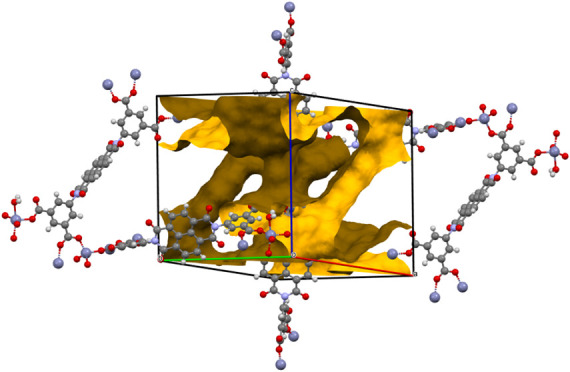

## Introduction

Metal–organic frameworks (MOFs) have revolutionized the fields of materials science and chemistry due to their exceptional structural diversity and functional versatility. These hybrid materials, composed of metal ions or clusters coordinated to organic ligands, exhibit unique properties that have paved the way for advances in gas storage, catalysis, and drug delivery. Over the past decade, significant progress has been made in the synthesis of MOFs, enabling the creation of a vast array of frameworks with diverse functionalities.^1^ Noteworthy advances include the development of MOFs with precise control over pore size and functionality, which have been extensively utilized in gas adsorption and separation technologies.^2,3^ While a large proportion of MOFs are made up by an organic linker bridging together metal secondary building units (SBUs), the synthesis of MOFs with mixed organic linkers,^4^ and mixed metals,^5^ has also been popularised— an effective approach toward synthesizing multifunctional MOFs. These efforts have led to the creation of a MOF subset within the Cambridge Structural Database (CSD), containing over 12,000 crystal structures of three-dimensional MOFs out of a total of over 100,000 MOF-like structures.^6^ Despite this significant achievement, the pursuit of novel MOFs continues to captivate researchers, driven by the immense possibilities these materials present in applications ranging from drug delivery to catalysis.^7,8^ This unexplored territory still presents an exciting challenge and opportunity for the development of MOFs with novel topologies and unique properties. In this work, we report the synthesis and characterization of a surprising MOF, termed BINDI-ZnSC (where SC denotes self-catenation), incorporating *N*,*N*′-bis­(5-isophthalic acid) naphthalenediimide (H_4_BINDI) combined with mono- and dinuclear zinc­(II) SBUs. A search in the CSD identifies 13 MOF structures comprising the BINDI ligand and zinc­(II), out of which three materials^9–11^ form from direct reaction of H_4_BINDI and zinc­(II) salts without coligands. The unique topology of BINDI-ZnSC contrasts with these previously reported MOFs despite closely related synthetic conditions. The compound {[NH_2_(CH_3_)_2_]­[Zn_2_(HBINDI)­BINDI_0.5_]}_*n*_·*n*(8DMF·5H_2_O) (CSD reference code FOMWOV^9^) has a much simpler self-catenated structure, while the others are apparently isostructural, noncatenated channel structures (see Supporting Information (SI) for discussion of these structures).^10,11^ In general, self-entanglement tends to increase the packing efficiency of a framework, giving either nonporous materials or microporous MOFs^12^ useful for efficient capture of small gases.^13^ In this case, however, BINDI-ZnSC exhibits an unprecedented and extremely complex self-catenation, resulting in pores of significant size. The key difference in the synthesis of BINDI-ZnSC is the inclusion of trifluoroacetic acid (TFA) as a modulator. *In situ* synchrotron diffraction studies show that modulators function by competing for the coordination sites of the metal centers in MOFs.^14,15^ This competition facilitates nucleation and crystal growth, as the modulators are subsequently substituted by organic linkers and, in this case, appears to result in significantly greater topological complexity.

## Results and Discussion

The synthesis of the organic linker H_4_BINDI was accomplished by modifying a known literature procedure.^16^ This involved the refluxing of 1,4,5,8-naphthalenetetracarboxylic dianhydride (**1**) and 5-aminoisophthalic acid (**2**) in dimethylformamide (DMF), as illustrated in the Supporting Information (Figure S1). The ligand was characterized by elemental analysis and Fourier transform infrared (FT-IR) and ^1^H NMR spectroscopy. The IR spectrum of H_4_BINDI (Figure S2) corresponds well with literature values.^17^

The single crystal structure of the H_2_BINDI^2–^ dianion as a DMF solvate of its bis­(dimethylammonium) salt was first reported in 2012,^18^ but the structure of the neutral ligand is unknown. Recrystallization of the ligand from dimethyl sulfoxide (DMSO) by vapor diffusion resulted in a DMSO solvate of the neutral ligand, which was characterized by single crystal X-ray diffraction (SC-XRD). The asymmetric unit contains six DMSO molecules and one molecule of H_4_BINDI. Two highly disordered solvent molecules are located between the planar parts of adjacent molecules of H_4_BINDI. These were modeled using the MASK option in Olex2.^19^ The other four, three of which exhibit a simple 2-fold disorder, are linked to the host molecule by O–H···O hydrogen bonds, Figure 1a. These hydrogen bonds are the main stabilizing interactions in this crystal structure, as adjacent molecules of H_4_BINDI are spaced too far apart for any mutual hydrogen bonding or π–π interactions to be present. It is likely that these hydrogen bonding interactions are responsible for the packing arrangement because in the dianion structure crystallized from DMF, the naphthalenediimide moieties participate in π–π stacking with a distance of 3.3 Å.^17^ Figure 1b illustrates the conformation of H_4_BINDI with the terminal benzene-derived rings perpendicular to the naphthalenediimide moiety. The incorporation of such large solvent-filled voids in the ligand structure underlines its tendency to induce void space in the solid state.

**Figure 1:**
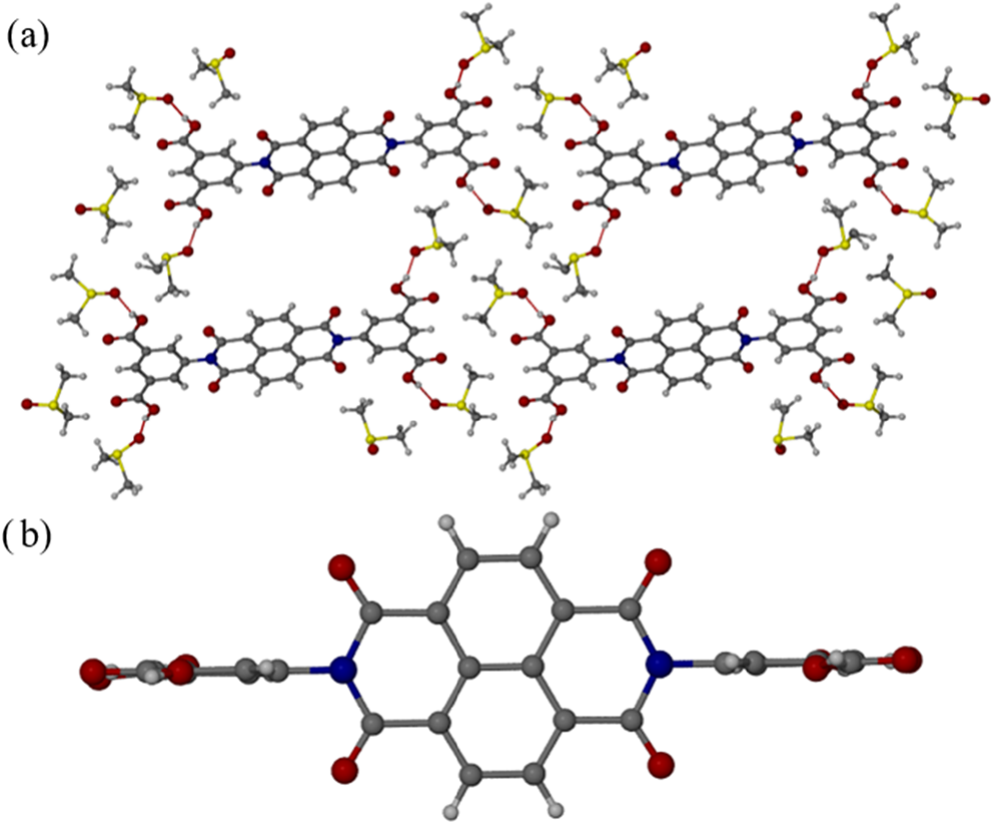
Single crystal structure of H_4_BINDI·6DMSO showing (a) the view of the unit cell along the *b* axis and (b) conformation of the organic linker. The apparent voids in the crystal structure are filled with disordered solvent, which has been removed for clarity. Red dashed lines indicate hydrogen bonding interactions. Gray: C. White: H. Red: O. Blue: N. Yellow: S.

The synthesis of an MOF derived from H_4_BINDI and a source of zinc­(II) ions was undertaken using a solvothermal method. A DMF solution of zinc­(II) nitrate hexahydrate and H_4_BINDI was mixed with TFA as a modulator^20^ and the mixture was sonicated and placed into an acid digestion vessel. The vessel was maintained in an isothermal oven at 120 °C for 72 h, followed by slow cooling to room temperature over the course of 6 h (Scheme 1). A range of ratios of zinc­(II): H_4_BINDI were explored from 2:1 to 4:1 of metal-to-ligand. All products from 2:1 to 4:1 zinc­(II): H_4_BINDI reactions appeared relatively crystalline (Figure S4). While both 2:1 and 3:1 reactions yielded no single crystals, the 4:1 ratio produced both dark, poorly crystalline material and brown-colored, plate-like single crystals of [Zn_3_(H_2_O)_2_(HBINDI)_2_]·*n*DMF (BINDI-ZnSC, *n* = 6.5 or 11.5), see Figure S5 for images of the sample. The as-synthesized MOF crystals were characterized via XRPD, SC-XRD, thermogravimetric analysis, FT-IR, and ^1^H NMR spectroscopy. The single crystal structure determination was carried out twice using both a conventional laboratory Mo–Kα source and synchrotron radiation at the I19 instrument at Diamond. The individual crystals contained different DMF occupancies and both data sets are summarized in the Supporting Information. The structures are otherwise closely related.

**Scheme 1:**
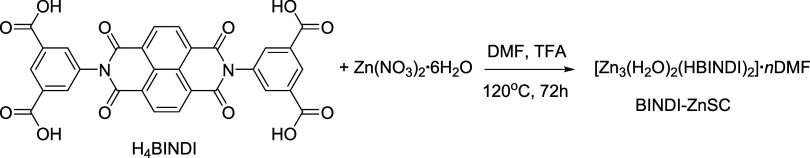
General Synthetic Procedure for the Preparation of the Zinc-Based MOF BINDI-ZnSC

The FT-IR spectrum of the as-synthesized single crystals of BINDI-ZnSC derived from the 4:1 reaction is shown in Figure 2a. The appearance of bands centered at 2931 and 2862 cm^–1^ assigned to DMF C–H stretching indicates that DMF solvent molecules are either encapsulated and/or coordinated inside the framework, as observed recently for lanthanide-based MOFs.^21^ Additionally, the bands centered at 1707 and 1667 cm^–1^ (Figure 2a, black line) shift to 1715 and 1656 cm^–1^, respectively, in the complex, indicating that coordination bonds have formed between zinc­(II) and H_4_BINDI. On the other hand, the wide band attributed to the carboxylic acid O–H stretching has slightly diminished in intensity but is still observed (Figure 2a, red line). Due to the excess of metal ions in this reaction (4:1 M:L), it is unlikely that there is unreacted H_4_BINDI present. Rather, the presence of undeprotonated carboxylic acid groups indicates that some organic linkers exist in a partially protonated form as part of the framework structure. Similarly to BINDI-ZnSC, a partially deprotonated MOF containing zinc­(II) SBUs connected with the H_4_BINDI organic linkers was reported in 2014 by Zhong and co-workers (ref code FOMWOV).^9^ The TGA thermogram of BINDI-ZnSC collected from room temperature up to 600 °C under nitrogen shows a gradual weight loss of 22% beginning close to room temperature and continuing up to 380 °C, attributed to solvent loss from the pores of BINDI-ZnSC (Figure S6). This amount of solvent is equivalent to 9 molecules of DMF per formula unit and is between the two occupancies determined by SC-XRD, suggesting that solvent can move fairly freely into and out of the MOF channels. Solvent loss is followed by the framework decomposition, where a further 60% of the weight is lost by 540 °C. Good agreement is observed between the experimental XRPD pattern and the one calculated from SC-XRD (*vide infra*), indicating bulk purity, albeit with significant (020) preferred orientation and evidence for some amorphous content, Figure 2b. A slight shift to a lower angle diffraction is observed in the room temperature experimental diffractogram because the SC-XRD analyses were performed at low temperature, which results in a slight contraction of the unit cell. Additional low-intensity peaks may represent impurities.

**Figure 2:**
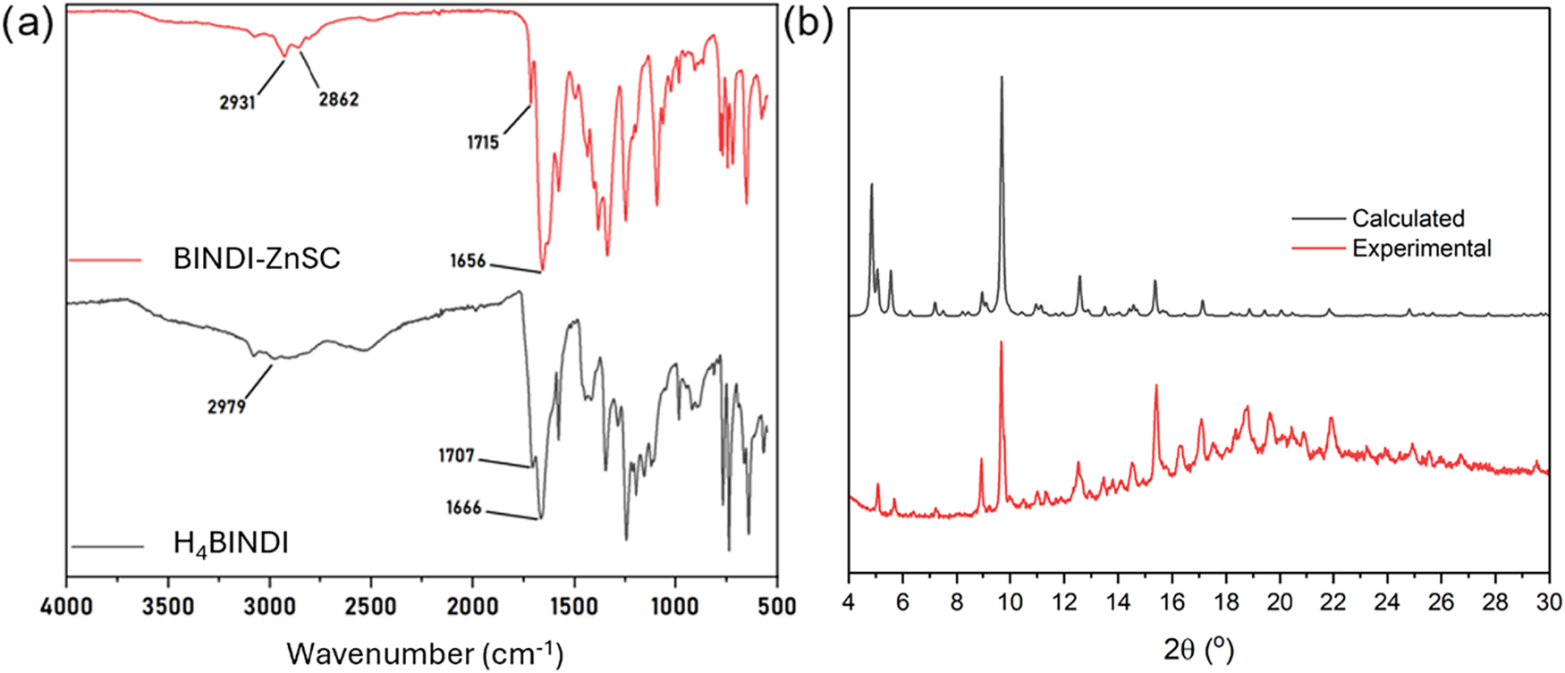
(a) FT-IR spectra of BINDI-ZnSC (red line) and free ligand H_4_BINDI (black line), and (b) powder X-ray diffraction spectra of the as-synthesized single crystals (red pattern) and the calculated pattern obtained from single crystal X-ray diffraction analysis with (020) preferred orientation (black pattern).

Solution-state ^1^H NMR spectroscopy was used to confirm the composition of the as-synthesized single crystals (Figure S7). Dissolution of BINDI-ZnSC crystals in *d*_6_-DMSO was ensured by the addition of one drop of concentrated hydrochloric acid. The presence of signals attributed to the protons of H_4_BINDI (8.32, 8.56, and 8.72 ppm) is observed in similar locations to the spectrum of the free linker (Figure S3). Additionally, signals attributed to solvent DMF protons are observed at 7.94, 2.89, and 2.72 ppm.

The crystal structure analyses of BINDI-ZnSC reveal that the structure comprises a topologically complicated zinc MOF with an empirical framework formula Zn_3_^2+^(HBINDI^3–^)_2_, Figure 3. The material also contains coordinated water, disordered lattice water, DMF, and TFA. The product crystallizes in the centrosymmetric space group *P*1̅ and the asymmetric unit contains three zinc­(II) metal centers and a total of two HBINDI^3–^ anions (one full and two half HBINDI anions in the asymmetric unit), resulting in a 1.5:1 ratio of metal/ligand (M/L). The presence of fully deprotonated BINDI^4–^ linkers alongside two oxonium ions to give charge balance can be ruled out because of the absence of the asymmetric bending mode (υ_4_) in the 1720–1740 cm^–1^ region, characteristic of oxonium ions, in the FT-IR spectrum of BINDI-ZnSC (Figure 2a).^22^ The M/L ratio of 1.5:1 is significantly lower than the ratio used for the synthesis (4:1 M/L); however, attempts to replicate the synthesis at a 1.5:1 M/L ratio did not yield any single crystals.

**Figure 3:**
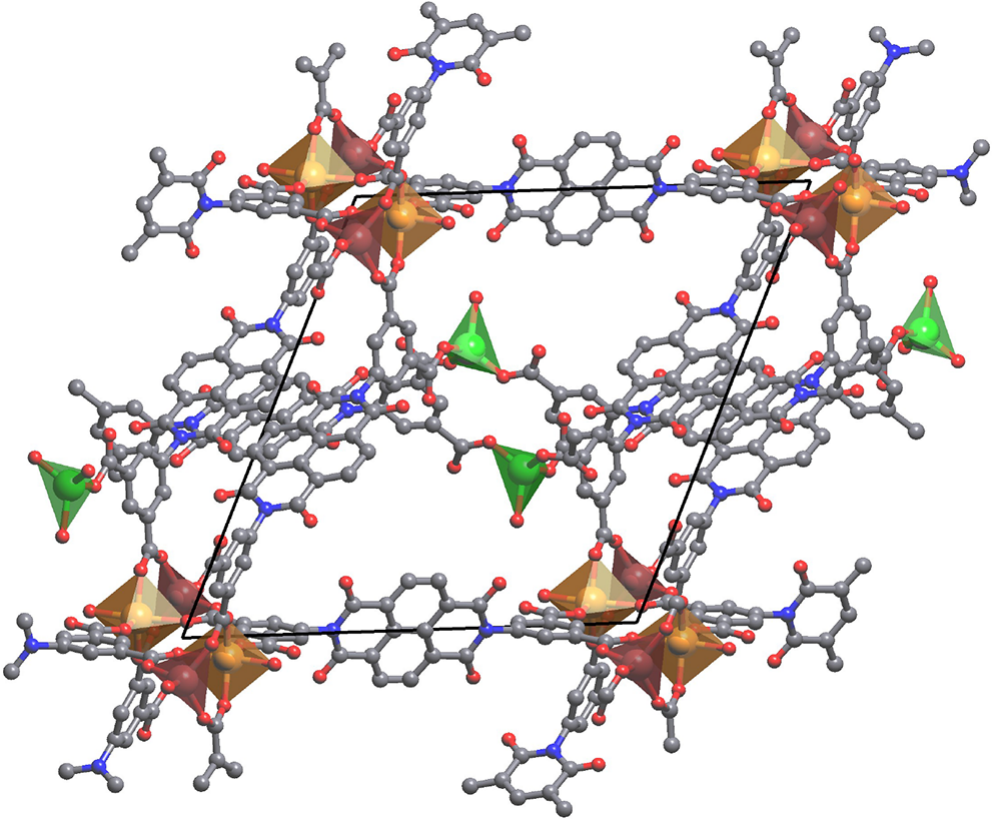
Unit cell of the BINDI-ZnSC host framework along the crystallographic *a* axis. Gray: C. Red: O. Blue: N. Monomeric Zn green, dimeric Zn brown 4-coordinated and orange 5-c. Hydrogen atoms omitted for clarity.

The metal centers in BINDI-ZnSC exhibit two different types of coordination environments in which two of the three unique zinc­(II) ions are 4-coordinate, while the other is 5-coordinate. These two different types of metal center are not incorporated into larger SBU clusters unlike some MOFs in which there are mixed coordination geometries.^23^ Instead, BINDI-ZnSC exhibits mono- and binuclear SBUs as shown in Figure 4a,b. In the case of the binuclear SBU, the zinc­(II) ions are 4- and 5-coordinate and adopt tetrahedral and distorted trigonal bipyramidal geometries, respectively. Both metal centers are coordinated to four different organic ligands, and an additional water molecule is connected to one of the axial positions of the 5-coordinate zinc­(II) center. These two metal centers in the binuclear SBUs are connected by three organic linkers. In contrast, the mononuclear SBUs are tetrahedral, in which one coordination bond is formed with the oxygen atom of a water molecule and the others come from the carboxylates of the organic linker (Figure 4c). The Zn–O_BINDI_ coordination bond lengths vary over a range of 1.808(10) −2.103(5) Å and the two Zn–O_H_2_O_ coordination bond lengths are 2.071(6) and 2.144(11) Å.

**Figure 4:**
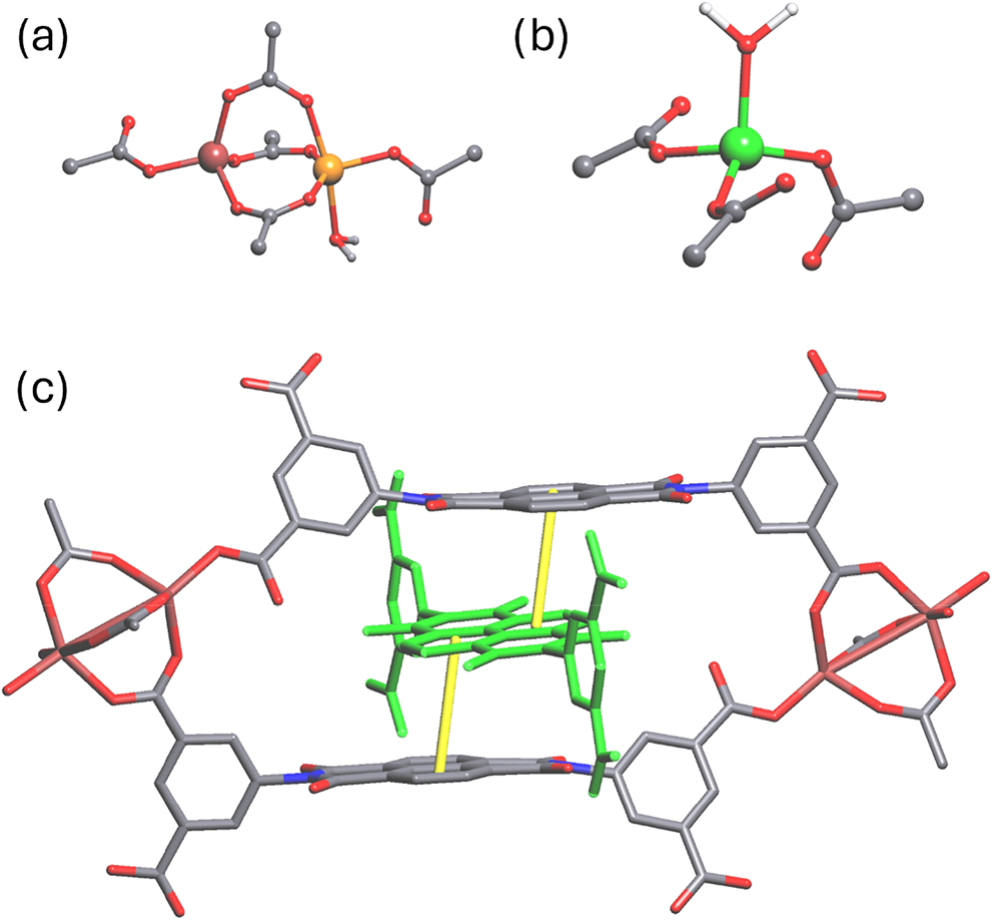
Secondary building units contained in the BINDI-ZnSC showing (a) the dinuclear SBU and (b) the mononuclear SBU. (c) Crystal structure of BINDI-ZnSC showing the self-catenation of ligands and how they bridge the SBUs. Yellow solid lines indicate π–π interactions between the naphthalene moieties of the BINDI ligands. Hydrogen atoms are omitted in panel (c) for clarity. The threading BINDI ligand is colored green for clarity.

Figure 4c shows the single crystal structure of BINDI-ZnSC as a self-catenated MOF consisting of mono- and binuclear SBUs. This structure features two HBINDI^3–^ linkers interconnected via binuclear SBUs, with an additional HBINDI^3–^ ligand threading through the macrocyclic ring formed between the linkers and zinc SBUs, facilitating π–π interactions involving two of the three crystallographically unique linkers. Two distinct π–π interactions are observed between the naphthalene moiety of the threading linker and the linkers adjacent to it. These π–π interactions exhibit interplanar distances of 3.509(4) and 3.659(3) Å and are shown in solid yellow lines in Figure 4c. These π–π distances are shorter than those reported for FOMWOV,^9^ in which the distances range from 3.8–5.3 Å. The orientation of the threading linker is slightly tilted, optimizing the π–π interaction distances compared to a parallel alignment of the linker to its adjacent counterparts. These π–π interactions, outside of coordination bonds between metal centers and the HBINDI^3–^ ligands, likely play an important role in stabilizing the framework.

The related MOF CSD entry FOMWOV^9^ contains a mixture of partially and fully deprotonated linkers—fully deprotonated (BINDI^4–^) and triply deprotonated (HBINDI^3–^)—that are connected via zinc­(II) SBUs. The FT-IR spectrum of this material also supports the partial deprotonation of the framework, with a small, wide band representing the O–H stretching of the carboxylic acid being present. This evidence strongly suggests that BINDI-ZnSC also contains partially deprotonated linkers, averaging a trianion (HBINDI^3–^) state as opposed to being fully deprotonated (BINDI^4–^). This assertion is further supported by a broader CSD^6^ search revealing 187 crystal structures of zinc­(II)-based MOFs with coordinated protonated carboxylic acid groups, revealing that such a coordination environment is not uncommon in MOFs.

The packing arrangement of BINDI-ZnSC reveals that it is a self-catenated network where HBINDI^3–^ linkers form sandwich-like π–π interactions (Figure 5). Two HBINDI^3–^ molecules coordinate to dinuclear SBUs and form a macrocycle. The third HBINDI^3–^ linker threads through the center of this ring and coordinates to the mononuclear tetrahedral SBUs. Thus, the mononuclear SBUs shown in Figure 4b,c are bridged together by the HBINDI^3–^ linkers that complete the sandwich-like π–π interactions between three independent organic linkers, as shown in Figure 5. As a result, there is an interconnected web of solvent-filled channels that spans the framework. The volume of solvent-filled channel per unit cell is 3730 Å^3^ (62.5% of the unit cell) based on a probe radius of 1.2 Å, suggesting that BINDI-ZnSC is potentially capable of encapsulating not only single molecules but molecular clusters of some small molecules and potentially represents an expanded version of the recently reported cluster-capturing MOFs.^21^

**Figure 5:**
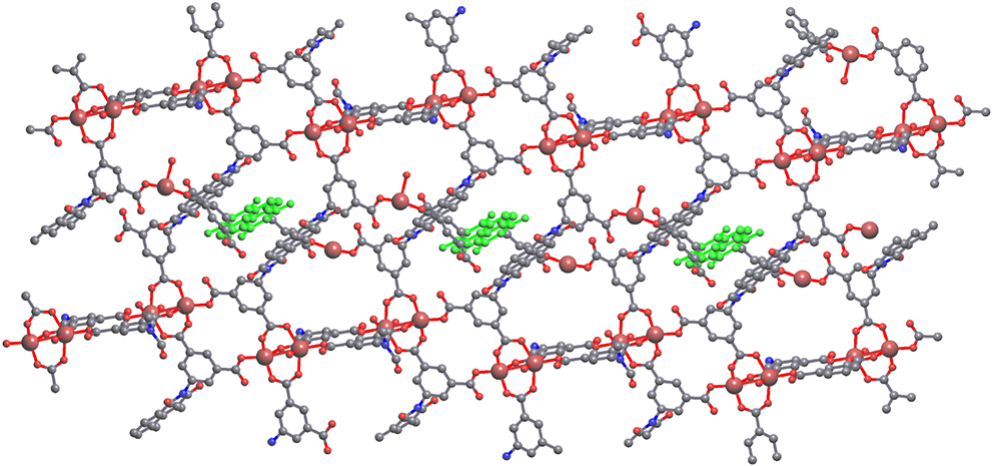
Packing arrangement of BINDI-ZnSC showing how the self-catenation is extended through the lattice, showing sandwich-like π–π interactions (green). Hydrogen atoms omitted for clarity.

To better understand the complex framework of BINDI-ZnSC, the topology was analyzed using the ToposPro software.^24^ The simplification of frameworks using the cluster representation method can be undertaken either using the “single node” or “all node” deconstruction methods.^25^ The main difference between these two methods is how the linkers and SBUs are simplified. The “single node” method treats organic linkers and SBUs as one node. In the “all node” method, SBUs are still treated as one node, but the branch points of the linkers are treated as separate nodes. This means that the advantage of the “all node” method is that it provides a more accurate description of the topology for flexible ligands. Figure 6 visualizes how these simplifications are applied to the linker and SBUs that make up the structure of BINDI-ZnSC. In the “single node” method shown with yellow spheres, all the linkers are 4-connected (4-c node), the dinuclear SBUs are 5-c and the mononuclear SBUs 3-c, while in the “all node” method, the linkers simplify to two 3-c nodes (green spheres), keeping the coordination of the SBUs unchanged. In the case of HBINDI^3–^, the “all node” method describes the topology more accurately, as the shape of the linker is better simplified via two 3-c nodes. Still, both methods will be discussed herein for full clarity and understanding of the underlying topology of BINDI-ZnSC.

**Figure 6:**
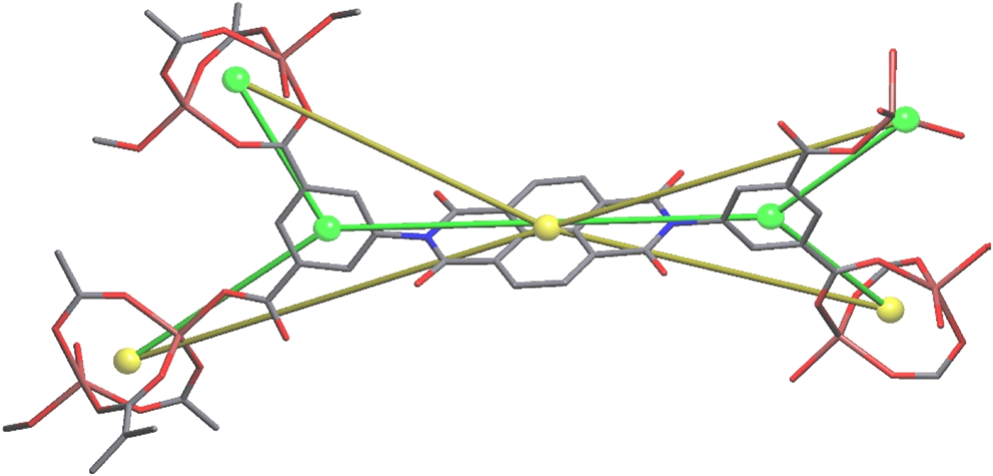
Visual representation of how the SBUs and the linker are simplified in the “single node” (yellow spheres) and “all node” (green spheres) methods. Gray: C. Red: O. Blue: N. Brown: Zn.

First, the “single node” method was used to visualize the underlying net of BINDI-ZnSC. Figure 7a shows how the simplified net looks with respect to the real framework. The “single node” approach yields a new 3,4^3^,5-c 5-nodal net with the point symbol {4.8^2^}_2_{4^2^.6.8^3^}_2_{4^2^.8^2^.10^2^}­{4^2^.8^4^}­{4^3^.6.8^6^}_2_. While the connectivity of the underlying net relatively well represents the real packing arrangement, there are still empty spaces that are also crossed by edges of the net, which indicates it is not the most efficient way to simplify this net. Figure 7b shows the underlying net of BINDI-ZnSC along the [100] direction as determined by the “all node” method, which yields a new 3^5^,5-c 6-nodal net with the point symbol {4.10^2^}_2_{4.12^2^}­{4.6.8}­{4^2^.6.8.10^6^}­{6.10^2^}. These two descriptions of this net have been deposited to the topcryst/topospro database for personal names as *dur*1 and *dur*2.^26^

**Figure 7:**
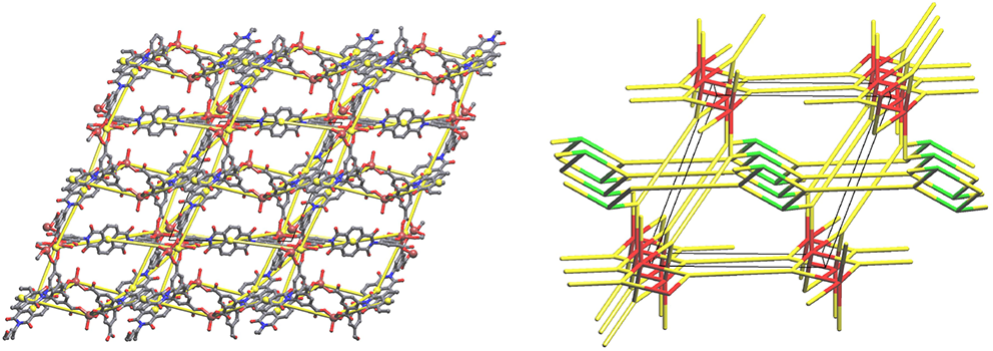
Underlying nets of BINDI-ZnSC as shown along the crystallographic *a* direction determined by using the (a) single node method 3,4^3^,5-c 5-nodal and (b) all node method 3^5^,5-c 6-nodal. In panel (a), Gray: C. Red: O. Blue: N. Brown: Zn. Yellow: Underlying net. In panel (b), Green: 3,3-c linker node. Red:5-c dinuclear SBUs. Green: 3-c mononuclear SBUs.

The Cambridge Structural Database^6^ (version 6.00 April 2025) was searched for any MOFs that contain any H_4_BINDI-derived organic linker, and the search yielded 107 hits. The specific search and the details associated with it are outlined in the Supporting Information. From these 107 hits, 78 (including 10 determinations of the neodymium MOF MAZFEC^27^) contain solely deprotonated H_4_BINDI as the organic linker bridging SBUs, while other MOFs contain mixed ligands. For example, the asymmetric unit of the CSD entry LARKOI^28^ contains one unit of BINDI^4–^ and two 3,5-diamino-1,2,4-triazole ligands bridging together zinc­(II) secondary building units. From the 78 homoleptic hits, only entry RERVIW exhibits the same 3:2 M/L ratio as that of BINDI-ZnSC.^29^ However, this framework contains manganese­(II) metal centers instead of zinc­(II) and, as a result, exhibits a different packing arrangement of the framework compared to that of BINDI-ZnSC. RERVIW crystallizes in the monoclinic *P*2_1_/*c* space group as opposed to the triclinic *P*1̅ of BINDI-ZnSC and exhibits a pillared Kagome lattice topology as a result of two anionic SBUs in which the manganese­(II) metal centers adopt a square pyramidal coordination geometry.

Another structure from the 78 materials consisting only of H_4_BINDI-derived ligands is FOMWOV,^9^ which contains zinc­(II) centers along with the sandwich-type π–π interactions of the organic linker, resulting in a self-catenated framework. In this case, the M/L ratio is 4:3. The synthesis of FOMWOV was achieved via the solvothermal method, where the reaction mixture (1:1 M/L ratio in DMF) was held at 110 °C for 72 h, then slowly cooled to room temperature over the course of 16 h at the rate of 5 °C h^–1^, yielding brown block-shaped single crystals. These reaction conditions are very similar to those used to synthesize BINDI-ZnSC, with the main differences being that the latter was synthesized at 120 °C and in the presence of TFA as a modulator. The structure of FOMWOV was incorrectly described as a 1D + 2D → 3D self-catenated array with dinuclear zinc­(II) SBUs. In fact, FOMWOV is not an example of polycatenation but is simply a unique self-catenated 3D framework. To truly be a 1D + 2D → 3D, the framework would have to be formed from the polycatenation of a 2-dimensional sheet through a one-dimensional ladder-like unit.^30^ Additionally, FOMWOV also contains partially protonated carboxylic acid units of the BINDI ligands. Two types of linkers are present: one in which three of the four carboxylic acid groups are deprotonated (HBINDI^3–^) while the other is fully deprotonated (BINDI^4–^) and they act respectively as 3-connected and 4-c toward the Zn_2_ SBU. This presence, similarly to BINDI-ZnSC, of fully and partially deprotonated organic linkers is likely due to the self-catenated nature of both BINDI-ZnSC and FOMWOV and likely arises from steric hindrance.

To better compare these frameworks, the underlying topology of FOMWOV was also determined using ToposPro.^25^ In the original report, the authors describe the framework as a (3,4,5)-connected net.^9^ However, this underlying net is the result of using the “single node” method, which simplifies the two organic linkers as 3-c and 4-c nodes and was later classified in TopCryst as 3,4,5T399.^31^ As previously discussed, this simplification does not yield the most accurate description of the underlying net. Thus, the “all node” method was employed to determine the underlying net of FOMWOV and is shown in Figure 8. Unlike BINDI-ZnSC, which contains six separate nodes, the underlying net of FOMWOV consists of three nodes: the two distinct deprotonated organic linkers (green) and the dinuclear zinc­(II) SBU (red). This method yields a (3,3,5)-connected net as opposed to the reported (3,4,5)-connected net, named 3,3,5T113. Figure 8 shows that there are no “free” organic linkers, *i.e*., all organic linkers participate in the self-catenation. In contrast, there are layers of self-catenated and “free” organic linkers that bridge two adjacent binuclear SBUs in the structure of BINDI-ZnSC.

**Figure 8:**
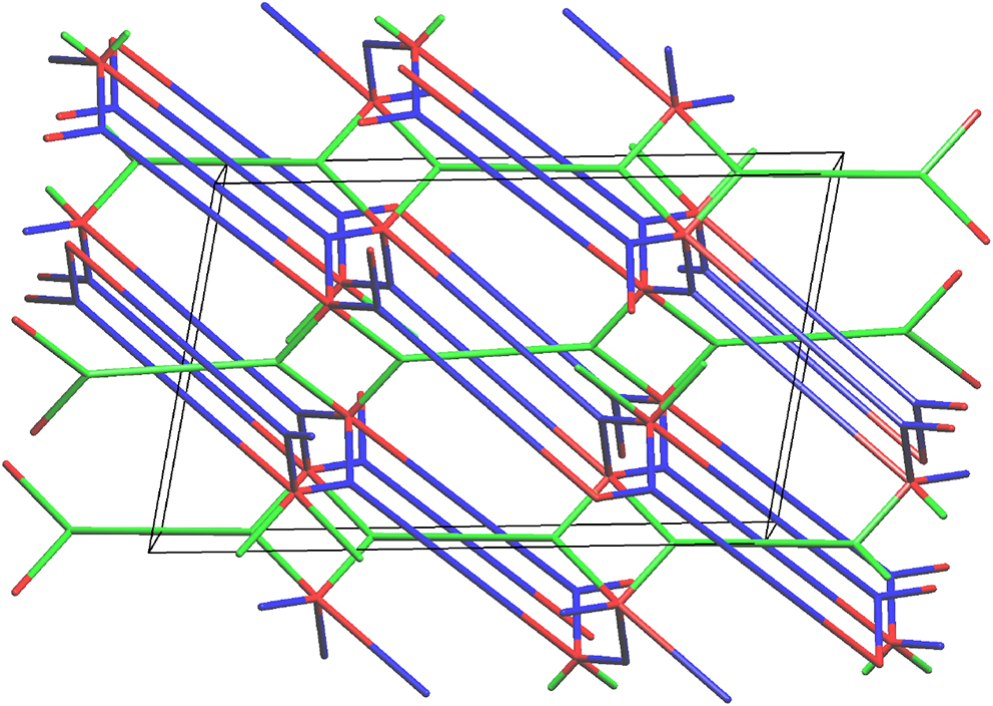
Underlying net 3,3,5T113 of FOMWOV as determined by the “all node” method shown along the *b* direction. Green and Blue: Organic linkers. Red: dinuclear SBU node.

## Conclusions

This work reports the synthesis and characterization of a very unusual partially self-catenated framework BINDI-ZnSC, consisting of mono- and binuclear zinc SBUs bridged together by partially deprotonated organic linkers. Two types of SBUs, mono- and dinuclear, are bridged together by the two types of HBINDI^3–^ organic linkers, leading to a partially self-catenated network with accessible pores. While certain similarities, such as the synthetic conditions, nature of the metal source, and self-catenation, are observed between BINDI-ZnSC and the previously reported FOMWOV, BINDI-ZnSC adopts a much more complicated and unprecedented topology, indicating that the use of a modulator in MOF synthesis can give rise to very surprising and topologically complex outcomes. The large channel pores and unique connectivity make it desirable for assessing future applications in gas capture and sensing.

## Supplementary Material



## Notes

Synthetic procedures and characterization; discussion of previous related structures GOZLEQ and KEVKEE; DSC search for BINDI MOF structures (PDF)
