# Supplementary material for: Modulator Driven Formation of a Very Complex Self-Catenated Zinc Metal–Organic Framework
Source: Cryst Growth Des. 2025 Sep 20;25(19):8181–7. doi: 10.1021/acs.cgd.5c00992 (PMC12492387; doi:10.1021/acs.cgd.5c00992)

Modulator Driven Formation of a Very Complex Self-Catenated Zinc Metal-Organic Framework

Alan Braschinsky[a], Davide M. Proserpio[b], Toby J. Blundell[a], Eduardo Rezende Triboni[c] and Jonathan W. Steed[a]*

a) Department of Chemistry, Durham University, South Road, Durham, DH1 3LE, UK
E-mail: jon.steed@durham.ac.uk

b) Università Degli Studi di Milano, Via Golgi, 19 - Corpo A, 20133 Milano, Italy.
Email: davide.proserpio@unimi.it

c) Escola de Engenhara de Lorena, Universidade de São Paulo, Estrada Municipal do Campinho, s/n – Pte. Nova, Lorena – SP, 12602-810, Brazil. Email: tribonier@usp.br

Contents

1. Materials and Characterisation 1
2. Synthesis 2
2.1 Synthesis of H_4BINDI 2
2.2 Synthesis of BINDI-ZnSC MOF 3
3. Characterisation Data 4
4. Discussion of previously reported Zn BINDI structures GOZLEQ and KEVKEE 10
5. References 10
6. CSD Search for BINDI MOF structures 11

1. Materials and Characterisation

All starting materials were purchased from Merck Life Sciences or Tokyo Chemical Industries and used without further purification. ^{1}H NMR spectra were obtained using the Bruker Avance III-HD-400 spectrometer with operating frequency of 400.07 MHz. MOF samples were prepared in 0.7 mL with a concentration of 1–3% (w/v). MOFs were dissolved by using the acid digestion method by adding a drop of HCl to a mixture of the MOFs in DMSO–d_6. FT-IR spectra were obtained using the PerkinElmer Spectrum 100 series spectrometer, fitted with a diamond universal Attenuated Total Reflectance (ATR) accessory. The spectra were collected in the range of 4000 cm^{-1} to 550 cm^{-1} at a resolution of 0.5 cm^{-1}. Elemental analysis was performed with the Exeter CE-440 Elemental Analyser and all analysed samples were dried prior to analysis. MOF crystals were dried using an Abderhalden drying pistol at 110°C for one

day prior to elemental analysis to ensure the removal of surface DMF. XRPD patterns were measured using the Bruker AXS D8 Advance in Bragg-Brentano geometry, equipped with a Lynxeye Soller PSD detector. Samples were measured on a Si low background sample holder and analysed using Cu-Kα radiation at a wavelength of 1.5406 Å. Samples were scanned over an angle range of 3-60° 2θ with a step size of 0.02° and a scan rate of 1s per step. TGA thermograms were obtained using the Perkin Elmer Pyris I thermogravimetric analyser and DSC was performed using the Q1000 DSC from TA Instruments. Samples were run from room temperature up to designated temperatures under nitrogen atmosphere. Elemental analysis was performed with the Exeter CE-440 Elemental Analyser. The X-ray single crystal data for H_4BINDI and BINDI-ZnSC (laboratory diffractometer) have been collected at a temperature of 120.0(2) K using MoKα radiation (λ = 0.71073 Å) on a Bruker D8 Venture with a Photon III MM C14 CPAD detector, IμS-III-microsource, focusing mirrors diffractometer equipped with a Cryostream (Oxford Cryosystems 700+) open-flow nitrogen cryostat. Data for poorly diffracting crystal BINDI-ZnSC (synchrotron) was obtained at 100.0(2) K at the I-19 beamline (Dectris Pilatus 2M pixel-array photon-counting detector, undulator, graphite monochromator, λ = 0.68890 Å) at the Diamond Light Source, Oxfordshire and processed using Xia2/DIALS[1,2]. The structures were solved using Olex2[3] with the ShelXT[4] structure solution program using Intrinsic Phasing and refined with the ShelXL[5] refinement package using Least Squares minimization on F^2. All non-hydrogen atoms were refined with anisotropic displacement parameters. Hydrogen atoms were located on the difference map and refined isotropically on a riding model unless otherwise specified. Crystallographic data for the structures have been deposited with the Cambridge Crystallographic Data Centre with deposition numbers CCDC-2470026-2470028 for H_4BINDI, BINDI-ZnSC (laboratory diffractometer) and BINDI-ZnSC (synchrotron) respectively. The topological analysis was undertaken using the software ToposPro.[6] All discussed materials were analysed using the “single node” and “all node” deconstruction methods as discussed in the work by Öhström and co-workers.[7]

2. Synthesis

2.1 Synthesis of H_4BINDI

5-Aminoisophthalic acid (0.760 g, 4.20 mmol) and 1,4,5,8-naphthalenetetracarboxylic acid (0.600 g, 2.00 mmol) were combined in DMF (20 mL). The mixture was heated to 110°C under reflux for 12 hours. After cooling down, 10 mL of deionized water was added to the resulting mixture to precipitate the product, which was subsequently filtered and washed with ethanol (3

× 20 mL). Single crystals of H_4BINDI were obtained by vapour diffusion of water into a DMSO solution. The material was characterised by FT-IR, elemental analysis, ^{1}H NMR and {^{1}H}^{13}C NMR spectroscopy and elemental analysis. Yield: 1.08g, 1.82 mmol, 91%.

^{1}H NMR (400 MHz, DMSO–d_6): δ = 13.51 (4H, s, COO**H**), 8.75 (4H, s, Ar**H**), 8.59 (2H, t, *J* = 1.6 Hz, Ar**H**), 8.35 (4H, d, *J* = 1.6 Hz, Ar**H**) ppm. {^{1}H}^{13}C NMR (400 MHz, DMSO–d_6): δ = 166.5, 163.5, 137.0, 134.9, 132.7, 130.8, 130.4, 127.7, 127.3 ppm. Elemental analysis calculated for $C_{30}H_{18}N_2O_4$. Expected C 57.15, H 2.88, N 4.44%. Found C 57.05, H 2.98, N 4.55%.

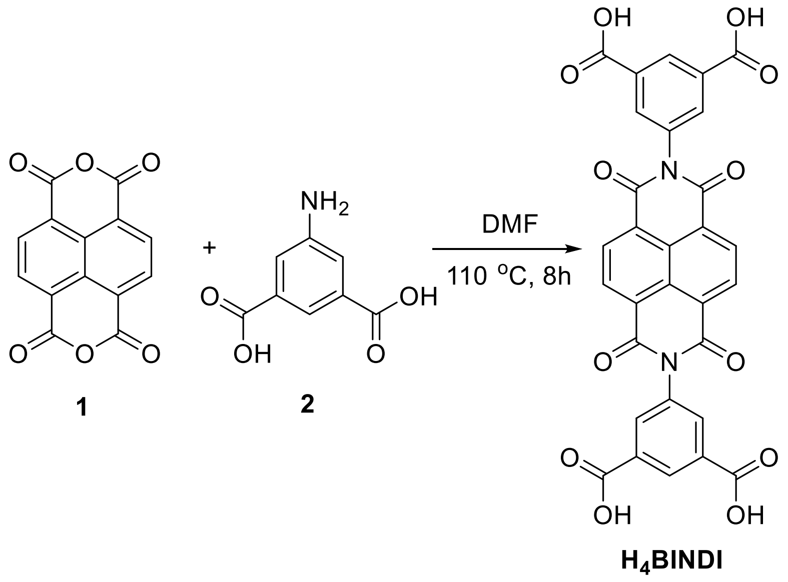

Figure S1. General synthetic procedure for the synthesis of H_4BINDI from **1** and **2**.

2.2 Synthesis of BINDI-ZnSC MOF

H_4BINDI (0.030 g, 0.050 mmol) and 2.0 mmol of zinc nitrate hexahydrate (0.59 g, 2.00 mmol) were dissolved in DMF (10 mL) in a 23 mL Teflon lined acid digestion vessel. To the resulting solution 0.1 mL of trifluoroacetic acid was added. The vessel was subsequently sealed and placed in an isothermal oven at 120°C for 72 hours. The solvothermal synthesis yields pink crystals amid semicrystalline material which were characterised by X-ray powder diffraction (XRPD), ^{1}H NMR spectroscopy, FT-IR. Additionally, the rhombic-shaped single crystals were studied *via* single crystal X-ray diffraction.

3. Characterisation Data

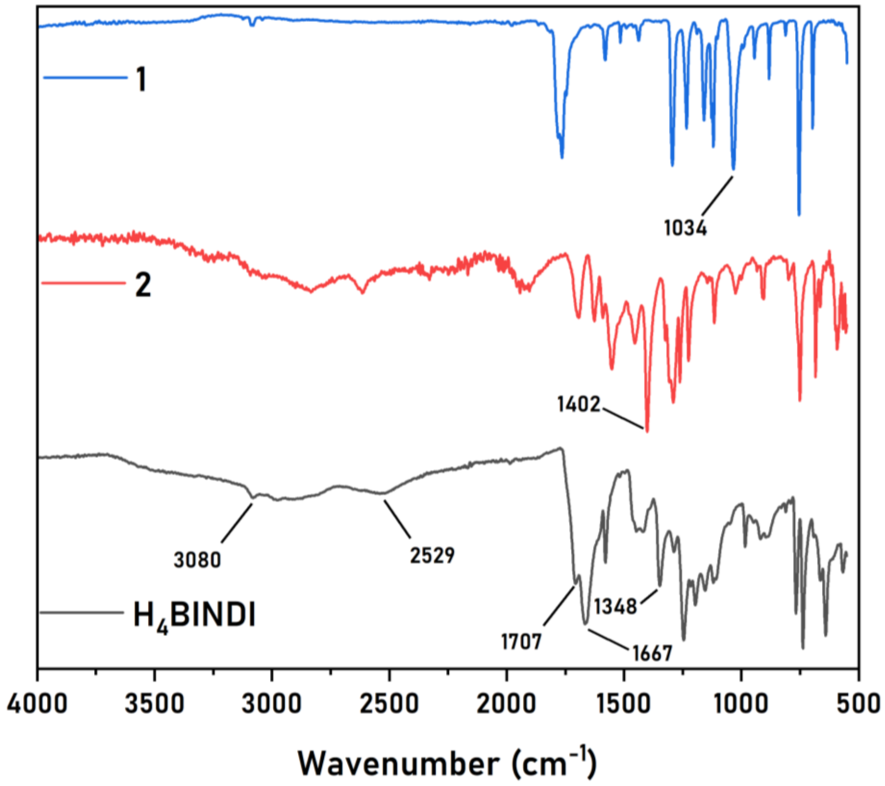

Figure S2. FT-IR spectra of **1** (blue line), **2** (red line) and H_4BINDI (black line).

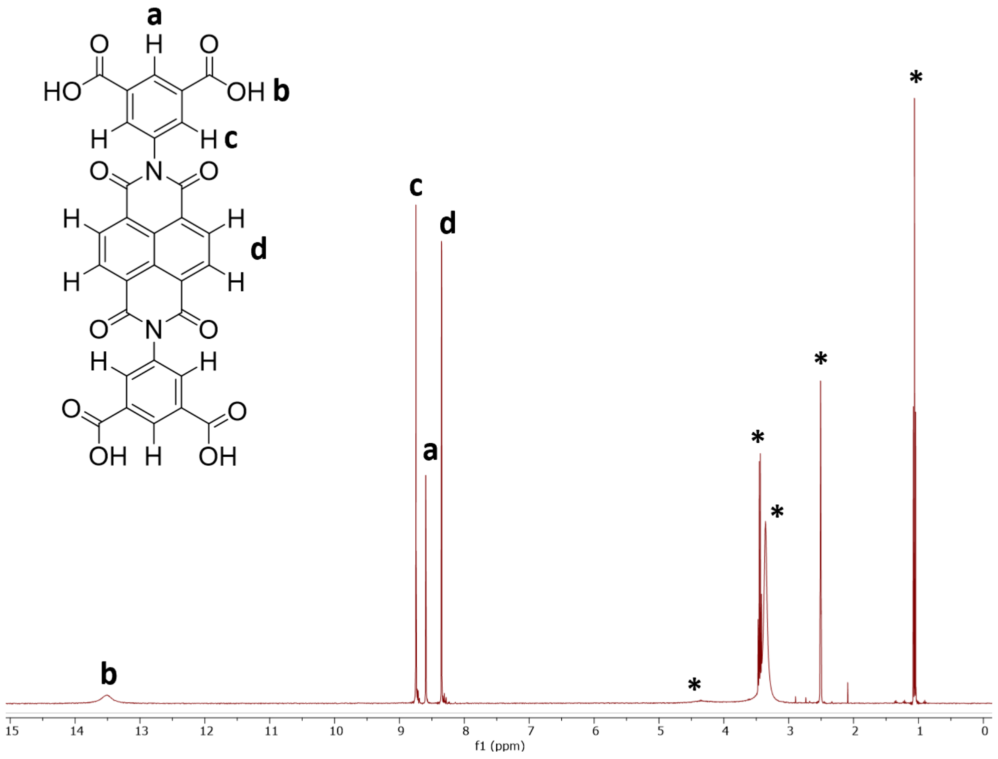

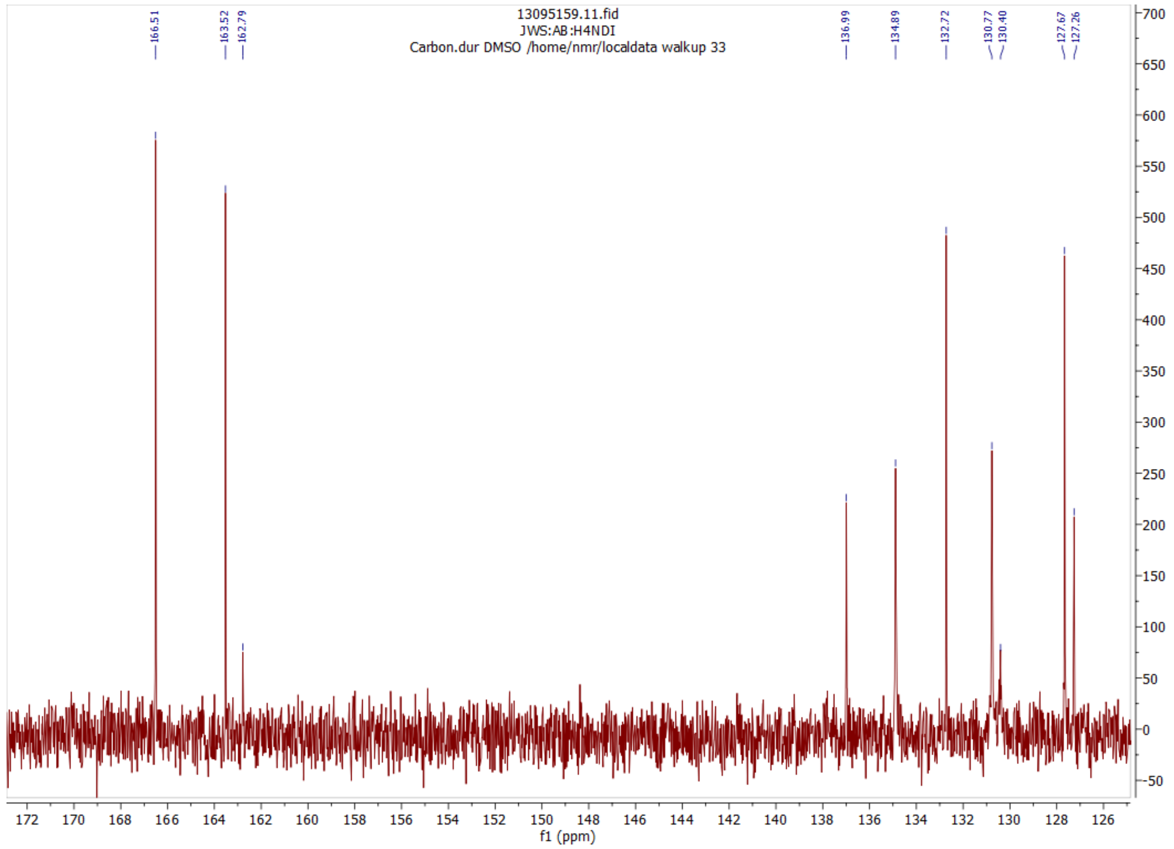

Figure S3. ^{1}H and ^{13}C NMR spectra of H_4BINDI. Spectrum acquired in d_6-DMSO. Solvent peaks in the ^{1}H spectrum have been labelled with an asterisk.

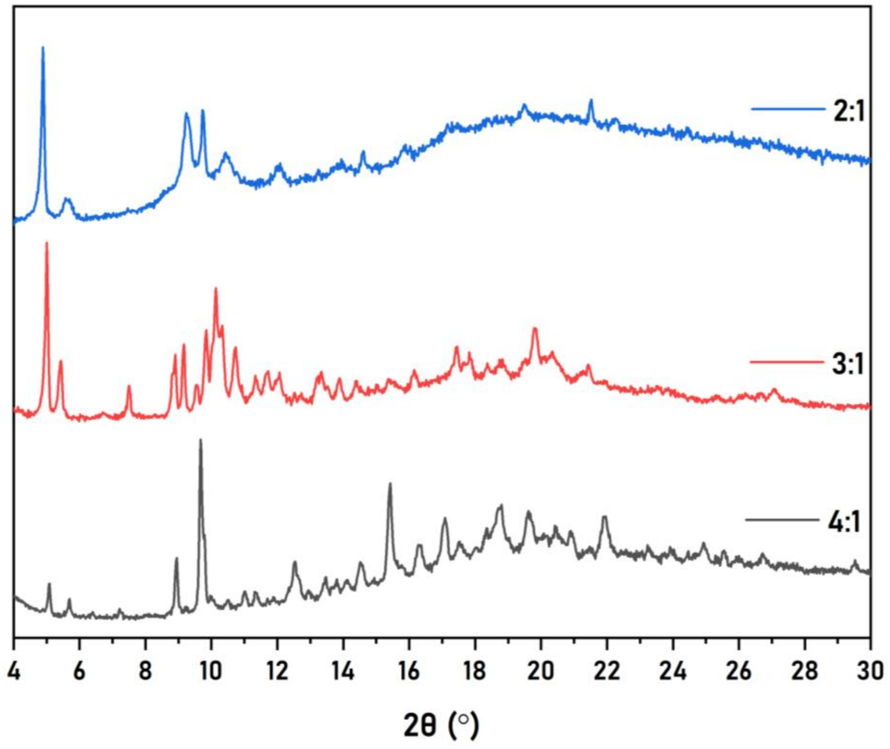

Figure S4. XRPD patterns of BINDI-ZnSC MOF reactions using ratios of 2:1 (blue trace), 3:1 (red trace) and 4:1 (black trace) metal:ligand.

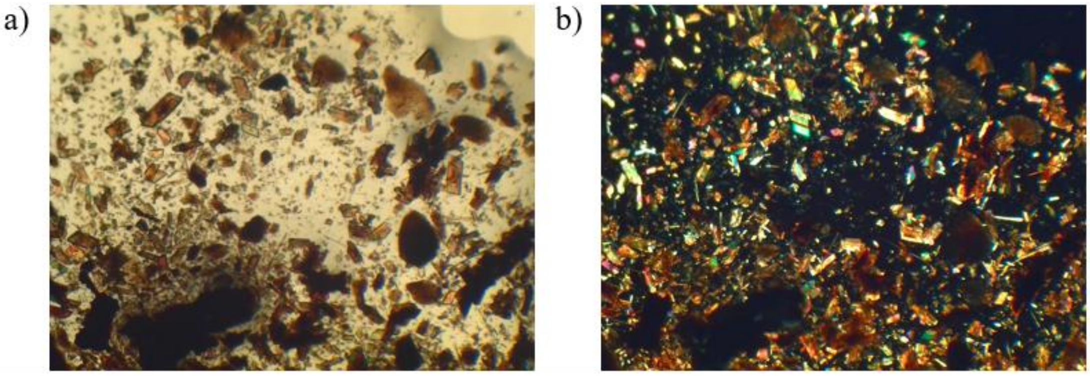

Figure S5. Optical microscopy images of single crystals of BINDI-ZnSC (4:1 reaction) along with the dark amorphous product.

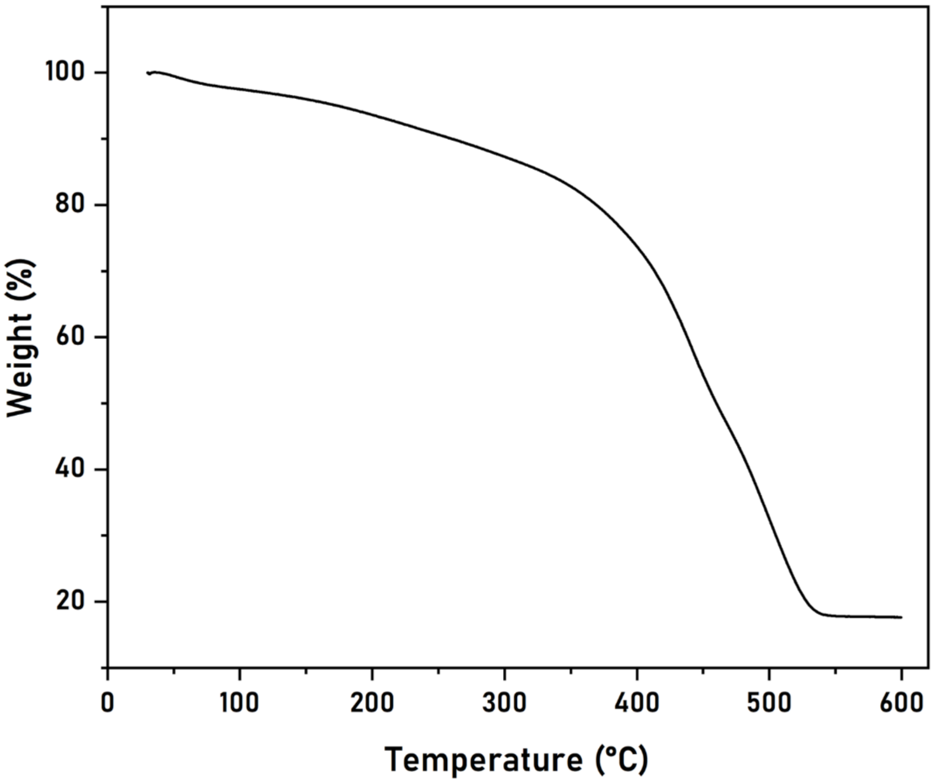

Figure S6. TGA thermogram of BINDI-ZnSC.

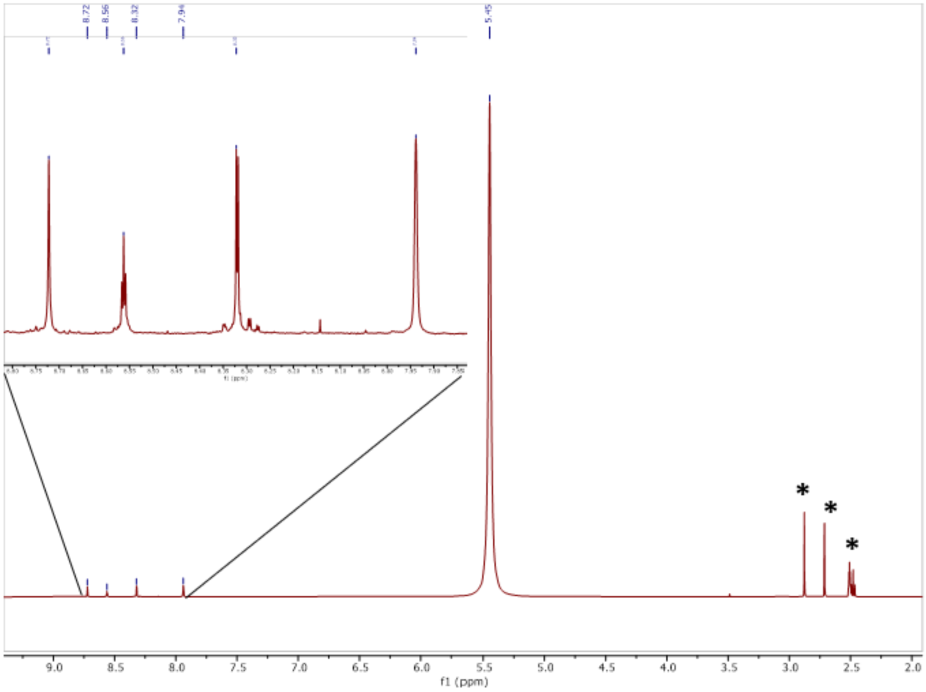

Figure S7. ^{1}H NMR spectrum of BINDI-ZnSC dissolved in d_6-DMSO with a drop of concentrated hydrochloric acid, including a zoom in of the region 7.94–8.72 ppm. Asterisks denote peaks attributed to solvent.

Table S1: Single crystal data for H_4BINDI.

Empirical formula	$C_{38}H_{38}N_2O_{16}S_4$
Formula weight	906.94
Temperature/K	120.00
Crystal system	monoclinic
Space group	P2/c
a/Å	22.1615(15)
b/Å	4.9703(4)
c/Å	44.997(3)
α/°	90
β/°	104.161(2)
γ/°	90
Volume/Å^3	4805.7(6)
Z	4
ρ_{calc}g/cm^3	1.469
μ/mm^{-1}	0.360
F(000)	2224.0
Crystal size/mm^3	0.21 × 0.03 × 0.02
Radiation	Mo Kα (λ = 0.71073)
2Θ range for data collection/°	3.734 to 51
Index ranges	-26 ≤ h ≤ 26, -6 ≤ k ≤ 5, -54 ≤ l ≤ 54
Reflections collected	78105
Independent reflections	8892 [R_{int} = 0.1435, R_{sigma} = 0.0854]
Data/restraints/parameters	8892/211/646
Goodness-of-fit on F^2	1.030
Final R indexes [I>=2σ (I)]	R_1 = 0.0977, wR_2 = 0.2414
Final R indexes [all data]	R_1 = 0.1582, wR_2 = 0.2788
Largest diff. peak/hole / e Å^{-3}	0.50/-0.59

Table S2: Single crystal data for BINDI-ZnSC (laboratory diffractometer).

Identification code	23srv149
Empirical formula	$C_{94.5}H_{106.5}N_{15.5}O_{37.5}Zn_3$
Formula weight	2255.56
Temperature/K	120.00
Crystal system	triclinic
Space group	P-1
a/Å	17.8650(10)
b/Å	19.6894(11)
c/Å	20.3421(12)
α/°	107.916(2)
β/°	113.737(2)
γ/°	97.408(2)
Volume/Å^3	5964.2(6)
Z	2
ρ_{calc}g/cm^3	1.256
μ/mm^{-1}	0.679
F(000)	2344.0

Crystal size/mm^3	0.173 × 0.088 × 0.051
Radiation	Mo Kα (λ = 0.71073)
2Θ range for data collection/°	3.888 to 41.63
Index ranges	-17 ≤ h ≤ 17, -19 ≤ k ≤ 19, -20 ≤ l ≤ 20
Reflections collected	101551
Independent reflections	12480 [R_{int} = 0.1006, R_{sigma} = 0.0602]
Data/restraints/parameters	12480/1465/845
Goodness-of-fit on F^2	1.061
Final R indexes [I>=2σ (I)]	R_1 = 0.0651, wR_2 = 0.1898
Final R indexes [all data]	R_1 = 0.0982, wR_2 = 0.2133
Largest diff. peak/hole / e Å^{-3}	0. 50/-0.41

Table S3: Single crystal data of BINDI-ZnSC (synchrotron).

Identification code	23srv149_2
Empirical formula	$C_{79.5}H_{71.5}N_{10.5}O_{32.5}Zn_3$
Formula weight	1888.06
Temperature/K	100.0(2)
Crystal system	triclinic
Space group	P-1
a/Å	17.8760(2)
b/Å	19.7569(3)
c/Å	20.3741(5)
α/°	107.475(2)
β/°	113.9320(10)
γ/°	97.1720(10)
Volume/Å^3	6017.1(2)
Z	2
ρ_{calc}g/cm^3	1.042
μ/mm^{-1}	0.608
F(000)	1940.0
Crystal size/mm^3	0.086 × 0.02 × 0.004
Radiation	Synchrotron (λ = 0.6889)
2Θ range for data collection/°	2.298 to 51.006
Index ranges	-22 ≤ h ≤ 22, -24 ≤ k ≤ 24, -25 ≤ l ≤ 25
Reflections collected	82289
Independent reflections	24435 [R_{int} = 0.1177, R_{sigma} = 0.1812]
Data/restraints/parameters	24435/1463/846
Goodness-of-fit on F^2	0.935
Final R indexes [I>=2σ (I)]	R_1 = 0.0816, wR_2 = 0.2380
Final R indexes [all data]	R_1 = 0.1392, wR_2 = 0.2648
Largest diff. peak/hole / e Å^{-3}	1.07/-0.53

4. Discussion of previously reported Zn BINDI structures GOZLEQ and KEVKEE

In addition to structure FOMWOV two further zinc(II) containing MOF structures derived from the deprotonated H_4BINDI ligand have been reported. The formulae assigned by the authors are $[Zn(BINDI)_{0.25}{\cdot}(C_2H_5NO)]_n$ (CSD refcode GOZLEQ[8]) and $[Zn_2(BINDI)(DMA)_2]{\cdot}2DMA$ (CSD refcode KEVKEE[9])

The GOZLEQ and KEVKEE structures are isoreticular and also have almost the same unit cell but two different space groups *Imma* and *Pbnm* (a subroup of *Imma*). Both have a topology in the single node method of 4-c lvt and in the all node method 3,4-c lil. A Platon check of KEVKEE shows that the space group is likely to be incorrect and application of CALC ADDSYM results in *Imma*. As a result, it seems likely that these are isostructural. While the formula given for KEVKEE is $[Zn_2(BINDI)(DMA)_2]{\cdot}2DMA$ the crystal structure coordinates appear to show a linear CH_3NCO type of unit which perhaps represents unresolved DMF disorder. The formula of $[Zn(BINDI)_{0.25}{\cdot}(C_2H_5NO)]_n$ for GOZLEQ explicitly includes this anion which is required for charge balance. The origin of this species, which is presumably a DMF degradation product is unclear and not discussed by the original authors.

5. References

(1) G. Winter, *J. Appl. Cryst.,* 2010, **43**, 186-190.

(2) G. Winter, D. G. Waterman, J. M. Parkhurst, A. S. Brewster, R. J. Gildea, M. Gerstel, L. Fuentes-Montero, M. Vollmar, T. Michels-Clark, I. D. Young, N. K. Sauter, G. Evans, *Acta Crystallogr.,* D, 2018, **74**, 85-97.

(3) O. V. Dolomanov, L. J. Bourhis, R. J. Gildea, J. A. K. Howard and H. Puschmann, *J Appl Crystallogr*, 2009, **42**, 339–341.

(4) Sheldrick, G.M. *Acta Crystallogr*. 2015, **A71**, 3–8.

(5) G. M. Sheldrick, *Acta Crystallogr., Sect. C: Struct. Chem.*, 2015, **71**, 3–8.

(6) V. A. Blatov, A. P. Shevchenko and D. M. Proserpio, *Cryst. Growth Des.*, 2014, **14**, 3576–3586.

(7) C. Bonneau, M. O'Keeffe, D. M. Proserpio, V. A. Blatov, S. R. Batten, S. A. Bourne, M. S. Lah, J. G. Eon, S. T. Hyde, S. B. Wiggin and L. Öhrström, *Cryst. Growth Des.*, 2018, **18**, 3411–3418.

(8) Mao, Y.; Shan, B.; Yu, J.; Liu, X.; Chu, J.; Ma, X.; Li, M.; Zheng, Y.; Zhu, B.; Zuo, M.; Shuxin, C., A novel naphthalene diimide-based compound:synthesis, photochromism, ammonia vapor and triethylamine detection. *J. Solid State Chem.* **2024**, *338*, 124902.

(9) Xu, H.-L.; Zeng, X.-S.; Li, J.; Xu, Y.-C.; Qiu, H.-J.; Xiao, D.-R., The impact of metal ions on photoinduced electron-transfer properties: four photochromic metal–organic frameworks based on a naphthalenediimide chromophore. *CrystEngComm* **2018**, *20*, 2430-2439.

6. CSD Search for BINDI MOF structures

Search Overview

Search:	search2
Date/Time done:	Mon Jun 30 12:33:47 2025
Database(s):	CSD version 6.00 (Apr 2025)
Restriction Info:	No refcode restrictions applied
Filters:	None
Percentage Completed:	100%
Number of Hits:	107

Single query used. Search found structures that:

match

Query 1

Query 1

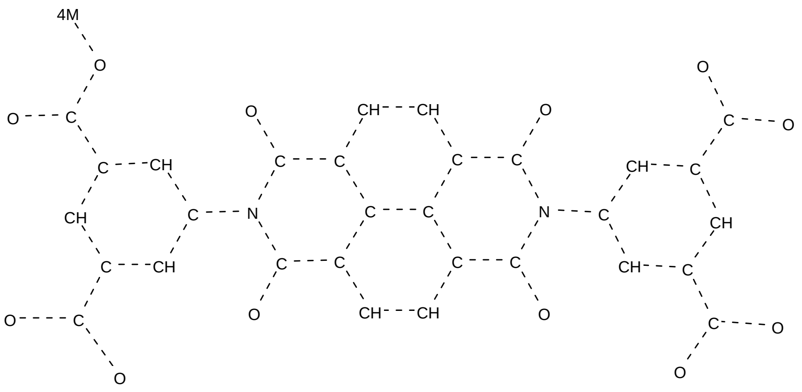

Search: search2 (Mon Jun 30 12:33:47 2025): Hit 1

ABUJIU

Reference: Le Zeng, Ling Huang, Zhonghe Wang, Jianwei Wei, Kai Huang, Wenhai Lin, Chunying Duan, Gang Han (2021) *Angew.Chem.,Int.Ed.* ,**60,**23569

Formula: $(C_{66}\ H_{34}\ Ca_4\ N_6\ O_{26})n,C_{16}\ H_{10}$

Compound Name: catena-[bis(μ-5,5'-(1,3,6,8-tetraoxo-1,3,6,8-tetrahydrobenzo[lmn][3,8]phenanthroline-2,7-diyl)bis(benzene-1,3-dicarboxylato))-bis(N,N-dimethylformamide)-tetra-calcium pyrene clathrate unknown solvate]

Synonym: Py@Ca-NDI

Space Group:	C2/c	***Cell:***	***a***	41.973(4)	***b***	10.404(0)	***c***	24.827(3)
Space Group No.:	15	***(Å,°)***	α	90.00	β	126.22(0)	γ	90.00

R-Factor (%): 9.10 ***Temperature(K):*** 150 ***Density(g/cm³):*** 1.283

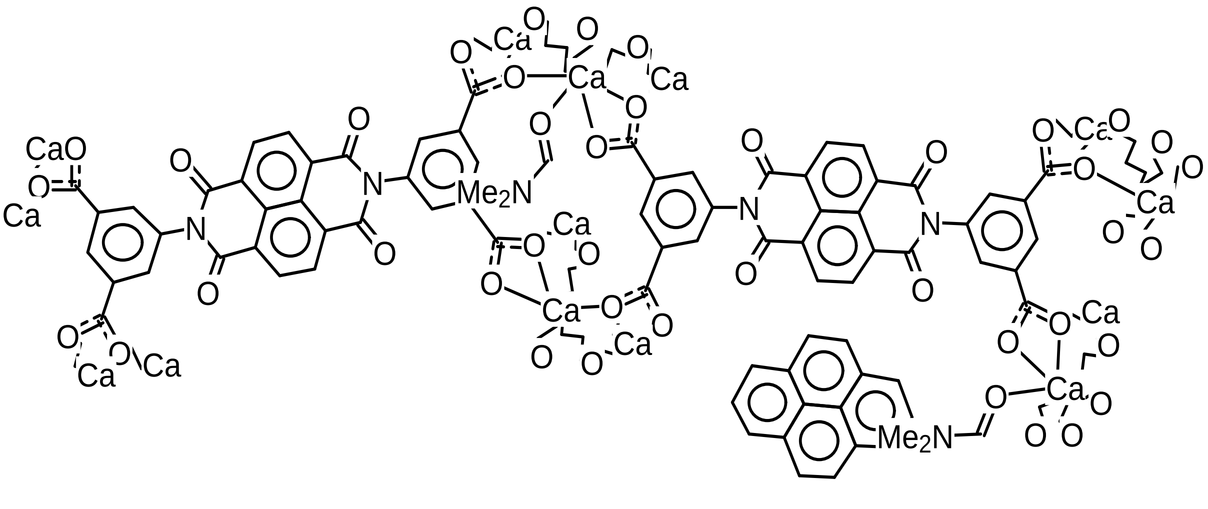

Search: search2 (Mon Jun 30 12:33:47 2025): Hit 2

ANOMOG

Reference:	J.A.Perman, A.J.Cairns, L.Wojtas, M.Eddaoudi, M.J.Zaworotko (2011) *CrystEngComm* ,**13,**3130
Formula:	$(C_{90}\ H_{42}\ Cu_6\ N_6\ O_{42})n$
Compound Name:	catena-(tris(μ_8-5,5'-(1,3,6,8-tetraoxobenzo[lmn][3,8]phenanthroline-2,7-diyl)bis(benzene-1,3-dicarboxylato))-hexaaqua-hexa-copper unknown solvate)

Space Group:	C2/m	***Cell:***	***a***	32.461(12)	***b***	18.024(6)	***c***	19.748(7)
Space Group No.:	12	***(Å,°)***	α	90.00	β	119.96(0)	γ	90.00

R-Factor (%):	8.19	***Temperature(K):***	100	***Density(g/cm^3):***	0.750

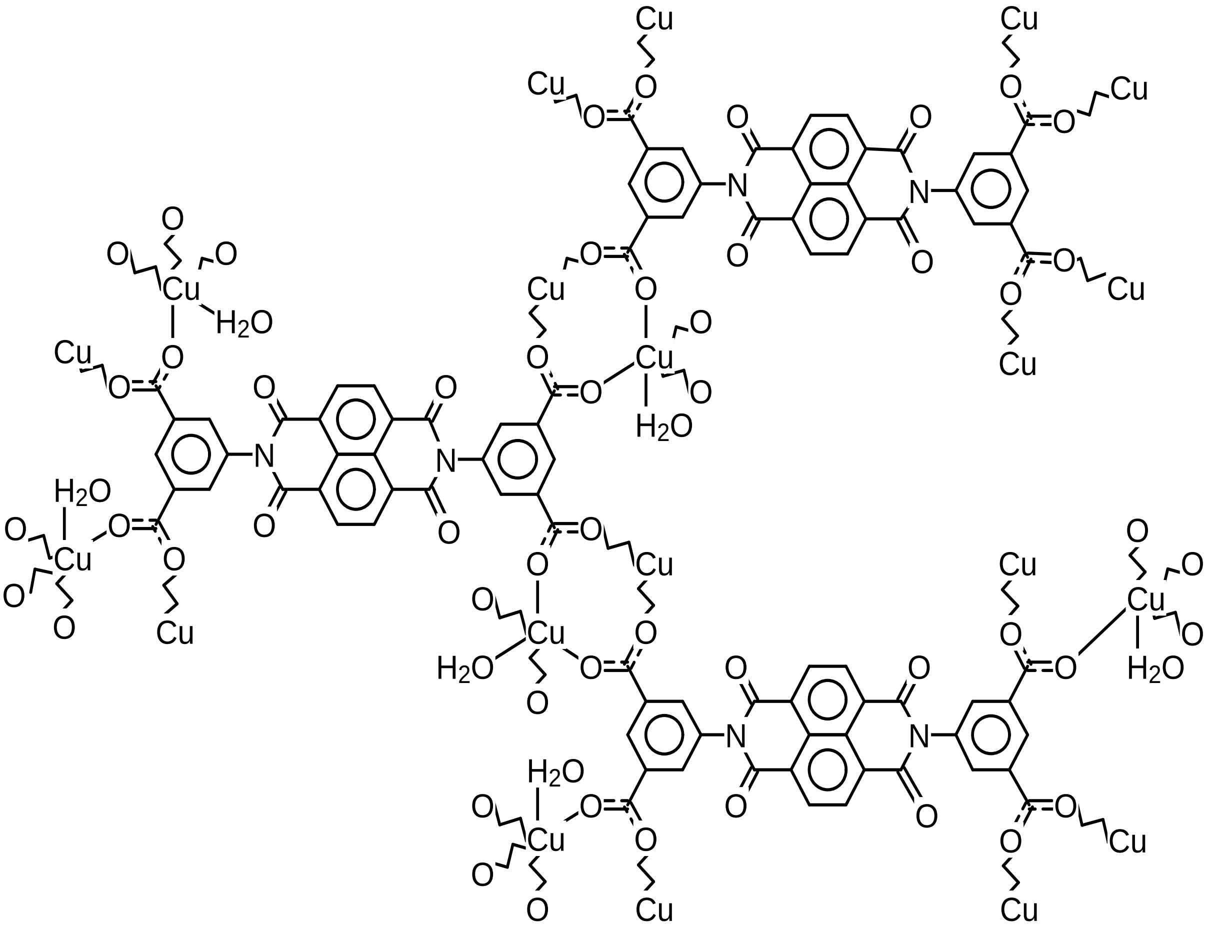

Search: search2 (Mon Jun 30 12:33:47 2025): Hit 3

ANOMUM

Reference: J.A.Perman, A.J.Cairns, L.Wojtas, M.Eddaoudi, M.J.Zaworotko (2011) *CrystEngComm* ,**13,**3130

Formula: $(C_{38}\ H_{28}\ Cu_2\ N_4\ O_{14})n$

Compound Name: catena-((μ_8-5,5'-(1,3,6,8-tetraoxobenzo[lmn][3,8]phenanthroline-2,7-diyl)bis(benzene-1,3-dicarboxylato))-bis(N,N-dimethylacetamide)-di-copper unknown solvate)

Space Group:	Imma	***Cell:***	***a***	15.225(0)	***b***	36.439(0)	***c***	10.304(0)
Space Group No.:	74	***(Å,°)***	α	90.00	β	90.00	γ	90.00

R-Factor (%): 3.75 ***Temperature(K):*** 100 ***Density(g/cm³):*** 1.036

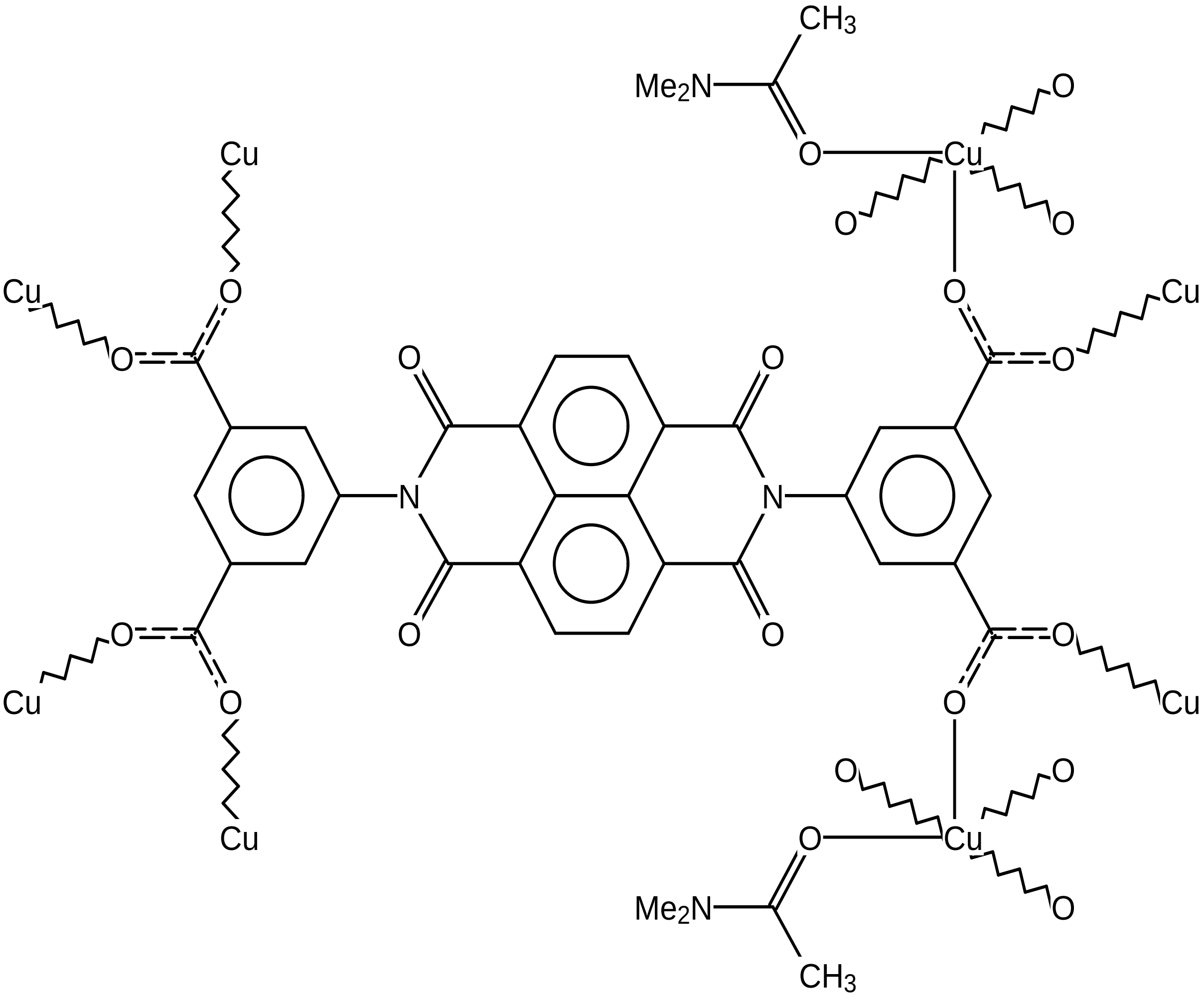

Search: search2 (Mon Jun 30 12:33:47 2025): Hit 4

ANONAT

Reference:	J.A.Perman, A.J.Cairns, L.Wojtas, M.Eddaoudi, M.J.Zaworotko (2011) *CrystEngComm* ,**13,**3130
Formula:	$(C_{44} H_{28} Cu_2 N_4 O_{12})n$
Compound Name:	catena-((μ_8-5,5'-(1,3,6,8-tetraoxobenzo[lmn][3,8]phenanthroline-2,7-diyl)bis(benzene-1,3-dicarboxylato))-bis(3,5-dimethylpyridine)-di-copper unknown solvate)

Space Group:	Imma	***Cell:***	***a*** 14.981(2)	***b*** 36.371(5)	***c*** 10.922(2)
Space Group No.:	74	***(Å,°)***	α 90.00	β 90.00	γ 90.00
R-Factor (%):	4.29	***Temperature(K):***	183	***Density(g/cm³):***	1.040

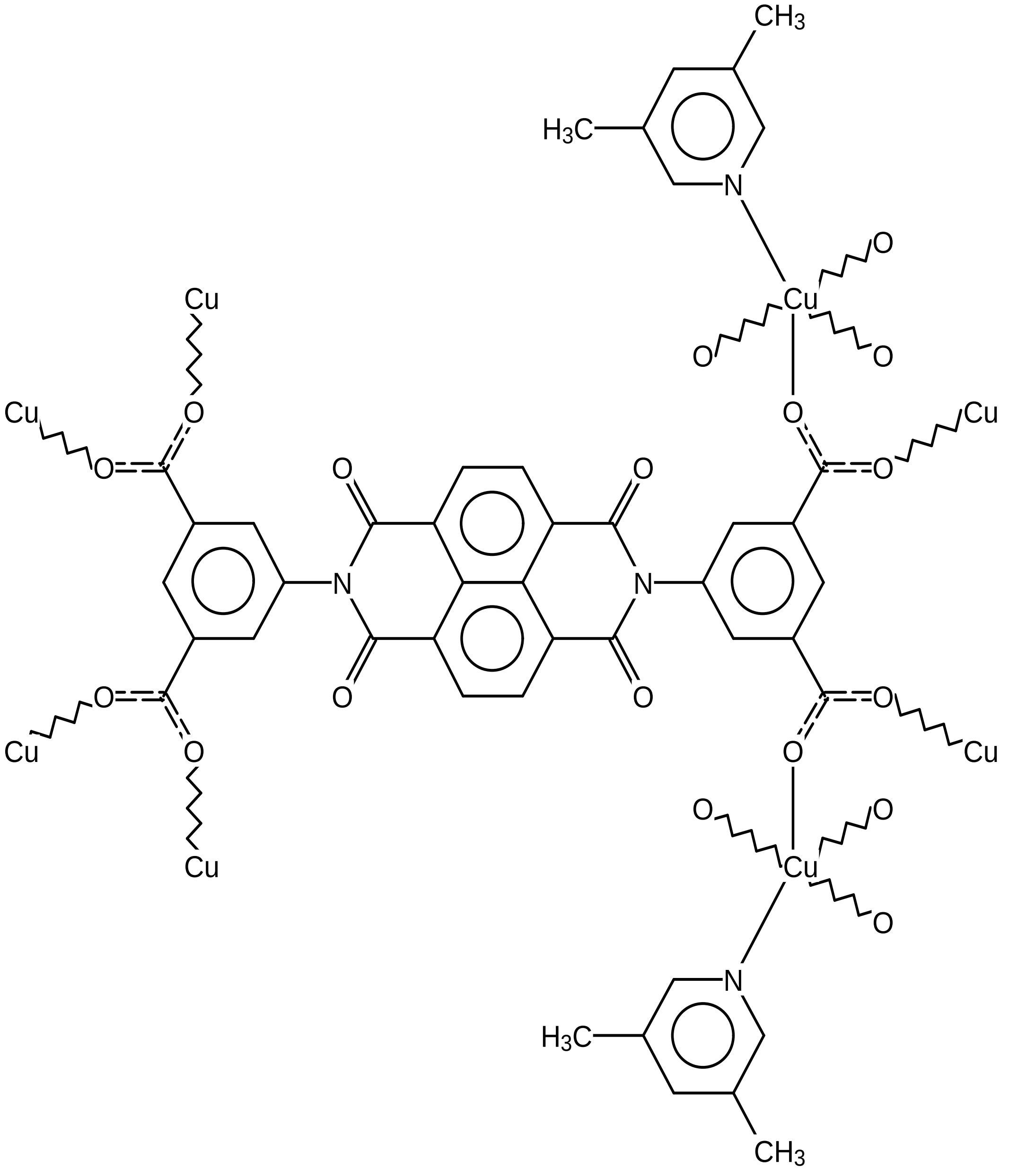

Search: search2 (Mon Jun 30 12:33:47 2025): Hit 5

ARALAJ

Reference: wen dai (2021)
CSD Communication(Private Communication) ,

Formula: $(C_{46} H_{44} N_6 O_{20} Pr_2)n,2(C_3 H_7 N_1 O_1),H_2 O_1$

Compound Name: catena-[(μ-5,5'-(1,3,6,8-tetraoxo-1,3,6,8-tetrahydrobenzo[lmn][3,8]phenanthroline-2,7-diyl)di(benzene-1,3-dicarboxylato))-bis(μ-acetato)-tetrakis(N,N-dimethylformamide)-di-praseodymium N,N-dimethylformamide solvate monohydrate]

Space Group:	P-1	***Cell:***	***a***	10.281(1)	***b***	12.858(1)	***c***	13.183(1)
Space Group No.:	2	***(Å,°)***	α	76.73(0)	β	70.11(0)	γ	88.78(0)

R-Factor (%): 7.90 ***Temperature(K):*** 293 ***Density(g/cm³):*** 1.509

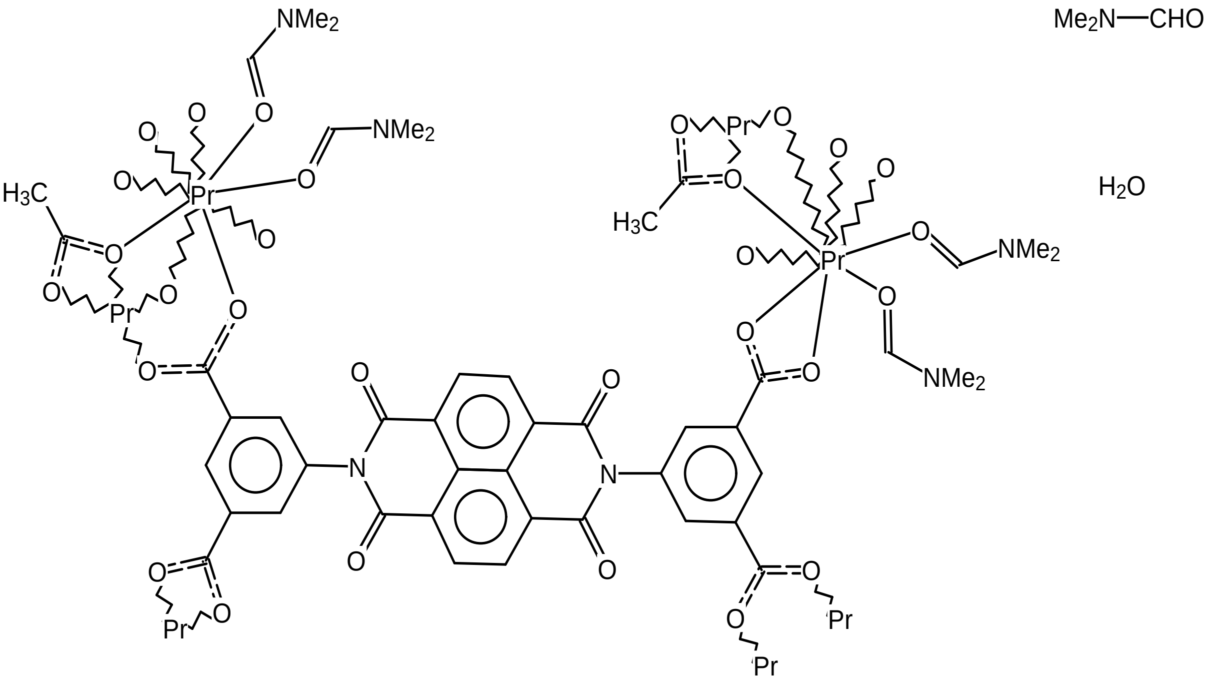

Search: search2 (Mon Jun 30 12:33:47 2025): Hit 6

BODCOQ

Reference:	Gui Xiong, Wangfu Xu, Lijuan Liang, Kun Huang, Xiangyu Zhang, Dabin Qin (2024) *J.Mol.Struct.* ,**1303,**137538
Formula:	$(C_{54} H_{26} N_{14} Ni_2 O_{12} S_2)n,2(H_2 O_1)$
Compound Name:	catena-[(μ-5,5'-(1,3,6,8-tetraoxo-1,3,6,8-tetrahydrobenzo[lmn][3,8]phenanthroline-2,7-diyl)bis(benzene-1,3-dicarboxylato))-bis(μ-4,7-bis(1H-imidazol-1-yl)-2,1,3-benzothiadiazole)-di-nickel dihydrate]

Space Group:	P-1	***Cell:***	***a***	10.086(4)	***b***	10.187(4)	***c***	13.565(4)
Space Group No.:	2	***(Å,°)***	α	69.91(1)	β	81.86(1)	γ	73.69(1)

R-Factor (%): 4.22 ***Temperature(K):*** 300 ***Density(g/cm³):*** 1.695

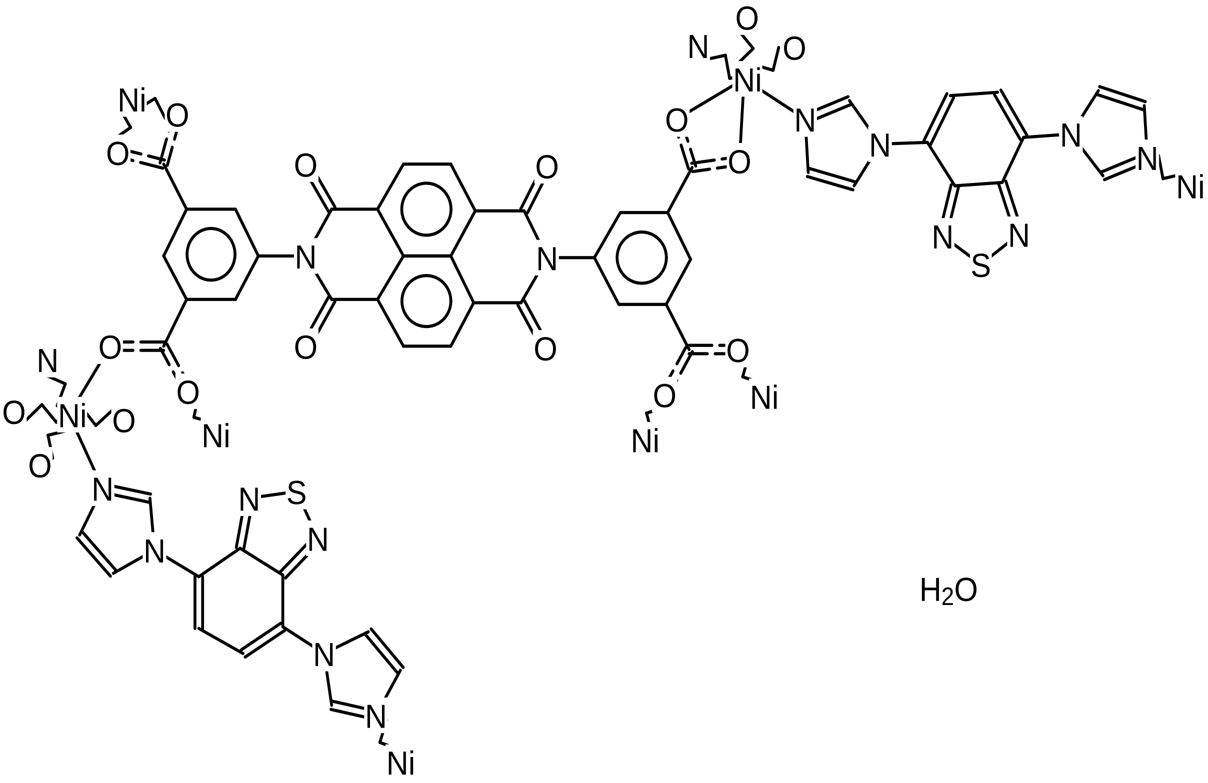

Search: search2 (Mon Jun 30 12:33:47 2025): Hit 7

COQTEJ

Reference:	A.Mallick, B.Garai, M.A.Addicoat, P.St.Petkov, T.Heine, R.Banerjee (2015) *Chemical Science* ,**6,**1420
Formula:	$(C_{72}\ H_{52}\ Mg_4\ N_8\ O_{30})n$
Compound Name:	catena-[bis(μ-5,5'-(1,3,6,8-tetraoxo-1,3,6,8-tetrahydrobenzo[lmn][3,8]phenanthroline-2,7-diyl)diisophthalato)-tetrakis(N,N-dimethylformamide)-diaqua-tetra-magnesium]

Space Group:	P2/c	***Cell:***	***a***	34.341(1)	***b***	10.045(0)	***c***	17.738(1)
Space Group No.:	13	***(Å,°)***	α	90.00	β	96.23(0)	γ	90.00

R-Factor (%):	10.57	***Temperature(K):***	293	***Density(g/cm³):***	0.877

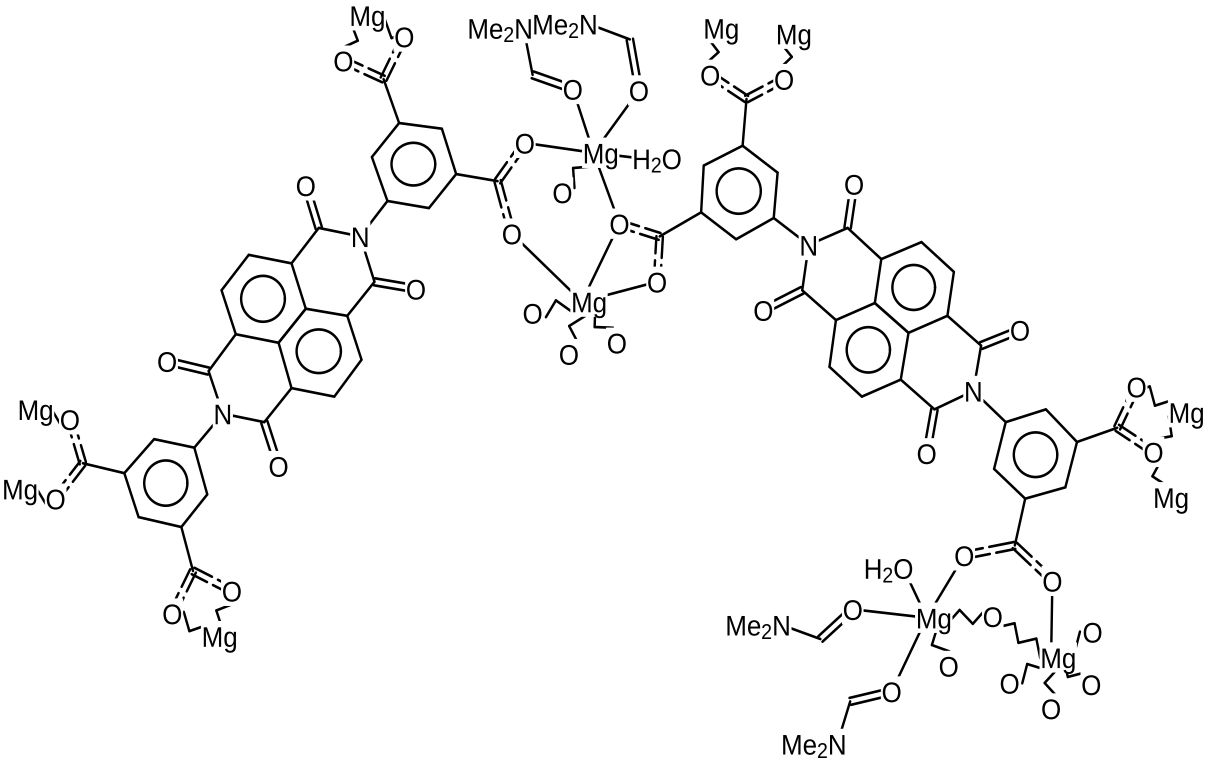

Search: search2 (Mon Jun 30 12:33:47 2025): Hit 8

EZAKUO

Reference:	wen dai (2021)<br>*CSD Communication(Private Communication) ,*
Formula:	$(C_{58}\ H_{32}\ N_6\ O_{16}\ Pr_2)n, C_3\ H_7\ N_1\ O_1, H_2\ O_1$
Compound Name:	catena-[(μ-5,5'-(1,3,6,8-tetraoxo-1,3,6,8-tetrahydrobenzo[lmn][3,8]phenanthroline-2,7-diyl)bis(benzene-1,3-dicarboxylato))-bis(μ-acetato)-bis(1,10-phenanthroline)-di-praseodymium(iii) N,N-dimethylformamide unknown solvate monohydrate]
Synonym:	FJU-211

Space Group:	P-1	***Cell:***	***a*** 9.825(0)	***b*** 12.753(0)	***c*** 16.659(0)		
Space Group No.:	2	***(Å,°)***	α 85.41(0)	β 77.55(0)	γ 81.44(0)		

R-Factor (%):	4.67	***Temperature(K):***	293	***Density(g/cm³):***	1.189

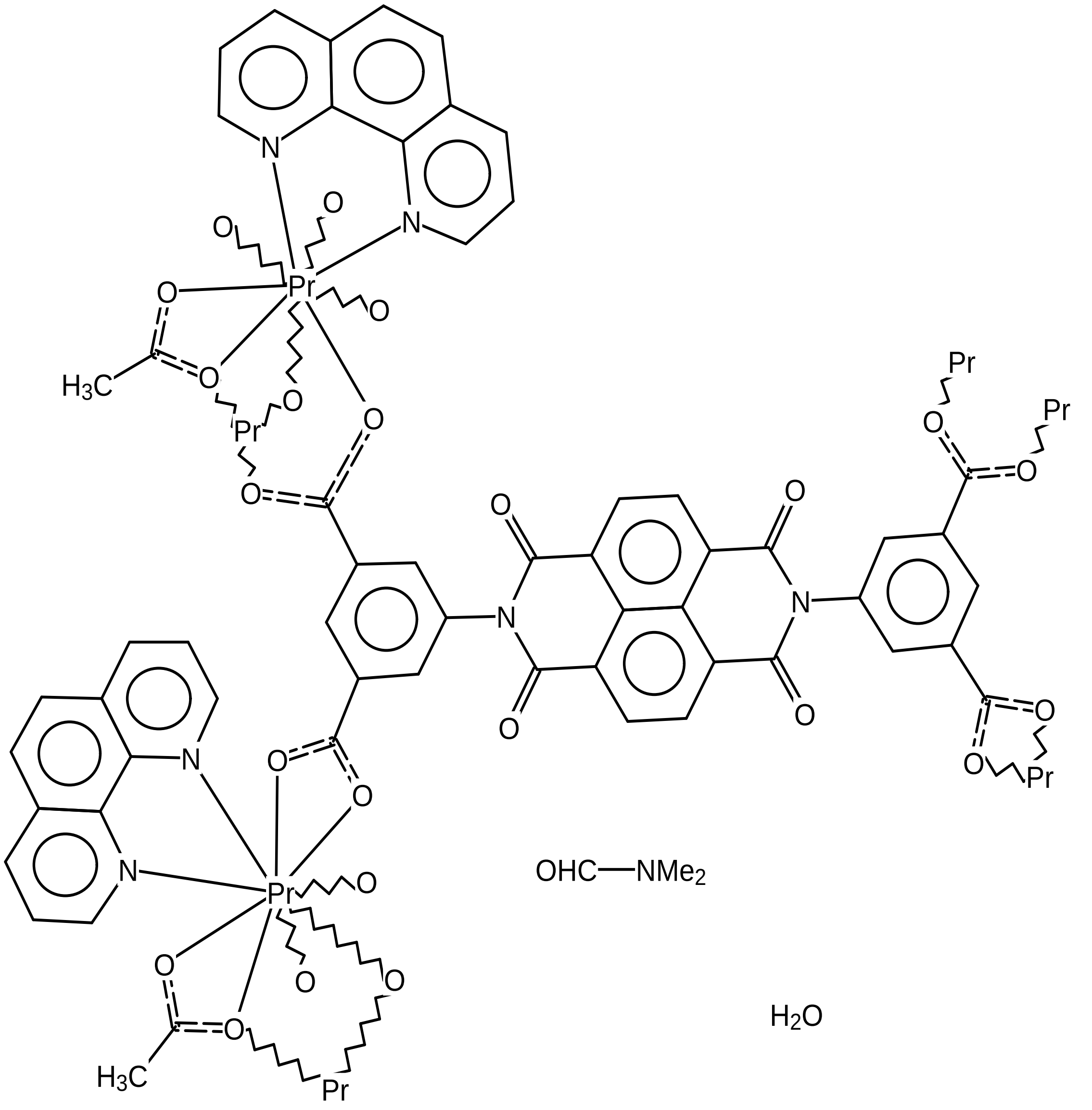

Search: search2 (Mon Jun 30 12:33:47 2025): Hit 9

EZAKUO01

Reference:	wen dai (2021)<br>*CSD Communication(Private Communication) ,*
Formula:	$(C_{58}\ H_{32}\ N_6\ O_{16}\ Pr_2)n,C_3\ H_7\ N_1\ O_1,H_2\ O_1$
Compound Name:	catena-[(μ-5,5'-(1,3,6,8-tetraoxo-1,3,6,8-tetrahydrobenzo[lmn][3,8]phenanthroline-2,7-diyl)di(benzene-1,3-dicarboxylato))-bis(μ-acetato)-bis(1,10-phenanthroline)-di-praseodymium N,N-dimethylformamide solvate monohydrate]
Synonym:	FJU-211

Space Group:	P-1	***Cell:***	***a***	9.931(1)	***b***	12.792(1)	***c***	17.070(2)
Space Group No.:	2	***(Å,°)***	α	84.12(1)	β	76.77(1)	γ	81.91(1)

R-Factor (%):	6.04	***Temperature(K):***	293	***Density(g/cm^3):***	1.149

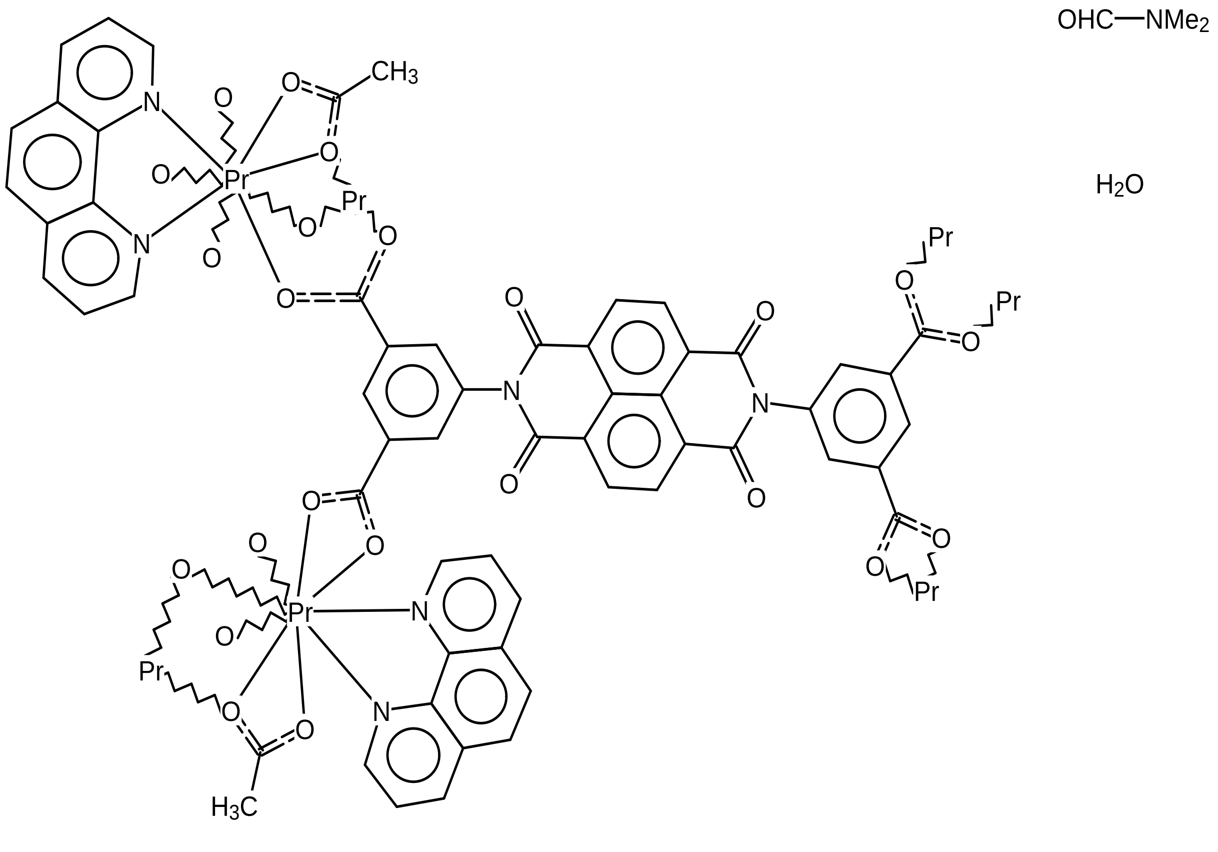

Search: search2 (Mon Jun 30 12:33:47 2025): Hit 10

FEDRAL

Reference: Zhenghan Jiang, Lingling Gao, Yujuan Zhang, Tuoping Hu
(2022) *Inorg.Chem.Commun.* ,**141,**109534

Formula: $(C_{74}\ H_{42}\ Co_2\ N_6\ O_{12})n,4(C_2\ H_3\ N_1)$

Compound Name: catena-[(μ-5,5'-(1,3,6,8-tetraoxo-1,3,6,8-tetrahydrobenzo[lmn][3,8]phenanthroline-2,7-diyl)bis(benzene-1,3-dicarboxylato))-bis(μ-4,4'-([1,1'-biphenyl]-4,4'-diyl)dipyridine)-di-cobalt(ii) acetonitrile solvate]

Space Group:	P-1	***Cell:***	***a*** 9.331(1)	***b*** 10.169(1)	***c*** 17.624(3)
Space Group No.:	2	***(Å,°)***	α 99.66(0)	β 90.85(0)	γ 100.31(0)

R-Factor (%): 4.81 ***Temperature(K):*** 296 ***Density(g/cm³):*** 1.526

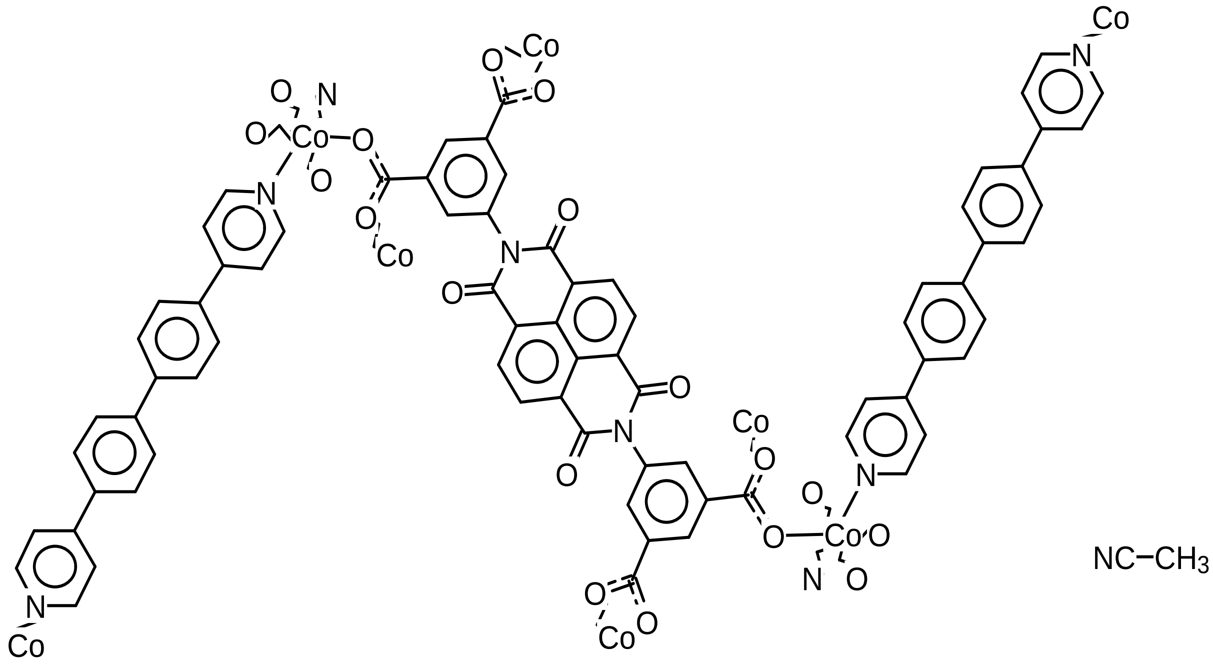

Search: search2 (Mon Jun 30 12:33:47 2025): Hit 11

FEDREP

Reference:	Zhenghan Jiang, Lingling Gao, Yujuan Zhang, Tuoping Hu<br>(2022) *Inorg.Chem.Commun.* ,**141,**109534
Formula:	$(C_{74} H_{42} Mn_2 N_6 O_{12})n$
Compound Name:	catena-[(μ-5,5'-(1,3,6,8-tetraoxo-1,3,6,8-tetrahydrobenzo[lmn][3,8]phenanthroline-2,7-diyl)bis(benzene-1,3-dicarboxylato))-bis(μ-4,4'-([1,1'-biphenyl]-4,4'-diyl)dipyridine)-di-manganese(ii) unknown solvate]

Space Group:	P-1	***Cell:***	***a*** 9.533(1)	***b*** 10.239(1)	***c*** 17.732(3)
Space Group No.:	2	***(Å,°)***	α 100.06(1)	β 90.42(1)	γ 100.16(1)

R-Factor (%): 6.58 ***Temperature(K):*** 296 ***Density(g/cm³):*** 1.305

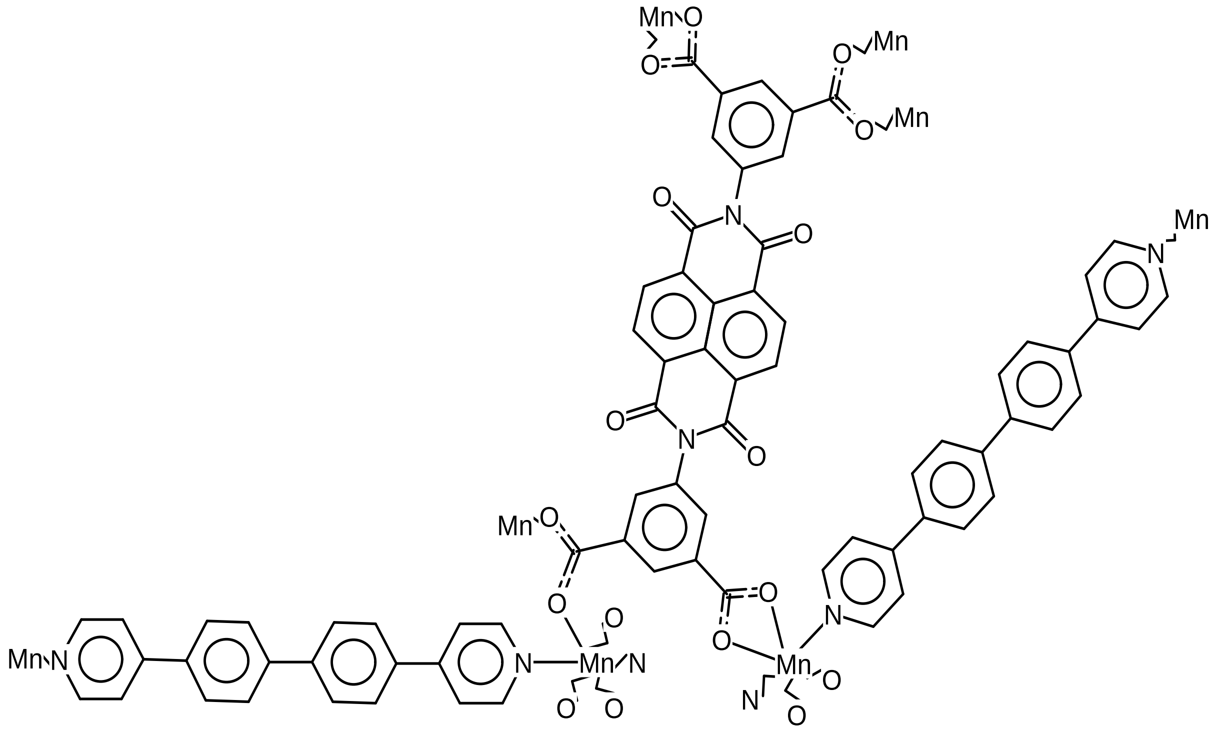

Search: search2 (Mon Jun 30 12:33:47 2025): Hit 12

FEMPAS

Reference:	Xiao-Feng Zhong, Guo-Jun Luo, Wen-Bin Li, Xiong-Hai Chen, Ying Wu, Yi-Hui Chen, Jia-Wen Ye, Jie Bai, Zong-Wen Mo, Xiao-Ming Chen (2022) *Dalton Trans.* ,**51,**14852
Formula:	$(C_{72} H_{52} La_2 N_8 O_{29})n,2(C_3 H_7 N_1 O_1)$
Compound Name:	catena-[bis(μ-5,5'-(1,3,6,8-tetraoxo-1,3,6,8-tetrahydrobenzo[lmn][3,8]phenanthroline-2,7-diyl)bis(benzene-1,3-dicarboxylato))-tetrakis(N,N-dimethylformamide)-aqua-di-lanthanum(iii) N,N-dimethylformamide solvate]

Space Group:	P-1	***Cell:***	***a*** 14.802(0)	***b*** 15.759(0)	***c*** 18.683(0)
Space Group No.:	2	***(Å,°)***	α 104.48(0)	β 97.44(0)	γ 95.26(0)

R-Factor (%):	5.23	***Temperature(K):***	298	***Density(g/cm^3):***	1.535

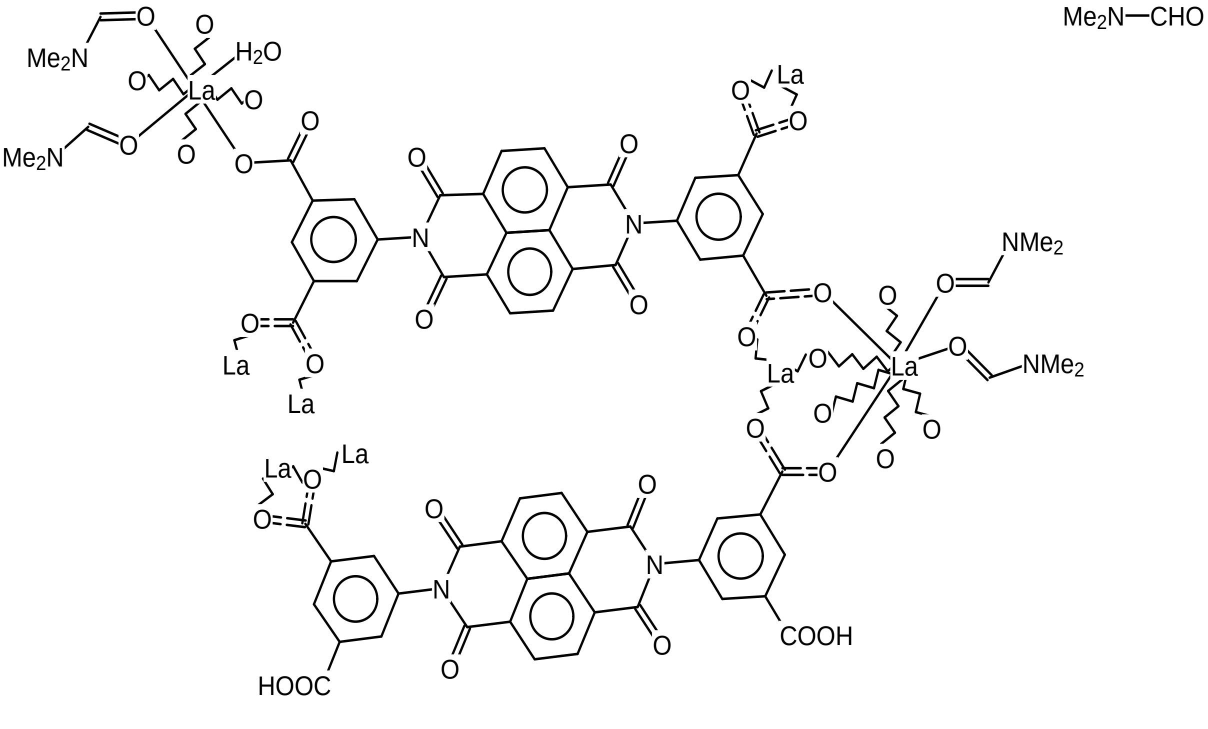

Search: search2 (Mon Jun 30 12:33:47 2025): Hit 13

FEMPEW

Reference: Xiao-Feng Zhong, Guo-Jun Luo, Wen-Bin Li, Xiong-Hai Chen, Ying Wu, Yi-Hui Chen, Jia-Wen Ye, Jie Bai, Zong-Wen Mo, Xiao-Ming Chen (2022) *Dalton Trans.* ,**51,**14852

Formula: $(C_{96}\ H_{72}\ La_4\ N_8\ O_{52})n,0.75(C_3\ H_7\ N_1\ O_1),4.5(H_2\ O_1)$

Compound Name: catena-[tris(μ-5,5'-(1,3,6,8-tetraoxo-1,3,6,8-tetrahydrobenzo[lmn][3,8]phenanthroline-2,7-diyl)bis(benzene-1,3-dicarboxylato))-bis(N,N-dimethylformamide)-tetradeca-aqua-tetra-lanthanum(iii) N,N-dimethylformamide solvate hydrate]

Space Group:	P-1	***Cell:***	***a***	10.314(0)	***b***	13.198(0)	***c***	19.865(0)
Space Group No.:	2	***(Å,°)***	α	89.55(0)	β	83.43(0)	γ	74.61(0)

R-Factor (%): 3.85 ***Temperature(K):*** 298 ***Density(g/cm^3):*** 1.835

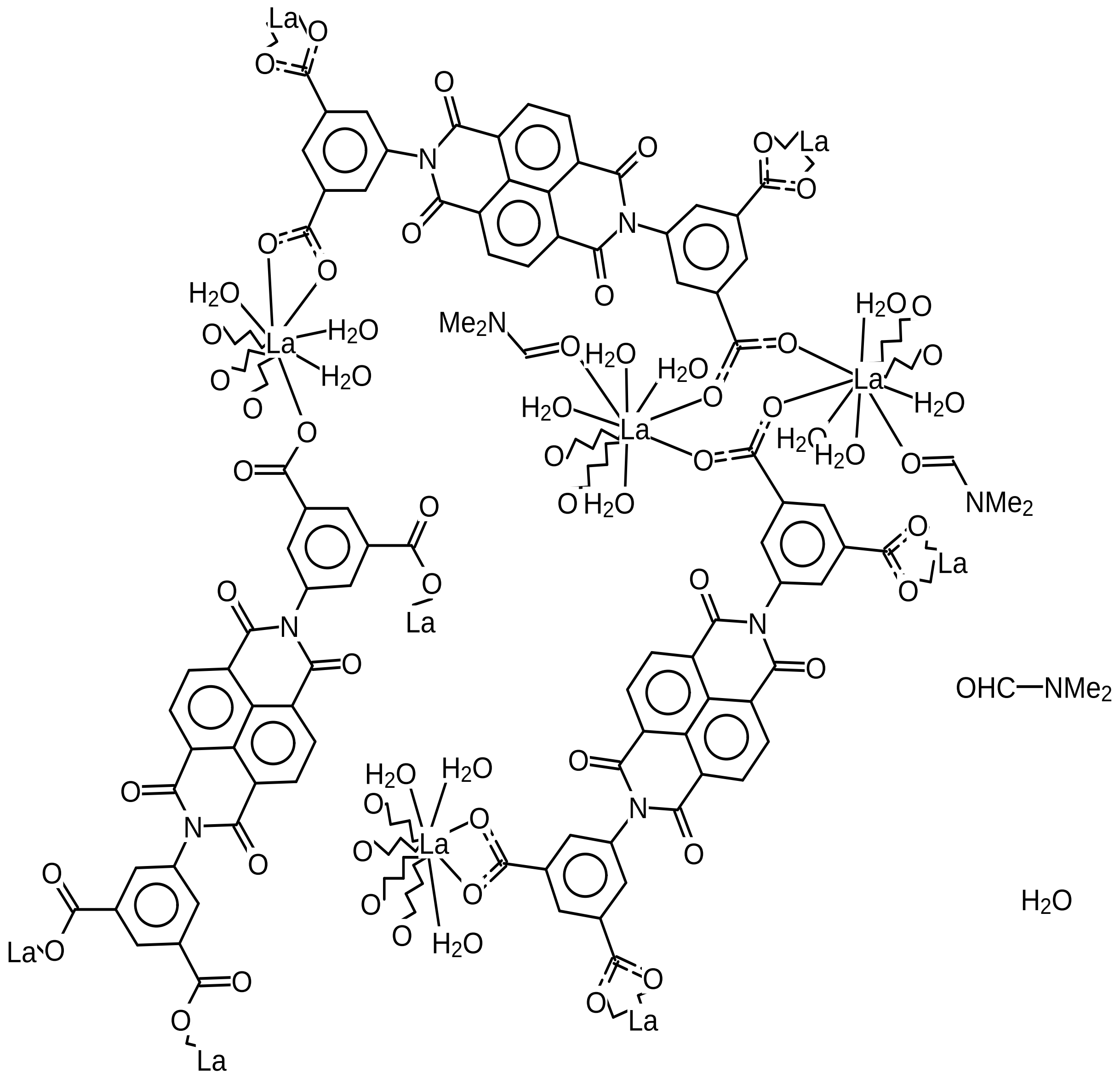

Search: search2 (Mon Jun 30 12:33:47 2025): Hit 14

FEMPIA

Reference:	Xiao-Feng Zhong, Guo-Jun Luo, Wen-Bin Li, Xiong-Hai Chen, Ying Wu, Yi-Hui Chen, Jia-Wen Ye, Jie Bai, Zong-Wen Mo, Xiao-Ming Chen (2022) *Dalton Trans.* ,**51,**14852
Formula:	$(C_{33}\ H_{20}\ La_1\ N_3\ O_{14})n,6.5(C_3\ H_7\ N_1\ O_1),3(H_2\ O_1)$
Compound Name:	catena-[(μ-5-[7-(3-carboxy-5-carboxylatophenyl)-1,3,6,8-tetraoxo-3,6,7,8-tetrahydrobenzo[lmn][3,8]phenanthrolin-2(1H)-yl]benzene-1,3-dicarboxylato)-(N,N-dimethylformamide)-aqua-lanthanum(iii) N,N-dimethylformamide solvate trihydrate]

Space Group:	C2/c	***Cell:***	***a*** 35.864(0)	***b*** 22.974(1)	***c*** 17.016(0)
Space Group No.:	15	***(Å,°)***	α 90.00	β 92.97(0)	γ 90.00

R-Factor (%):	7.71	***Temperature(K):***	298	***Density(g/cm3):***	1.281

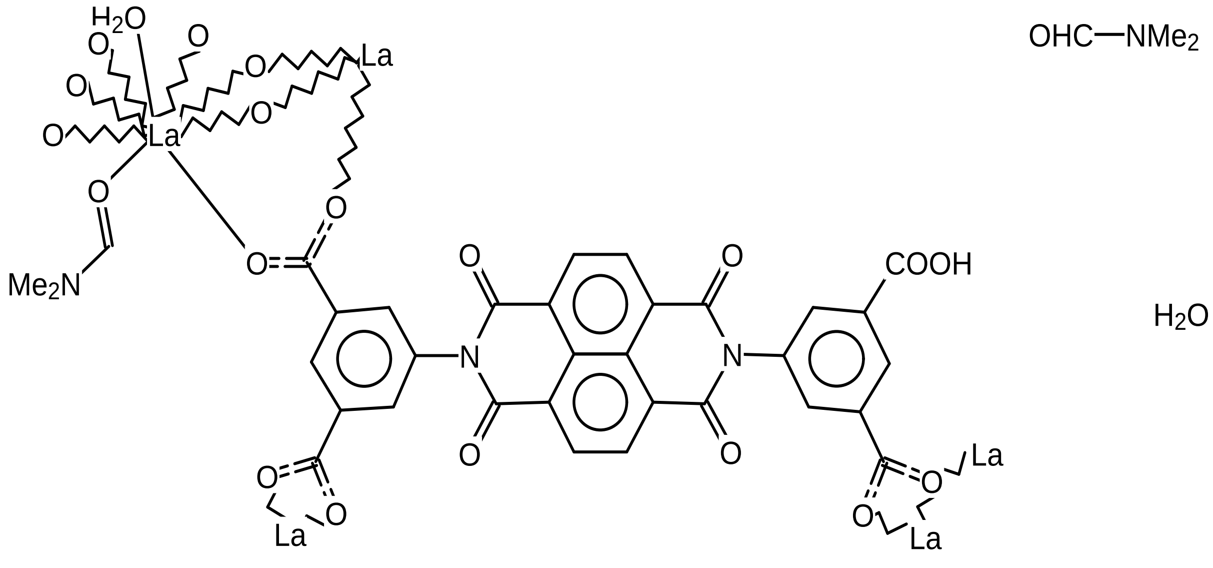

Search: search2 (Mon Jun 30 12:33:47 2025): Hit 15

FOMWOV

Reference:	Di-Chang Zhong, Lie-Qiang Liao, Ji-Hua Deng, Qing Chen, Ping Lian, Xu-Zhong Luo (2014) *Chem.Commun.* ,**50,** 15807
Formula:	$(C_{90} H_{32} N_6 O_{36} Zn_4{}^{2-})n,16(C_3 H_7 N_1 O_1),2(C_2 H_8 N_1{}^{1+}),10(H_2 O_1)$
Compound Name:	catena-(bis(dimethylammonium) bis(μ-hydrogen 5,5'-(1,3,6,8-tetraoxo-1,3,6,8-tetrahydrobenzo[lmn][3,8]phenanthroline-2,7-diyl)diisophthalato)-(μ-5,5'-(1,3,6,8-tetraoxo-1,3,6,8-tetrahydrobenzo[lmn][3,8]phenanthroline-2,7-diyl)diisophthalato)-tetra-zinc N,N-dimethylformamide solvate decahydrate)

Space Group:	C2/c	***Cell:***	***a*** 38.596(8)	***b*** 15.279(3)	***c*** 23.600(5)
Space Group No.:	15	***(Å,°)***	α 90.00	β 101.29(3)	γ 90.00

R-Factor (%): 7.83 ***Temperature(K):*** 293 ***Density(g/cm³):*** 1.692

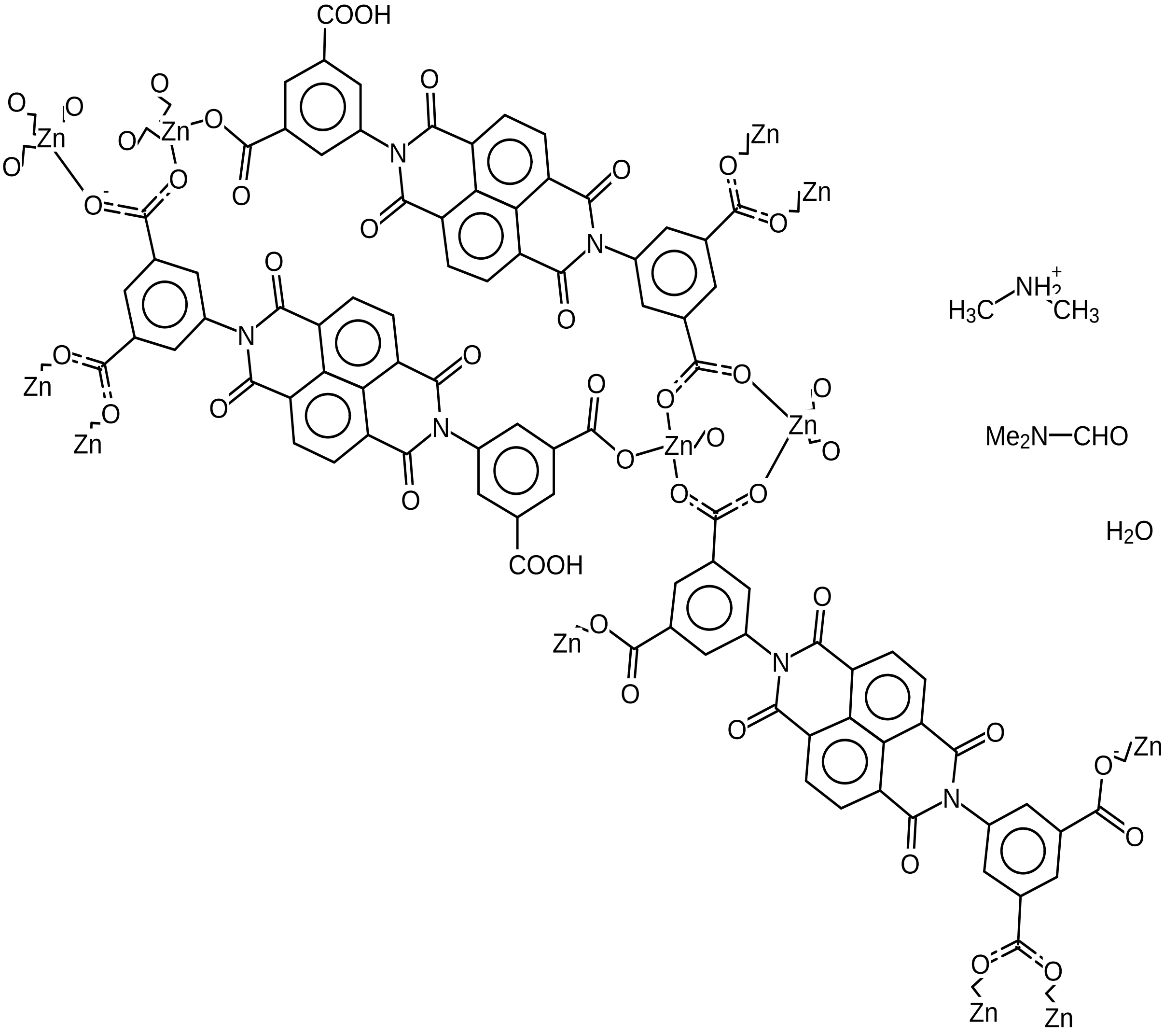

Search: search2 (Mon Jun 30 12:33:47 2025): Hit 16

FOTCOK

Reference:	Wu Xue (2024)<br>*CSD Communication(Private Communication) ,*
Formula:	$(C_{60} H_{20} Bi_2 N_4 O_{24}{}^{2-})n,2(C_2 H_8 N_1{}^{1+})$
Compound Name:	catena-[bis(dimethylammonium) bis(μ-5,5'-(1,3,6,8-tetraoxo-1,3,6,8-tetrahydrobenzo[lmn][3,8]phenanthroline-2,7-diyl)di(benzene-1,3-dicarboxylato))-di-bismuth(iii) unknown solvate]

Space Group:	P21/n	***Cell:***	***a***	9.943(0)	***b***	18.726(0)	***c***	35.820(1)
Space Group No.:	14	***(Å,°)***	α	90.00	β	94.45(0)	γ	90.00

R-Factor (%): 6.99 ***Temperature(K):*** 193 ***Density(g/cm³):*** 0.845

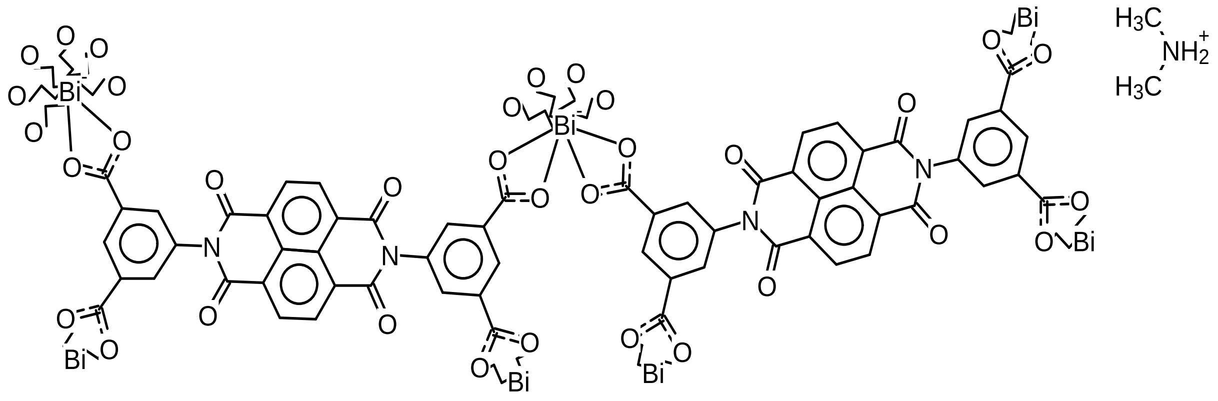

Search: search2 (Mon Jun 30 12:33:47 2025): Hit 17

FURGIM

Reference:	Ruo-Nan Wang, Yu-Chuan Tan, Wei Liu, Zi-Yi Wang, Jun-Die Zhang, Qin-Yu Zhu (2025) *Chem.Commun.* ,**61,**2289
Formula:	$(C_{52} H_{34} N_6 O_{14} S_4 Zn_2)$n,2$(C_3 H_7 N_1 O_1)$,2$(H_2 O_1)$
Compound Name:	catena-[(μ-4-{2-[4-(pyridin-4-yl)-2H-1,3-dithiol-2-ylidene]-2H-1,3-dithiol-4-yl}pyridine)-(μ-5,5'-(1,3,6,8-tetraoxo-1,3,6,8-tetrahydrobenzo[lmn][3,8]phenanthroline-2,7-diyl)bis(benzene-1,3-dicarboxylato))-bis(N,N-dimethylformamide)-di-zinc(ii) N,N-dimethylformamide unknown solvate dihydrate]

Space Group:	Pccn	***Cell:***	***a*** 32.963(1)	***b*** 13.319(0)	***c*** 15.410(0)		
Space Group No.:	56	***(Å,°)***	α 90.00	β 90.00	γ 90.00		

R-Factor (%): 12.77 ***Temperature(K):*** 100 ***Density(g/cm³):*** 1.383

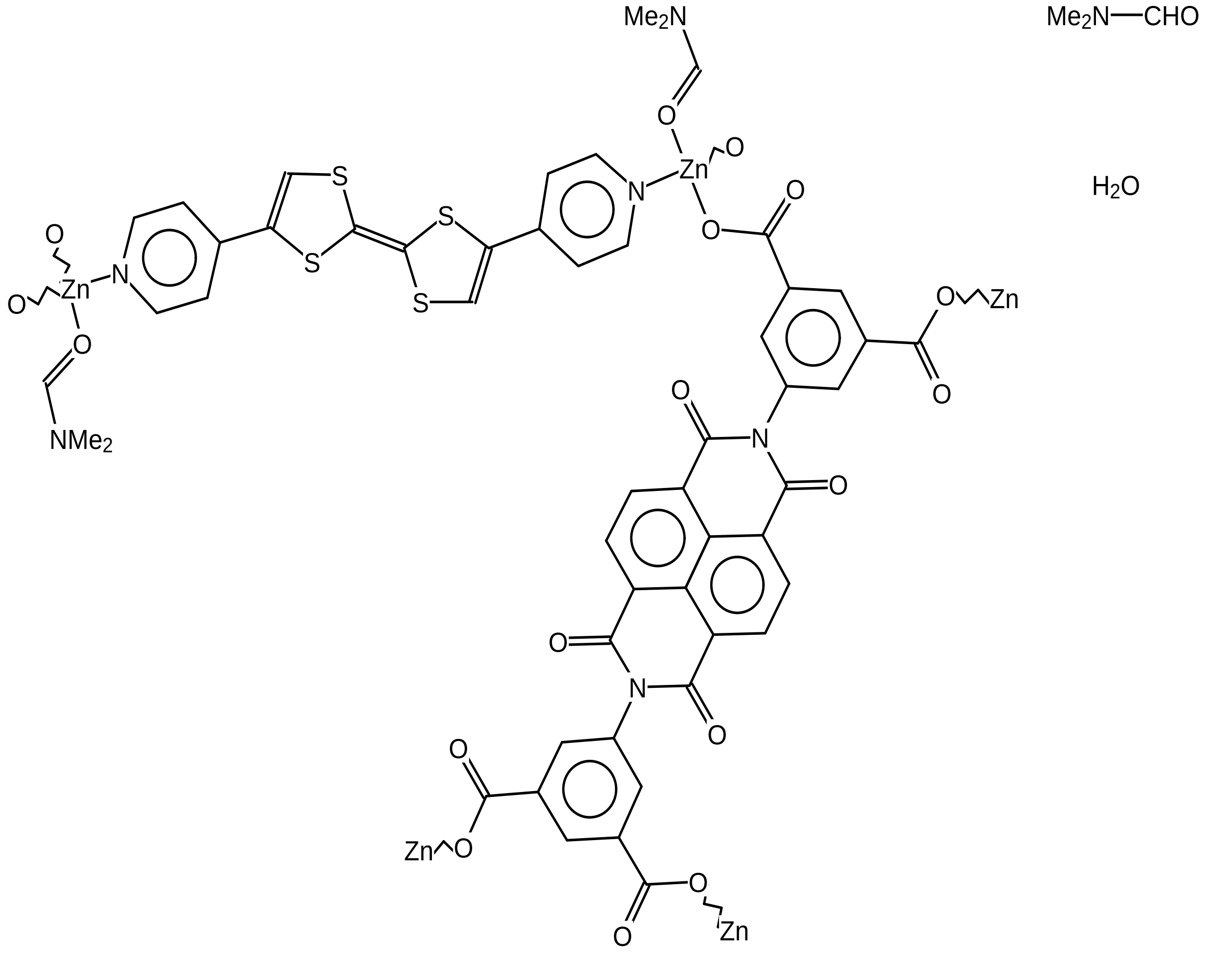

Search: search2 (Mon Jun 30 12:33:47 2025): Hit 18

GARPUO

Reference:	Jian-Jun Liu, Jia-Jia Fu, Teng Liu, Chi-Xian He, Fei-Xiang Cheng (2021) *J.Mol.Struct.* ,132011
Formula:	$(C_{54} H_{66} Ce_2 N_{10} O_{20}{}^{2+})n,n(O_{19} W_6{}^{2-})$
Compound Name:	catena-((μ-5,5'-(1,3,6,8-tetraoxo-1,3,6,8-tetrahydrobenzo[lmn][3,8]phenanthroline-2,7-diyl)bis(benzene-1,3-dicarboxylato))-octakis(dimethylformamide)-di-cerium tridecakis(μ-oxo)-hexaoxo-hexa-tungsten)

Space Group:	P-1	***Cell:***	***a***	10.912(0)	***b***	12.564(0)	***c***	14.340(0)
Space Group No.:	2	***(Å,°)***	α	82.70(0)	β	81.48(0)	γ	75.53(0)

R-Factor (%):	3.87	***Temperature(K):***	153	***Density(g/cm³):***	2.536

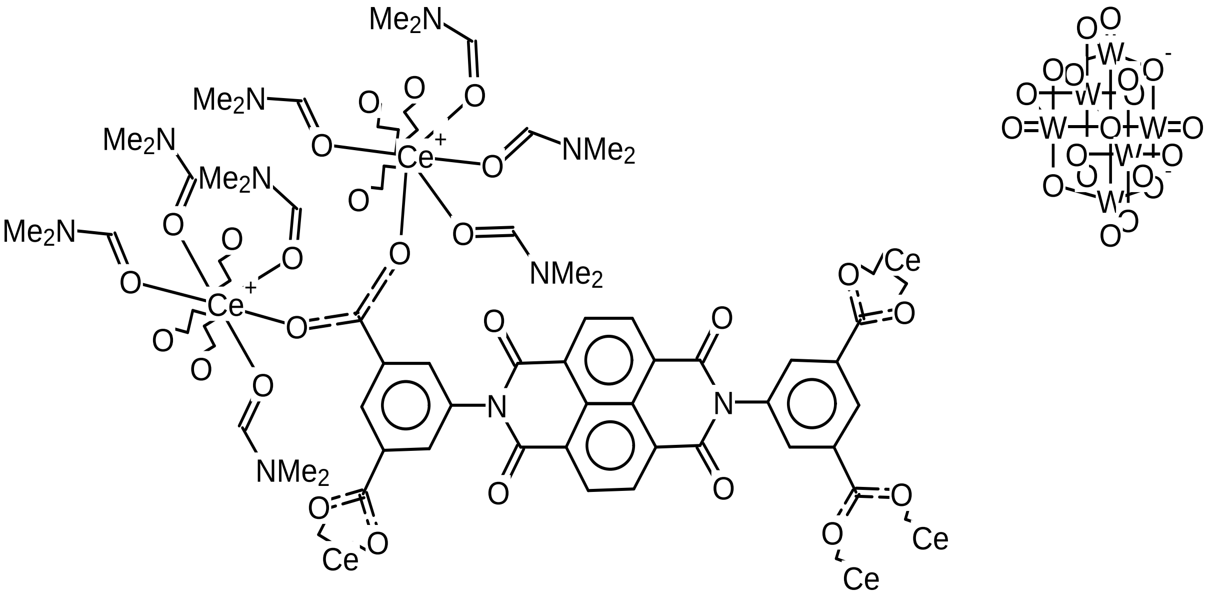

Search: search2 (Mon Jun 30 12:33:47 2025): Hit 19

GIHTIE

Reference: Zhonghe Wang, Le Zeng, Cheng He, Chunying Duan
(2022) *ACS Applied Materials and Interfaces* ,**14,**7980

Formula: $(C_{42} H_{38} N_6 O_{16} Sr_2)n,C_{14} H_8 O_2$

Compound Name: catena-[(μ-5,5'-(1,3,6,8-tetraoxo-1,3,6,8-tetrahydrobenzo[lmn][3,8]phenanthroline-2,7-diyl)bis(benzene-1,3-dicarboxylato))-tetrakis(N,N-dimethylformamide)-di-strontium(ii) anthracene-9,10-dione]

Space Group:	I41/a	***Cell:***	***a***	28.528(1)	***b***	28.528(1)	***c***	13.680(0)
Space Group No.:	88	***(Å,°)***	α	90.00	β	90.00	γ	90.00

R-Factor (%): 4.79 ***Temperature(K):*** 298 ***Density(g/cm³):*** 1.511

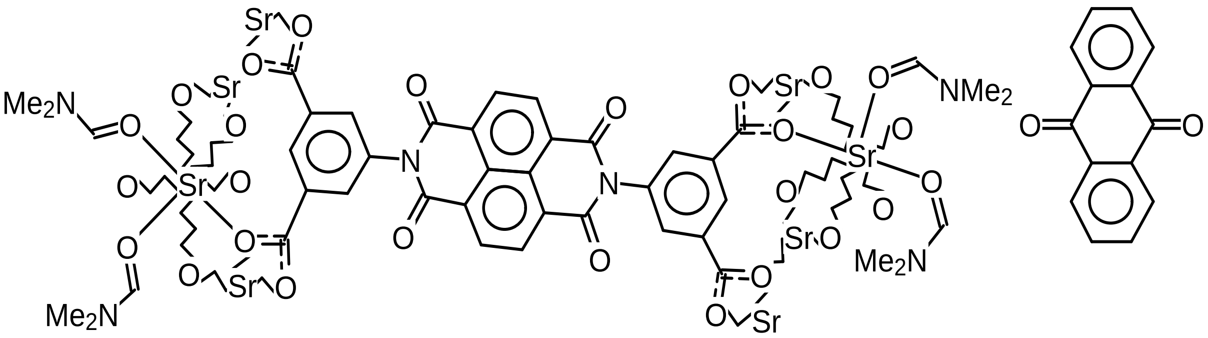

Search: search2 (Mon Jun 30 12:33:47 2025): Hit 20

GOZLEQ

Reference:	Yuling Mao, Boyu Shan, Jia Yu, Xinxin Liu, Jingying Chu, Xinyue Ma, Miao Li, Yimeng Zheng, Baili Zhu, Minghui Zuo, Cui Shuxin (2024) *J.Solid State Chem.* ,**338,**							
Formula:	$(C_{34} H_{20} N_4 O_{14} Zn_2)n$							
Space Group:	Imma	***Cell:***	***a***	15.120(1)	***b***	36.799(4)	***c***	10.512(1)
Space Group No.:	74	***(Å,°)***	α	90.00	β	90.00	γ	90.00
R-Factor (%):	7.84	***Temperature(K):***	296	***Density(g/cm³):***	0.953			

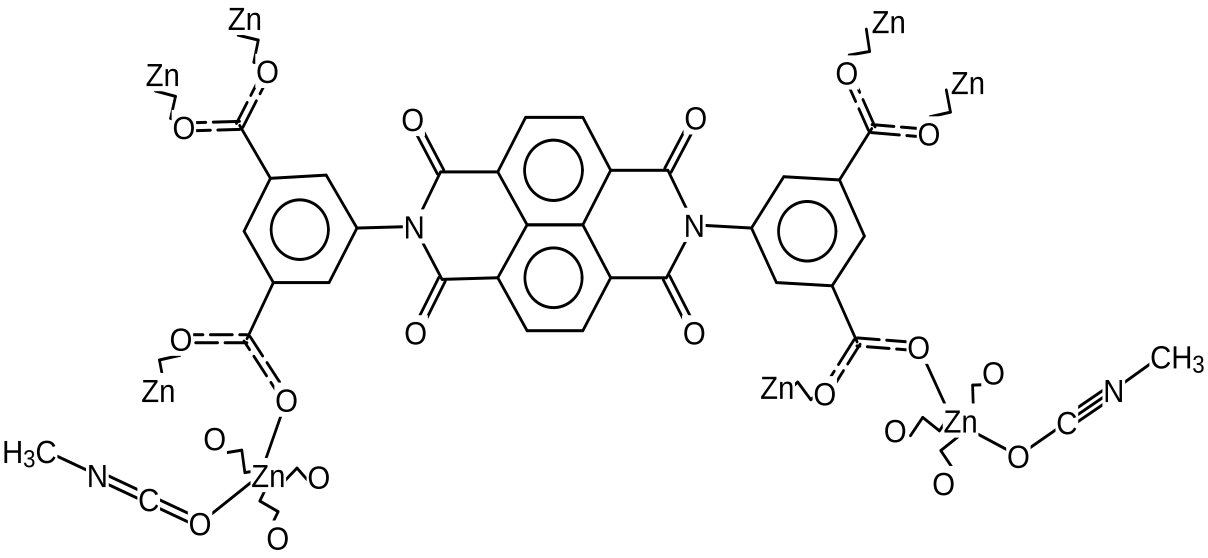

Search: search2 (Mon Jun 30 12:33:47 2025): Hit 21

GULQIR

Reference:	Zhonghe Wang, Yang Tang, Songtao Liu, Liang Zhao, Huaqing Li, Cheng He, Chunying Duan (2024) *Nat.Commun.* ,**15,**8813
Formula:	$(C_{42}\ H_{38}\ Fe_2\ N_6\ O_{16})n$
Compound Name:	catena-[(μ-5,5'-(1,3,6,8-tetraoxo-1,3,6,8-tetrahydrobenzo[lmn][3,8]phenanthroline-2,7-diyl)di(benzene-1,3-dicarboxylato))-tetrakis(N,N-dimethylformamide)-di-iron unknown solvate]

Space Group:	P21/c	***Cell:***	***a*** 20.133(5)	***b*** 16.304(4)	***c*** 10.135(2)
Space Group No.:	14	***(Å,°)***	α 90.00	β 103.08(0)	γ 90.00
R-Factor (%):	7.00	***Temperature(K):***	250	***Density(g/cm³):***	1.019

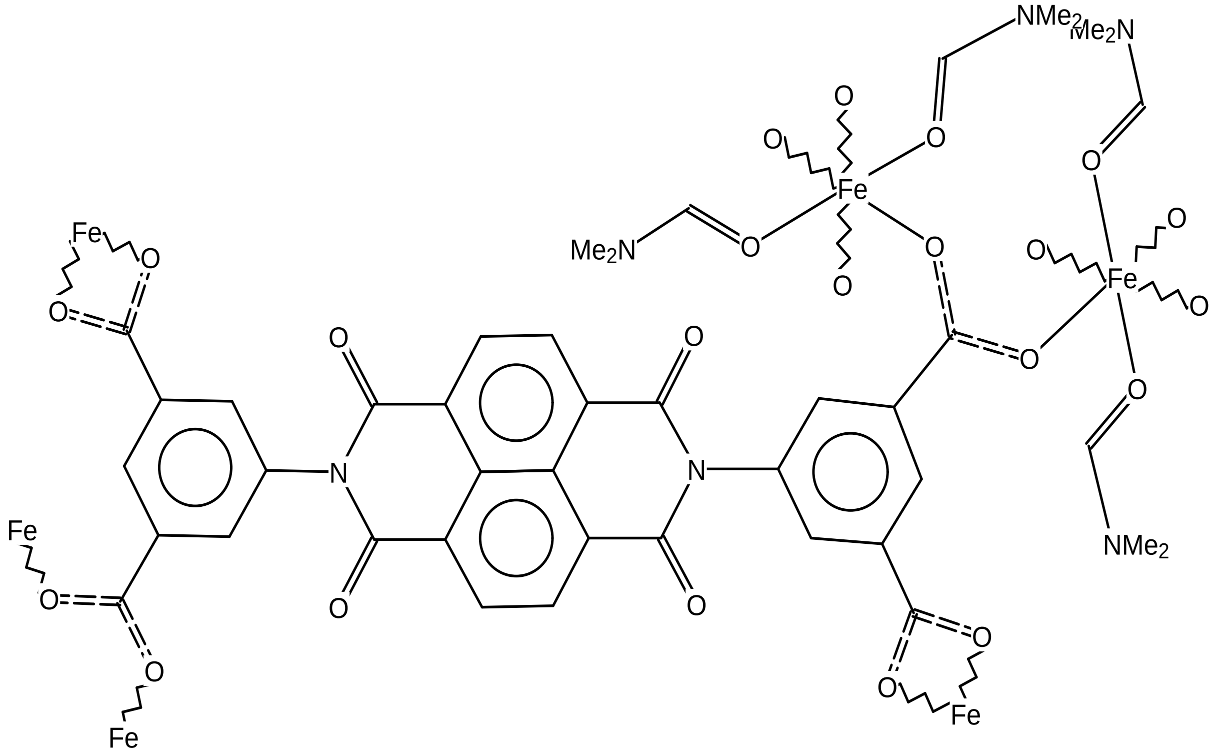

Search: search2 (Mon Jun 30 12:33:47 2025): Hit 22

HAMLIT

Reference: Shixing Zhang, Hongming He, Fuxing Sun, Nian Zhao, Jianshi Du, Qinhe Pan, Guangshan Zhu (2016) *Inorg.Chem.Commun.* , **79,**55

Formula: $2(C_2H_8N_1{}^{1+}),n(C_{120}H_{62}N_{36}O_{40}Zn_8{}^{2-}),8(C_3H_7N_1O_1)$

Compound Name: catena-[bis(Dimethylammonium) bis(μ-2,7-bis(3,5-dicarboxylatophenyl)-1,3,6,8-tetraoxo-1,3,6,8-tetrahydrobenzo[lmn][3,8]phenanthroline)-hexakis(μ-adenin-9-yl)-tetra-aqua-octa-zinc N,N-dimethylformamide solvate]

Synonym: JUC-188

Space Group:	P-1	***Cell:***	***a*** 12.147(0)	***b*** 18.109(0)	***c*** 24.134(0)
Space Group No.:	2	***(Å,°)***	α 107.54(0)	β 98.67(0)	γ 91.33(0)

R-Factor (%): 5.00 ***Temperature(K):*** 100 ***Density(g/cm³):*** 1.280

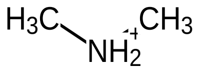

$$n\text{-}Zn_8\text{--}$$
$$\text{--}[\mu_4\text{-}C_{30}H_{10}N_2O_{12}\text{-}\kappa^6O,O',O'',O''',O'''',O''''']_2[\mu_4\text{-}C_{30}H_{10}N_2O_{12}\text{-}\kappa^4O,O',O'',O''']\text{--}$$
$$\text{--}[\mu_3\text{-}C_5H_4N_5\text{-}\kappa^3N,N',N'']_6[H_2O]_4{}^{2-}$$

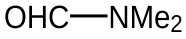

Search: search2 (Mon Jun 30 12:33:47 2025): Hit 23

HEWHAW

Reference: Zi-Hao Zhu, Ze-Long Liang, Sheng-Li Hou, Yao Xie, Yue Ma, Yan Zhang, Bin Zhao (2021) *J. Energy Chem.* ,**63,**328

Formula: $(C_{60} H_{20} Bi_2 N_4 O_{24}{}^{2-})n,2(C_2 H_8 N_1{}^{1+}),8(C_3 H_7 N_1 O_1),4(H_2 O_1)$

Compound Name: catena-[bis(dimethylammonium) bis(μ-5,5'-(1,3,6,8-tetraoxo-1,3,6,8-tetrahydrobenzo[lmn][3,8]phenanthroline-2,7-diyl)di(benzene-1,3-dicarboxylato))-di-bismuth(iii) N,N-dimethylformamide solvate tetrahydrate]

Space Group:	P21/n	***Cell:***	***a*** 9.956(0)	***b*** 18.721(0)	***c*** 35.579(1)
Space Group No.:	14	***(Å,°)***	α 90.00	β 94.03(0)	γ 90.00

R-Factor (%): 8.59 ***Temperature(K):*** 121 ***Density(g/cm^3):*** 1.179

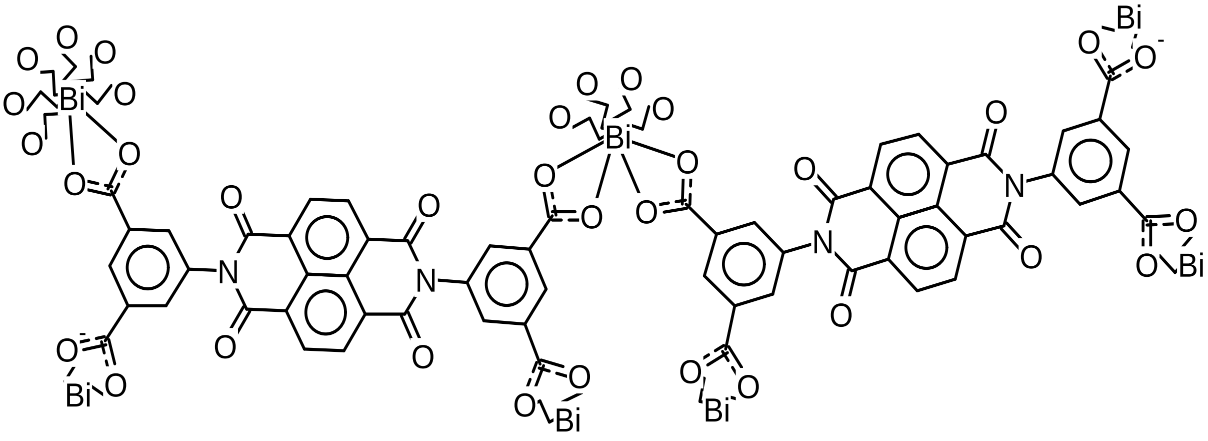

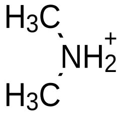

OHC−NMe2

H_2O

Search: search2 (Mon Jun 30 12:33:47 2025): Hit 24

HUQJOV

Reference: Li-Jun Zhai, Chen-Xu Jiao, Jin-Fang Liang, Jie Zhang, Xiao-Yan Niu, Tuo-Ping Hu, Yu-Lan Niu (2020) *Jiegou Huaxue(Chin.)(Chin.J.Struct.Chem.)* ,**39,**772

Formula: $(C_{48} H_{26} N_6 O_{12} Zn_1)n$

Compound Name: catena-[(μ-1,1'-([1,1'-biphenyl]-4,4'-diyl)di(1H-imidazole))-(μ-dihydrogen 5,5'-(1,3,6,8-tetraoxo-1,3,6,8-tetrahydrobenzo[lmn][3,8]phenanthroline-2,7-diyl)di(benzene-1,3-dicarboxylato))-zinc unknown solvate]

Space Group:	P21/c	***Cell:***	***a*** 8.456(0)	***b*** 15.188(0)	***c*** 32.996(1)
Space Group No.:	14	***(Å,°)***	α 90.00	β 90.85(0)	γ 90.00

R-Factor (%): 5.20 ***Temperature(K):*** 100 ***Density(g/cm³):*** 1.480

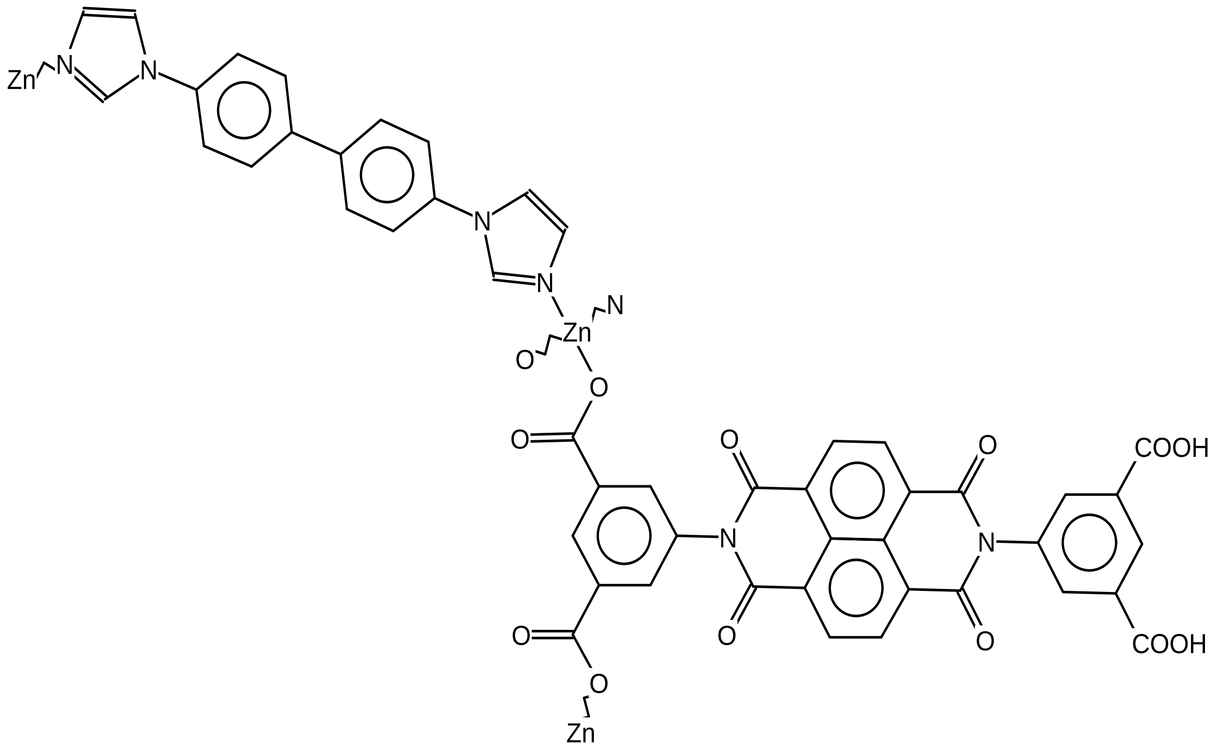

Search: search2 (Mon Jun 30 12:33:47 2025): Hit 25

HUQJUB

Reference:	Li-Jun Zhai, Chen-Xu Jiao, Jin-Fang Liang, Jie Zhang, Xiao-Yan Niu, Tuo-Ping Hu, Yu-Lan Niu (2020) *Jiegou Huaxue(Chin.)(Chin.J.Struct.Chem.)* ,**39,**772
Formula:	$(C_{44} H_{28} Cd_2 N_6 O_{14})n,2(C_2 H_6 O_1)$
Compound Name:	catena-[(μ-5,5'-(1,3,6,8-tetraoxo-1,3,6,8-tetrahydrobenzo[lmn][3,8]phenanthroline-2,7-diyl)di(benzene-1,3-dicarboxylato))-(μ-1,1'-[1,4-phenylenebis(methylene)]di(1H-imidazole))-diaqua-di-cadmium ethanol solvate]

Space Group:	P-1	***Cell:***	***a*** 8.244(0)		***b*** 10.327(0)		***c*** 13.478(0)
Space Group No.:	2	***(Å,°)***	α 92.10		β 91.11		γ 109.46(0)

R-Factor (%):	4.45	***Temperature(K):***	100	***Density(g/cm³):***	1.816

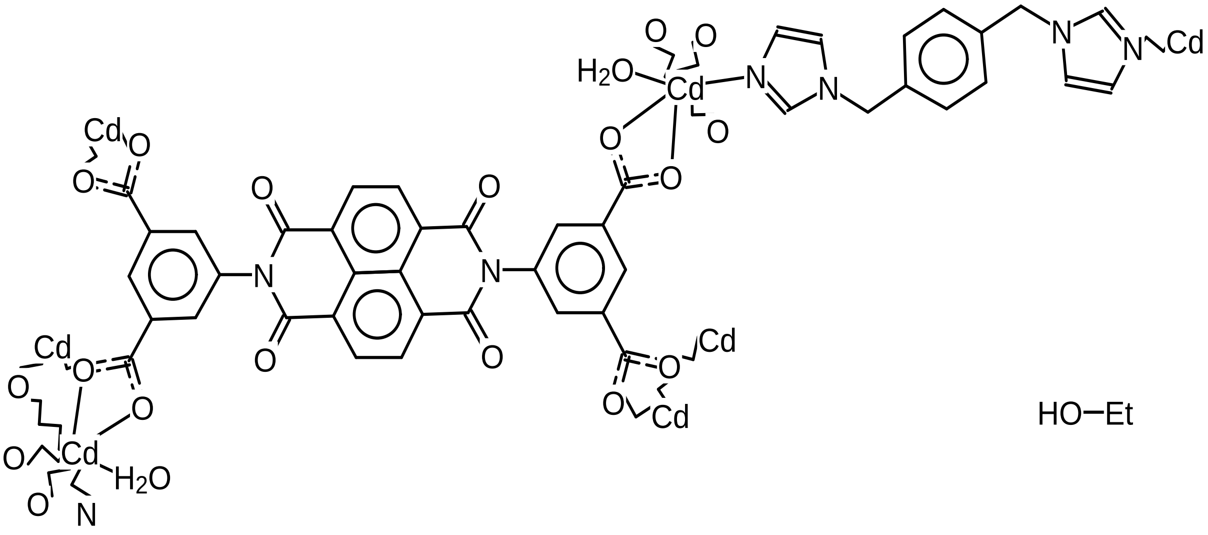

Search: search2 (Mon Jun 30 12:33:47 2025): Hit 26

IGIFOW

Reference: Yuanyuan Zhang, Xuan Zhang, Zhijie Chen, K.Otake, G.Peterson, Yongwei Chen, Xingjie Wang, L.Redfern, S.Goswami, Peng Li, T.Islamoglu, Bo Wang, O.K.Farha (2020) *ChemSusChem* ,**13,** 1710

Formula: $(C_{34}\ H_{30}\ N_2\ O_{36}\ Zr_6)n$

Compound Name: catena-(tetrakis(μ-formato)-tetrakis(μ-hydroxo)-tetrakis(μ-oxo)-(μ-5,5'-(1,3,6,8-tetraoxo-1,3,6,8-tetrahydrobenzo[lmn][3,8]phenanthroline-2,7-diyl)bis(benzene-1,3-dicarboxylato))-tetra-aqua-tetrahydroxy-hexa-zirconium unknown solvate)

Synonym: NU-1401-AS-DMF

Space Group:	Ibam	***Cell:***	***a***	21.456(1)	***b***	25.233(2)	***c***	33.455(3)
Space Group No.:	72	***(Å,°)***	α	90.00	β	90.00	γ	90.00

R-Factor (%): 6.55 ***Temperature(K):*** 200 ***Density(g/cm³):*** 1.166

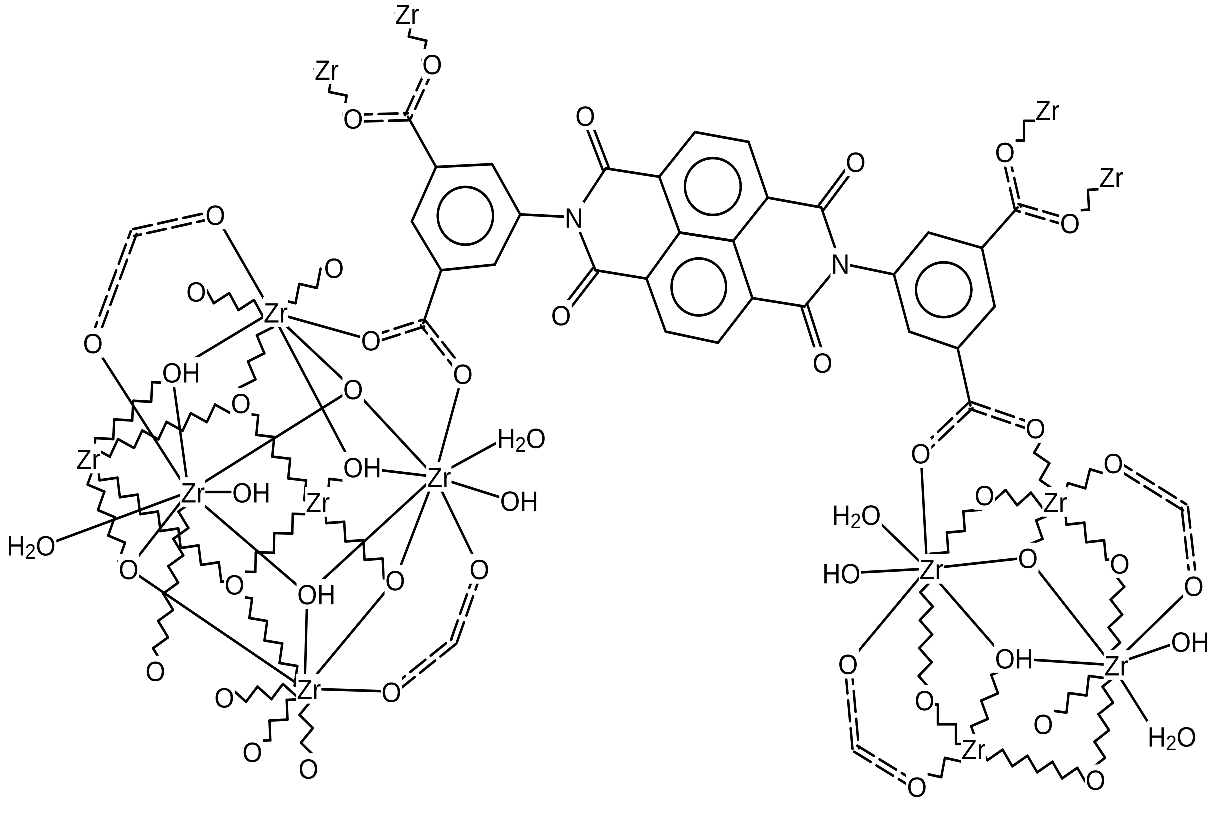

Search: search2 (Mon Jun 30 12:33:47 2025): Hit 27

IGIGAJ

Reference:	Yuanyuan Zhang, Xuan Zhang, Zhijie Chen, K.Otake, G.Peterson, Yongwei Chen, Xingjie Wang, L.Redfern, S.Goswami, Peng Li, T.Islamoglu, Bo Wang, O.K.Farha (2020) *ChemSusChem* ,**13,** 1710
Formula:	$(C_{65}\ H_{66}\ N_4\ O_{72}\ Zr_{12})n$
Compound Name:	catena-[bis[μ-5,5'-(1,3,6,8-tetraoxo-1,3,6,8-tetrahydrobenzo[lmn][3,8]phenanthroline-2,7-diyl)bis(benzene-1,3-dicarboxylato)]-octakis(μ-hydroxido)-octakis(μ-oxido)-pentakis(μ-formato)-undecakis(hydroxido)-undeca-aqua-dodeca-zirconium(iv) unknown solvate]
Synonym:	NU-1401-water

Space Group:	Ibam	***Cell:***	***a***	17.937(3)	***b***	25.232(3)	***c***	35.902(5)
Space Group No.:	72	***(Å,°)***	α	90.00	β	90.00	γ	90.00

R-Factor (%):	5.44	***Temperature(K):***	200	***Density(g/cm³):***	1.288

$$n\text{-}Zr_{12}[\mu_8\text{-}C_{30}H_{10}N_2O_{12}]_2[\mu_3\text{-}HO]_8[\mu_3\text{-}O]_8\text{--}$$
$$\text{--}[\mu_2\text{-}CHO_2\text{-}\kappa^2O,O']_5\text{--}$$
$$\text{--}[H_2O]_{11}[HO]_{11}$$

Search: search2 (Mon Jun 30 12:33:47 2025): Hit 28

IGIGEN

Reference:	Yuanyuan Zhang, Xuan Zhang, Zhijie Chen, K.Otake, G.Peterson, Yongwei Chen, Xingjie Wang, L.Redfern, S.Goswami, Peng Li, T.Islamoglu, Bo Wang, O.K.Farha (2020) *ChemSusChem* ,**13,** 1710
Formula:	$(C_{63}\ H_{62}\ N_4\ O_{68}\ Zr_{12})n$
Compound Name:	catena-[bisis[μ-5,5'-(1,3,6,8-tetraoxo-1,3,6,8-tetrahydrobenzo[lmn][3,8]phenanthroline-2,7-diyl)bis(benzene-1,3-dicarboxylato)]-octakis(μ-hydroxido)-octakis(μ-oxido)-tris(μ-formato)-tridecakis(hydroxido)-nona-aqua-dodeca-zirconium(iv) unknown solvate]
Synonym:	NU-1401-EtOH

Space Group:	Ibam	***Cell:***	***a***	16.434(1)	***b***	25.391(3)	***c***	36.506(2)
Space Group No.:	72	***(Å,°)***	α	90.00	β	90.00	γ	90.00

R-Factor (%): 6.42 ***Temperature(K):*** 200 ***Density(g/cm³):*** 1.333

$$n\text{-}Zr_{12}[\mu_8\text{-}C_{30}H_{10}N_2O_{12}]_2[\mu_3\text{-}HO]_8[\mu_3\text{-}O]_8\text{--}$$
$$\text{--}[\mu_2\text{-}CHO_2\text{-}\kappa^2O,O']_3[HO]_{13}[H_2O]_9$$

Search: search2 (Mon Jun 30 12:33:47 2025): Hit 29

IGIGIR

Reference: Yuanyuan Zhang, Xuan Zhang, Zhijie Chen, K.Otake, G.Peterson, Yongwei Chen, Xingjie Wang, L.Redfern, S.Goswami, Peng Li, T.Islamoglu, Bo Wang, O.K.Farha (2020) *ChemSusChem* ,**13,** 1710

Formula: $(C_{66} H_{56} N_4 O_{70} Zr_{12})n,H_2 O_1$

Compound Name: catena-[bis[μ-5,5'-(1,3,6,8-tetraoxo-1,3,6,8-tetrahydrobenzo[lmn][3,8]phenanthroline-2,7-diyl)bis(benzene-1,3-dicarboxylato)]-bis(μ-carbonato)-octakis(μ-hydroxido)-decakis(μ-oxido)-tetrakis(μ-formato)-tetrakis(hydroxido)-deca-aqua-dodeca-zirconium(iv) monohydrate unknown solvate]

Synonym: NU-1401-CO2

Space Group:	Ibam	***Cell:***	***a***	16.496(2)	***b***	25.452(3)	***c***	36.185(5)
Space Group No.:	72	***(Å,°)***	α	90.00	β	90.00	γ	90.00

R-Factor (%): 6.16 ***Temperature(K):*** 200 ***Density(g/cm³):*** 1.372

$n\text{-}Zr_{12}[\mu_8\text{-}C_{30}H_{10}N_2O_{12}]_2[\mu_3\text{-}HO]_8[\mu_3\text{-}O]_{10}$--
--$[\mu_2\text{-}CHO_2\text{-}\kappa^2O,O']_4$--
--$[\mu_2\text{-}CO_3\text{-}\kappa^3O,O',O'']_2[HO]_4[H_2O]_{10}$

H_2O

Search: search2 (Mon Jun 30 12:33:47 2025): Hit 30

IHUBEW

Reference: Kexin Liu, Lingsong Wang, Shuyu Li, Huapeng Liu, Dong Zhang, Mengjia Jiang, Wei Chen, Fei Jiao, Xiaotao Zhang, Wenping Hu (2023) *Adv.Funct.Mater.* ,**33,**2306871

Formula: $(C_{42} H_{38} N_6 O_{16} Sr_2)n,C_{16} H_{14}$

Compound Name: catena-[(μ-5,5'-(1,3,6,8-tetraoxo-1,3,6,8-tetrahydrobenzo[lmn][3,8]phenanthroline-2,7-diyl)bis(benzene-1,3-dicarboxylato))-tetrakis(N,N-dimethylformamide)-di-strontium(ii) 9,10-dimethylanthracene clathrate]

Space Group:	I41/a	***Cell:***	***a***	28.289(0)	***b***	28.289(0)	***c***	13.890(0)
Space Group No.:	88	***(Å,°)***	α	90.00	β	90.00	γ	90.00

R-Factor (%): 2.63 ***Temperature(K):*** 160 ***Density(g/cm³):*** 1.511

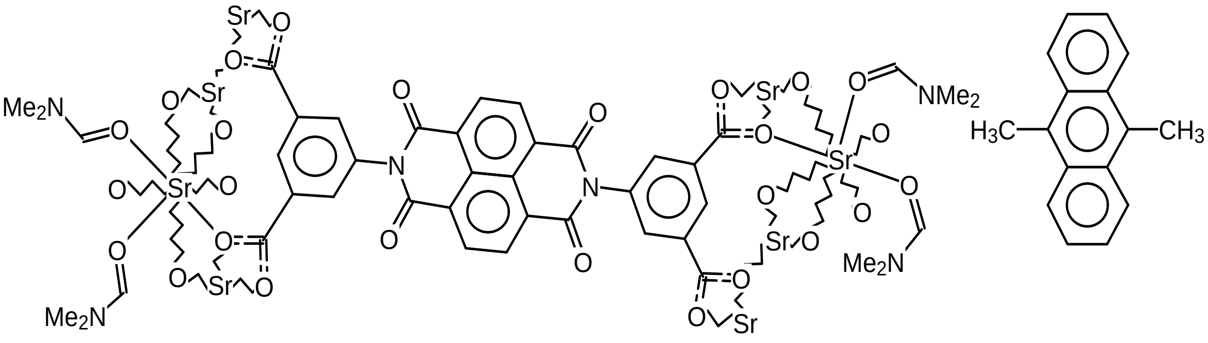

Search: search2 (Mon Jun 30 12:33:47 2025): Hit 31

ILALUF

Reference:	Fangna Dai, Xiaokang Wang, Yutong Wang, Zhanning Liu, Daofeng Sun (2020) *Angew.Chem.,Int.Ed.* ,**59,**22372
Formula:	$(C_{30}\ H_{10}\ Ba_2\ N_2\ O_{12})n$
Compound Name:	catena-[(5,5'-(1,3,6,8-tetraoxo-1,3,6,8-tetrahydrobenzo[lmn][3, 8]phenanthroline-2,7-diyl)bis(benzene-1,3-dicarboxylato)-di-barium(ii) unknown solvate)]
Synonym:	UPC-600

Space Group:	P-1	***Cell:***	***a***	8.270(0)	***b***	8.485(0)	***c***	13.509(1)
Space Group No.:	2	***(Å,°)***	α	81.40(0)	β	74.52(0)	γ	75.95(0)
R-Factor (%):	9.46	***Temperature(K):***	150		***Density(g/cm³):***	1.628		

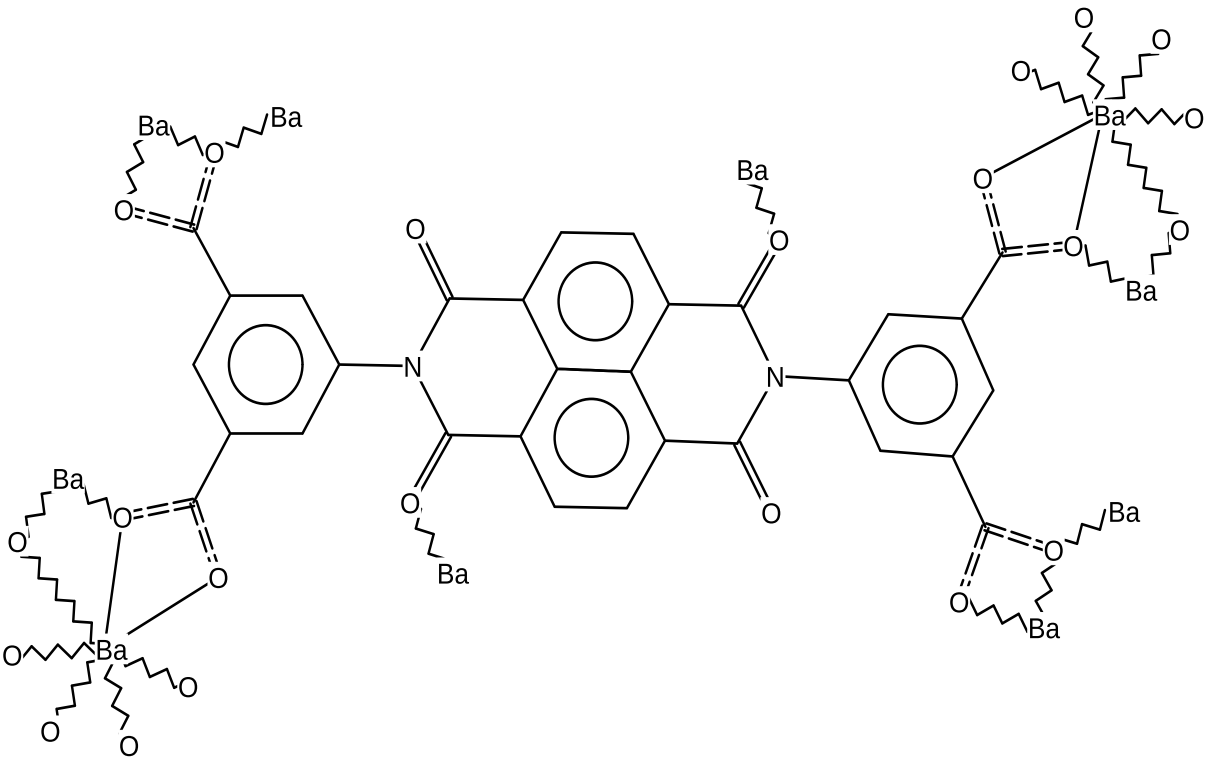

Search: search2 (Mon Jun 30 12:33:47 2025): Hit 32

IYACOD

Reference: G.Radha, T.Leelasree, D.Muthukumar, R.S.Pillai, H.Aggarwal (2021) *New J.Chem.* ,**45,**12931

Formula: $(C_{34} H_{30} N_2 O_{36} Zr_6)n$

Compound Name: catena-(tetrakis(μ-formato)-tetrakis(μ-hydroxo)-tetrakis(μ-oxo)-(μ-5,5'-(1,3,6,8-tetraoxo-1,3,6,8-tetrahydrobenzo[lmn][3,8]phenanthroline-2,7-diyl)bis(benzene-1,3-dicarboxylato))-tetra-aqua-tetrahydroxy-hexa-zirconium unknown solvate)

Space Group:	Ibam	***Cell:***	***a***	21.490(1)	***b***	25.293(0)	***c***	33.120(1)
Space Group No.:	72	***(Å,°)***	α	90.00	β	90.00	γ	90.00

R-Factor (%): 7.16 ***Temperature(K):*** 100 ***Density(g/cm^3):*** 1.173

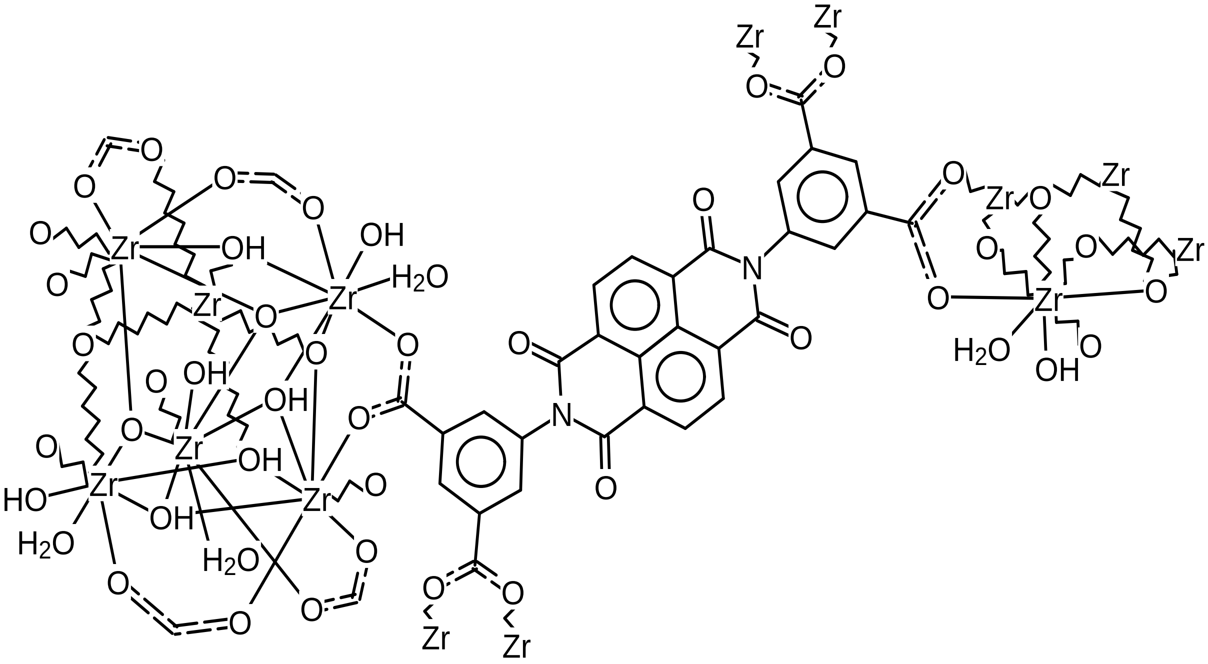

Search: search2 (Mon Jun 30 12:33:47 2025): Hit 33

KEVKAA

Reference:	Hui-Ling Xu, Xiao-Shan Zeng, Jie Li, Yu-Ci Xu, Hai-Jiang Qiu, Dong-Rong Xiao (2018) *CrystEngComm* ,**20,**2430
Formula:	$(C_{60} H_{26} Cd_1 N_4 O_{24})n,4(C_3 H_7 N_1 O_1)$
Compound Name:	catena-[(μ-5,5'-(1,3,6,8-tetraoxo-1,3,6,8-tetrahydrobenzo[lmn][3,8]phenanthroline-2,7-diyl)di(benzene-1,3-dicarboxylic acid))-(μ-5,5'-(1,3,6,8-tetraoxo-1,3,6,8-tetrahydrobenzo[lmn][3,8]phenanthroline-2,7-diyl)di(hydrogen benzene-1,3-dicarboxylato))-cadmium(ii) N,N-dimethylformamide solvate]

Space Group:	I2/c	***Cell:***	***a*** 18.553(0)	***b*** 22.854(0)	***c*** 20.096(0)
Space Group No.:	15	***(Å,°)***	α 90.00	β 107.35(0)	γ 90.00
R-Factor (%):	5.50	***Temperature(K):***	293	***Density(g/cm³):***	1.300

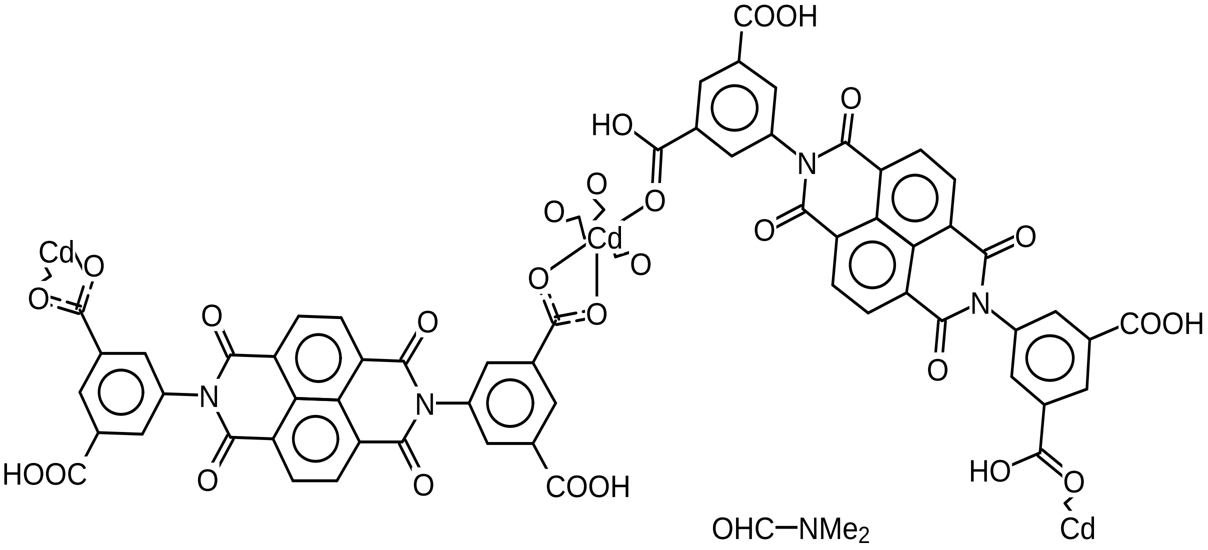

Search: search2 (Mon Jun 30 12:33:47 2025): Hit 34

KEVKEE

Reference: Hui-Ling Xu, Xiao-Shan Zeng, Jie Li, Yu-Ci Xu, Hai-Jiang Qiu, Dong-Rong Xiao (2018) *CrystEngComm* ,**20,**2430

Formula: $(C_{38} H_{28} N_4 O_{14} Zn_2)n,2(C_4 H_9 N_1 O_1)$

Compound Name: catena-[(μ-5,5'-(1,3,6,8-tetraoxo-1,3,6,8-tetrahydrobenzo[lmn][3,8]phenanthroline-2,7-diyl)di(benzene-1,3-dicarboxylato))-bis(N,N-dimethylacetamide)-di-zinc N,N-dimethylacetamide solvate]

Space Group:	Pbnm	***Cell:***	***a***	10.219(0)	***b***	36.856(0)	***c***	15.234(0)
Space Group No.:	62	***(Å,°)***	α	90.00	β	90.00	γ	90.00

R-Factor (%): 7.02 ***Temperature(K):*** 100 ***Density(g/cm^3):*** 1.238

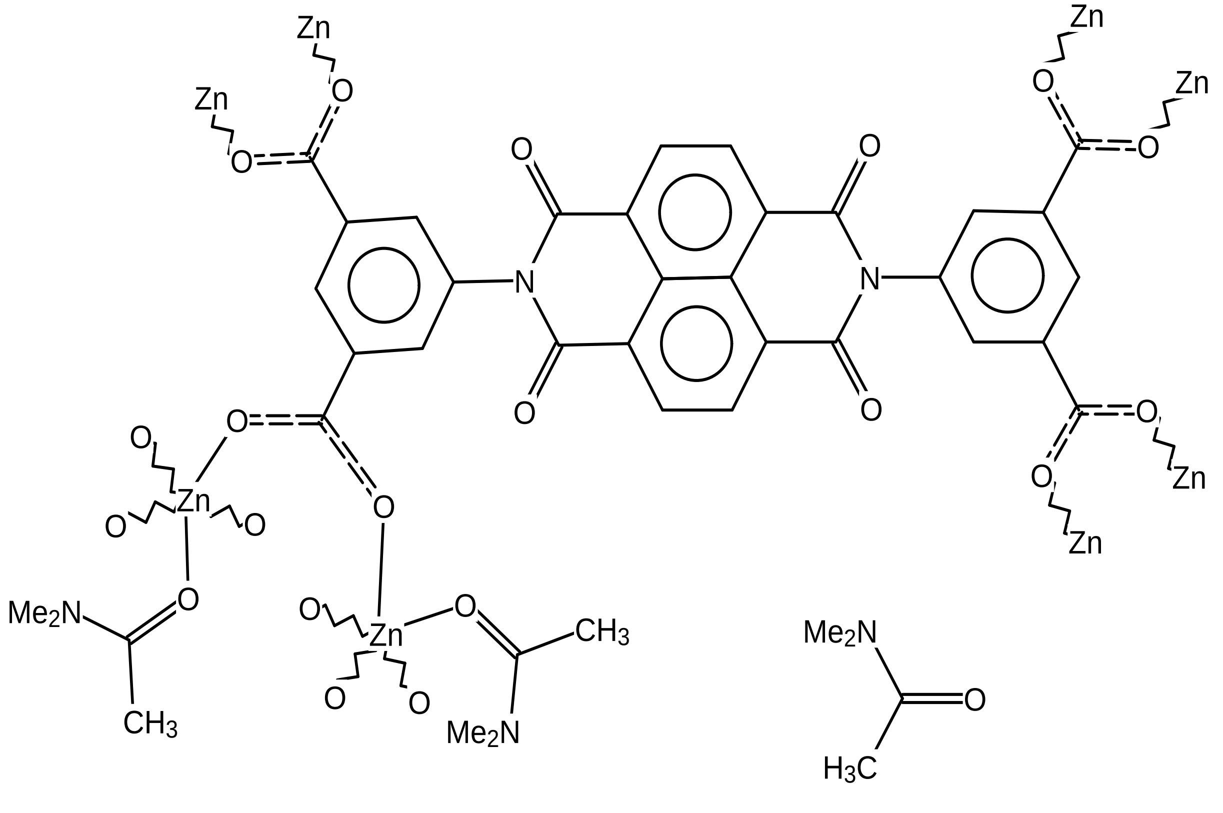

Search: search2 (Mon Jun 30 12:33:47 2025): Hit 35

KEVKII

Reference:	Hui-Ling Xu, Xiao-Shan Zeng, Jie Li, Yu-Ci Xu, Hai-Jiang Qiu, Dong-Rong Xiao (2018) *CrystEngComm* ,**20,**2430
Formula:	$(C_{81} H_{69} Ba_4 N_{11} O_{31})$n,7$(C_3 H_7 N_1 O_1)$
Compound Name:	catena-[bis(μ-5,5'-(1,3,6,8-tetraoxo-1,3,6,8-tetrahydrobenzo[lmn][3,8]phenanthroline-2,7-diyl)di(benzene-1,3-dicarboxylato))-tetrakis(μ-N,N-dimethylformamide)-tris(N,N-dimethylformamide)-tetra-barium(ii) N,N-dimethylformamide solvate]

Space Group:	P21/c	***Cell:***	***a*** 23.419(0)	***b*** 13.908(0)	***c*** 33.604(0)		
Space Group No.:	14	***(Å,°)***	α 90.00	β 90.93(0)	γ 90.00		

R-Factor (%):	9.81	***Temperature(K):***	100	***Density(g/cm^3):***	1.671

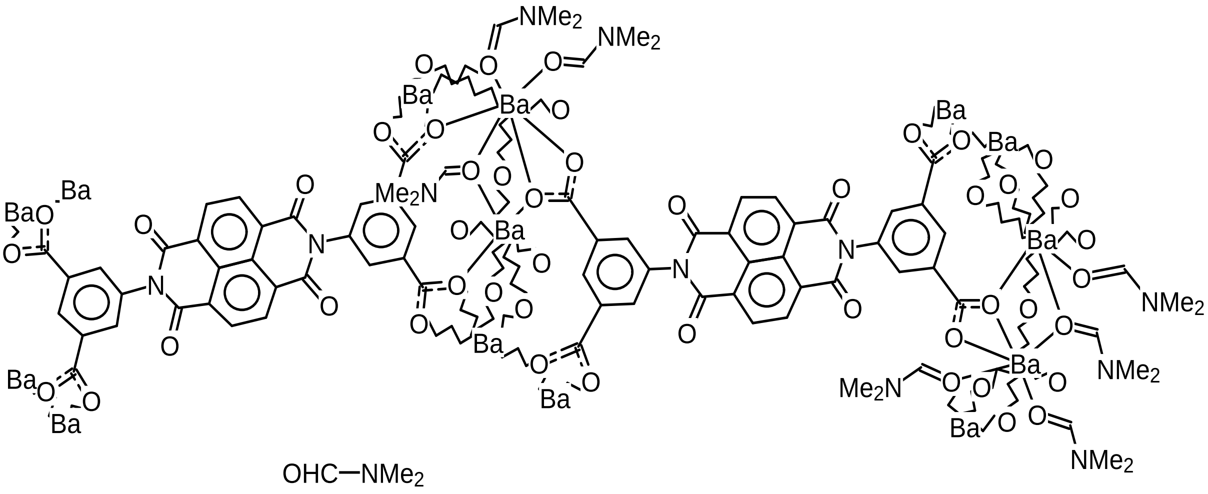

Search: search2 (Mon Jun 30 12:33:47 2025): Hit 36

KEVKOO

Reference:	Hui-Ling Xu, Xiao-Shan Zeng, Jie Li, Yu-Ci Xu, Hai-Jiang Qiu, Dong-Rong Xiao (2018) *CrystEngComm* ,**20,**2430
Formula:	$(C_{42}\ H_{38}\ Ca_2\ Cl_1\ N_5\ O_{15})n,C_4\ H_9\ N_1\ O_1$
Compound Name:	catena-[(μ-hydrogen 5,5'-(1,3,6,8-tetraoxo-1,3,6,8-tetrahydrobenzo[lmn][3,8]phenanthroline-2,7-diyl)di(benzene-1,3-dicarboxylato))-tris(N,N-dimethylacetamide)-chloro-di-calcium(ii) N,N-dimethylacetamide solvate]

Space Group:	P-1	***Cell:***	***a***	9.889(0)	***b***	16.718(0)	***c***	17.304(0)
Space Group No.:	2	***(Å,°)***	α	63.93(0)	β	78.65(0)	γ	86.53(0)

R-Factor (%):	6.17	***Temperature(K):***	292	***Density(g/cm³):***	1.392

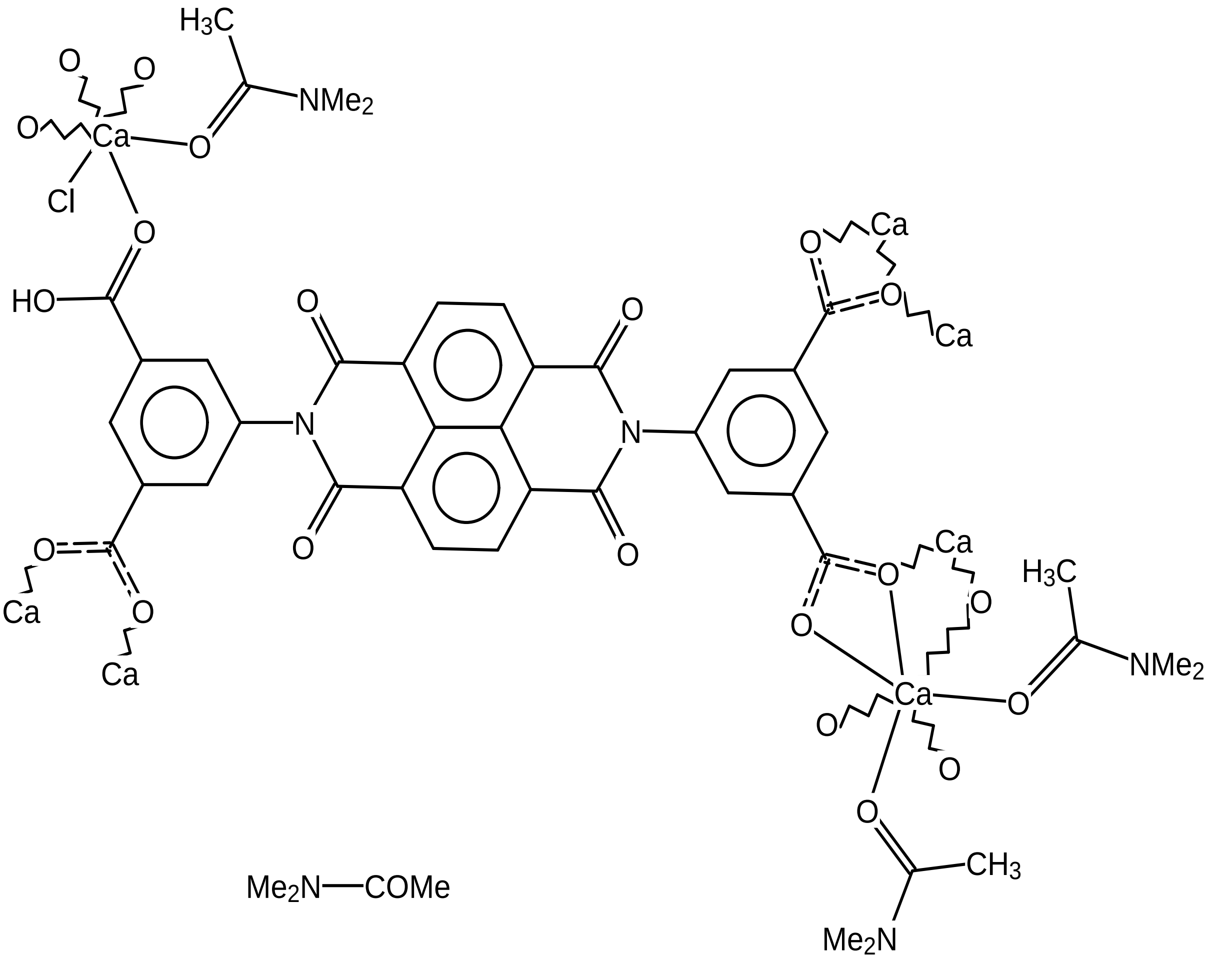

Search: search2 (Mon Jun 30 12:33:47 2025): Hit 37

KIKTIK

Reference: Yan Zhou, Lan Qin, Meng-Ke Wu, Lei Han (2018)
Cryst.Growth Des. ,**18,**5738

Formula: $(C_{60} H_{24} Cd_1 N_4 O_{24}{}^{2-})n,2n(C_2 H_8 N_1{}^{1+}),5n(C_3 H_7 N_1 O_1)$

Compound Name: catena-(bis(dimethylammonium) bis(μ-dihydrogen 5,5'-(1,3,6,8-tetraoxo-1,3,6,8-tetrahydrobenzo[lmn][3,8]phenanthroline-2,7-diyl)bis(benzene-1, 3-dicarboxylato))-cadmium(ii) dimethylformamide solvate)

Space Group:	C2/c	***Cell:***	***a*** 23.127(1)	***b*** 23.140(0)	***c*** 18.669(0)		
Space Group No.:	15	***(Å,°)***	α 90.00	β 123.08(0)	γ 90.00		

R-Factor (%): 3.95 ***Temperature(K):*** 293 ***Density(g/cm³):*** 1.392

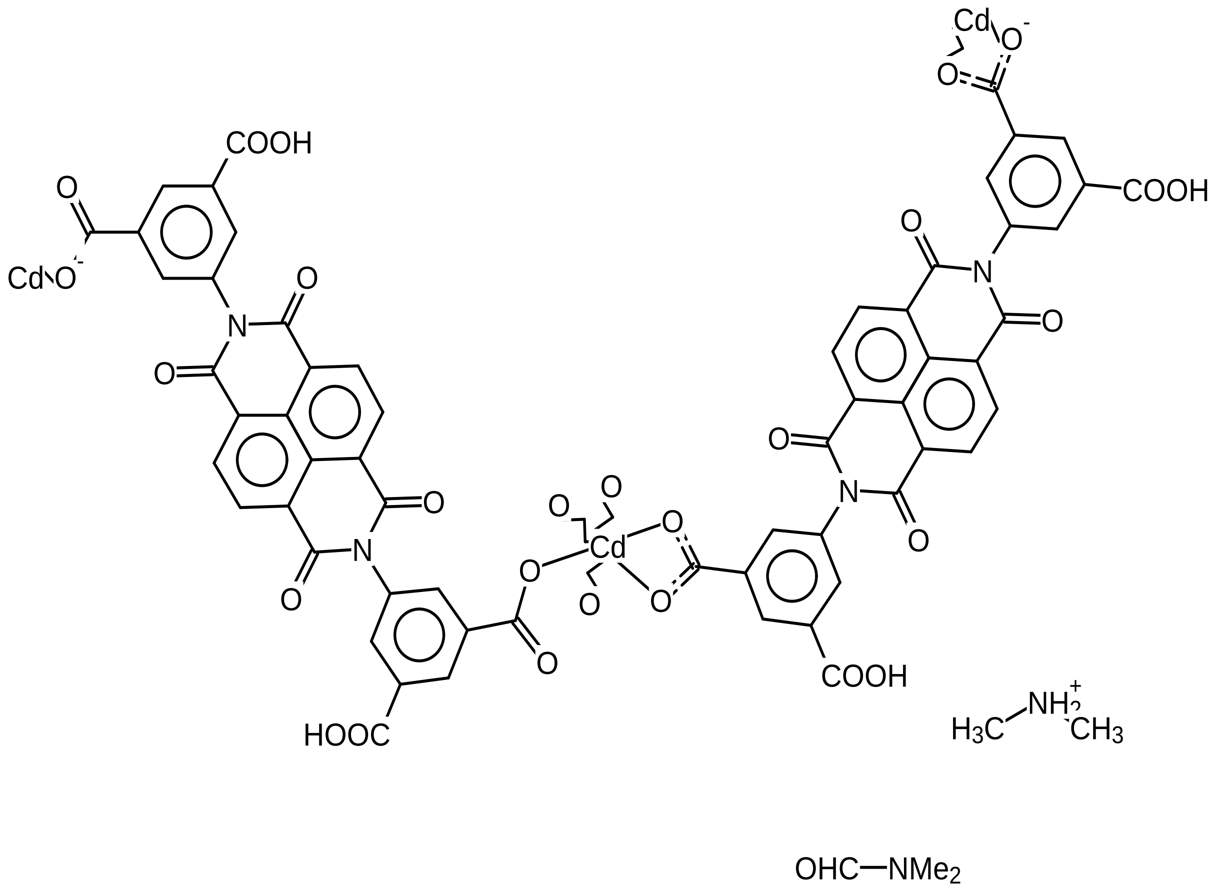

Search: search2 (Mon Jun 30 12:33:47 2025): Hit 38

KUDVIR

Reference: Li-Jun Zhai, Hong-Dao Li, Ling-Ling Gao, Jie Zhang, Yu-Lan Niu, Tuo-Ping Hu (2020) *Polyhedron* ,**180,**114417

Formula: $(C_{48} H_{30} N_6 Ni_1 O_{14})n$

Compound Name: catena-[(μ-3,3'-(1,3,6,8-tetraoxo-1,3,6,8-tetrahydrobenzo[lmn][3,8]phenanthroline-2,7-diyl)bis(5-carboxybenzoato))-(μ-1,1'-([1,1'-biphenyl]-4,4'-diyl)di(1H-imidazole))-diaqua-nickel(ii)]

Space Group:	P-1	***Cell:***	***a***	7.225(0)	***b***	8.579(0)	***c***	16.357(0)
Space Group No.:	2	***(Å,°)***	α	84.50(0)	β	89.89(0)	γ	88.89(0)

R-Factor (%): 3.31 ***Temperature(K):*** 295 ***Density(g/cm³):*** 1.602

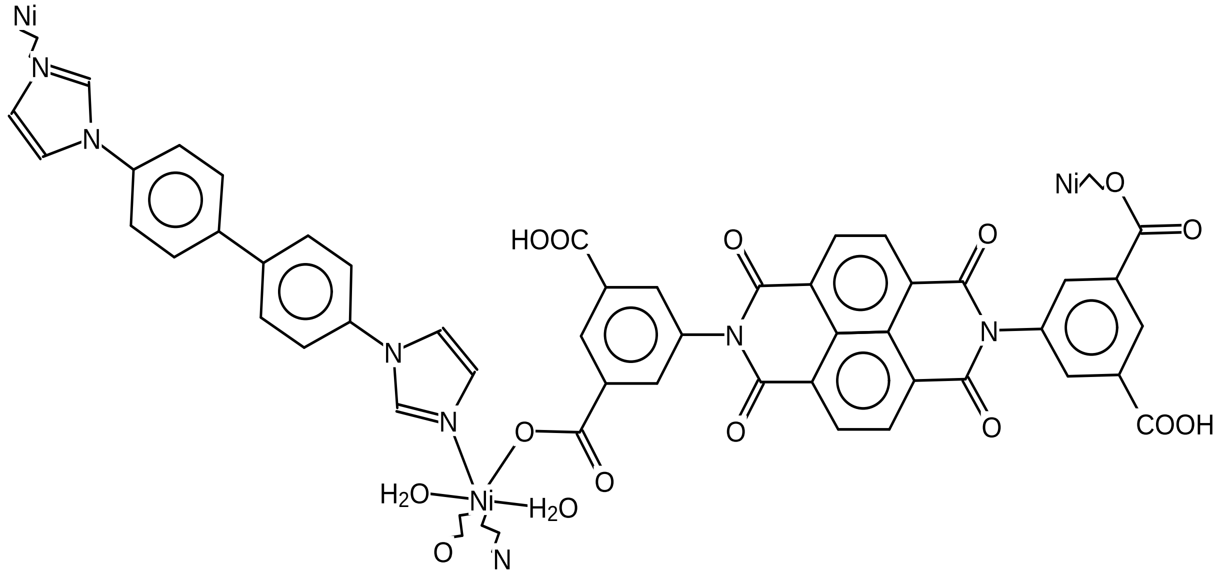

Search: search2 (Mon Jun 30 12:33:47 2025): Hit 39

KUDVOX

Reference:	Li-Jun Zhai, Hong-Dao Li, Ling-Ling Gao, Jie Zhang, Yu-Lan Niu, Tuo-Ping Hu (2020) *Polyhedron* ,**180,**114417						
Formula:	$(C_{70}\ H_{54}\ N_{10}\ Ni_2\ O_{16})n,4(C_4\ H_9\ N_1\ O_1),7(H_2\ O_1)$						
Compound Name:	catena-[(μ-5,5'-(1,3,6,8-tetraoxo-1,3,6,8-tetrahydrobenzo[lmn][3,8]phenanthroline-2,7-diyl)di(benzene-1,3-dicarboxylato))-bis(μ-1,1'-[[1,1'-biphenyl]-4,4'-diylbis(methylene)]di(1H-imidazole))-tetra-aqua-di-nickel(ii) N,N-dimethylacetamide unknown solvate heptahydrate]						
Space Group:	P21/c	***Cell:***	***a*** 14.562(1)		***b*** 21.916(2)		***c*** 16.413(1)
Space Group No.:	14	***(Å,°)***	α 90.00		β 99.03(0)		γ 90.00
R-Factor (%):	6.83	***Temperature(K):***	296		***Density(g/cm³):***		1.209

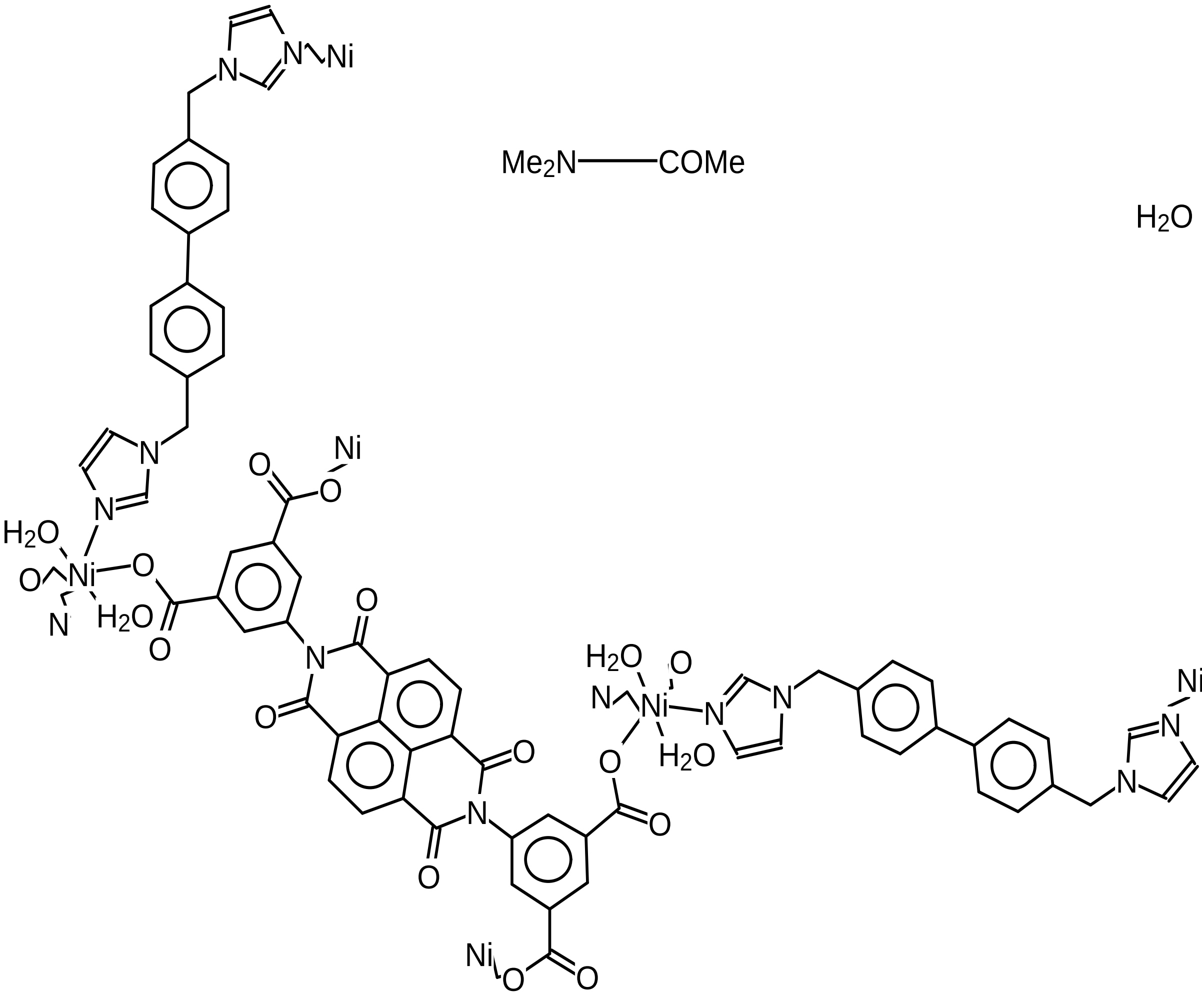

Search: search2 (Mon Jun 30 12:33:47 2025): Hit 40

KUZGAQ

Reference: Xiaokang Wang, Yutong Wang, Xia Wang, Kebin Lu, Weifeng Jiang, Pei-Pei Cui, Hongguo Hao, Fangna Dai (2020) *Dalton Trans.* ,**49,**15473

Formula: $(C_{46} H_{46} Eu_2 N_8 O_{22})n$

Compound Name: catena-[[μ-5,5'-(1,3,6,8-tetraoxo-1,3,6,8-tetrahydrobenzo[lmn][3,8]phenanthroline-2,7-diyl)bis(benzene-1,3-dicarboxylato)]-tetrakis(N,N-dimethylacetamide solvate)-bis(nitrato)-di-europium(iii) unknown solvate]

Space Group:	I2/c	***Cell:***	***a*** 16.423(0)	***b*** 11.417(0)	***c*** 34.139(0)
Space Group No.:	15	***(Å,°)***	α 90.00	β 102.65(0)	γ 90.00

R-Factor (%): 4.05 ***Temperature(K):*** 297 ***Density(g/cm³):*** 1.454

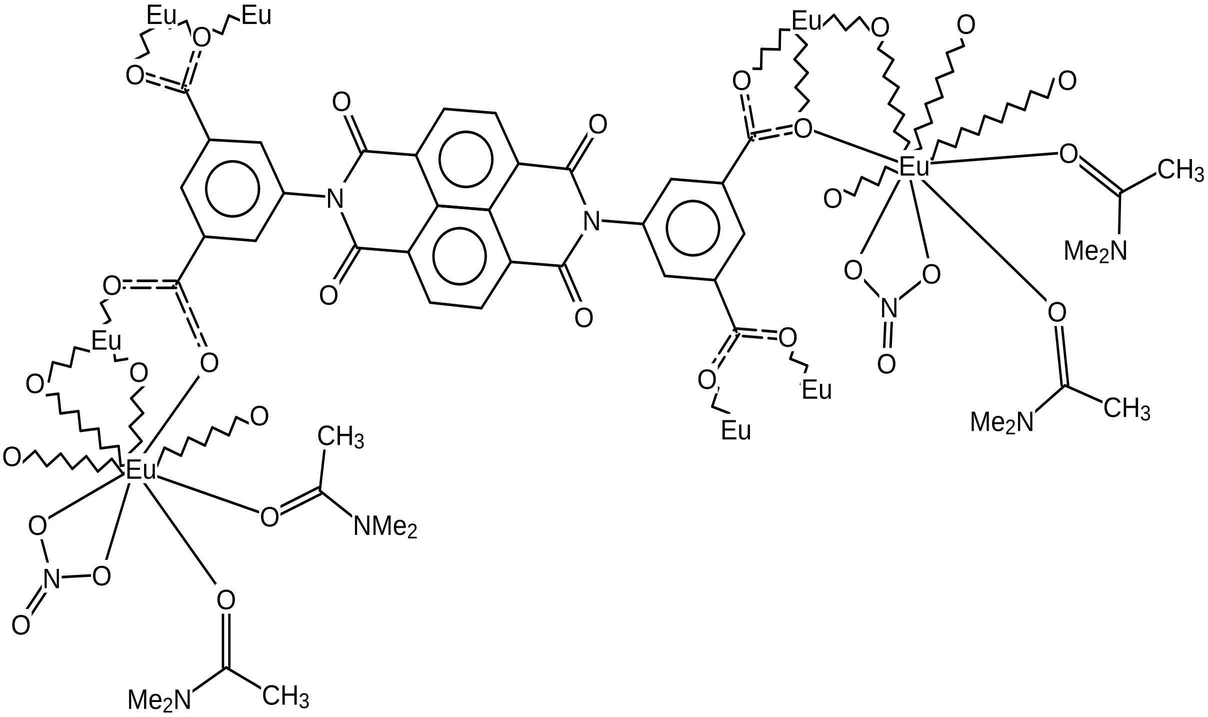

Search: search2 (Mon Jun 30 12:33:47 2025): Hit 41

KUZGEU

Reference: Xiaokang Wang, Yutong Wang, Xia Wang, Kebin Lu, Weifeng Jiang, Pei-Pei Cui, Hongguo Hao, Fangna Dai (2020) *Dalton Trans.* ,**49,**15473

Formula: $(C_{46} H_{46} N_8 Nd_2 O_{22})n$

Compound Name: catena-[[μ-5,5'-(1,3,6,8-tetraoxo-1,3,6,8-tetrahydrobenzo[lmn][3,8]phenanthroline-2,7-diyl)bis(benzene-1,3-dicarboxylato)]-tetrakis(N,N-dimethylacetamide)-bis(nitrato)-di-neodymium(iii) unknown solvate]

Space Group:	I2/c	***Cell:***	***a*** 16.585(0)	***b*** 11.528(0)	***c*** 33.991(0)
Space Group No.:	15	***(Å,°)***	α 90.00	β 102.20(0)	γ 90.00

R-Factor (%): 7.28 ***Temperature(K):*** 297 ***Density(g/cm³):*** 1.413

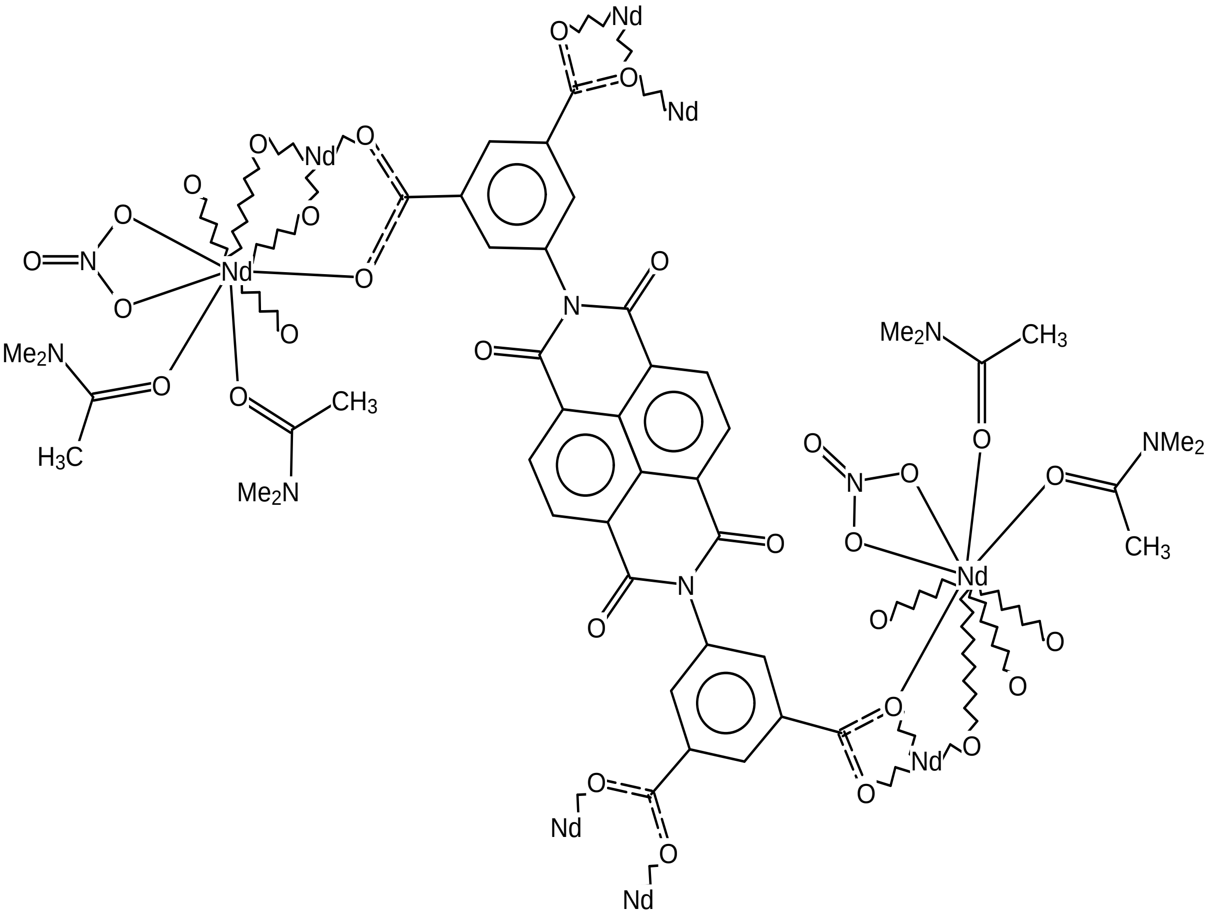

Search: search2 (Mon Jun 30 12:33:47 2025): Hit 42

KUZGIY

Reference:	Xiaokang Wang, Yutong Wang, Xia Wang, Kebin Lu, Weifeng Jiang, Pei-Pei Cui, Hongguo Hao, Fangna Dai (2020) *Dalton Trans.* ,**49,**15473
Formula:	$(C_{102}\ H_{71}\ Eu_4\ N_9\ O_{46})n,0.5(H_2\ O_1)$
Compound Name:	catena-[tris[μ-5,5'-(1,3,6,8-tetraoxo-1,3,6,8-tetrahydrobenzo[lmn][3,8]phenanthroline-2,7-diyl)bis(benzene-1,3-dicarboxylato)]-tris(N,N-dimethylacetamide)-hepta-aqua-tetra-europium(iii) hemihydrate]

Space Group:	P-1	***Cell:***	***a*** 10.906(0)	***b*** 15.057(1)	***c*** 19.040(0)
Space Group No.:	2	***(Å,°)***	α 73.97(0)	β 79.66(0)	γ 80.86(0)
R-Factor (%):	8.38	***Temperature(K):***	297	***Density(g/cm³):***	1.570

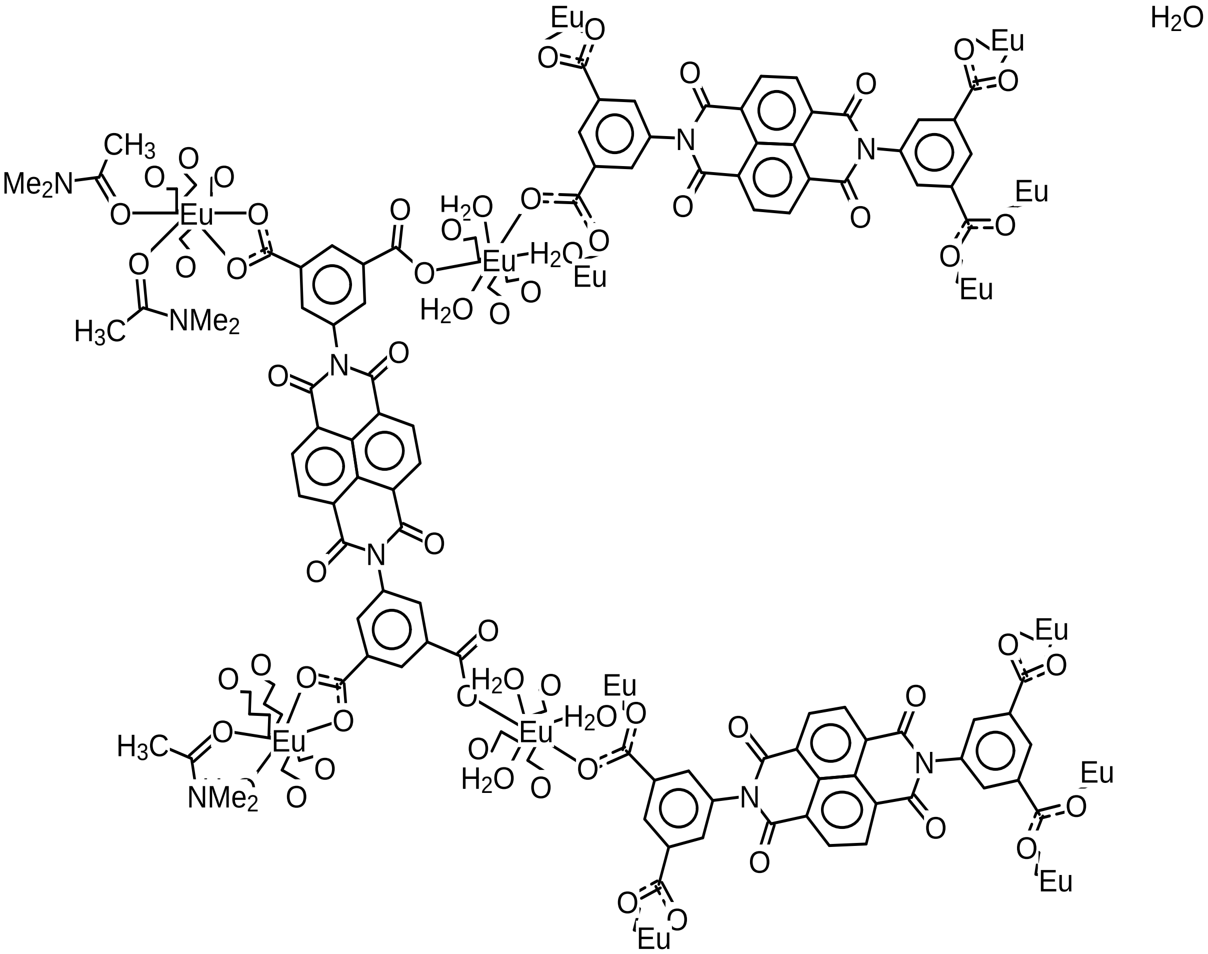

Search: search2 (Mon Jun 30 12:33:47 2025): Hit 43

KUZGOE

Reference: Xiaokang Wang, Yutong Wang, Xia Wang, Kebin Lu, Weifeng Jiang, Pei-Pei Cui, Hongguo Hao, Fangna Dai (2020) *Dalton Trans.* ,**49,**15473

Formula: $(C_{102} H_{71} Dy_4 N_9 O_{46})n,H_2 O_1$

Compound Name: catena-[tris[μ-5,5'-(1,3,6,8-tetraoxo-1,3,6,8-tetrahydrobenzo[lmn][3,8]phenanthroline-2,7-diyl)bis(benzene-1,3-dicarboxylato)]-tris(N,N-dimethylacetamide)-hepta-aqua-tetra-dysprosium(iii) monohydrate]

Space Group:	P-1	***Cell:***	***a*** 10.856(0)	***b*** 14.930(0)	***c*** 19.040(0)		
Space Group No.:	2	***(Å,°)***	α 74.91(0)	β 79.67(0)	γ 80.04(0)		

R-Factor (%): 6.26 ***Temperature(K):*** 271 ***Density(g/cm³):*** 1.616

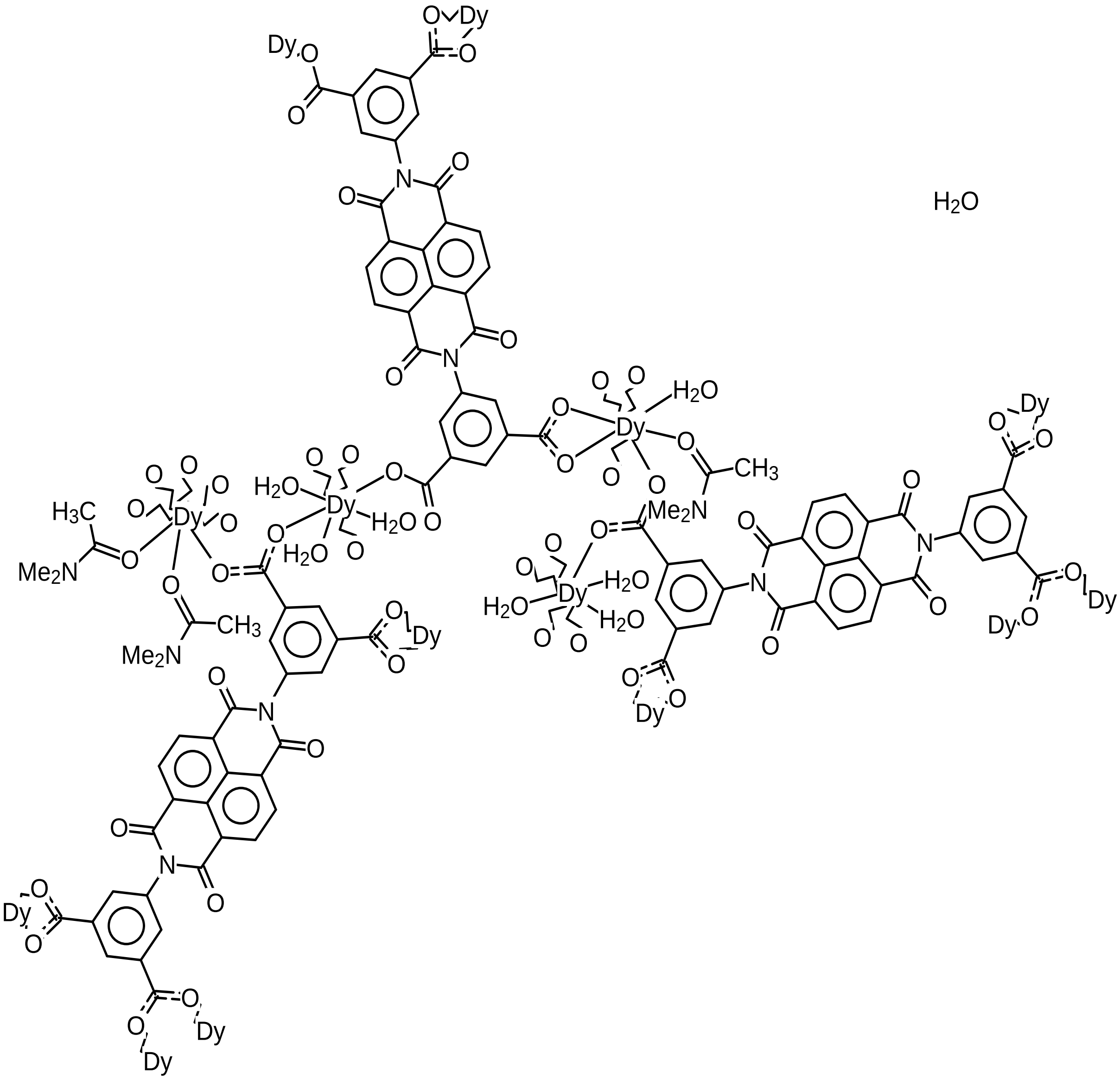

Search: search2 (Mon Jun 30 12:33:47 2025): Hit 44

KUZGUK

Reference:	Xiaokang Wang, Yutong Wang, Xia Wang, Kebin Lu, Weifeng Jiang, Pei-Pei Cui, Hongguo Hao, Fangna Dai (2020) *Dalton Trans.* ,**49,**15473
Formula:	$(C_{102} H_{71} N_9 O_{46} Sm_4)n,H_2 O_1$
Compound Name:	catena-[tris[μ-5,5'-(1,3,6,8-tetraoxo-1,3,6,8-tetrahydrobenzo[lmn][3,8]phenanthroline-2,7-diyl)bis(benzene-1,3-dicarboxylato)]-tris(N,N-dimethylacetamide)-hepta-aqua-tetra-samarium(iii) monohydrate]

Space Group:	P-1	***Cell:***	***a***	10.951(0)	***b***	14.931(0)	***c***	19.154(1)
Space Group No.:	2	***(Å,°)***	α	74.15(0)	β	79.20(0)	γ	80.10(0)

R-Factor (%):	6.09	***Temperature(K):***	279	***Density(g/cm³):***	1.572

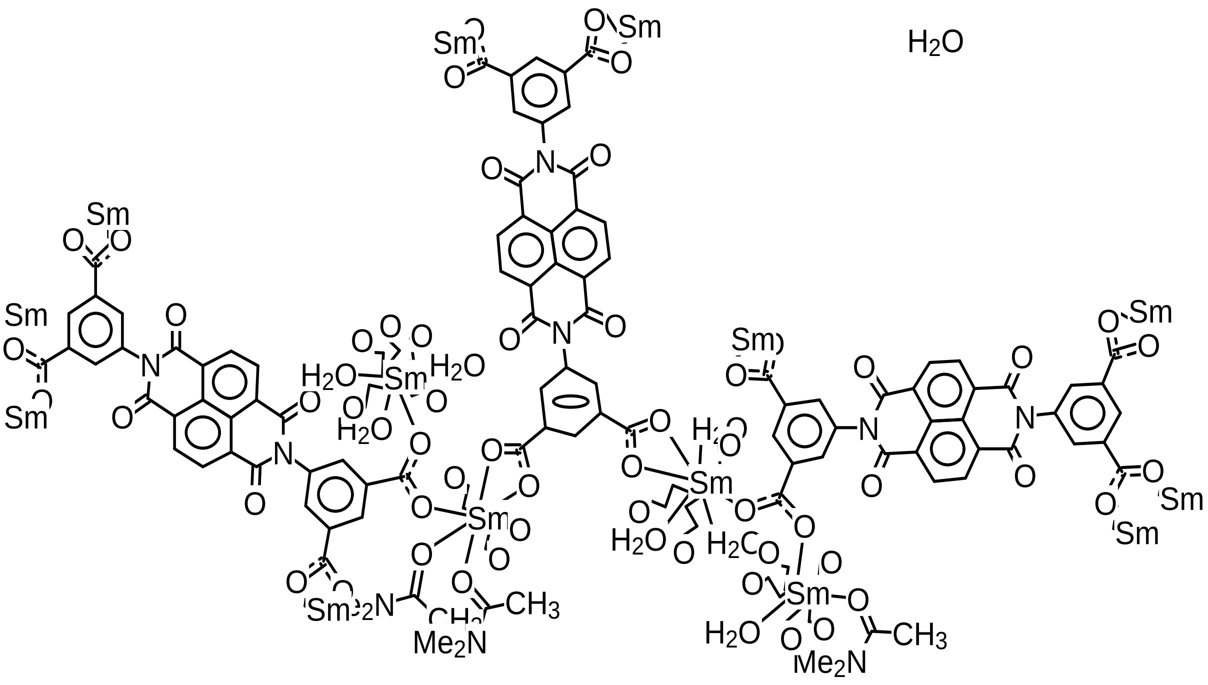

Search: search2 (Mon Jun 30 12:33:47 2025): Hit 45

LARKOI

Reference:	R.Das, T.Ezhil, C.M.Nagaraja (2021) *Cryst.Growth Des.* , **22,**598
Formula:	$(C_{34}\ H_{22}\ N_{12}\ O_{14}\ Zn_3)n$
Compound Name:	catena-[(μ-5,5'-(1,3,6,8-tetraoxo-1,3,6,8-tetrahydrobenzo[lmn][3,8]phenanthroline-2,7-diyl)bis(benzene-1,3-dicarboxylato))-bis(μ-3,5-diamino-1,2,4-triazolyl)-diaqua-tri-zinc(ii) unknown solvate]

Space Group:	Ibam	***Cell:***	***a*** 15.466(1)	***b*** 21.360(1)	***c*** 42.277(3)		
Space Group No.:	72	***(Å,°)***	α 90.00	β 90.00	γ 90.00		
R-Factor (%):	7.84	***Temperature(K):***	293	***Density(g/cm³):***	0.969		

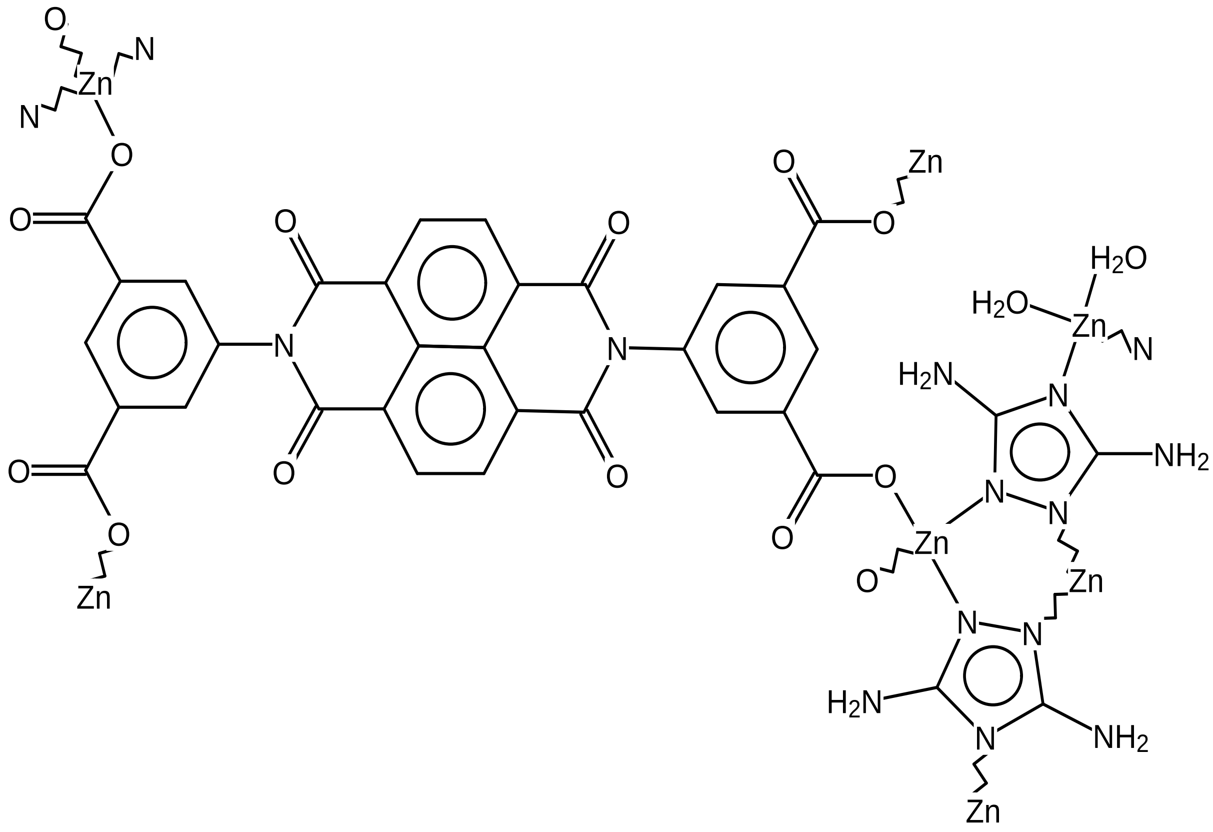

Search: search2 (Mon Jun 30 12:33:47 2025): Hit 46

LEJCEM

Reference: Jian-Jun Liu, Jia-Jia Fu, Teng Liu, Fei-Xiang Cheng (2022)
J.Solid State Chem. ,**312,**123236

Formula: $(C_{54}\ H_{66}\ Ce_{2}\ N_{10}\ O_{20}\ {}^{2+})n,Mo_{6}\ O_{19}\ {}^{2-}$

Compound Name: catena-[(μ-5,5'-(1,3,6,8-tetraoxo-1,3,6,8-tetrahydrobenzo[lmn][3,8]phenanthroline-2,7-diyl)di(benzene-1,3-dicarboxylato))-octakis(N,N-dimethylformamide)-di-cerium triadecakis(μ-oxido)-hexaoxo-hexa-molybdenum]

Space Group:	P-1	***Cell:***	***a***	10.901(0)	***b***	12.543(0)	***c***	14.319(0)
Space Group No.:	2	***(Å,°)***	α	82.59(0)	β	81.69(0)	γ	75.57(0)

R-Factor (%): 2.58 ***Temperature(K):*** 153 ***Density(g/cm³):*** 2.076

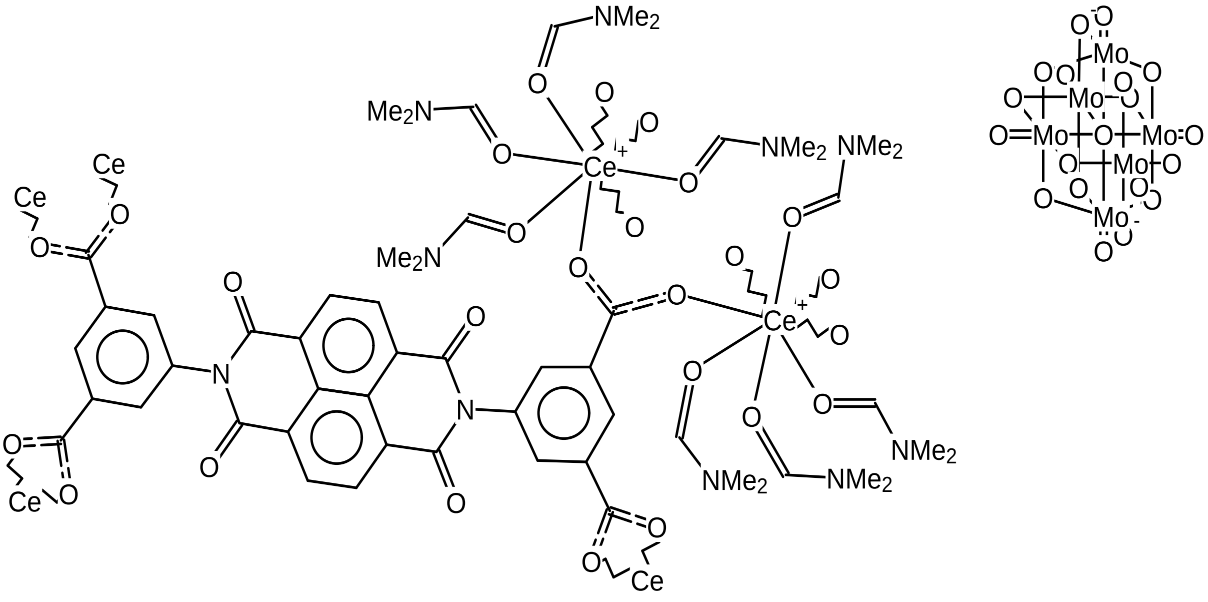

Search: search2 (Mon Jun 30 12:33:47 2025): Hit 47

LEPREG

Reference:	Guohua Ren, Lingling Gao, Xiaoqing Wang, Liming Fan, Tuoping Hu (2018) *Inorg.Chim.Acta* ,**471,**746
Formula:	$(C_{54}\ H_{34}\ Cd_2\ N_{10}\ O_{14})n,7(H_2\ O_1)$
Compound Name:	catena-[(μ-2,7-bis(3,5-dicarboxylatophenyl)-1,3,6,8-tetraoxo-1,3,6,8-tetrahydrobenzo[lmn][3,8]phenanthroline)-bis(μ-1,1'-(1,4-phenylene)di(1H-imidazole))-diaqua-di-cadmium heptahydrate]

Space Group:	P-1	***Cell:***	***a*** 10.130(0)	***b*** 10.560(0)	***c*** 13.962(0)
Space Group No.:	2	***(Å,°)***	α 103.63(0)	β 90.85(0)	γ 100.10(0)
R-Factor (%):	4.67	***Temperature(K):***	296	***Density(g/cm³):***	1.627

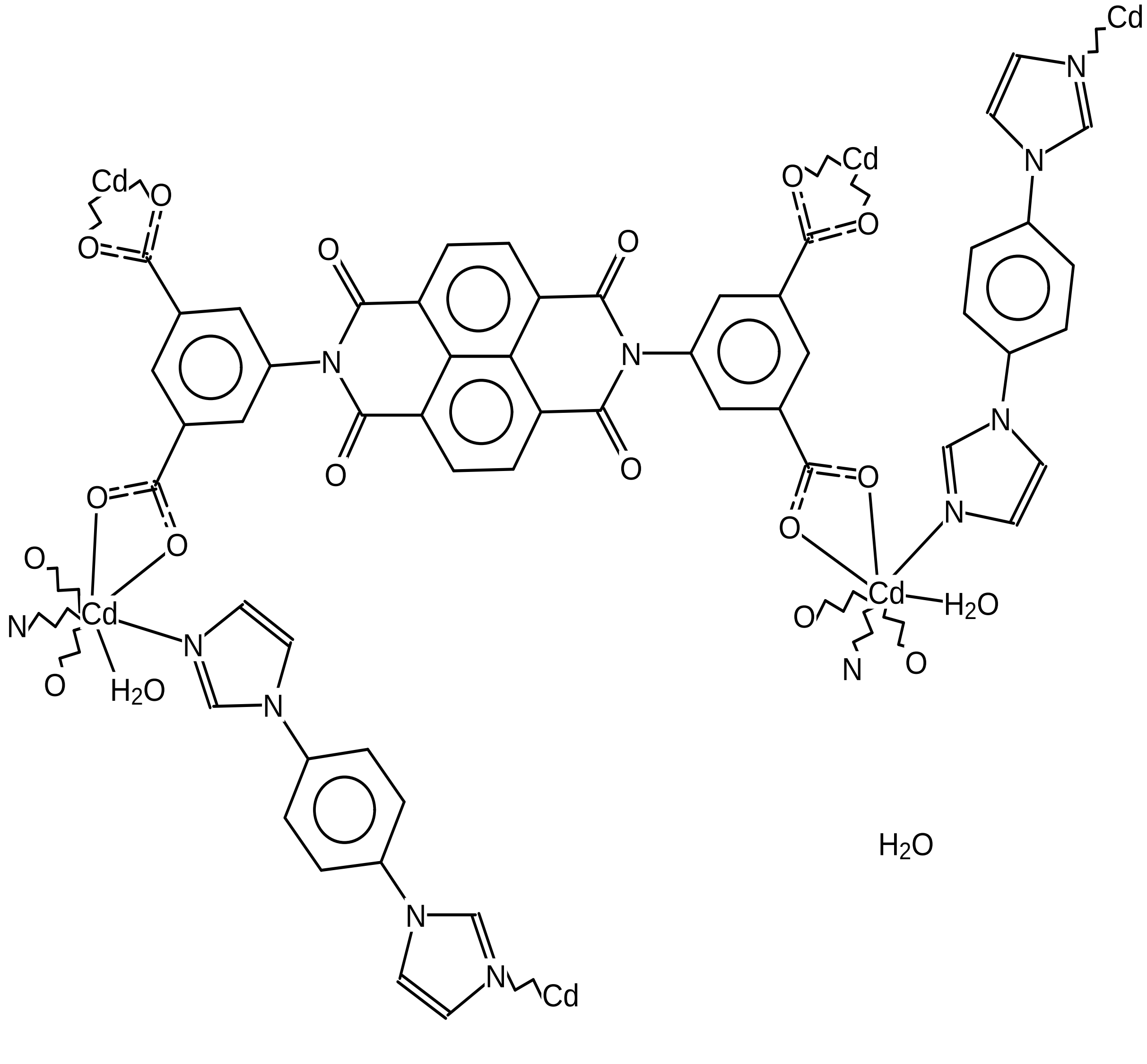

Search: search2 (Mon Jun 30 12:33:47 2025): Hit 48

LEPRIK

Reference: Guohua Ren, Lingling Gao, Xiaoqing Wang, Liming Fan, Tuoping Hu (2018) *Inorg.Chim.Acta* ,**471,**746

Formula: $(C_{48} H_{26} Cd_2 N_6 O_{13})n,H_2 O_1$

Compound Name: catena-[(μ-2,7-bis(3,5-dicarboxylatophenyl)-1,3,6,8-tetraoxo-1,3,6,8-tetrahydrobenzo[lmn][3,8]phenanthroline)-(1,1'-(biphenyl-4,4'-diyl)di(1H-imidazole))-aqua-di-cadmium monohydrate]

Space Group:	P-1	***Cell:***	***a*** 8.488(3)	***b*** 9.994(3)	***c*** 25.984(8)
Space Group No.:	2	***(Å,°)***	α 91.92(1)	β 94.71(0)	γ 108.39(0)

R-Factor (%): 5.48 ***Temperature(K):*** 293 ***Density(g/cm³):*** 1.816

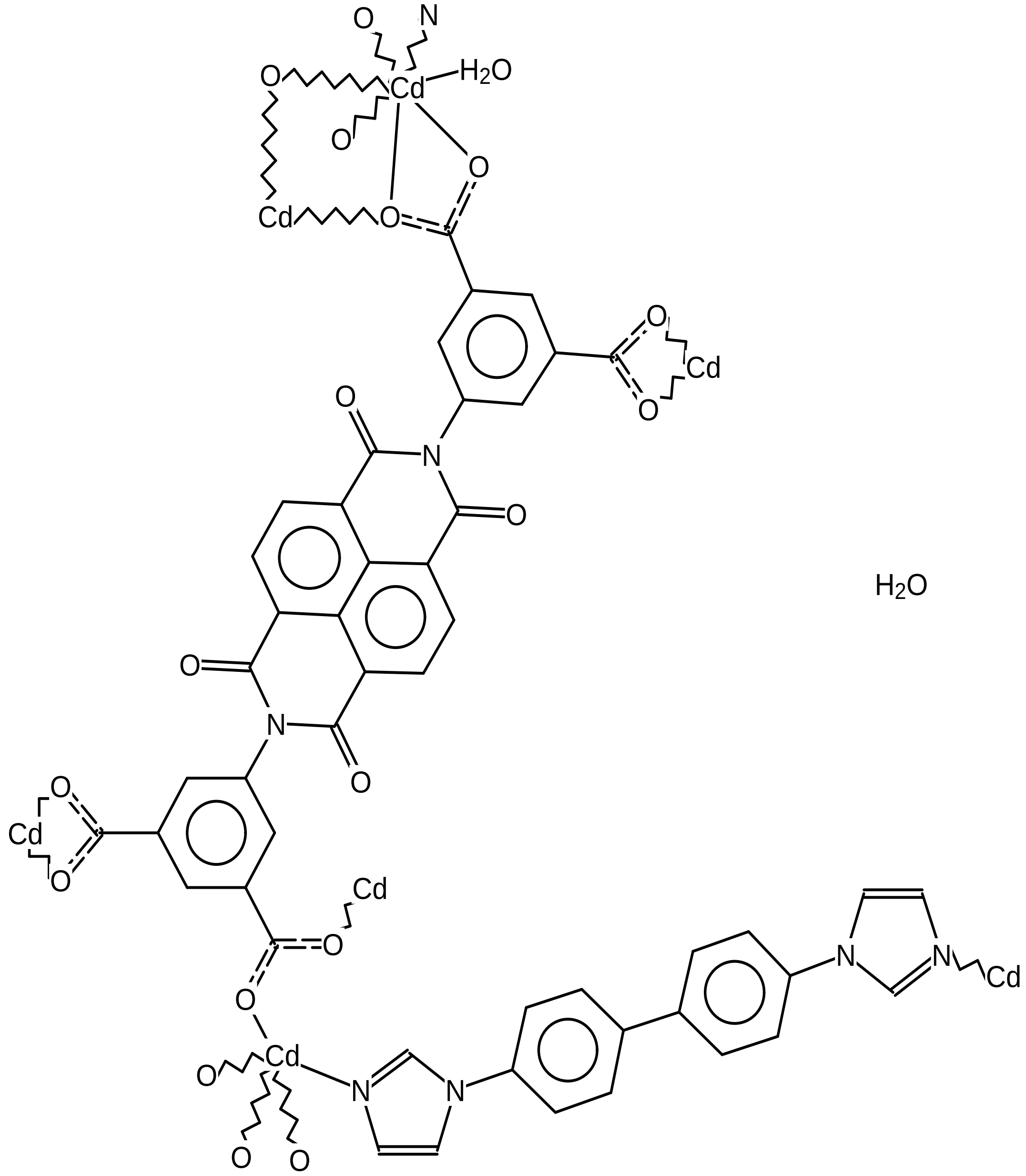

Search: search2 (Mon Jun 30 12:33:47 2025): Hit 49

LIKZAJ

Reference:	Yingxiang Ye, Zhenlin Ma, Liangji Chen, Haizhen Lin, Quanjie Lin, Lizhen Liu, Ziyin Li, Shimin Chen, Zhangjing Zhang, Shengchang Xiang (2018) *J.Mater.Chem.A* ,**6,**20822
Formula:	$(C_{30}\ H_{14}\ Cu_2\ N_2\ O_{14})n$
Compound Name:	catena-[(μ-5,5'-(1,3,6,8-tetraoxo-1,3,6,8-tetrahydrobenzo[lmn][3,8]phenanthroline-2,7-diyl)di(benzene-1,3-dicarboxylato))-diaqua-di-copper(ii) unknown solvate]
Synonym:	FJU-101

Space Group:	P4212	***Cell:***	***a***	18.247(0)	***b***	18.247(0)	***c***	16.911(0)
Space Group No.:	90	***(Å,°)***	α	90.00	β	90.00	γ	90.00

R-Factor (%):	5.13	***Temperature(K):***	150	***Density(g/cm^3):***	0.889

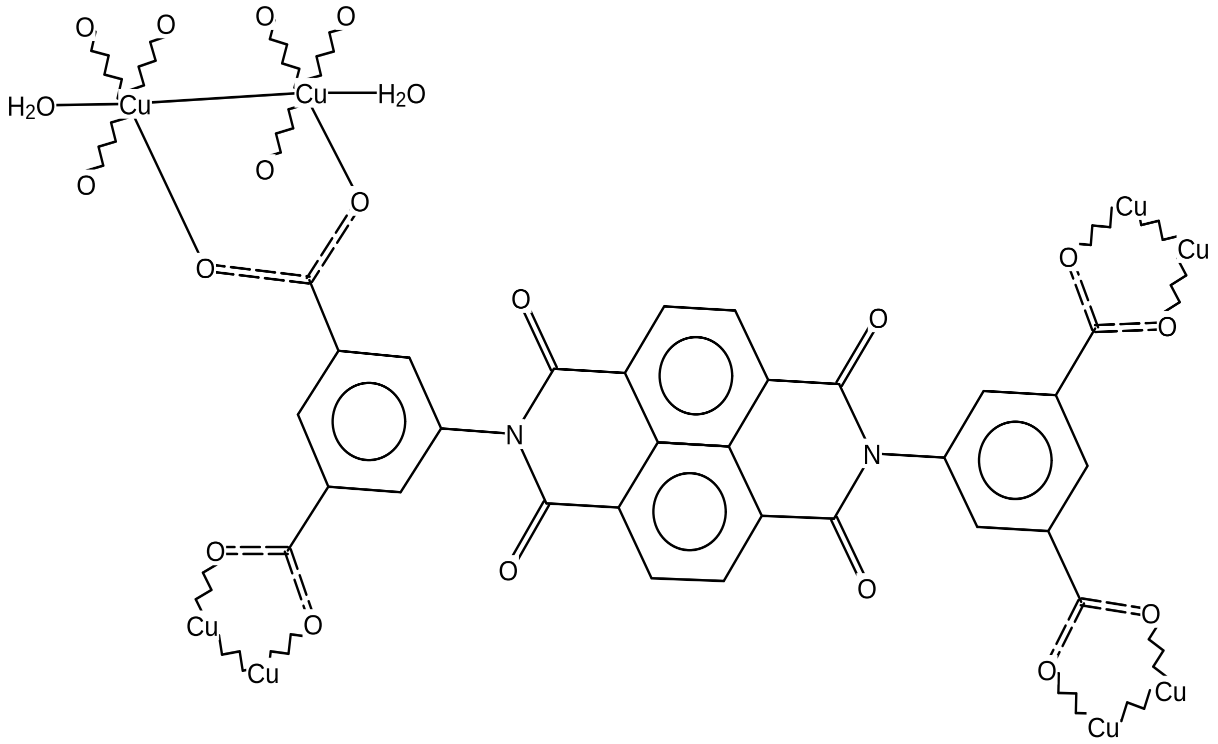

Search: search2 (Mon Jun 30 12:33:47 2025): Hit 50

LIKZEN

Reference: Yingxiang Ye, Zhenlin Ma, Liangji Chen, Haizhen Lin, Quanjie Lin, Lizhen Liu, Ziyin Li, Shimin Chen, Zhangjing Zhang, Shengchang Xiang (2018) *J.Mater.Chem.A* ,**6,**20822

Formula: $(C_{38} H_{28} Cu_2 N_4 O_{14})n$

Compound Name: catena-[(μ-5,5'-(1,3,6,8-tetraoxo-1,3,6,8-tetrahydrobenzo[lmn][3,8]phenanthroline-2,7-diyl)di(benzene-1,3-dicarboxylato))-bis(N,N-dimethylacetamide)-di-copper(ii) unknown solvate]

Synonym: FJU-102

Space Group:	Imma	***Cell:***	***a***	15.226(0)	***b***	36.442(1)	***c***	10.331(0)
Space Group No.:	74	***(Å,°)***	α	90.00	β	90.00	γ	90.00

R-Factor (%): 4.12 ***Temperature(K):*** 150 ***Density(g/cm^3):*** 1.033

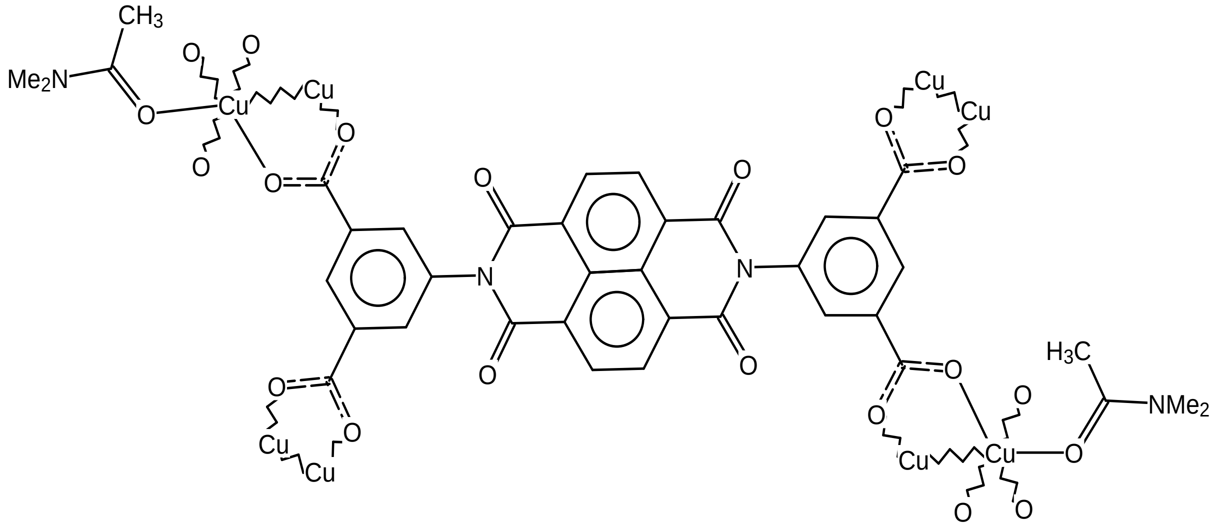

Search: search2 (Mon Jun 30 12:33:47 2025): Hit 51

LOHTIP

Reference: Seonghun Park, Juhyung Lee, Bongkyeom Kim, Chan-Yong Jung, Sang-Eun Bae, Joongoo Kang, Dohyun Moon, Jinhee Park (2024) *J.Am.Chem.Soc.* ,**146,**9293

Formula: $Ni_1{}^{2+}$,$(C_{30} H_{14} N_2 Ni_1 O_{14}{}^{2-})n$,2.88($C_3 H_7 N_1 O_1$),$H_2 O_1$,0.47($C_2 H_8 N_1{}^{1+}$),0.47($C_1$ H

Compound Name: catena-[dimethylammonium nickel (μ-5,5'-(1,3,6,8-tetraoxo-1,3,6,8-tetrahydrobenzo[lmn][3,8]phenanthroline-2,7-diyl)bis(benzene-1,3-dicarboxylato))-diaqua-nickel(ii) formate N,N-dimethylformamide solvate monohydrate]

Synonym: DGIST-10-cage

Space Group:	Fm-3m	***Cell:***	***a*** 40.633(5)	***b*** 40.633(5)	***c*** 40.633(5)
Space Group No.:	225	***(Å,°)***	α 90.00	β 90.00	γ 90.00

R-Factor (%): 8.46 ***Temperature(K):*** 298 ***Density(g/cm³):*** 0.603

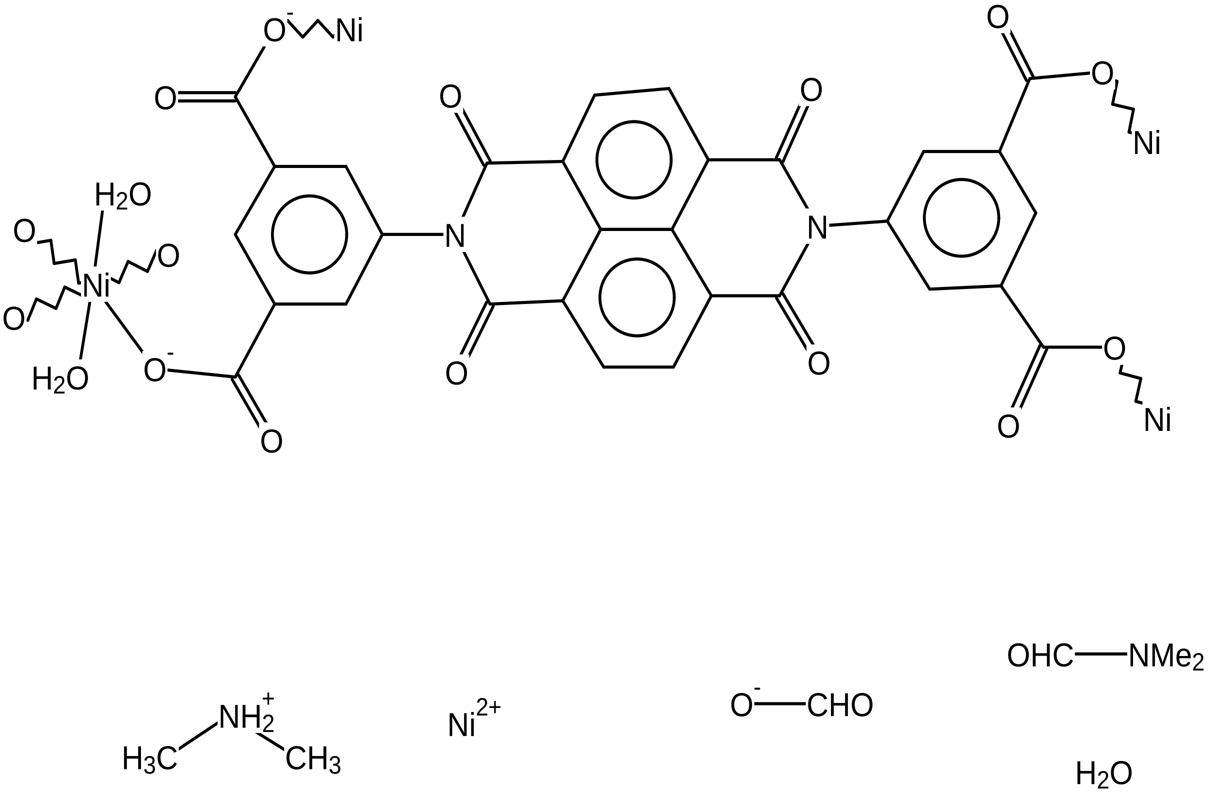

Search: search2 (Mon Jun 30 12:33:47 2025): Hit 52

LOHTOV

Reference: Seonghun Park, Juhyung Lee, Bongkyeom Kim, Chan-Yong Jung, Sang-Eun Bae, Joongoo Kang, Dohyun Moon, Jinhee Park (2024) *J.Am.Chem.Soc.* ,**146,**9293

Formula: $(C_{35}\ H_{31}\ N_4\ Ni_2\ O_{16})$n,0.06$(C_{30}\ H_{10}\ N_2\ O_{12}{}^{5-})$,1.3$(C_2\ H_8\ N_1{}^{1+})$,$C_1\ H_1\ O_2{}^{1-}$,2.49$(C_3$ [illegible]

Compound Name: catena-[dimethylammonium (5,5'-(1,3,6,8-tetraoxo-1,3,6,8-tetrahydrobenzo[lmn][3,8]phenanthroline-2,7-diyl)bis(benzene-1,3-dicarboxylate) radical anion) (μ-5,5'-(1,3,6,8-tetraoxo-1,3,6,8-tetrahydrobenzo[lmn][3,8]phenanthroline-2,7-diyl)bis(benzene-1,3-dicarboxylato) radical anion)-(μ-aqua)-(N,N-dimethylformamide)-(dimethylammonium)-diaqua-nickel(ii) formate N,N-dimethylformamide solvate hydrate]

Synonym: DGIST-10-stack

Space Group:	Pccn	***Cell:***	***a***	18.071(4)	***b***	19.069(4)	***c***	33.238(7)
Space Group No.:	56	***(Å,°)***	α	90.00	β	90.00	γ	90.00

R-Factor (%): 13.10 ***Temperature(K):*** 263 ***Density(g/cm^3):*** 1.396

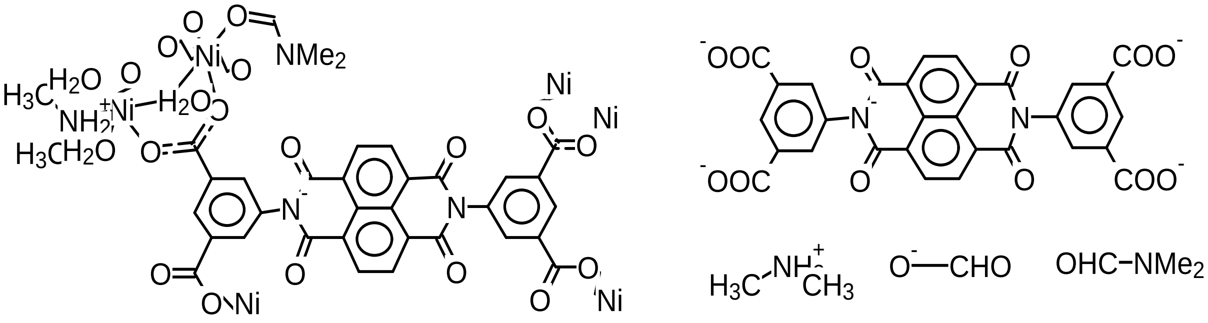

Search: search2 (Mon Jun 30 12:33:47 2025): Hit 53

LOZPOJ

Reference:	Weijun Dai, Chixian He, Sirui Li, Yuanrong Xu, Feixiang Cheng, Jian-Jun Liu (2024) *Inorg.Chem.Front.* ,**11,**5185
Formula:	$(C_{62} H_{82} Ce_2 N_{10} O_{20}{}^{2+})$2n,$Mo_{12} O_{40} Si_1{}^{4-}$
Compound Name:	catena-[bis((μ-5,5'-(1,3,6,8-tetraoxo-1,3,6,8-tetrahydrobenzo[lmn][3,8]phenanthroline-2,7-diyl)di(benzene-1,3-dicarboxylato))-octakis(N,N-dimethylacetamide)-di-cerium(iii)) tetracosakis(μ-oxo)-(μ-silicato)-dodecaoxo-dodeca-molybdenum unknown solvate]

Space Group:	P42/n	***Cell:***	***a***	23.769(3)	***b***	23.769(3)	***c***	16.668(3)
Space Group No.:	86	***(Å,°)***	α	90.00	β	90.00	γ	90.00

R-Factor (%):	6.15	***Temperature(K):***	100	***Density(g/cm³):***	1.747

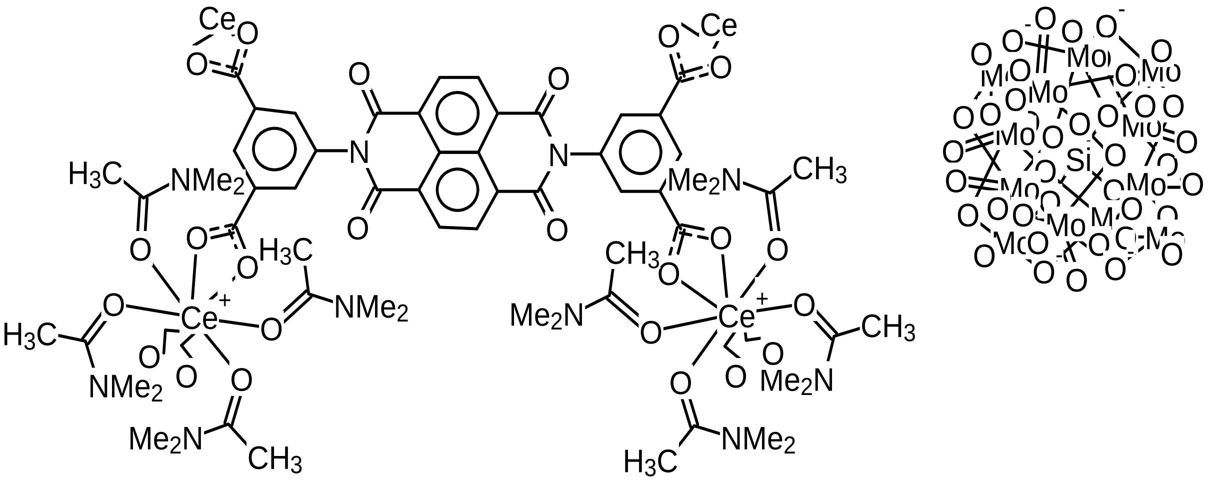

Search: search2 (Mon Jun 30 12:33:47 2025): Hit 54

MAZFEC

Reference:	Seonghun Park, Juhyung Lee, Hwakyeung Jeong, Sangeun Bae, Joongoo Kang, Dohyun Moon, Jinhee Park (2022) *Chem (Cell Press)* ,**8,**1993
Formula:	$(C_{49}\ H_{43}\ N_7\ Nd_2\ O_{21})n,1.5(C_3\ H_7\ N_1\ O_1)$
Compound Name:	catena-[(μ-5,5'-(1,3,6,8-tetraoxo-1,3,6,8-tetrahydrobenzo[lmn][3,8]phenanthroline-2,7-diyl)bis(benzene-1,3-dicarboxylato) radical anion)-(benzoato)-(nitrato)-tetrakis(N,N-dimethylformamide)-di-neodymium(iii) N,N-dimethylformamide solvate]
Synonym:	75-DGIST-4 (0.12 min)

Space Group:	C2/c	***Cell:***	***a*** 31.471(6)	***b*** 13.874(3)	***c*** 14.395(3)		
Space Group No.:	15	***(Å,°)***	α 90.00	β 95.15(3)	γ 90.00		
R-Factor (%):	8.29	***Temperature(K):***	263	***Density(g/cm³):***	1.553		

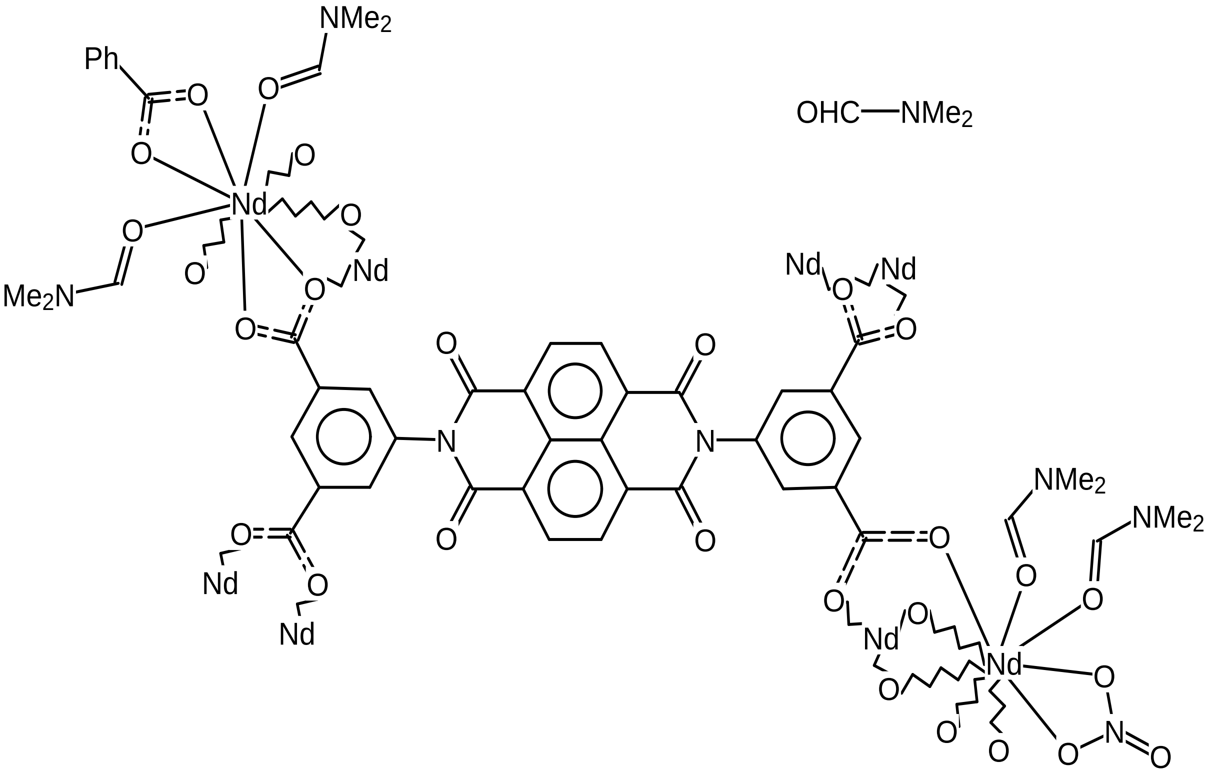

Search: search2 (Mon Jun 30 12:33:47 2025): Hit 55

MAZFEC01

Reference:	Seonghun Park, Juhyung Lee, Hwakyeung Jeong, Sangeun Bae, Joongoo Kang, Dohyun Moon, Jinhee Park (2022) *Chem (Cell Press)* ,**8,**1993
Formula:	$(C_{49} H_{43} N_7 Nd_2 O_{21})n,1.5(C_3 H_7 N_1 O_1)$
Compound Name:	catena-[(μ-5,5'-(1,3,6,8-tetraoxo-1,3,6,8-tetrahydrobenzo[lmn][3,8]phenanthroline-2,7-diyl)bis(benzene-1,3-dicarboxylato) radical anion)-(benzoato)-(nitrato)-tetrakis(N,N-dimethylformamide)-di-neodymium(iii) N,N-dimethylformamide solvate]
Synonym:	75-DGIST-4 (0.36 min)

Space Group:	C2/c	***Cell:***	***a*** 31.444(6)	***b*** 13.855(3)	***c*** 14.398(3)
Space Group No.:	15	***(Å,°)***	α 90.00	β 95.03(3)	γ 90.00
R-Factor (%):	7.34	***Temperature(K):***	263	***Density(g/cm^3):***	1.556

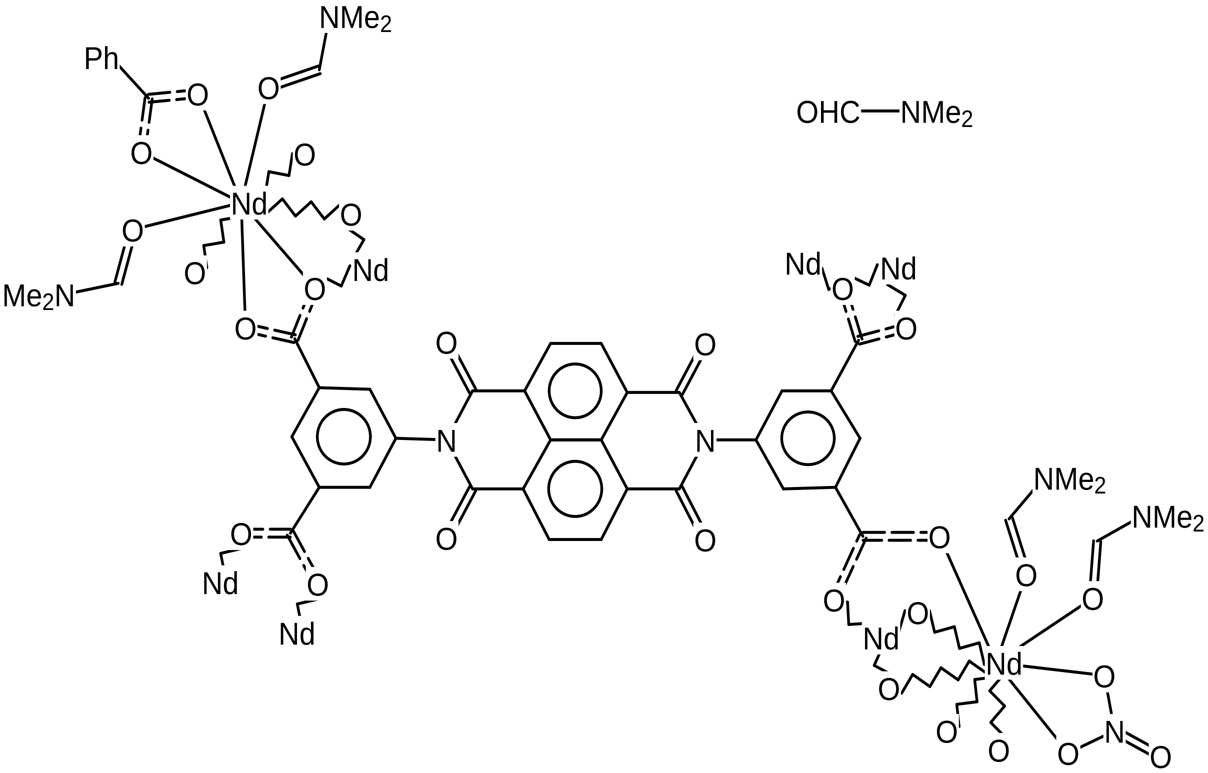

Search: search2 (Mon Jun 30 12:33:47 2025): Hit 56

MAZFEC02

Reference:	Seonghun Park, Juhyung Lee, Hwakyeung Jeong, Sangeun Bae, Joongoo Kang, Dohyun Moon, Jinhee Park (2022) *Chem (Cell Press)* ,**8,**1993							
Formula:	(C_{49} H_{43} N_7 Nd_2 O_{21})n,1.5(C_3 H_7 N_1 O_1)							
Compound Name:	catena-[(μ-5,5'-(1,3,6,8-tetraoxo-1,3,6,8-tetrahydrobenzo[lmn][3,8]phenanthroline-2,7-diyl)bis(benzene-1,3-dicarboxylato) radical anion)-(benzoato)-(nitrato)-tetrakis(N,N-dimethylformamide)-di-neodymium(iii) N,N-dimethylformamide solvate]							
Synonym:	75-DGIST-4 (0.48 min)							
Space Group:	C2/c	***Cell:***	***a***	31.431(6)	***b***	13.848(3)	***c***	14.400(3)
Space Group No.:	15	***(Å,°)***	α	90.00	β	94.99(3)	γ	90.00
R-Factor (%):	7.05	***Temperature(K):***	263		***Density(g/cm³):***	1.557		

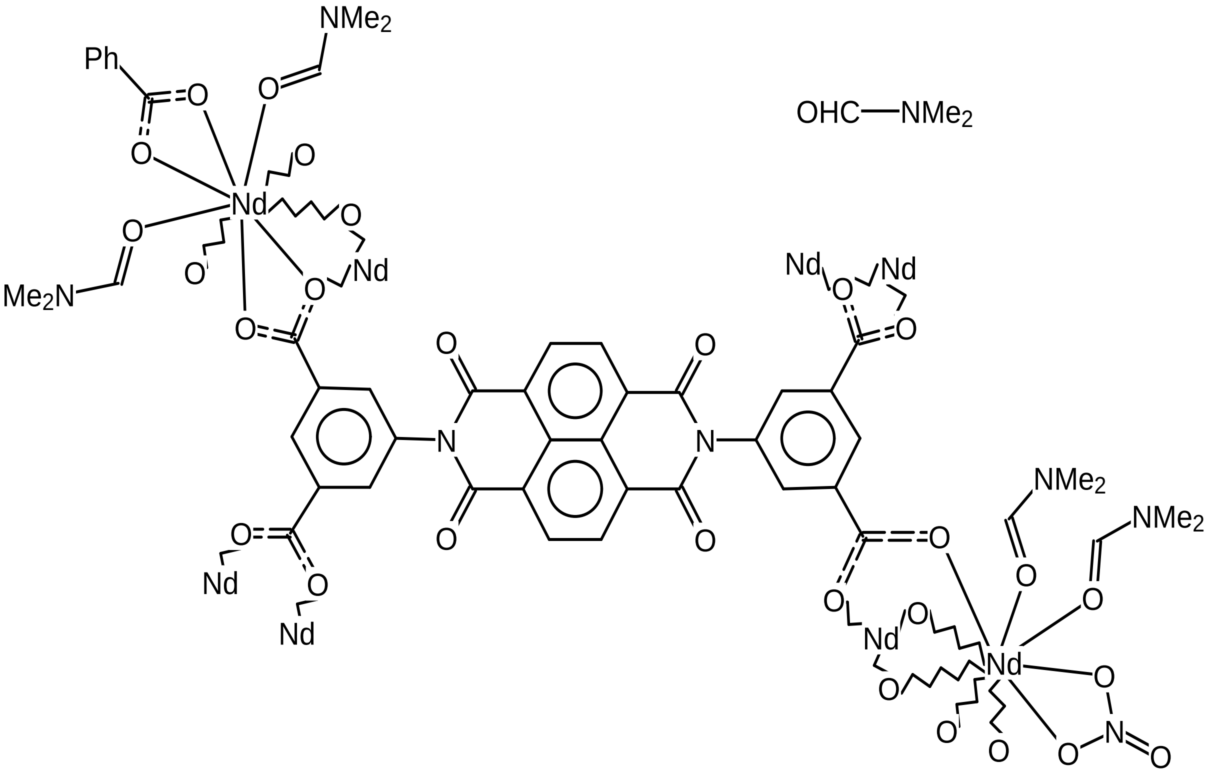

Search: search2 (Mon Jun 30 12:33:47 2025): Hit 57

MAZFEC03

Reference:	Seonghun Park, Juhyung Lee, Hwakyeung Jeong, Sangeun Bae, Joongoo Kang, Dohyun Moon, Jinhee Park (2022) *Chem (Cell Press)* ,**8,**1993
Formula:	$(C_{49}\,H_{43}\,N_7\,Nd_2\,O_{21})n,1.5(C_3\,H_7\,N_1\,O_1)$
Compound Name:	catena-[(μ-5,5'-(1,3,6,8-tetraoxo-1,3,6,8-tetrahydrobenzo[lmn][3,8]phenanthroline-2,7-diyl)bis(benzene-1,3-dicarboxylato) radical anion)-(benzoato)-(nitrato)-tetrakis(N,N-dimethylformamide)-di-neodymium(iii) N,N-dimethylformamide solvate]
Synonym:	75-DGIST-4 (1.17 min)

Space Group:	C2/c	***Cell:***	***a*** 31.388(6)	***b*** 13.818(3)	***c*** 14.408(3)		
Space Group No.:	15	***(Å,°)***	α 90.00	β 94.87(3)	γ 90.00		
R-Factor (%):	6.55	***Temperature(K):***	263	***Density(g/cm^3):***	1.562		

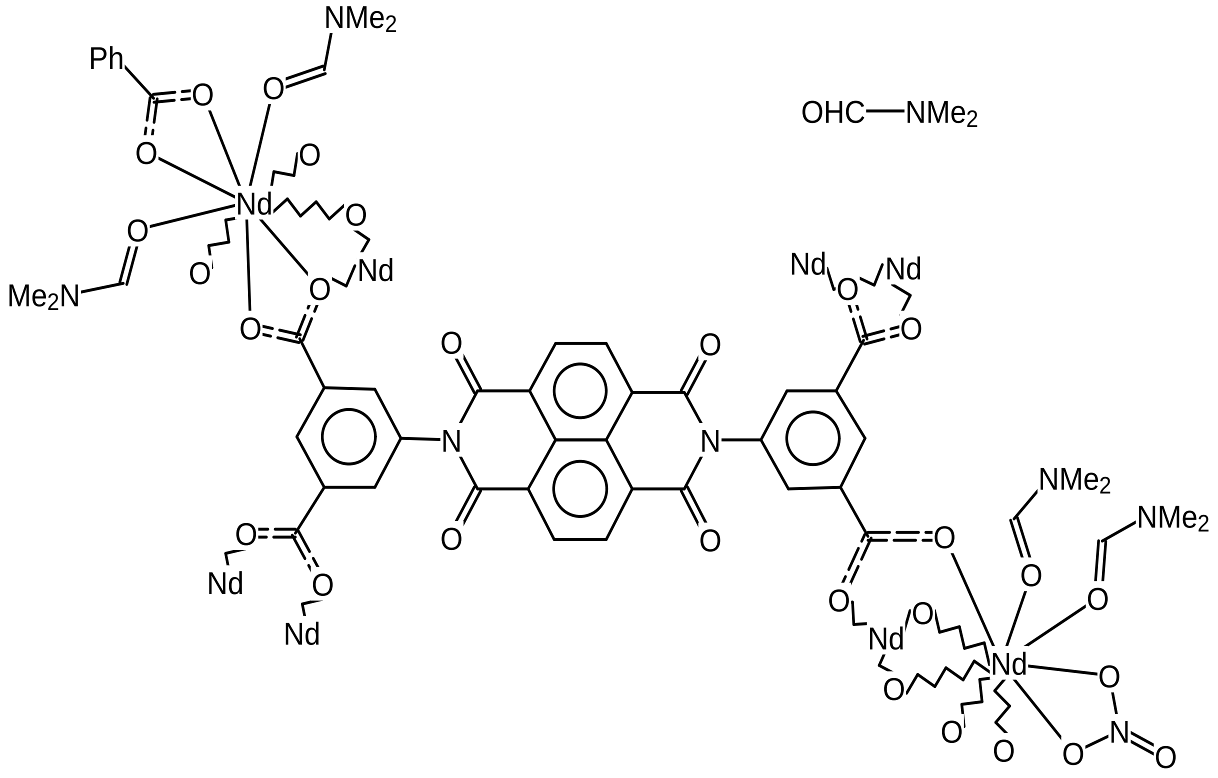

Search: search2 (Mon Jun 30 12:33:47 2025): Hit 58

MAZFEC04

Reference: Seonghun Park, Juhyung Lee, Hwakyeung Jeong, Sangeun Bae, Joongoo Kang, Dohyun Moon, Jinhee Park (2022) *Chem (Cell Press)* ,**8,**1993

Formula: $(C_{49} H_{43} N_7 Nd_2 O_{21})n,1.5(C_3 H_7 N_1 O_1)$

Compound Name: catena-[(μ-5,5'-(1,3,6,8-tetraoxo-1,3,6,8-tetrahydrobenzo[lmn][3,8]phenanthroline-2,7-diyl)bis(benzene-1,3-dicarboxylato) radical anion)-(benzoato)-(nitrato)-tetrakis(N,N-dimethylformamide)-di-neodymium(iii) N,N-dimethylformamide solvate]

Synonym: 75-DGIST-4 (1.79 min)

Space Group:	C2/c	***Cell:***	***a*** 31.379(6)	***b*** 13.793(3)	***c*** 14.414(3)		
Space Group No.:	15	***(Å,°)***	α 90.00	β 94.89(3)	γ 90.00		

R-Factor (%): 6.72 ***Temperature(K):*** 263 ***Density(g/cm³):*** 1.564

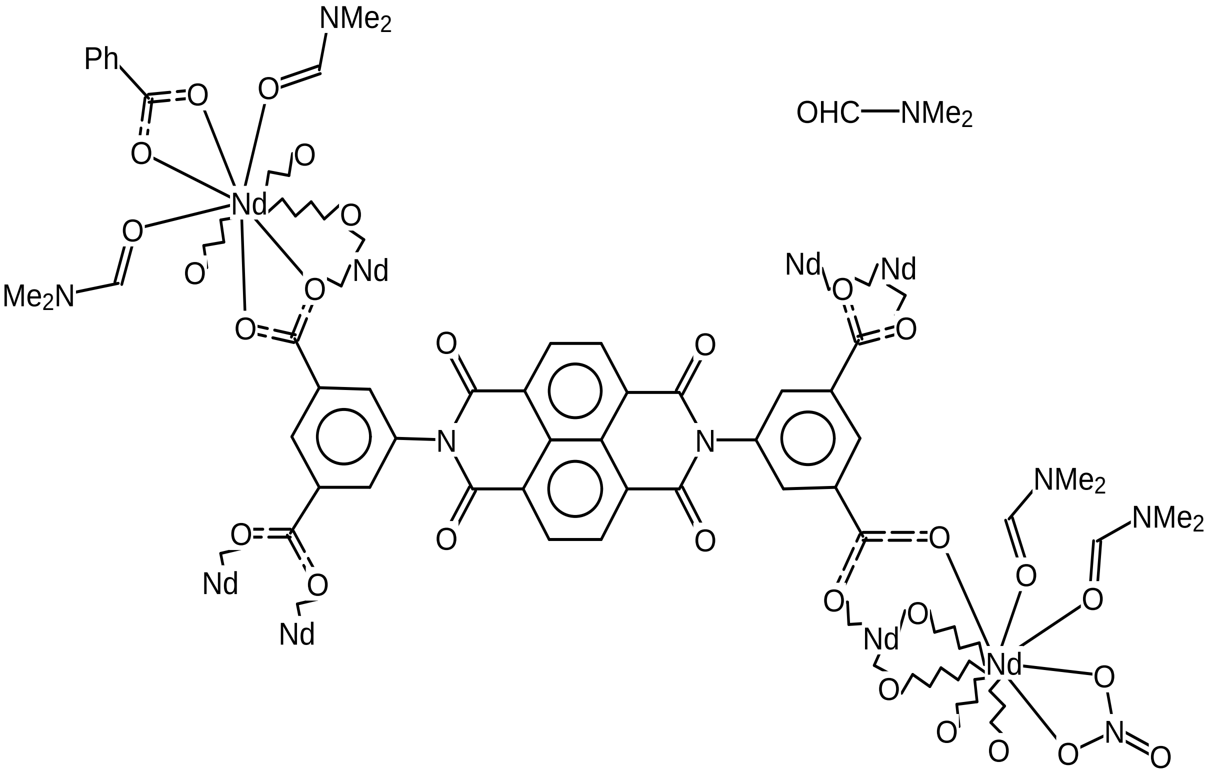

Search: search2 (Mon Jun 30 12:33:47 2025): Hit 59

MAZFEC05

Reference:	Seonghun Park, Juhyung Lee, Hwakyeung Jeong, Sangeun Bae, Joongoo Kang, Dohyun Moon, Jinhee Park (2022) *Chem (Cell Press)* ,**8,**1993
Formula:	$(C_{49} H_{43} N_7 Nd_2 O_{21})n,1.5(C_3 H_7 N_1 O_1)$
Compound Name:	catena-[(μ-5,5'-(1,3,6,8-tetraoxo-1,3,6,8-tetrahydrobenzo[lmn][3,8]phenanthroline-2,7-diyl)bis(benzene-1,3-dicarboxylato) radical anion)-(benzoato)-(nitrato)-tetrakis(N,N-dimethylformamide)-di-neodymium(iii) N,N-dimethylformamide solvate]
Synonym:	75-DGIST-4 (7.91 min)

Space Group:	C2/c	***Cell:***	***a*** 31.364(6)	***b*** 13.750(3)	***c*** 14.422(3)
Space Group No.:	15	***(Å,°)***	α 90.00	β 94.94(3)	γ 90.00

R-Factor (%): 7.07 ***Temperature(K):*** 263 ***Density(g/cm³):*** 1.569

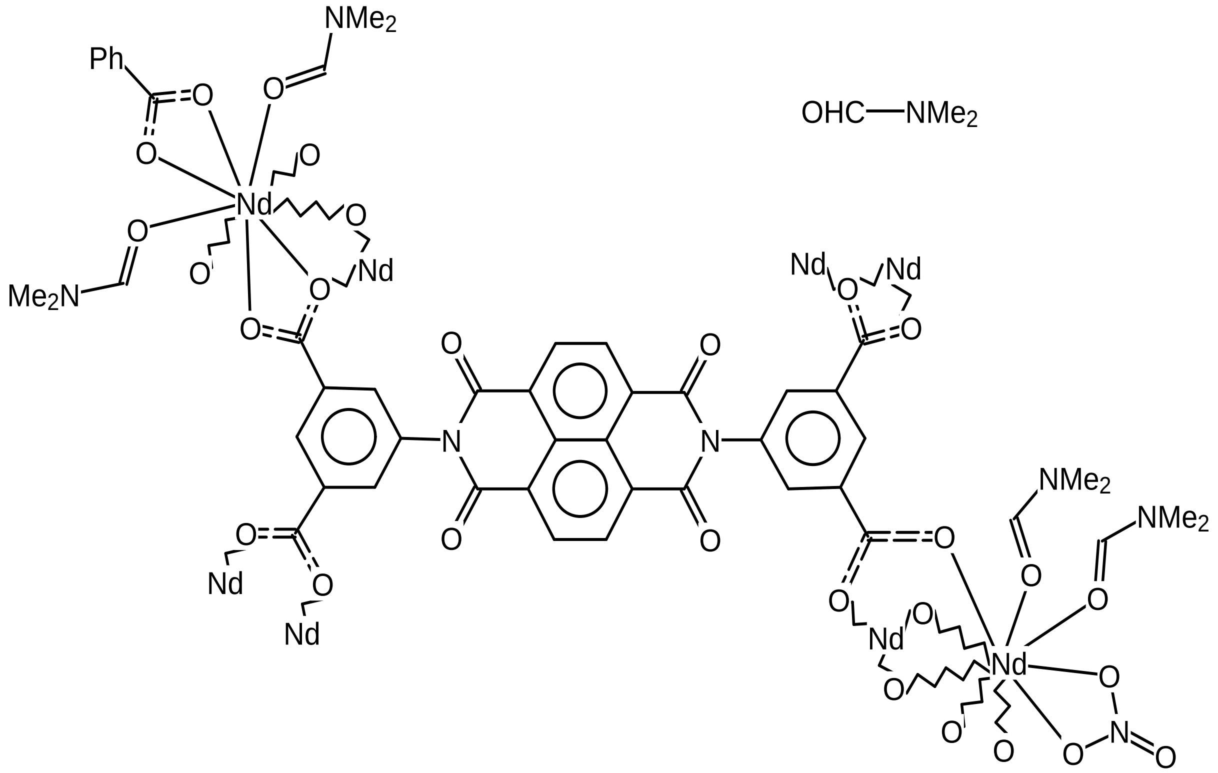

Search: search2 (Mon Jun 30 12:33:47 2025): Hit 60

MAZFEC06

Reference: Seonghun Park, Juhyung Lee, Hwakyeung Jeong, Sangeun Bae, Joongoo Kang, Dohyun Moon, Jinhee Park (2022) *Chem (Cell Press)* ,**8,**1993

Formula: $(C_{49} H_{43} N_7 Nd_2 O_{21})n,1.5(C_3 H_7 N_1 O_1)$

Compound Name: catena-[(μ-5,5'-(1,3,6,8-tetraoxo-1,3,6,8-tetrahydrobenzo[lmn][3,8]phenanthroline-2,7-diyl)bis(benzene-1,3-dicarboxylato) radical anion)-(benzoato)-(nitrato)-tetrakis(N,N-dimethylformamide)-di-neodymium(iii) N,N-dimethylformamide solvate]

Synonym: 75-DGIST-4 (23.15 min)

Space Group:	C2/c	***Cell:***	***a***	31.367(6)	***b***	13.675(3)	***c***	14.436(3)
Space Group No.:	15	***(Å,°)***	α	90.00	β	95.05(3)	γ	90.00

R-Factor (%): 7.78 ***Temperature(K):*** 263 ***Density(g/cm³):*** 1.577

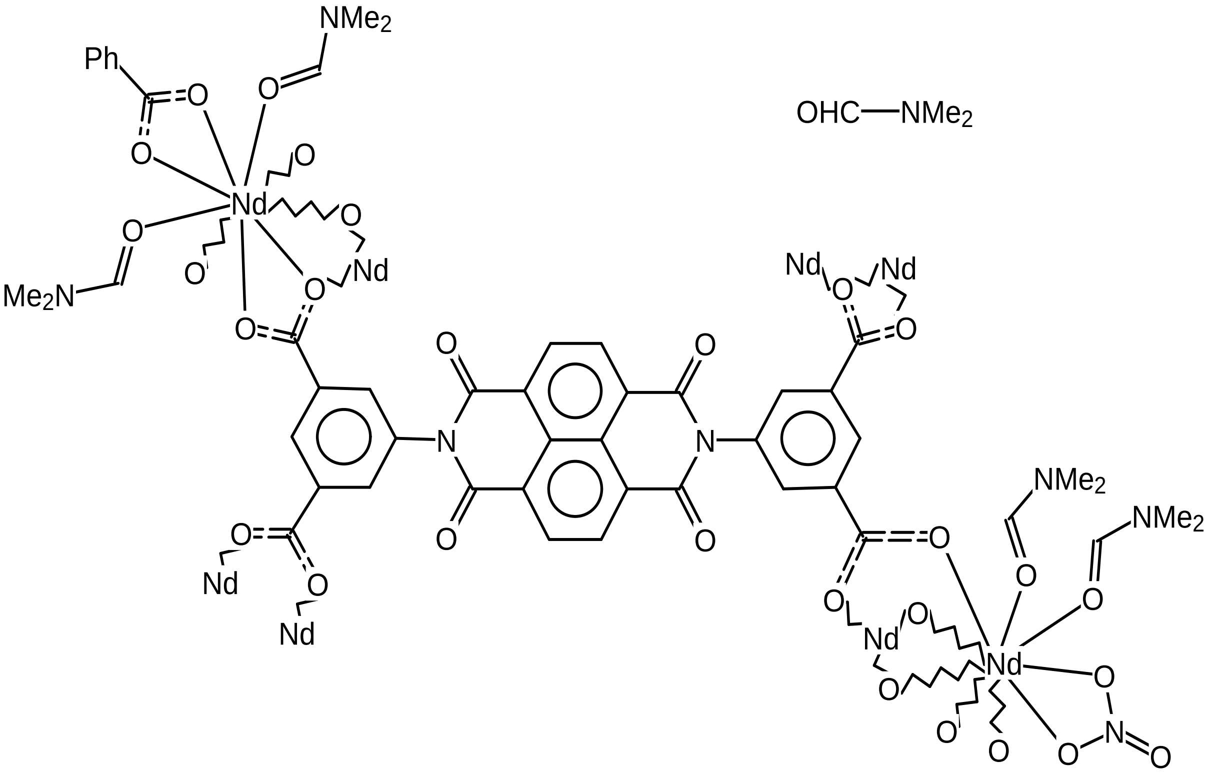

Search: search2 (Mon Jun 30 12:33:47 2025): Hit 61

MAZFEC07

Reference: Seonghun Park, Juhyung Lee, Hwakyeung Jeong, Sangeun Bae, Joongoo Kang, Dohyun Moon, Jinhee Park (2022) *Chem (Cell Press)* ,**8,**1993

Formula: $(C_{49}\ H_{43}\ N_7\ Nd_2\ O_{21})n,1.5(C_3\ H_7\ N_1\ O_1)$

Compound Name: catena-[(μ-5,5'-(1,3,6,8-tetraoxo-1,3,6,8-tetrahydrobenzo[lmn][3,8]phenanthroline-2,7-diyl)bis(benzene-1,3-dicarboxylato) radical anion)-(benzoato)-(nitrato)-tetrakis(N,N-dimethylformamide)-di-neodymium(iii) N,N-dimethylformamide solvate]

Synonym: 75-DGIST-4 (35.27 min)

Space Group:	C2/c	***Cell:***	***a***	31.382(6)	***b***	13.621(3)	***c***	14.437(3)
Space Group No.:	15	***(Å,°)***	α	90.00	β	95.23(3)	γ	90.00

R-Factor (%): 8.54 ***Temperature(K):*** 263 ***Density(g/cm^3):*** 1.582

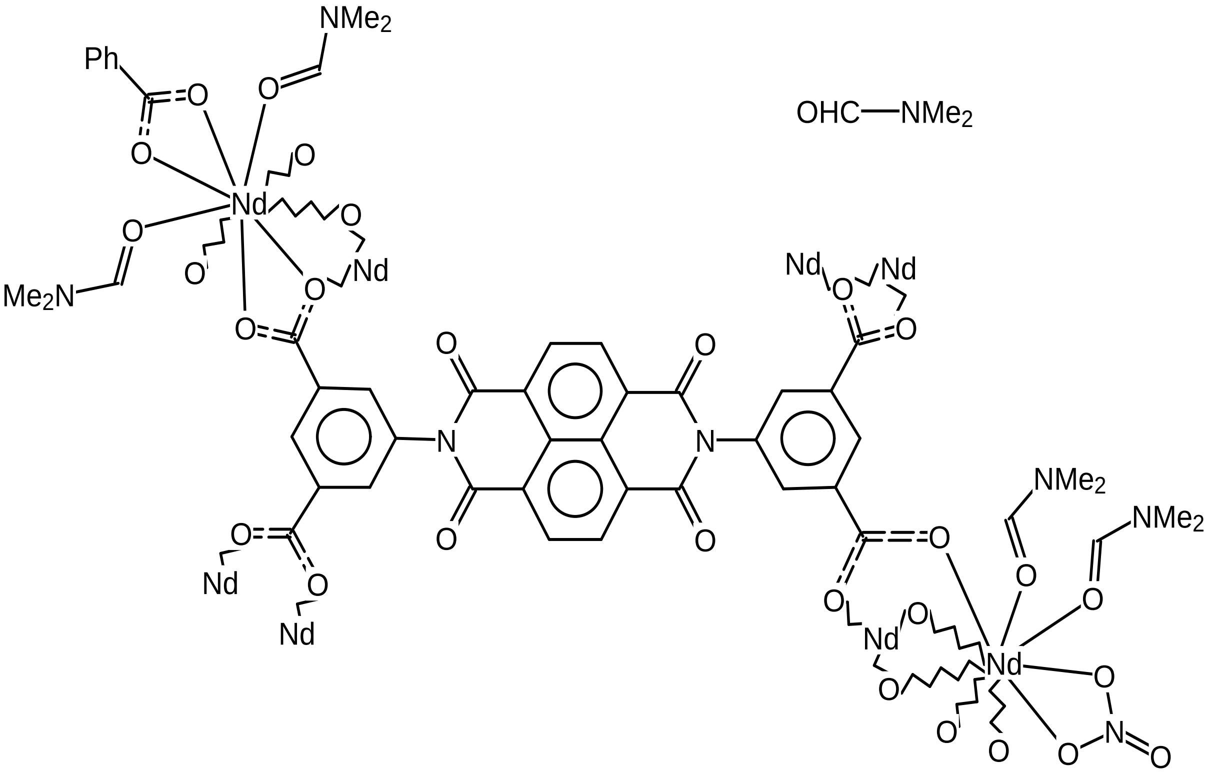

Search: search2 (Mon Jun 30 12:33:47 2025): Hit 62

MAZFEC08

Reference: Seonghun Park, Juhyung Lee, Hwakyeung Jeong, Sangeun Bae, Joongoo Kang, Dohyun Moon, Jinhee Park (2022) *Chem (Cell Press)* ,**8,**1993

Formula: $(C_{49} H_{43} N_7 Nd_2 O_{21})n,1.5(C_3 H_7 N_1 O_1)$

Compound Name: catena-[(μ-5,5'-(1,3,6,8-tetraoxo-1,3,6,8-tetrahydrobenzo[lmn][3,8]phenanthroline-2,7-diyl)bis(benzene-1,3-dicarboxylato) radical anion)-(benzoato)-(nitrato)-tetrakis(N,N-dimethylformamide)-di-neodymium(iii) N,N-dimethylformamide solvate]

Synonym: 75-DGIST-4 (125.56 min)

Space Group:	C2/c	***Cell:***	***a*** 31.523(6)	***b*** 13.437(3)	***c*** 14.443(3)
Space Group No.:	15	***(Å,°)***	α 90.00	β 96.03(3)	γ 90.00

R-Factor (%): 14.15 ***Temperature(K):*** 263 ***Density(g/cm³):*** 1.598

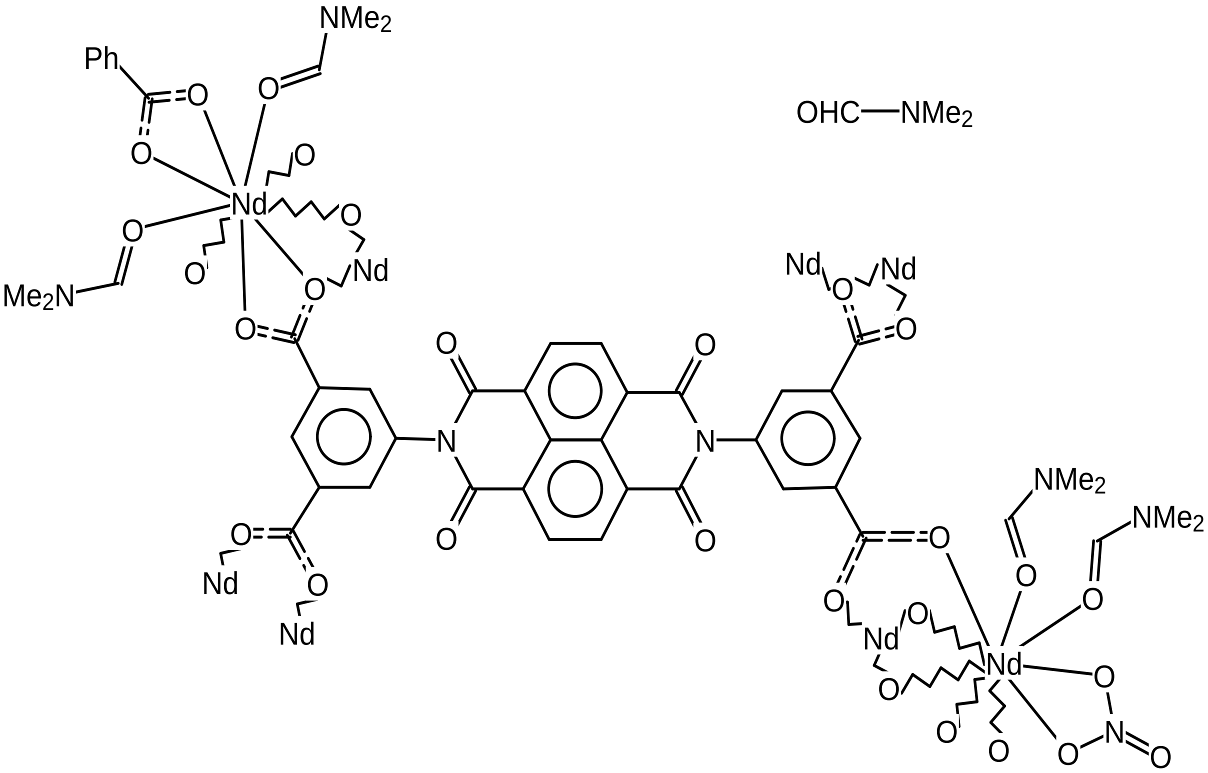

Search: search2 (Mon Jun 30 12:33:47 2025): Hit 63

MAZFEC09

Reference: Seonghun Park, Juhyung Lee, Hwakyeung Jeong, Sangeun Bae, Joongoo Kang, Dohyun Moon, Jinhee Park (2022) *Chem (Cell Press)* ,**8,**1993

Formula: $(C_{49}\ H_{43}\ N_7\ Nd_2\ O_{21})n,1.5(C_3\ H_7\ N_1\ O_1)$

Compound Name: catena-[(μ-5,5'-(1,3,6,8-tetraoxo-1,3,6,8-tetrahydrobenzo[lmn][3,8]phenanthroline-2,7-diyl)bis(benzene-1,3-dicarboxylato) radical anion)-(benzoato)-(nitrato)-tetrakis(N,N-dimethylformamide)-di-neodymium(iii) N,N-dimethylformamide solvate]

Synonym: 75-DGIST-4 (845.68 min)

Space Group:	C2/c	***Cell:***	***a*** 31.416(6)	***b*** 13.346(3)	***c*** 14.436(3)
Space Group No.:	15	***(Å,°)***	α 90.00	β 95.74(3)	γ 90.00

R-Factor (%): 13.94 ***Temperature(K):*** 263 ***Density(g/cm³):*** 1.615

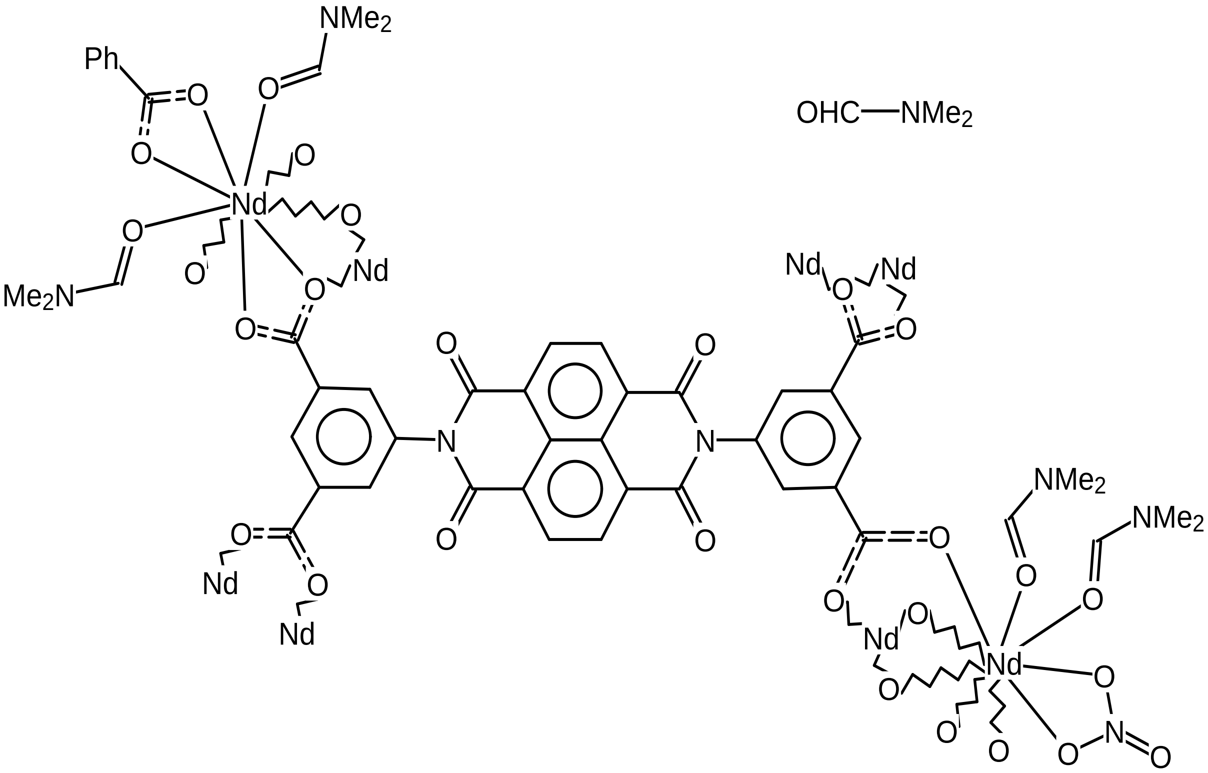

Search: search2 (Mon Jun 30 12:33:47 2025): Hit 64

MAZFIG

Reference: Seonghun Park, Juhyung Lee, Hwakyeung Jeong, Sangeun Bae, Joongoo Kang, Dohyun Moon, Jinhee Park (2022) *Chem (Cell Press)* ,**8,**1993

Formula: $(C_{49} H_{43} N_6 Nd_2 O_{18}{}^{1+})n,0.6(C_3 H_7 N_1 O_1),0.46(C_2 H_8 N_1),0.12(C_1 H_1 O_2),N_1 O_3{}^{1-}$

Compound Name: catena-[dimethylammonium (μ-5,5'-(1,3,6,8-tetraoxo-1,3,6,8-tetrahydrobenzo[lmn][3,8]phenanthroline-2,7-diyl)bis(benzene-1,3-dicarboxylato) radical anion)-(benzoato)-tetrakis(N,N-dimethylformamide) -di-neodymium(iii) formate nitrate N,N-dimethylformamide solvate]

Synonym: 150-DGIST-4

Space Group:	C2/c	***Cell:***	***a***	33.149(1)	***b***	14.065(0)	***c***	14.298(0)
Space Group No.:	15	***(Å,°)***	α	90.00	β	100.26(0)	γ	90.00

R-Factor (%): 8.48 ***Temperature(K):*** 173 ***Density(g/cm³):*** 1.443

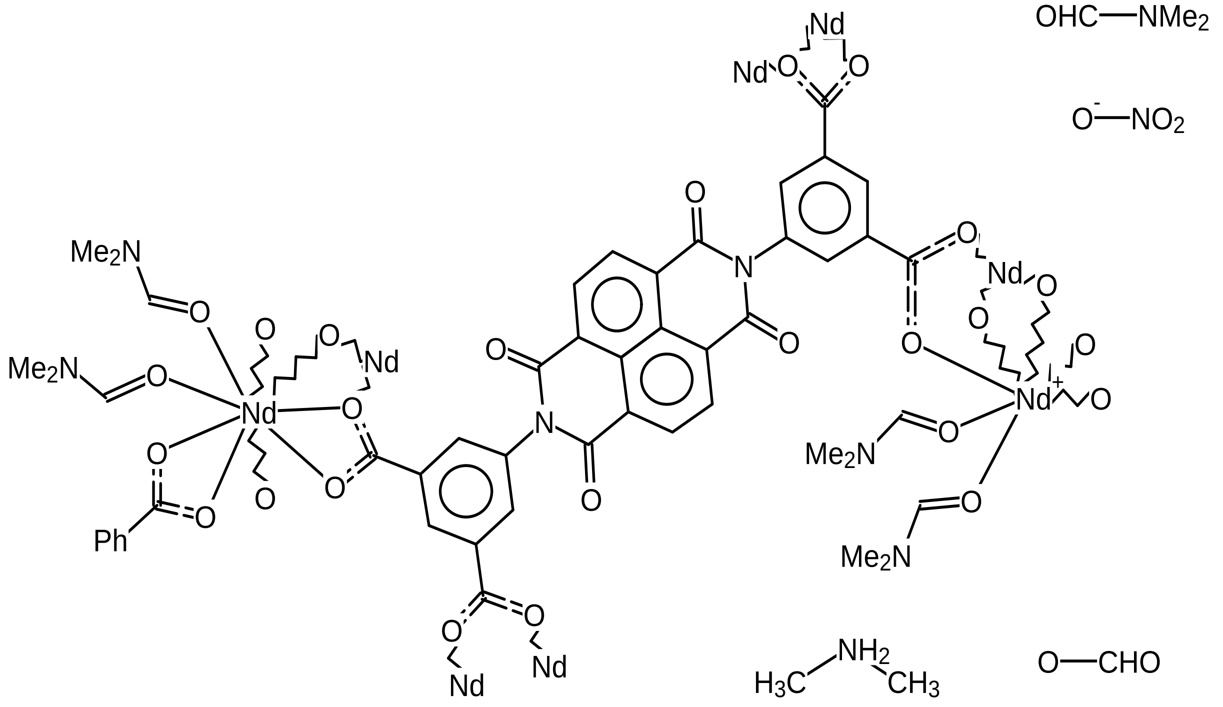

Search: search2 (Mon Jun 30 12:33:47 2025): Hit 65

MAZFOM

Reference:	Seonghun Park, Juhyung Lee, Hwakyeung Jeong, Sangeun Bae, Joongoo Kang, Dohyun Moon, Jinhee Park (2022) *Chem (Cell Press)* ,**8,**1993
Formula:	$(C_{49} H_{43} N_7 Nd_2 O_{21})n,0.92(C_2 H_8 N_1\ ^{1+}),1.6(C_3 H_7 N_1 O_1),0.24(C_1 H_1 O_2\ ^{1-}),0.68(H_1$
Compound Name:	catena-[dimethylammonium (μ-5,5'-(1,3,6,8-tetraoxo-1,3,6,8-tetrahydrobenzo[lmn][3,8]phenanthroline-2,7-diyl)bis(benzene-1,3-dicarboxylato))-(benzoato)-tetrakis(N,N-dimethylformamide)-di-neodymium(iii) formate hydroxide N,N-dimethylformamide solvate]
Synonym:	oξ-150-DGIST-4

Space Group:	C2/c	***Cell:***	***a*** 30.493(6)	***b*** 13.188(3)	***c*** 14.654(3)
Space Group No.:	15	***(Å,°)***	α 90.00	β 93.00(3)	γ 90.00
R-Factor (%):	4.87	***Temperature(K):*** 100	***Density(g/cm³):*** 1.734		

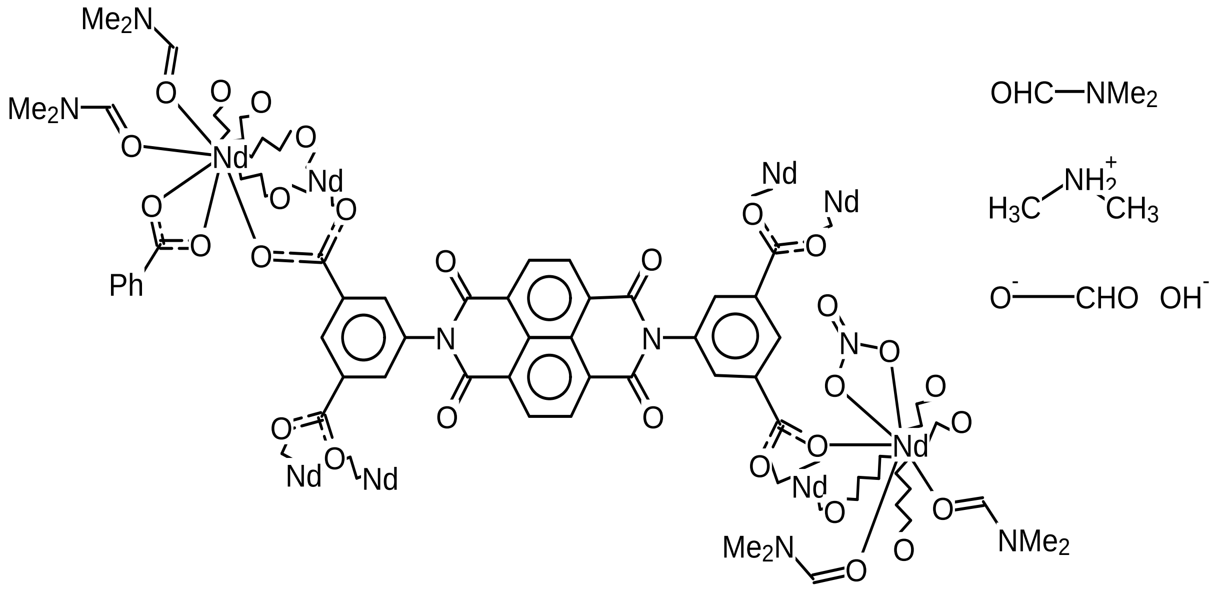

Search: search2 (Mon Jun 30 12:33:47 2025): Hit 66

MAZFUS

Reference:	Seonghun Park, Juhyung Lee, Hwakyeung Jeong, Sangeun Bae, Joongoo Kang, Dohyun Moon, Jinhee Park (2022) *Chem (Cell Press)* ,**8,**1993
Formula:	$(C_{33}\ H_{20}\ N_3\ Nd_1\ O_{14})$n,6.88$(C_3\ H_7\ N_1\ O_1)$,0.63$(C_1\ H_2\ O_2)$,1.38$(H_2\ O_1)$
Compound Name:	catena-[(μ-hydrogen 5,5'-(1,3,6,8-tetraoxo-1,3,6,8-tetrahydrobenzo[lmn][3,8]phenanthroline-2,7-diyl)bis(benzene-1,3-dicarboxylato))-(N,N-dimethylformamide)-aqua-neodymium(iii) formic acid N,N-dimethylformamide solvate hydrate]
Synonym:	85-DGIST-5

Space Group:	C2/c	***Cell:***	***a*** 35.907(4)	***b*** 21.909(2)	***c*** 16.937(1)
Space Group No.:	15	***(Å,°)***	α 90.00	β 92.59(0)	γ 90.00

R-Factor (%):	11.38	***Temperature(K):***	173	***Density(g/cm^3):***	1.381

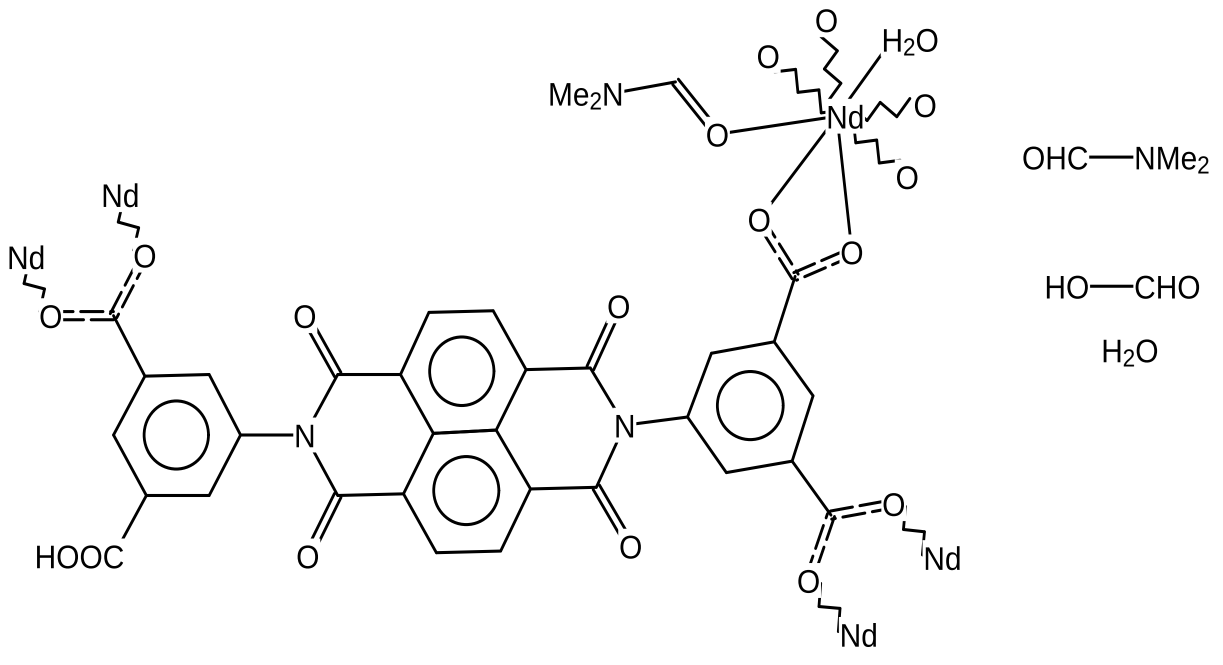

Search: search2 (Mon Jun 30 12:33:47 2025): Hit 67

MIFZEK

Reference:	Sai Ma, Lingling Gao, Yujuan Zhang, Tuoping Hu (2023)<br>*Cryst.Growth Des.* ,**23,**2455						
Formula:	$(C_{58}\ H_{38}\ N_{10}\ O_{12}\ Zn_2)$n,1.5$(C_3\ H_7\ N_1\ O_1)$						
Compound Name:	catena-[(μ-5,5'-(1,3,6,8-tetraoxo-1,3,6,8-tetrahydrobenzo[lmn][3,8]phenanthroline-2,7-diyl)di(benzene-1,3-dicarboxylato))-bis(μ-1,1'-[1,3-phenylenebis(methylene)]di(1H-imidazole))-di-zinc N,N-dimethylformamide solvate]						
Space Group:	C2/c	***Cell:***	***a*** 39.341(8)	***b*** 9.744(2)	***c*** 19.049(4)		
Space Group No.:	15	***(Å,°)***	α 90.00	β 111.42(0)	γ 90.00		
R-Factor (%):	5.52	***Temperature(K):***	273	***Density(g/cm³):***	1.277		

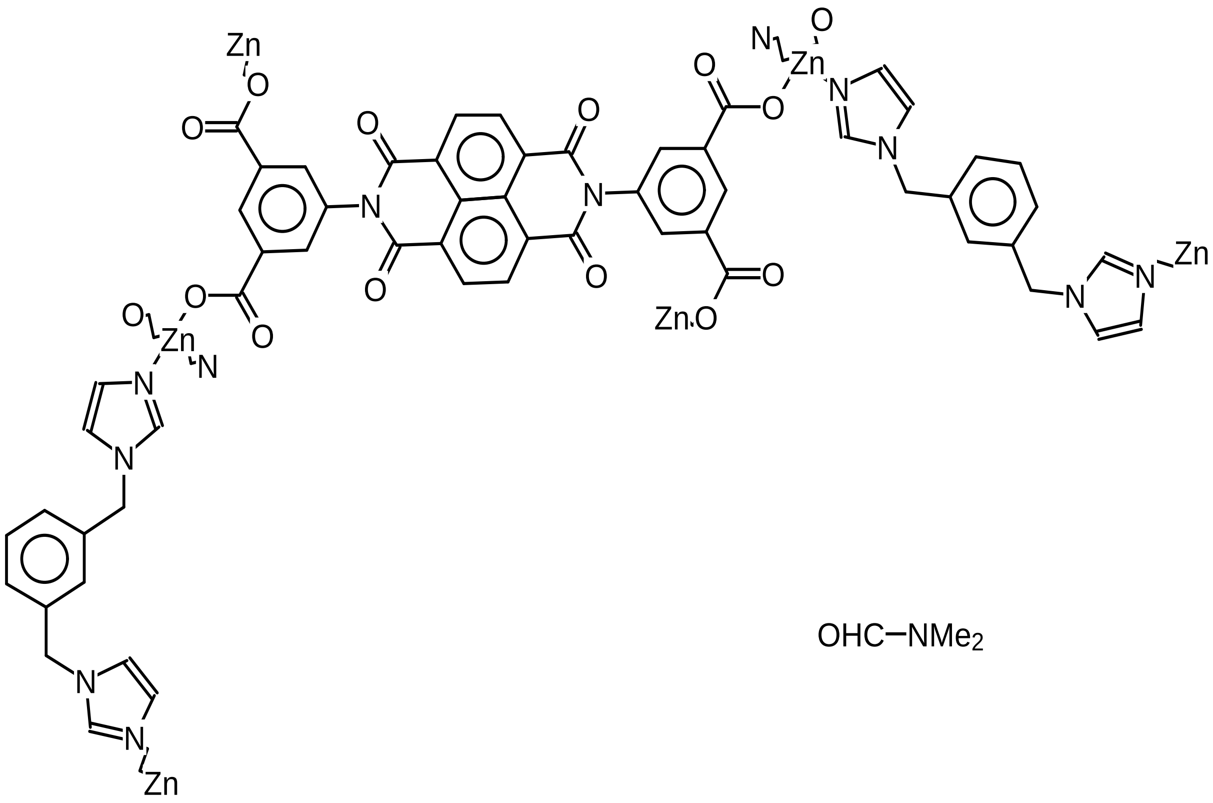

Search: search2 (Mon Jun 30 12:33:47 2025): Hit 68

MIFZIO

Reference: Sai Ma, Lingling Gao, Yujuan Zhang, Tuoping Hu (2023)
Cryst.Growth Des. ,**23,**2455

Formula: $(C_{58}H_{38}N_{10}O_{12}Zn_2)n,C_2H_6O_1,2(C_3H_7N_1O_1),2(H_2O_1)$

Compound Name: catena-[(μ-5,5'-(1,3,6,8-tetraoxo-1,3,6,8-tetrahydrobenzo[lmn][3,8]phenanthroline-2,7-diyl)di(benzene-1,3-dicarboxylato))-bis(μ-1,1'-[1,3-phenylenebis(methylene)]di(1H-imidazole))-di-zinc ethanol N,N-dimethylformamide solvate dihydrate]

Space Group:	P21/c	***Cell:***	***a*** 10.219(4)	***b*** 40.435(18)	***c*** 8.482(4)		
Space Group No.:	14	***(Å,°)***	α 90.00	β 105.51(1)	γ 90.00		

R-Factor (%): 13.16 ***Temperature(K):*** 273 ***Density(g/cm^3):*** 1.402

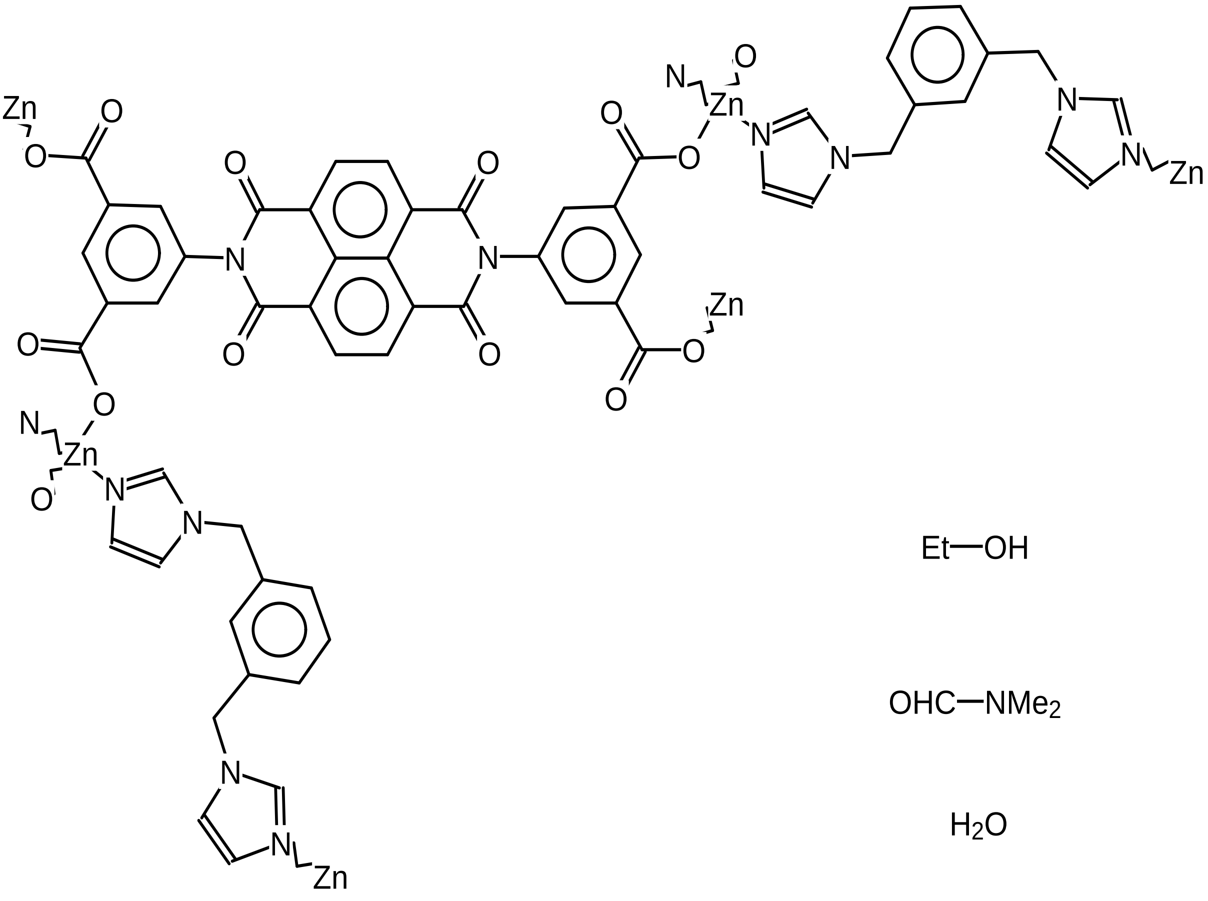

Search: search2 (Mon Jun 30 12:33:47 2025): Hit 69

MIKJEZ

Reference:	Le Zeng, Tiexin Zhang, Renhai Liu, Wenming Tian, Kaifeng Wu, Jingyi Zhu, Zhonghe Wang, Cheng He, Jing Feng, Xiangyang Guo, A.I.Douka, Chunying Duan (2023) *Nat.Commun.* ,**14,** 4002						
Formula:	$(C_{60} H_{20} Cd_1 N_4 O_{24}{}^{4-})n,3(C_3 H_7 N_1 O_1),4(C_2 H_8 N_1{}^{1+}),1.5(H_2 O_1)$						
Compound Name:	catena-[tetrakis(dimethylammonium) bis(μ-5,5'-(1,3,6,8-tetraoxo-1,3,6,8-tetrahydrobenzo[lmn][3,8]phenanthroline-2,7-diyl)bis(benzene-1,3-dicarboxylato))-cadmium N,N-dimethylformamide solvate sesquihydrate]						
Space Group:	C2/c	***Cell:***	***a*** 22.980(0)	***b*** 22.795(1)	***c*** 18.543(0)		
Space Group No.:	15	***(Å,°)***	α 90.00	β 122.78(0)	γ 90.00		
R-Factor (%):	5.30	***Temperature(K):***	293	***Density(g/cm³):***	1.402		

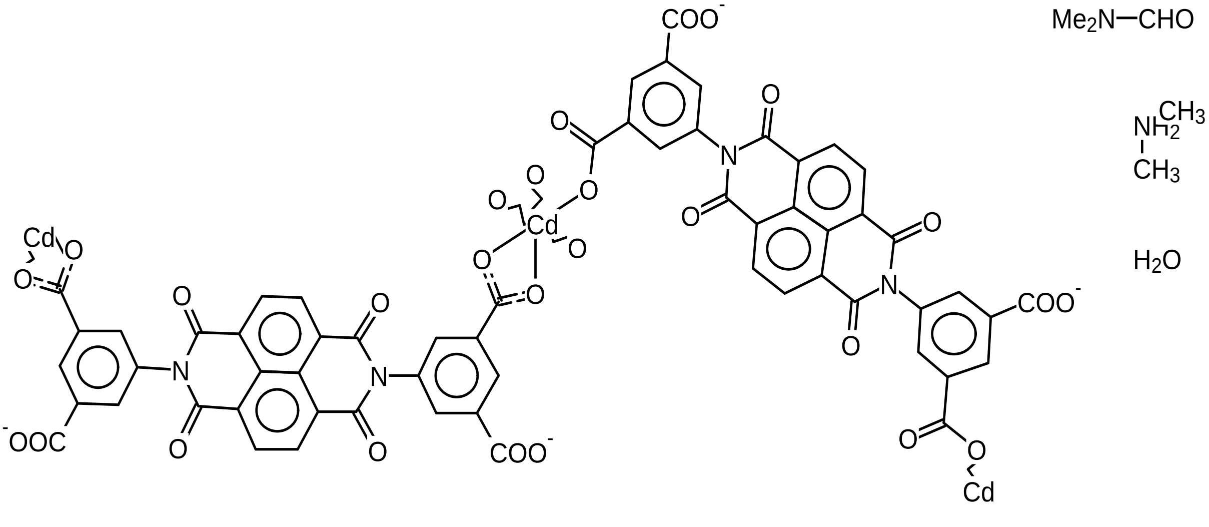

Search: search2 (Mon Jun 30 12:33:47 2025): Hit 70

MIKKEA

Reference: Le Zeng, Tiexin Zhang, Renhai Liu, Wenming Tian, Kaifeng Wu, Jingyi Zhu, Zhonghe Wang, Cheng He, Jing Feng, Xiangyang Guo, A.I.Douka, Chunying Duan (2023) *Nat.Commun.* ,**14,** 4002

Formula: $(C_{42} H_{38} N_6 O_{16} Sr_2)n,C_{16} H_{10}$

Compound Name: catena-[(μ-5,5'-(1,3,6,8-tetraoxo-1,3,6,8-tetrahydrobenzo[lmn][3,8]phenanthroline-2,7-diyl)bis(benzene-1,3-dicarboxylato))-tetrakis(N,N-dimethylformamide)-di-strontium(ii) pyrene]

Space Group:	I41/a	***Cell:***	***a***	28.325(1)	***b***	28.325(1)	***c***	13.777(0)
Space Group No.:	88	***(Å,°)***	α	90.00	β	90.00	γ	90.00

R-Factor (%): 2.62 ***Temperature(K):*** 150 ***Density(g/cm³):*** 1.515

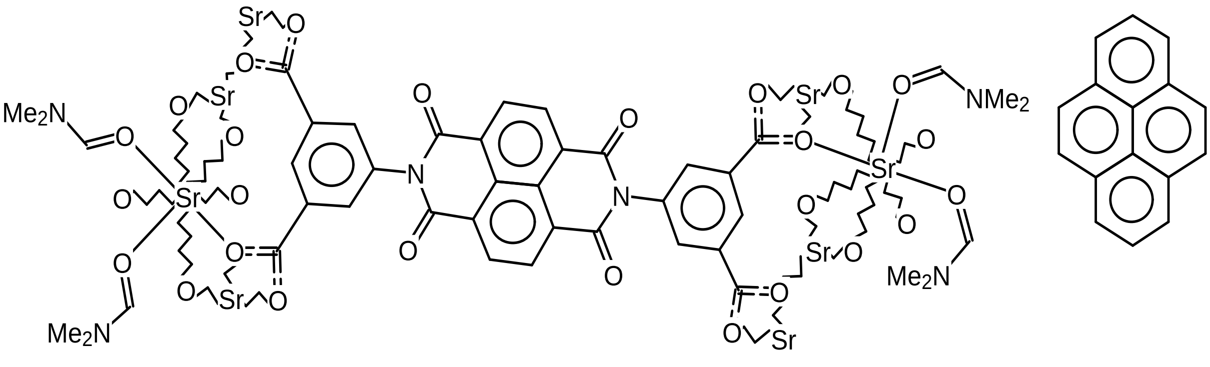

Search: search2 (Mon Jun 30 12:33:47 2025): Hit 71

NANTII

Reference: Hai-Long Zhang, Jian-Zhen Liao, Wenbin Yang, Xiao-Yuan Wu, Can-Zhong Lu (2017) *Dalton Trans.* ,**46,**4898

Formula: $(C_{62} H_{82} Ce_2 N_{10} O_{20}{}^{2+})2n,n(O_{40} Si_1 W_{12}{}^{4-}),3n(C_4 H_9 N_1 O_1)$

Compound Name: catena-[bis((μ-5,5'-(1,3,6,8-tetraoxo-1,3,6,8-tetrahydrobenzo[lmn][3,8]phenanthroline-2,7-diyl)di(benzene-1,3-dicarboxylato))-octakis(N,N-dimethylacetamide)-di-cerium(iii)) (μ-orthosilicato)-tetracosakis(μ-oxo)-dodecqoxo-dodeca-tungsten N,N-dimethylacetamide solvate]

Synonym: LONF-1

Space Group:	P42/n	***Cell:***	***a***	23.714(3)	***b***	23.714(3)	***c***	16.575(3)
Space Group No.:	86	***(Å,°)***	α	90.00	β	90.00	γ	90.00

R-Factor (%): 7.61 ***Temperature(K):*** 123 ***Density(g/cm³):*** 2.234

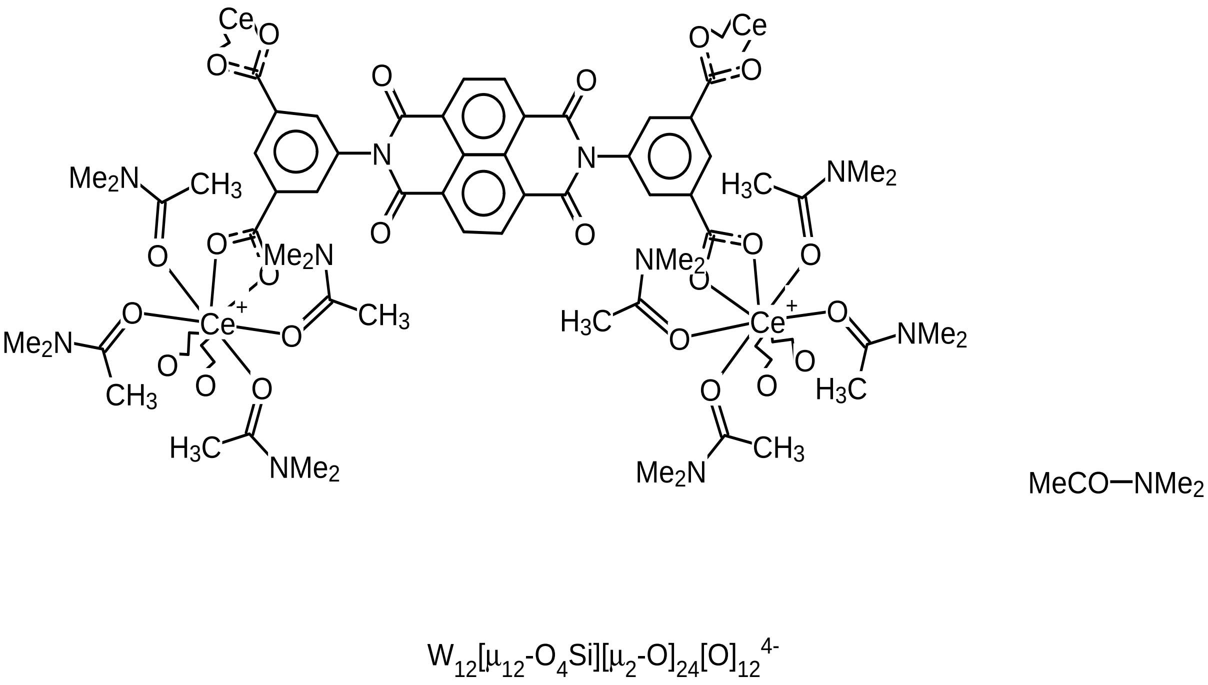

Search: search2 (Mon Jun 30 12:33:47 2025): Hit 72

NOWKIW

Reference:	G.H.Dang, Y.B.N.Tran, T.V.Pham, Vu T.Pham, N.T.H.Luu, H.D.Nguyen, P.T.K.Nguyen, H.T.D.Nguyen, Thanh Truong (2019) *ChemPlusChem* ,**84,**1046
Formula:	$(C_{30} H_{17} Ce_1 N_2 O_{15})n$
Compound Name:	catena-[(μ-hydrogen 5,5'-(1,3,6,8-tetraoxo-1,3,6,8-tetrahydrobenzo[lmn][3,8]phenanthroline-2,7-diyl)di(benzene-1,3-dicarboxylato))-triaqua-cerium unknown solvate]

Space Group:	P-1	***Cell:***	***a*** 8.426(0)	***b*** 11.430(0)	***c*** 17.992(0)
Space Group No.:	2	***(Å,°)***	α 93.35(0)	β 95.93(0)	γ 107.91(0)
R-Factor (%):	3.26	***Temperature(K):*** 296	***Density(g/cm³):*** 1.598		

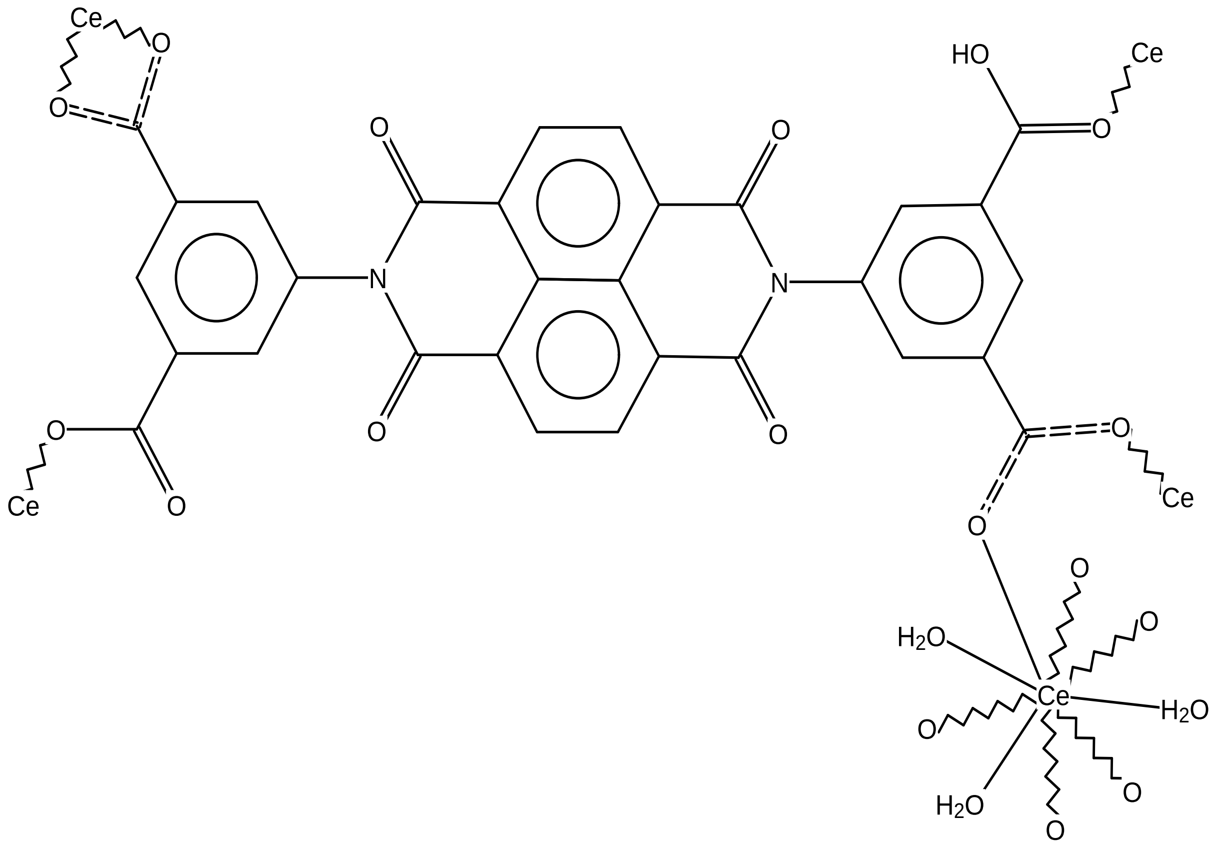

Search: search2 (Mon Jun 30 12:33:47 2025): Hit 73

OFOTAI

Reference:	Wen-Bin Li, Ying Wu, Xiao-Feng Zhong, Xiong-Hai Chen, Gang Liang, Jia-Wen Ye, Zong-Wen Mo, Xiao-Ming Chen (2023) *Angew.Chem.,Int.Ed.* ,**62,**e2023035
Formula:	$(C_{33}\ H_{19}\ N_3\ O_{14}\ Sr_2)n,0.8(C_3\ H_7\ N_1\ O_1)$
Compound Name:	catena-[(μ-5,5'-(1,3,6,8-tetraoxo-1,3,6,8-tetrahydrobenzo[lmn][3,8]phenanthroline-2,7-diyl)bis(benzene-1,3-dicarboxylato))-(N,N-dimethylformamide)-aqua-di-strontium(ii) N,N-dimethylformamide solvate]
Synonym:	WYU-61

Space Group:	P-1	***Cell:***	***a*** 8.057(0)	***b*** 8.485(0)	***c*** 13.619(0)
Space Group No.:	2	***(Å,°)***	α 80.83(0)	β 74.42(0)	γ 76.89(0)

R-Factor (%): 3.64 ***Temperature(K):*** 298 ***Density(g/cm^3):*** 1.750

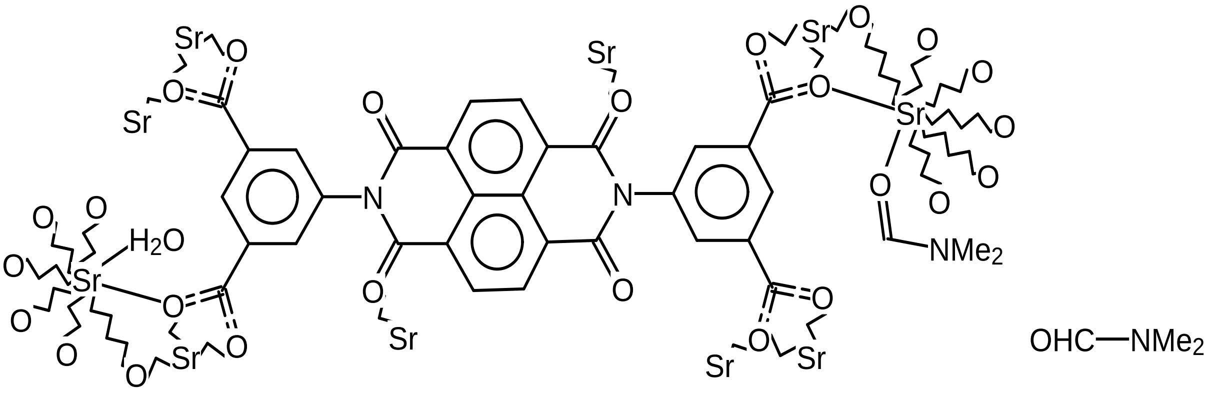

Search: search2 (Mon Jun 30 12:33:47 2025): Hit 74

OFOTEM

Reference:	Wen-Bin Li, Ying Wu, Xiao-Feng Zhong, Xiong-Hai Chen, Gang Liang, Jia-Wen Ye, Zong-Wen Mo, Xiao-Ming Chen (2023) *Angew.Chem.,Int.Ed.* ,**62,**e2023035
Formula:	$(C_{30} H_{14} N_2 O_{14} Sr_2)n,7(H_2 O_1)$
Compound Name:	catena-[(μ-5,5'-(1,3,6,8-tetraoxo-1,3,6,8-tetrahydrobenzo[lmn][3,8]phenanthroline-2,7-diyl)bis(benzene-1,3-dicarboxylato))-diaqua-di-strontium(ii) heptahydrate]
Synonym:	WYU-62

Space Group:	P-1	***Cell:***	***a*** 8.270(0)	***b*** 8.300(0)	***c*** 13.465(0)
Space Group No.:	2	***(Å,°)***	α 79.50(0)	β 74.69(0)	γ 78.18(0)

R-Factor (%):	4.02	***Temperature(K):***	296	***Density(g/cm³):***	1.782

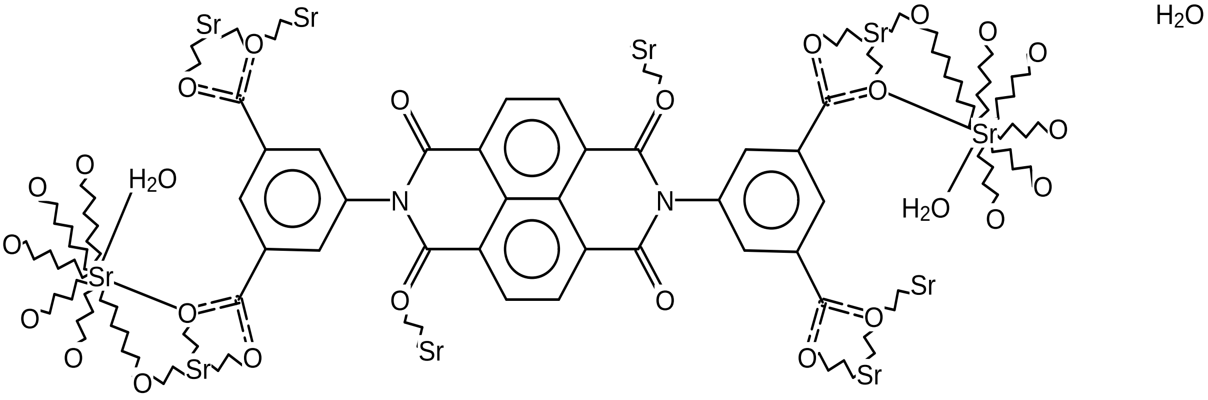

Search: search2 (Mon Jun 30 12:33:47 2025): Hit 75

OFOTIQ

Reference:	Wen-Bin Li, Ying Wu, Xiao-Feng Zhong, Xiong-Hai Chen, Gang Liang, Jia-Wen Ye, Zong-Wen Mo, Xiao-Ming Chen (2023) *Angew.Chem.,Int.Ed.* ,**62,**e2023035
Formula:	$(C_{30} H_{14} N_2 O_{14} Sr_2)n,C_6 H_6,H_2 O_1$
Compound Name:	catena-[(μ-5,5'-(1,3,6,8-tetraoxo-1,3,6,8-tetrahydrobenzo[lmn][3,8]phenanthroline-2,7-diyl)bis(benzene-1,3-dicarboxylato))-diaqua-di-strontium(ii) benzene solvate monohydrate]
Synonym:	WYU-61a benzene solvate

Space Group:	P-1	***Cell:***	***a*** 8.074(0)	***b*** 8.308(0)	***c*** 13.707(1)
Space Group No.:	2	***(Å,°)***	α 82.70(0)	β 74.39(0)	γ 77.58(0)

R-Factor (%):	8.87	***Temperature(K):***	150	***Density(g/cm³):***	1.728

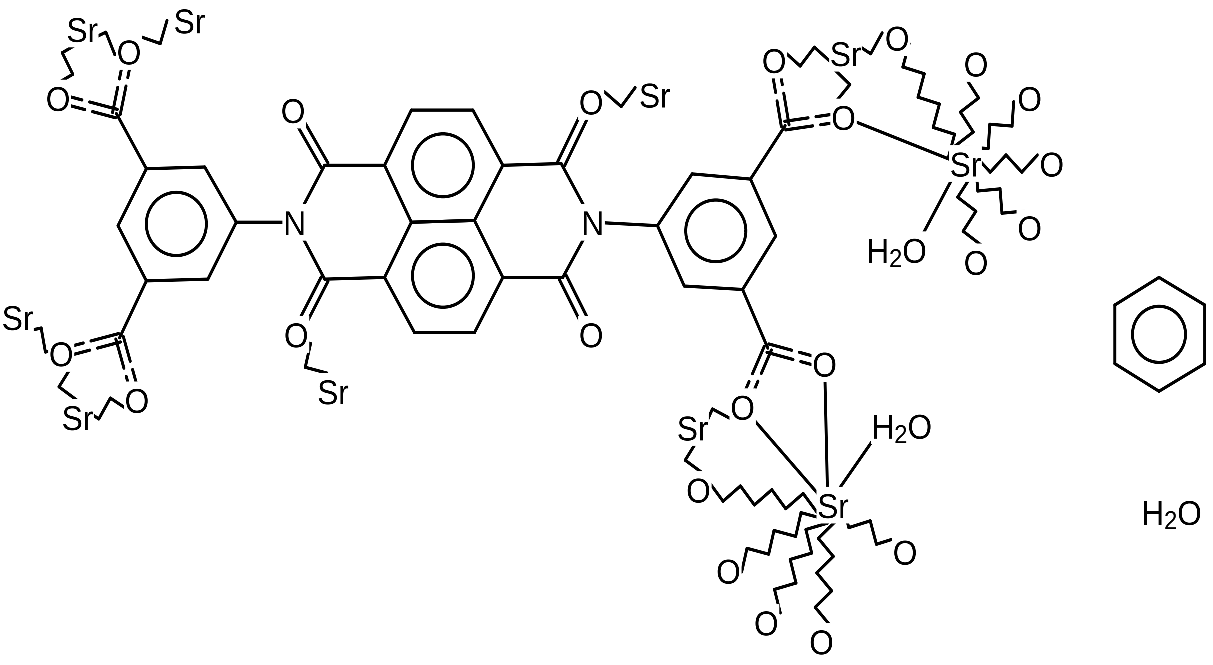

Search: search2 (Mon Jun 30 12:33:47 2025): Hit 76

OFOTOW

Reference:	Wen-Bin Li, Ying Wu, Xiao-Feng Zhong, Xiong-Hai Chen, Gang Liang, Jia-Wen Ye, Zong-Wen Mo, Xiao-Ming Chen (2023) *Angew.Chem.,Int.Ed.* ,**62,**e2023035
Formula:	$(C_{30} H_{14} N_2 O_{14} Sr_2)n,C_7 H_8$
Compound Name:	catena-[(μ-5,5'-(1,3,6,8-tetraoxo-1,3,6,8-tetrahydrobenzo[lmn][3,8]phenanthroline-2,7-diyl)bis(benzene-1,3-dicarboxylato))-diaqua-di-strontium(ii) toluene solvate]
Synonym:	WYU-61a toluene solvate

Space Group:	P-1	***Cell:***	***a*** 8.068(0)	***b*** 8.167(0)	***c*** 13.924(0)
Space Group No.:	2	***(Å,°)***	α 88.36(0)	β 73.27(0)	γ 78.40(0)

R-Factor (%):	5.08	***Temperature(K):***	298	***Density(g/cm³):***	1.725

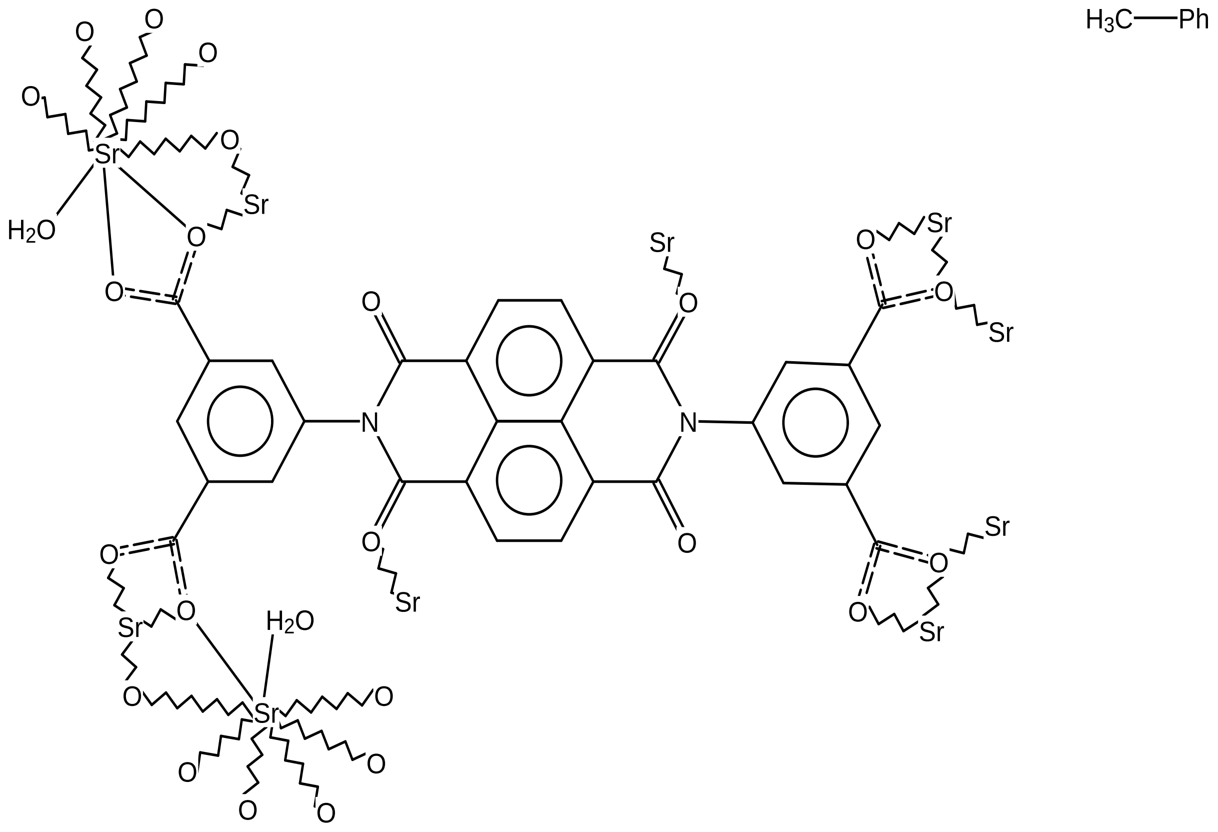

Search: search2 (Mon Jun 30 12:33:47 2025): Hit 77

OFOTUC

Reference: Wen-Bin Li, Ying Wu, Xiao-Feng Zhong, Xiong-Hai Chen, Gang Liang, Jia-Wen Ye, Zong-Wen Mo, Xiao-Ming Chen (2023) *Angew.Chem.,Int.Ed.* ,**62,**e2023035

Formula: $(C_{30} H_{14} N_2 O_{14} Sr_2)n,C_8 H_{10}$

Compound Name: catena-[(μ-5,5'-(1,3,6,8-tetraoxo-1,3,6,8-tetrahydrobenzo[lmn][3,8]phenanthroline-2,7-diyl)bis(benzene-1,3-dicarboxylato))-diaqua-di-strontium(ii) m-xylene solvate]

Synonym: WYU-61a m-xylene solvate

Space Group:	P-1	***Cell:***	***a***	8.049(0)	***b***	8.184(0)	***c***	13.973(0)
Space Group No.:	2	***(Å,°)***	α	88.66(0)	β	74.33(0)	γ	78.88(0)

R-Factor (%): 5.66 ***Temperature(K):*** 259 ***Density(g/cm³):*** 1.734

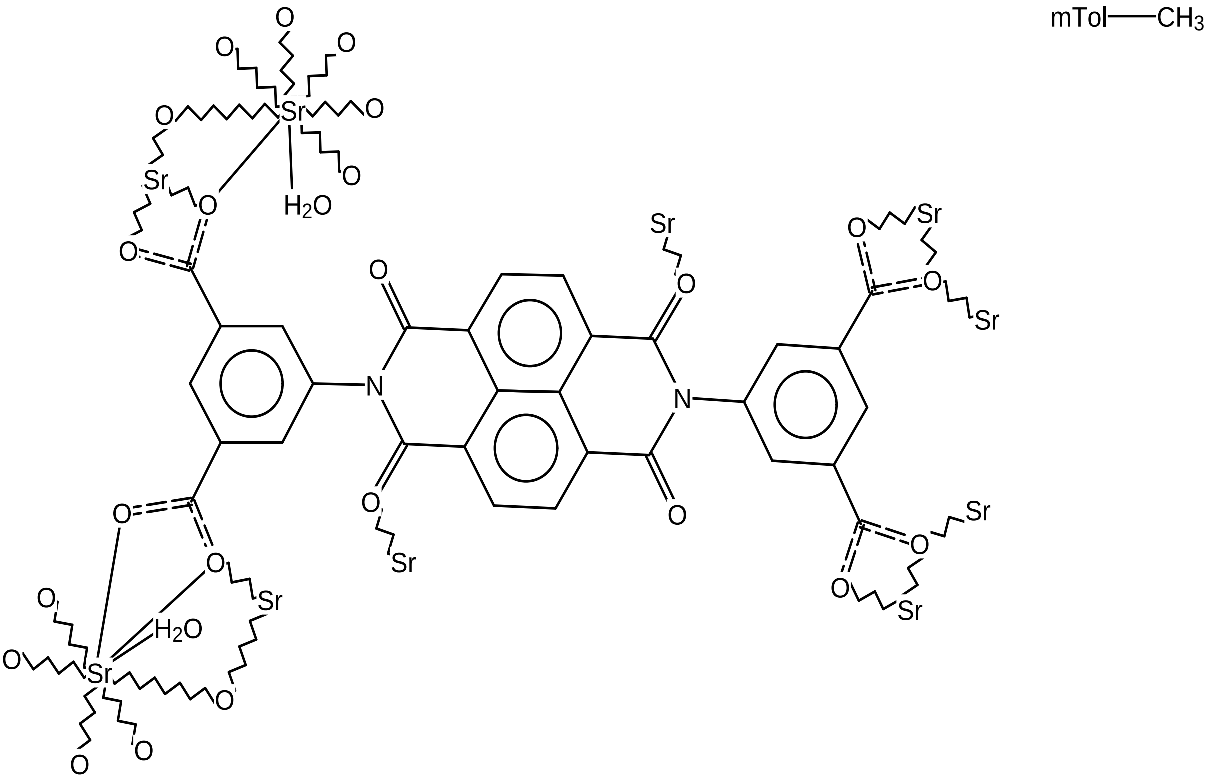

Search: search2 (Mon Jun 30 12:33:47 2025): Hit 78

OFOVAK

Reference:	Wen-Bin Li, Ying Wu, Xiao-Feng Zhong, Xiong-Hai Chen, Gang Liang, Jia-Wen Ye, Zong-Wen Mo, Xiao-Ming Chen (2023) *Angew.Chem.,Int.Ed.* ,**62,**e2023035
Formula:	$(C_{30} H_{14} N_2 O_{14} Sr_2)n,C_8 H_{10}$
Compound Name:	catena-[(μ-5,5'-(1,3,6,8-tetraoxo-1,3,6,8-tetrahydrobenzo[lmn][3,8]phenanthroline-2,7-diyl)bis(benzene-1,3-dicarboxylato))-diaqua-di-strontium(ii) p-xylene solvate]
Synonym:	WYU-61a p-xylene solvate

Space Group:	P-1	***Cell:***	***a*** 8.057(0)	***b*** 8.229(0)	***c*** 13.913(0)		
Space Group No.:	2	***(Å,°)***	α 88.59(0)	β 73.66(0)	γ 78.20(0)		
R-Factor (%):	7.34	***Temperature(K):***	296	***Density(g/cm³):***	1.741		

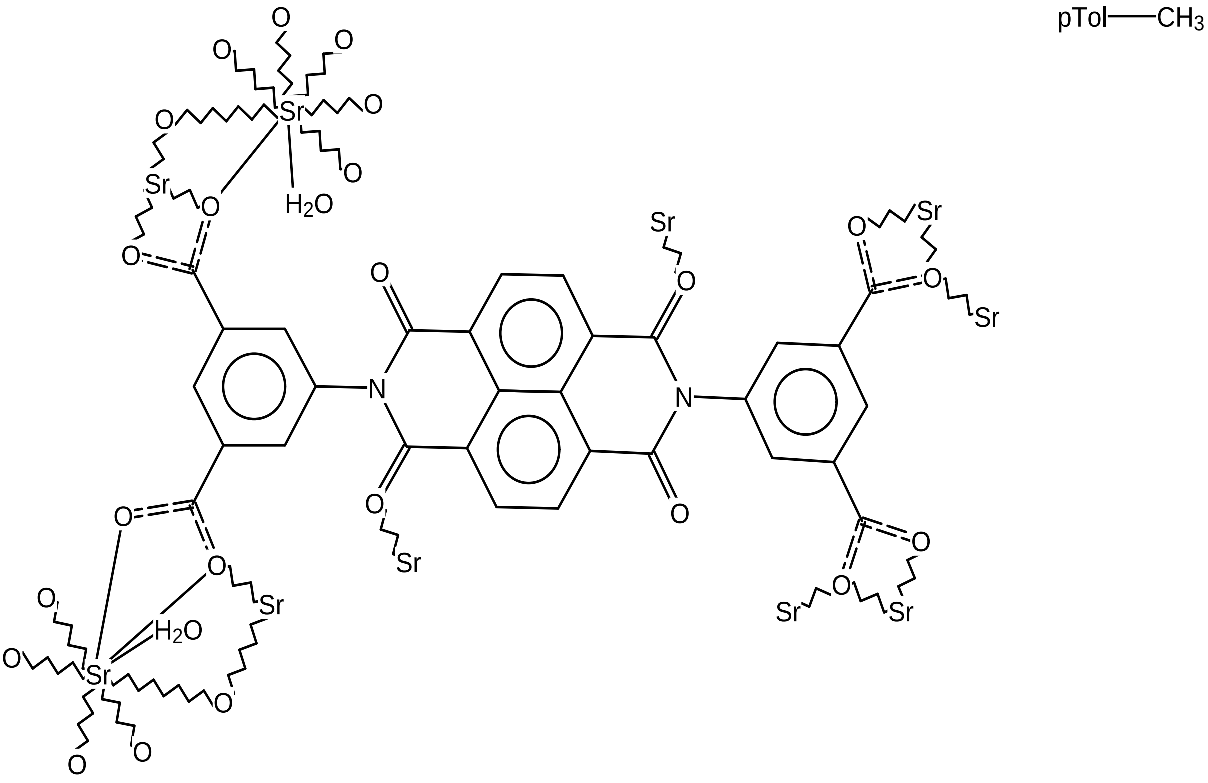

Search: search2 (Mon Jun 30 12:33:47 2025): Hit 79

OFOVEO

Reference:	Wen-Bin Li, Ying Wu, Xiao-Feng Zhong, Xiong-Hai Chen, Gang Liang, Jia-Wen Ye, Zong-Wen Mo, Xiao-Ming Chen (2023) *Angew.Chem.,Int.Ed.* ,**62,**e2023035
Formula:	$(C_{30}\,H_{14}\,N_2\,O_{14}\,Sr_2)n,C_8\,H_{10}$
Compound Name:	catena-[(μ-5,5'-(1,3,6,8-tetraoxo-1,3,6,8-tetrahydrobenzo[lmn][3,8]phenanthroline-2,7-diyl)bis(benzene-1,3-dicarboxylato))-diaqua-di-strontium(ii) o-xylene solvate]
Synonym:	WYU-61a o-xylene solvate

Space Group:	P-1	***Cell:***	***a*** 8.043(0)	***b*** 8.267(0)	***c*** 13.961(0)		
Space Group No.:	2	***(Å,°)***	α 89.28(0)	β 73.67(0)	γ 78.31(0)		
R-Factor (%):	7.91	***Temperature(K):***	297	***Density(g/cm3):***	1.730		

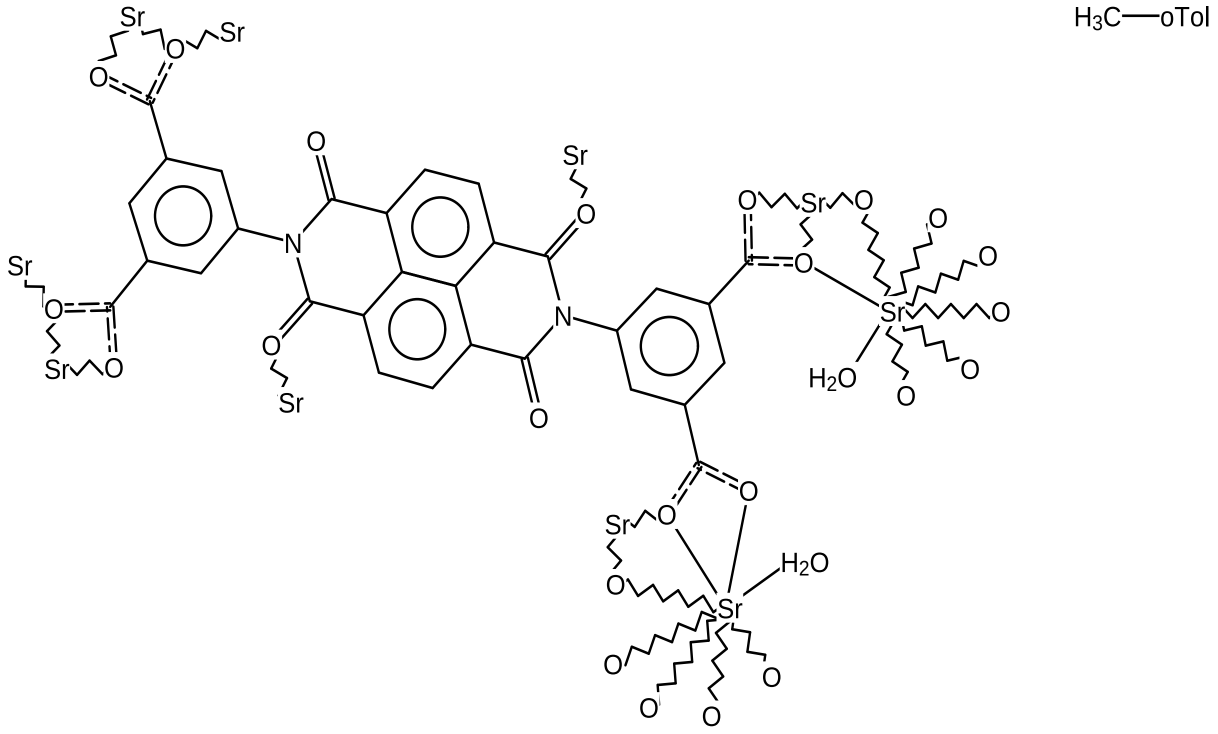

Search: search2 (Mon Jun 30 12:33:47 2025): Hit 80

PUQPID

Reference:	Haonan Wu, Lingling Gao, Jie Zhang, Lijun Zhai, Ting Gao, Xiaoyan Niu, Tuoping Hu (2020) *J.Mol.Struct.* ,**1219,**128613
Formula:	$(C_{82}\ H_{46}\ Co_2\ N_{10}\ O_{12})n$
Compound Name:	catena-[(μ-5,5'-(1,3,6,8-tetraoxo-1,3,6,8-tetrahydrobenzo[lmn][3,8]phenanthroline-2,7-diyl)bis(benzene-1,3-dicarboxylato))-bis(μ-1,1'-([1,1'-biphenyl]-4,4'-diyl)bis(1H-benzimidazole))-di-cobalt(ii) unknown solvate]

Space Group:	P21/c	***Cell:***	***a*** 10.260(1)	***b*** 15.415(1)	***c*** 23.582(0)
Space Group No.:	14	***(Å,°)***	α 90.00	β 81.86(0)	γ 90.00
R-Factor (%):	8.87	***Temperature(K):*** 141	***Density(g/cm³):*** 1.332		

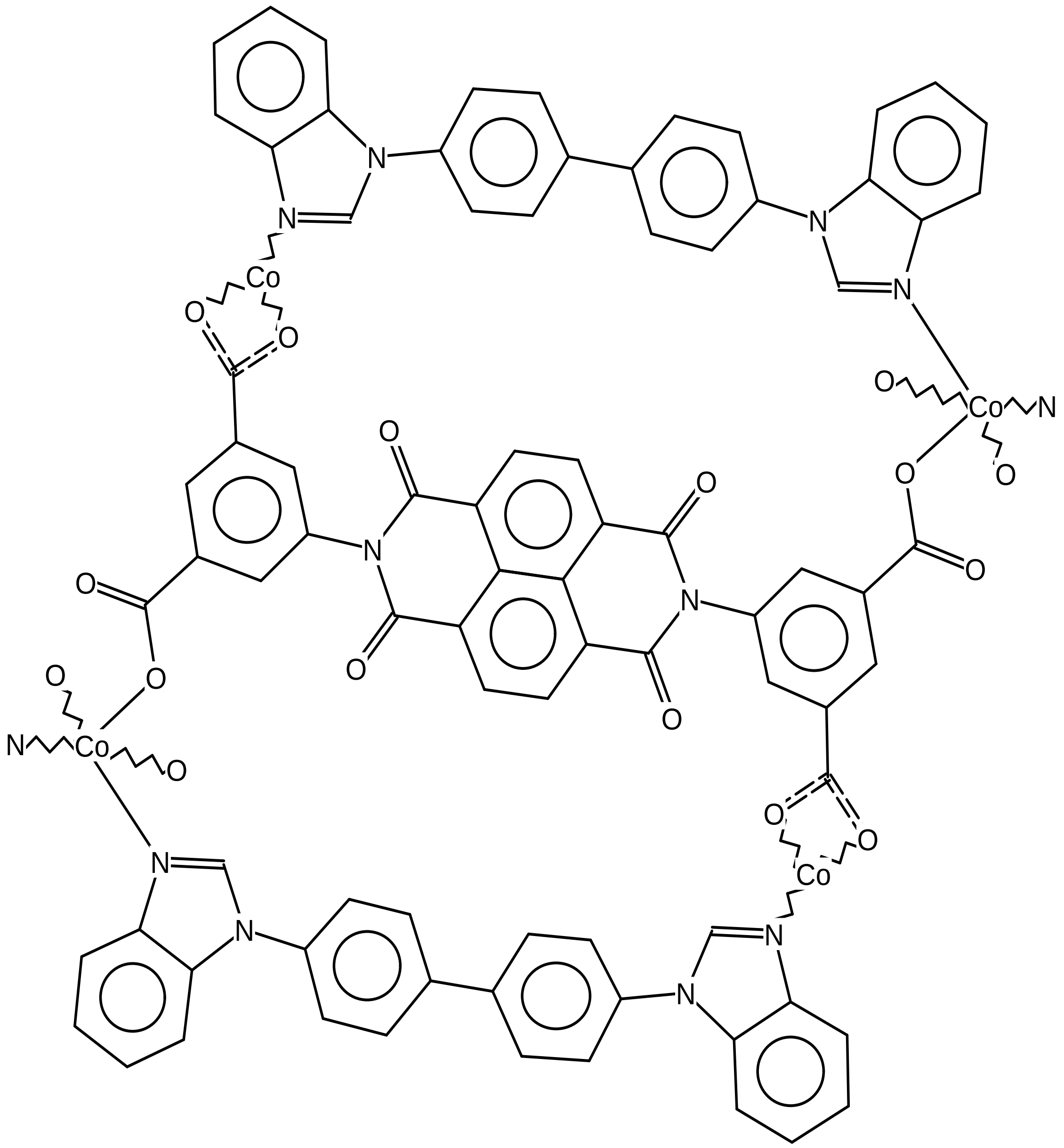

Search: search2 (Mon Jun 30 12:33:47 2025): Hit 81

PUQPOJ

Reference:	Haonan Wu, Lingling Gao, Jie Zhang, Lijun Zhai, Ting Gao, Xiaoyan Niu, Tuoping Hu (2020) *J.Mol.Struct.* ,**1219,**128613
Formula:	$(C_{82} H_{46} Cd_2 N_{10} O_{12})n,4(H_2 O_1)$
Compound Name:	catena-[(μ-5,5'-(1,3,6,8-tetraoxo-1,3,6,8-tetrahydrobenzo[lmn][3,8]phenanthroline-2,7-diyl)bis(benzene-1,3-dicarboxylato))-bis(μ-1,1'-([1,1'-biphenyl]-4,4'-diyl)bis(1H-benzimidazole))-di-cadmium(ii) tetrahydrate]

Space Group:	P21/c	***Cell:***	***a*** 10.567(0)	***b*** 15.126(1)	***c*** 24.276(1)		
Space Group No.:	14	***(Å,°)***	α 90.00	β 98.27(0)	γ 90.00		
R-Factor (%):	12.06	***Temperature(K):***	150	***Density(g/cm³):***	1.436		

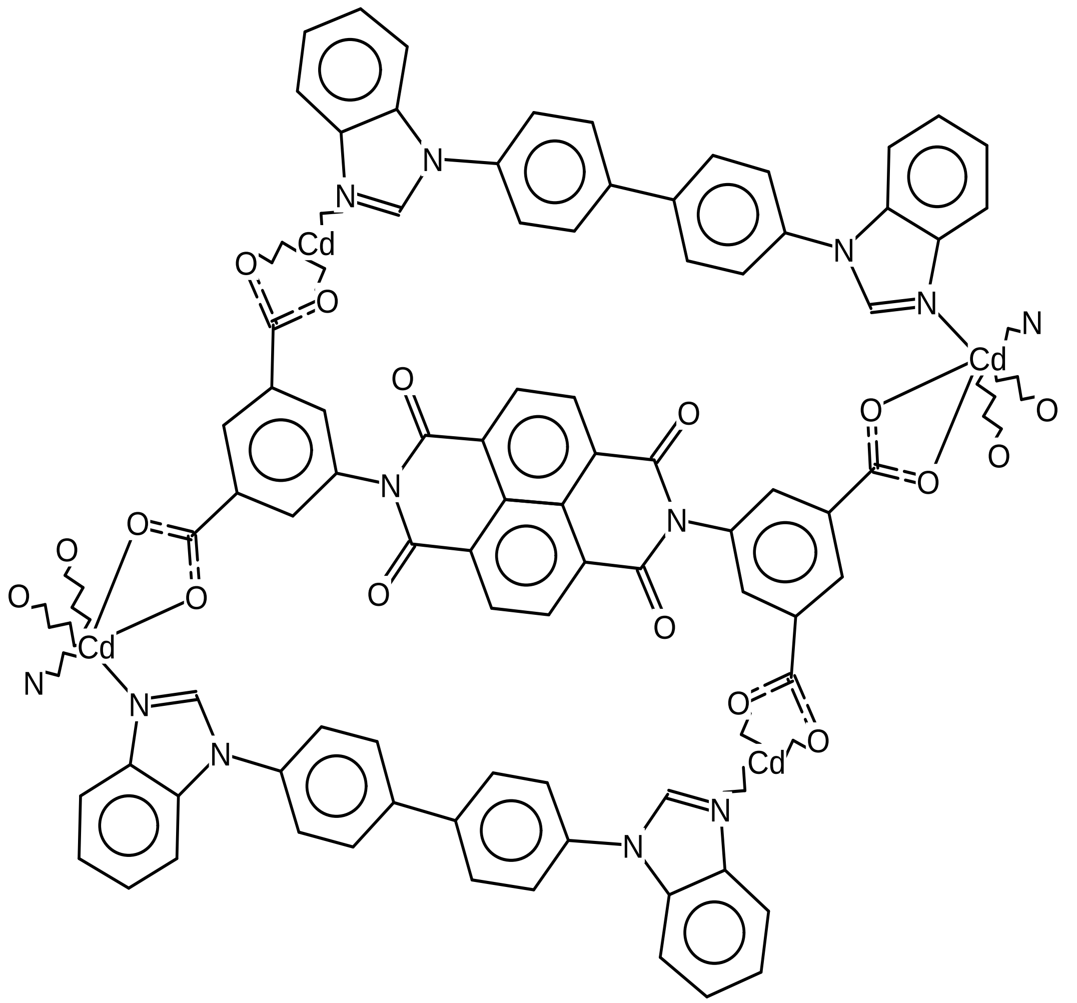

H_2O

Search: search2 (Mon Jun 30 12:33:47 2025): Hit 82

PUQPUP

Reference: Haonan Wu, Lingling Gao, Jie Zhang, Lijun Zhai, Ting Gao, Xiaoyan Niu, Tuoping Hu (2020) *J.Mol.Struct.* ,**1219,**128613

Formula: $(C_{82} H_{46} N_{10} O_{12} Zn_2)$n,3($C_3 H_7 N_1 O_1$),3($H_2 O_1$)

Compound Name: catena-[(μ-5,5'-(1,3,6,8-tetraoxo-1,3,6,8-tetrahydrobenzo[lmn][3,8]phenanthroline-2,7-diyl)bis(benzene-1,3-dicarboxylato))-bis(μ-1,1'-([1,1'-biphenyl]-4,4'-diyl)bis(1H-benzimidazole))-di-zinc(ii) N,N-dimethylformamide solvate trihydrate]

Space Group:	P21/c	***Cell:***	***a***	10.417(2)	***b***	15.712(3)	***c***	23.359(5)
Space Group No.:	14	***(Å,°)***	α	90.00	β	98.64(3)	γ	90.00

R-Factor (%): 5.35 ***Temperature(K):*** 293 ***Density(g/cm³):*** 1.553

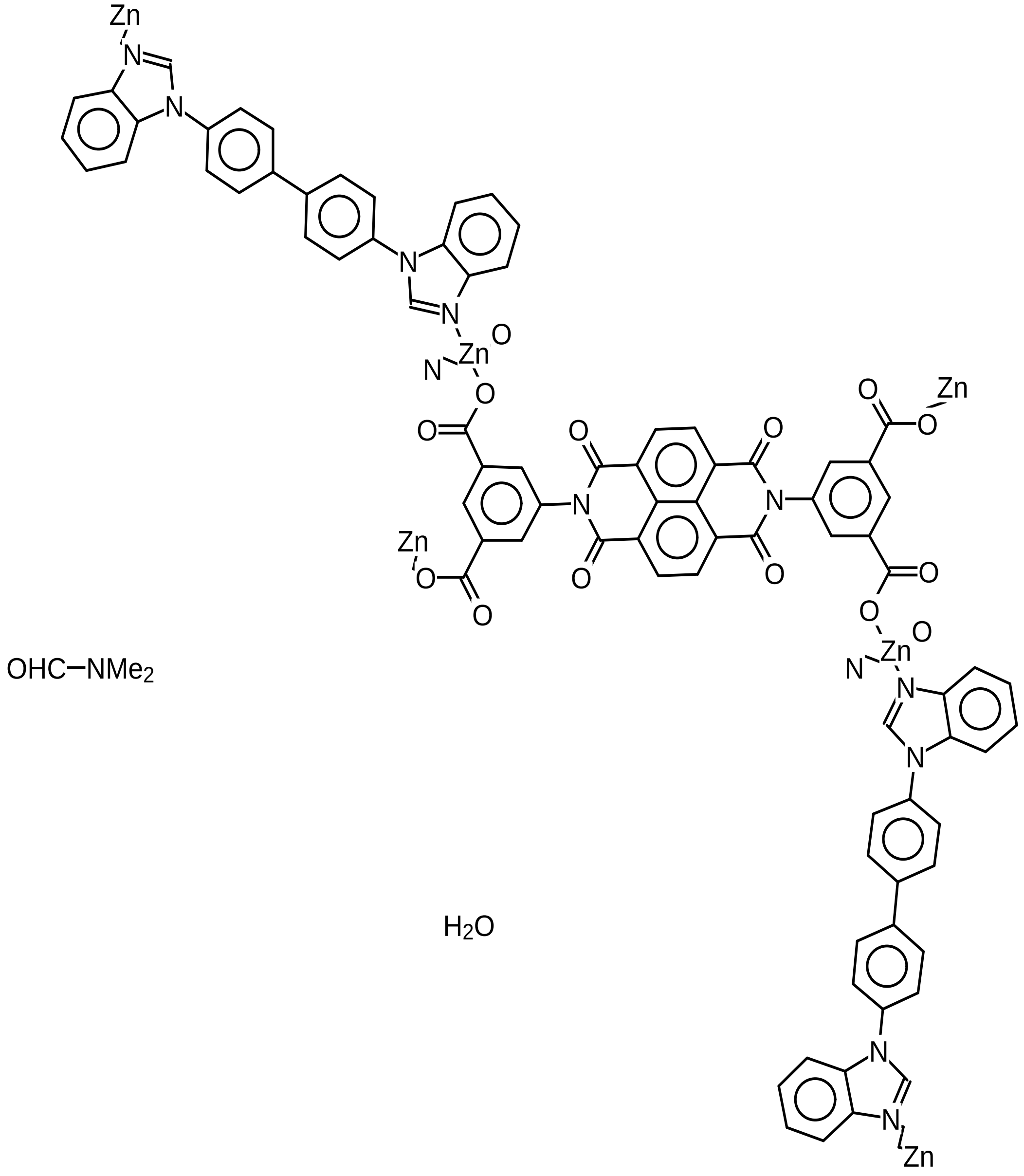

Search: search2 (Mon Jun 30 12:33:47 2025): Hit 83

PUYGUO

Reference:	Xiaokang Wang (2020)<br>*CSD Communication(Private Communication) ,*
Formula:	$(C_{33} H_{21} Ba_2 N_3 O_{16})n,C_3 H_7 N_1 O_1$
Compound Name:	catena-[(μ-5,5'-(1,3,6,8-tetraoxo-1,3,6,8-tetrahydrobenzo[lmn][3,8]phenanthroline-2,7-diyl)bis(benzene-1,3-dicarboxylato))-(μ-aqua)-(N,N-dimethylformamide)-aqua-di-barium(ii) N,N-dimethylformamide unknown solvate]

Space Group:	P21212	***Cell:***	***a***	32.748(0)	***b***	14.076(0)	***c***	12.376(0)
Space Group No.:	18	***(Å,°)***	α	90.00	β	90.00	γ	90.00

R-Factor (%):	6.65	***Temperature(K):***	150	***Density(g/cm³):***	1.238

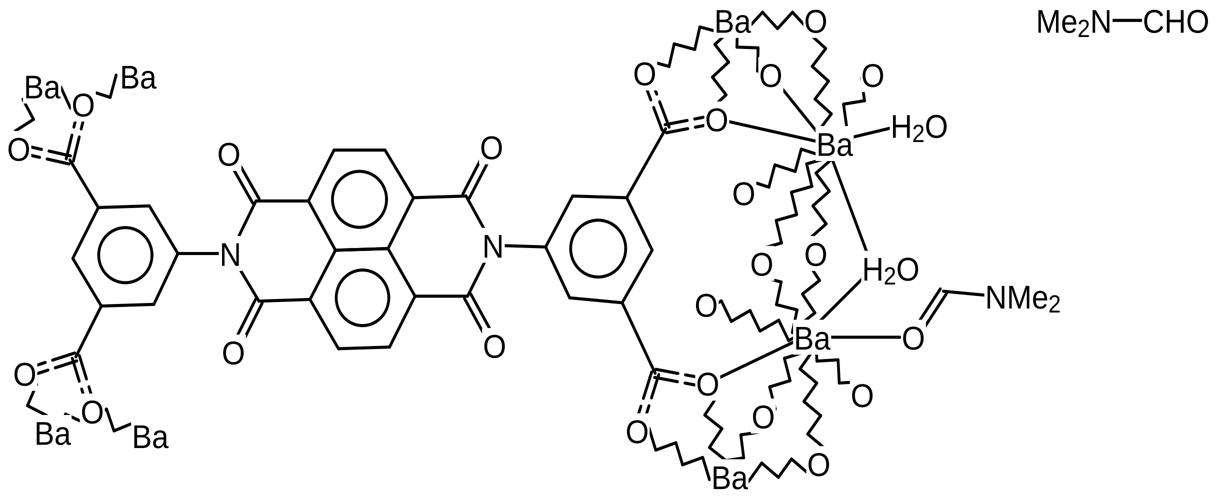

Search: search2 (Mon Jun 30 12:33:47 2025): Hit 84

PUYGUO01

Reference: Fangna Dai, Xiaokang Wang, Yutong Wang, Zhanning Liu, Daofeng Sun (2020) *Angew.Chem.,Int.Ed.* ,**59,**22372

Formula: $(C_{33} H_{21} Ba_2 N_3 O_{16})n,C_3 H_7 N_1 O_1$

Compound Name: catena-[(μ-5,5'-(1,3,6,8-tetraoxo-1,3,6,8-tetrahydrobenzo[lmn][3,8]phenanthroline-2,7-diyl)bis(benzene-1,3-dicarboxylato))-(μ-aqua)-(N,N-dimethylformamide)-aqua-di-barium(ii) N,N-dimethylformamide unknown solvate]

Space Group:	P21212	***Cell:***	***a***	32.748(0)	***b***	14.076(0)	***c***	12.376(0)
Space Group No.:	18	***(Å,°)***	α	90.00	β	90.00	γ	90.00

R-Factor (%): 6.65 ***Temperature(K):*** 150 ***Density(g/cm³):*** 1.238

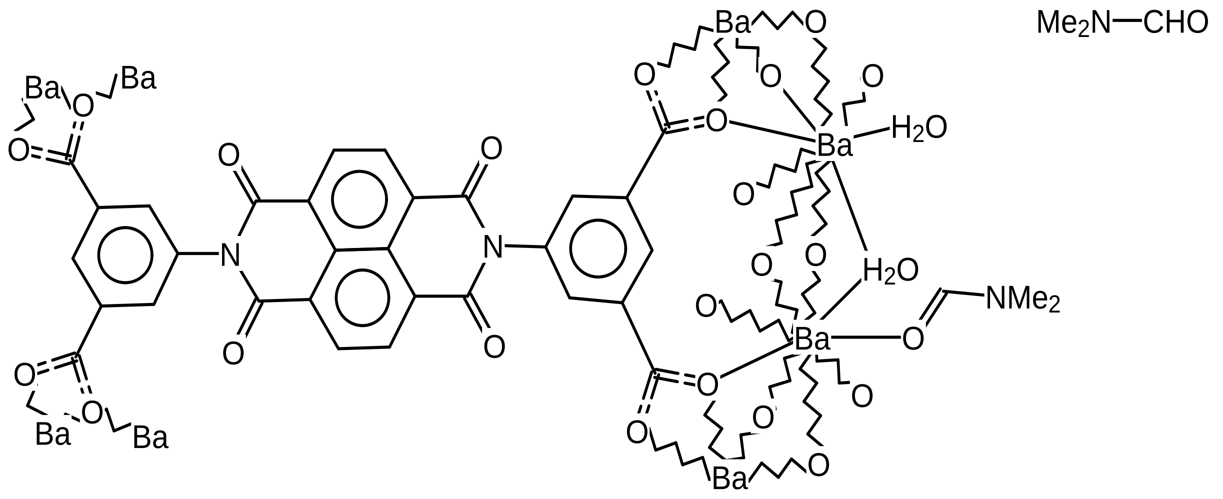

Search: search2 (Mon Jun 30 12:33:47 2025): Hit 85

PUYHAV

Reference:	Xiaokang Wang (2020) *CSD Communication(Private Communication) ,*						
Formula:	$(C_{30} H_{18} Ba_2 N_2 O_{16})n$						
Compound Name:	catena-[(μ-5,5'-(1,3,6,8-tetraoxo-1,3,6,8-tetrahydrobenzo[lmn][3,8]phenanthroline-2,7-diyl)di(benzene-1,3-dicarboxylato))-tetra-aqua-di-barium(ii) unknown solvate]						
Space Group:	P-1	***Cell:***	***a*** 8.371(0)	***b*** 8.513(1)	***c*** 13.525(1)		
Space Group No.:	2	***(Å,°)***	α 72.79(1)	β 80.71(0)	γ 76.27(0)		
R-Factor (%):	7.43	***Temperature(K):***	293	***Density(g/cm³):***	1.748		

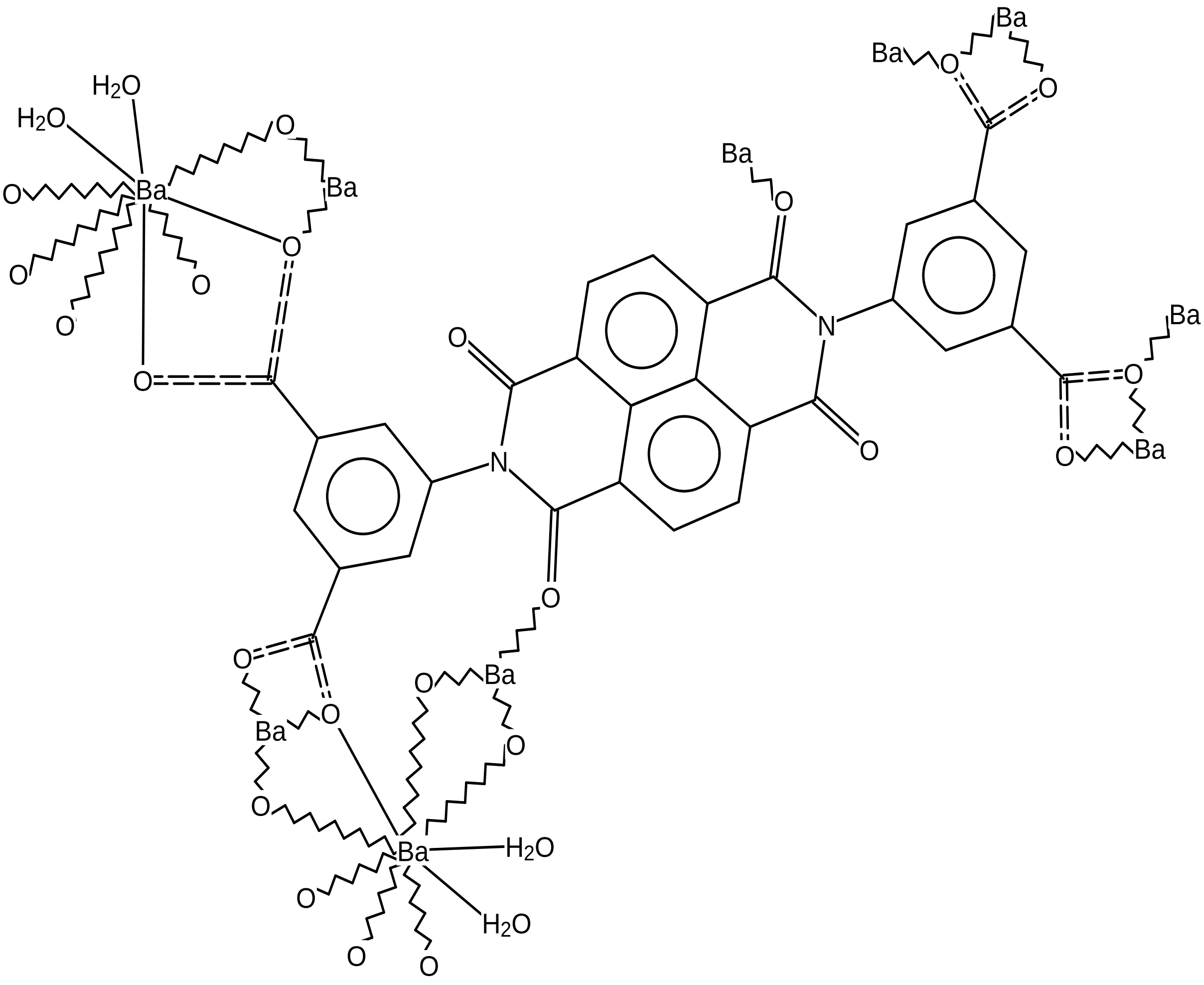

Search: search2 (Mon Jun 30 12:33:47 2025): Hit 86

PUYHAV01

Reference: Fangna Dai, Xiaokang Wang, Yutong Wang, Zhanning Liu, Daofeng Sun (2020) *Angew.Chem.,Int.Ed.* ,**59,**22372

Formula: (C_{30} H_{18} Ba_2 N_2 O_{16})n

Compound Name: catena-[(μ-5,5'-(1,3,6,8-tetraoxo-1,3,6,8-tetrahydrobenzo[lmn][3,8]phenanthroline-2,7-diyl)di(benzene-1,3-dicarboxylato))-tetra-aqua-di-barium(ii) unknown solvate]

Space Group:	P-1	***Cell:***	***a*** 8.371(0)	***b*** 8.513(1)	***c*** 13.525(1)		
Space Group No.:	2	***(Å,°)***	α 72.79(1)	β 80.71(0)	γ 76.27(0)		

R-Factor (%): 7.43 ***Temperature(K):*** 293 ***Density(g/cm³):*** 1.748

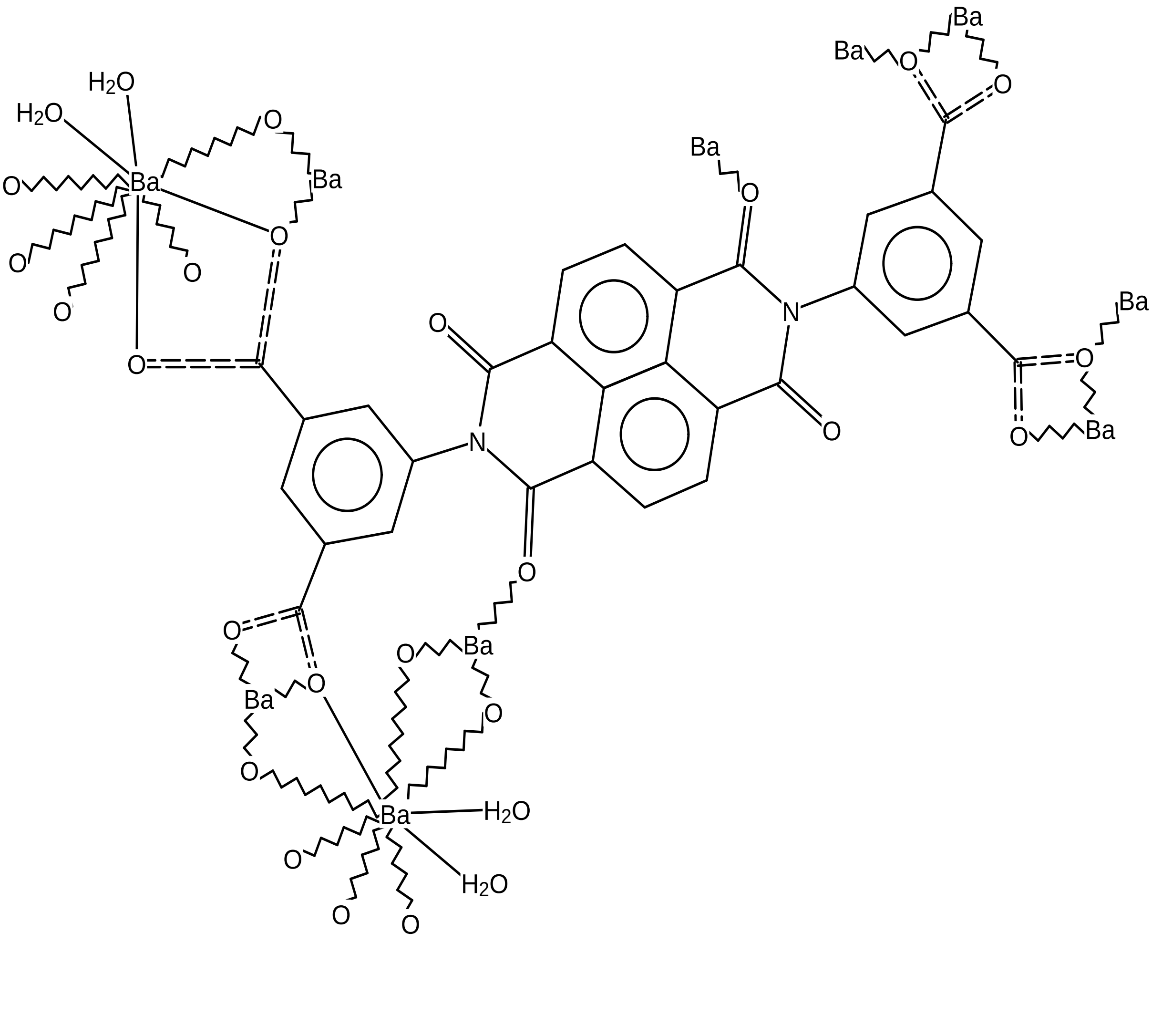

Search: search2 (Mon Jun 30 12:33:47 2025): Hit 87

PUYHEZ

Reference:	Xiaokang Wang (2020)<br>*CSD Communication(Private Communication) ,*							
Formula:	$(C_{33}\ H_{19}\ Ba_2\ N_3\ O_{14})n$							
Compound Name:	catena-[(μ-5,5'-(1,3,6,8-tetraoxo-1,3,6,8-tetrahydrobenzo[lmn][3,8]phenanthroline-2,7-diyl)bis(benzene-1,3-dicarboxylato))-(N,N-dimethylformamide)-aqua-di-barium(ii) unknown solvate]							
Space Group:	P-1	***Cell:***	***a***	9.447(0)	***b***	10.554(0)	***c***	17.338(0)
Space Group No.:	2	***(Å,°)***	α	81.25(0)	β	81.60(0)	γ	78.37(0)
R-Factor (%):	6.85	***Temperature(K):***	150	***Density(g/cm³):***	1.911			

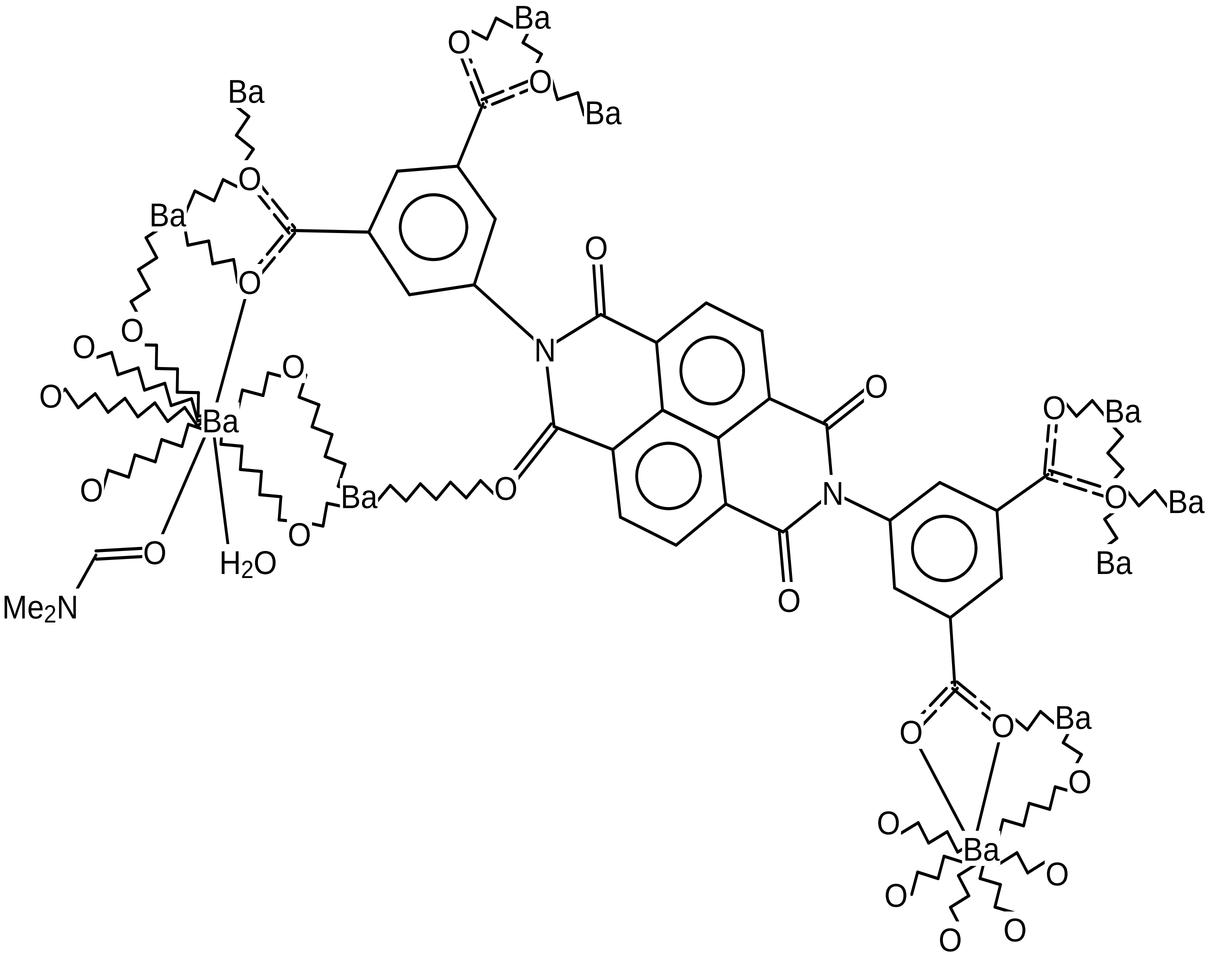

Search: search2 (Mon Jun 30 12:33:47 2025): Hit 88

PUYHEZ01

Reference:	Fangna Dai, Xiaokang Wang, Yutong Wang, Zhanning Liu, Daofeng Sun (2020) *Angew.Chem.,Int.Ed.* ,**59,**22372							
Formula:	$(C_{33}\ H_{19}\ Ba_2\ N_3\ O_{14})n$							
Compound Name:	catena-[(μ-5,5'-(1,3,6,8-tetraoxo-1,3,6,8-tetrahydrobenzo[lmn][3,8]phenanthroline-2,7-diyl)bis(benzene-1,3-dicarboxylato))-(N,N-dimethylformamide)-aqua-di-barium(ii) unknown solvate]							
Space Group:	P-1	***Cell:***	***a***	9.447(0)	***b***	10.554(0)	***c***	17.338(0)
Space Group No.:	2	***(Å,°)***	α	81.25(0)	β	81.60(0)	γ	78.37(0)
R-Factor (%):	6.85	***Temperature(K):***	150	***Density(g/cm³):***	1.911			

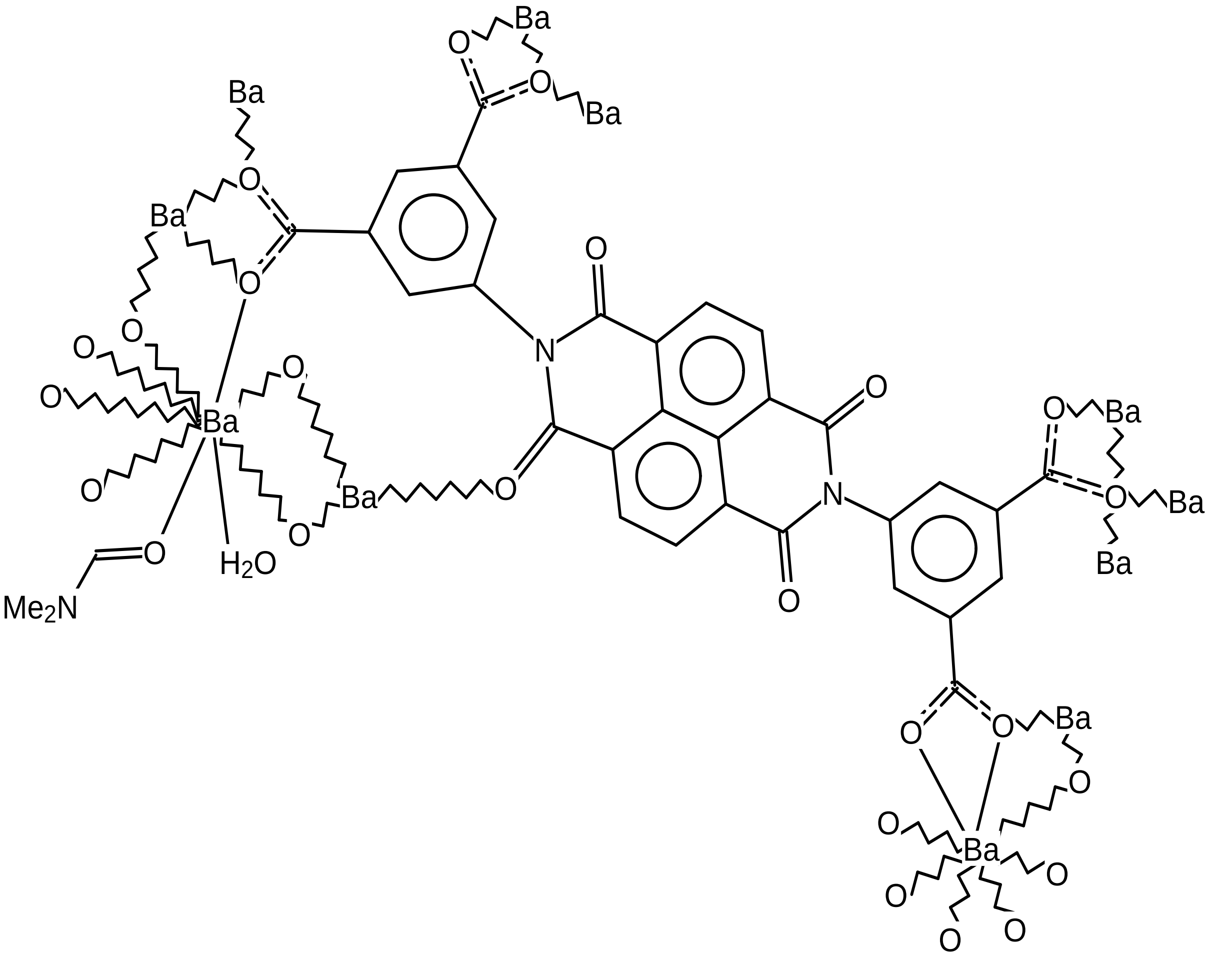

Search: search2 (Mon Jun 30 12:33:47 2025): Hit 89

QOVHER

Reference:	Ming-Hua You, Meng-Hua Li, Hao-Hong Li, Yong Chen, Mei-Jin Lin (2019) *Dalton Trans.* ,**48,**17381
Formula:	$(C_{42} H_{38} N_6 O_{16} Sr_2)n,2(C_3 H_7 N_1 O_1)$
Compound Name:	catena-[(μ-5,5'-(1,3,6,8-tetraoxo-1,3,6,8-tetrahydrobenzo[lmn][3,8]phenanthroline-2,7-diyl)di(benzene-1,3-dicarboxylato))-tetrakis(N,N-dimethylformamide)-di-strontium(ii) N,N-dimethylformamide solvate]

Space Group:	I41/a	***Cell:***	***a***	28.614(4)	***b***	28.614(4)	***c***	13.749(3)
Space Group No.:	88	***(Å,°)***	α	90.00	β	90.00	γ	90.00

R-Factor (%):	5.63	***Temperature(K):***	293	***Density(g/cm^3):***	1.421

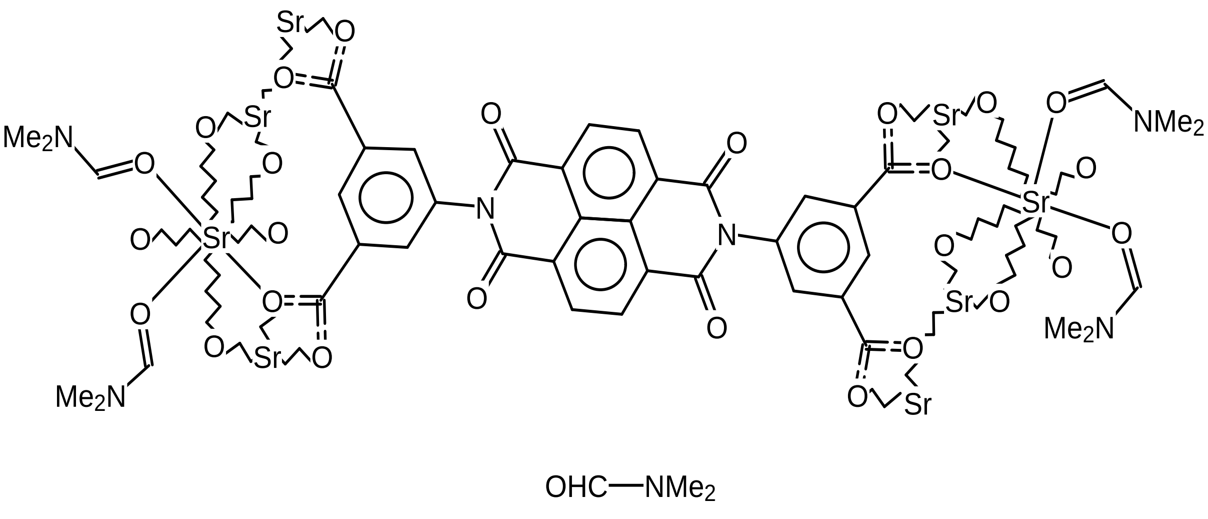

Search: search2 (Mon Jun 30 12:33:47 2025): Hit 90

RERVIW

Reference:	Yan-Xi Tan, Shao-Xia Lin, Caiping Liu, Yiyin Huang, Mi Zhou, Qiang Kang, Daqiang Yuan, Maochun Hong (2018) *Applied Catalysis B: Environmental(Appl.Catal.,B)* ,**227,**425
Formula:	(C_{60} H_{22} Cl_1 Mn_3 N_4 O_{25} $^{3-}$)n,C_4 H_9 N_1 O_1,3(C_2 H_8 N_1 $^{1+}$)
Compound Name:	catena-(tris(dimethylammonium) bis(μ-5,5'-(1,3,6,8-tetraoxo-1,3,6,8-tetrahydrobenzo[lmn][3,8]phenanthroline-2,7-diyl)bis(benzene-1,3-dicarboxylate))-aqua-chloro-tri-manganese dimethylacetamide unknown solvate)
Synonym:	FJI-Y1

Space Group:	P21/c	***Cell:***	***a*** 18.655(0)	***b*** 21.156(0)	***c*** 33.458(0)
Space Group No.:	14	***(Å,°)***	α 90.00	β 127.77(0)	γ 90.00

R-Factor (%):	7.54	***Temperature(K):***	100	***Density(g/cm³):***	1.034

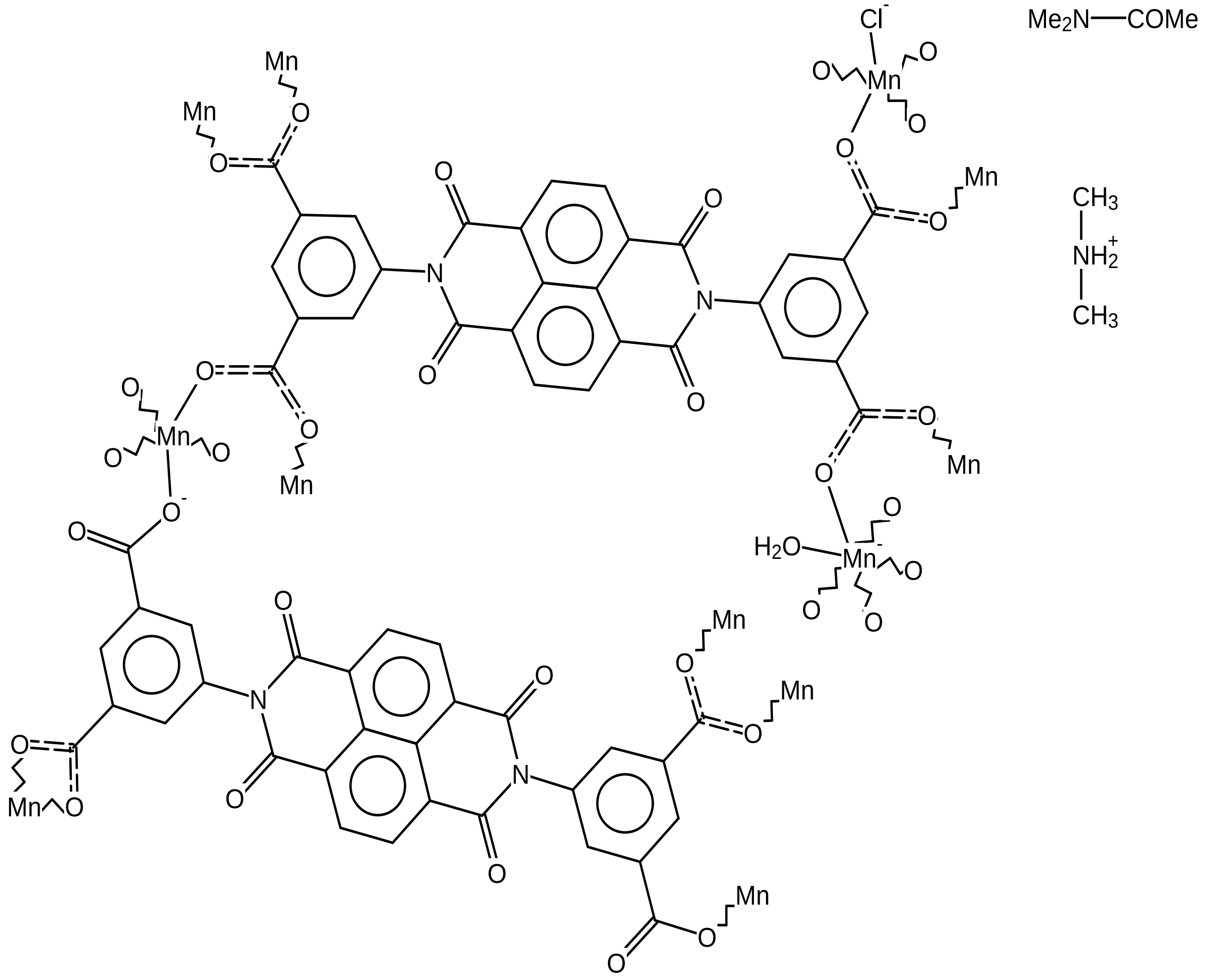

Search: search2 (Mon Jun 30 12:33:47 2025): Hit 91

RERVOC

Reference:	Yan-Xi Tan, Shao-Xia Lin, Caiping Liu, Yiyin Huang, Mi Zhou, Qiang Kang, Daqiang Yuan, Maochun Hong (2018) *Applied Catalysis B: Environmental(Appl.Catal.,B)* ,**227,**425
Formula:	$(C_{120} H_{44} Cl_2 Mn_6 N_8 O_{50}{}^{6-})n,n(C_{30} H_{24} N_6 Ru_1{}^{2+}),4n(C_2 H_8 N_1{}^{1+})$
Compound Name:	catena-(tetrakis(dimethylammonium) tris(2,2'-bipyridine)-ruthenium(ii) tetrakis(μ-5,5'-(1,3,6,8-tetraoxo-1,3,6,8-tetrahydrobenzo[lmn][3,8]phenanthroline-2,7-diyl)bis(benzene-1,3-dicarboxylate))-diaqua-dichloro-hexa-manganese unknown solvate)
Synonym:	FJI-Y2

Space Group:	P21/c	***Cell:***	***a*** 31.465(0)	***b*** 21.187(0)	***c*** 33.759(0)		
Space Group No.:	14	***(Å,°)***	α 90.00	β 109.05(0)	γ 90.00		
R-Factor (%):	10.24	***Temperature(K):***	200	***Density(g/cm³):***	1.109		

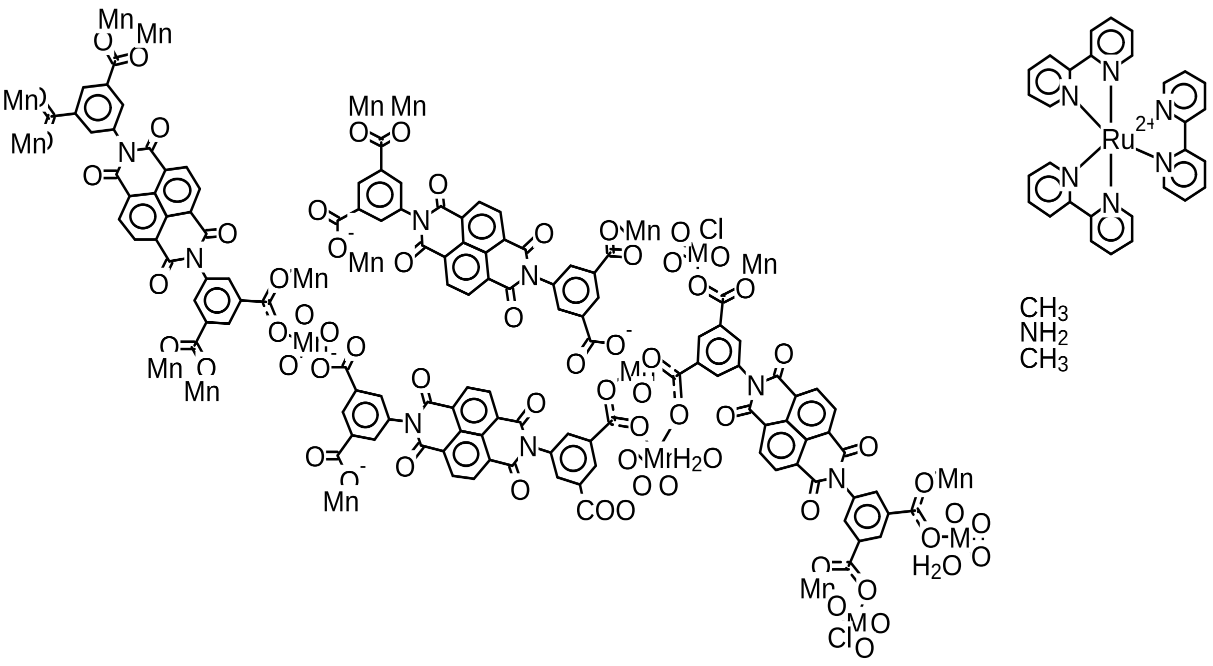

Search: search2 (Mon Jun 30 12:33:47 2025): Hit 92

ROVZIO

Reference:	zhai lijun (2019)<br>*CSD Communication(Private Communication) ,*
Formula:	$(C_{54} H_{38} N_{10} Ni_2 O_{16})n,2(C_4 H_9 N_1 O_1),5(H_2 O_1)$
Compound Name:	catena-[(μ-5,5'-(1,3,6,8-tetraoxo-1,3,6,8-tetrahydrobenzo[lmn][3,8]phenanthroline-2,7-diyl)di(benzene-1,3-dicarboxylato))-bis(μ-1,1'-(1,4-phenylene)di(1H-imidazole))-di-nickel N,N-dimethylacetamide solvate pentahydrate]

Space Group:	P21/m	***Cell:***	***a*** 13.637(0)	***b*** 14.445(0)	***c*** 17.472(0)		
Space Group No.:	11	***(Å,°)***	α 90.00	β 96.75(0)	γ 90.00		
R-Factor (%):	7.13	***Temperature(K):***	293	***Density(g/cm³):***	1.423		

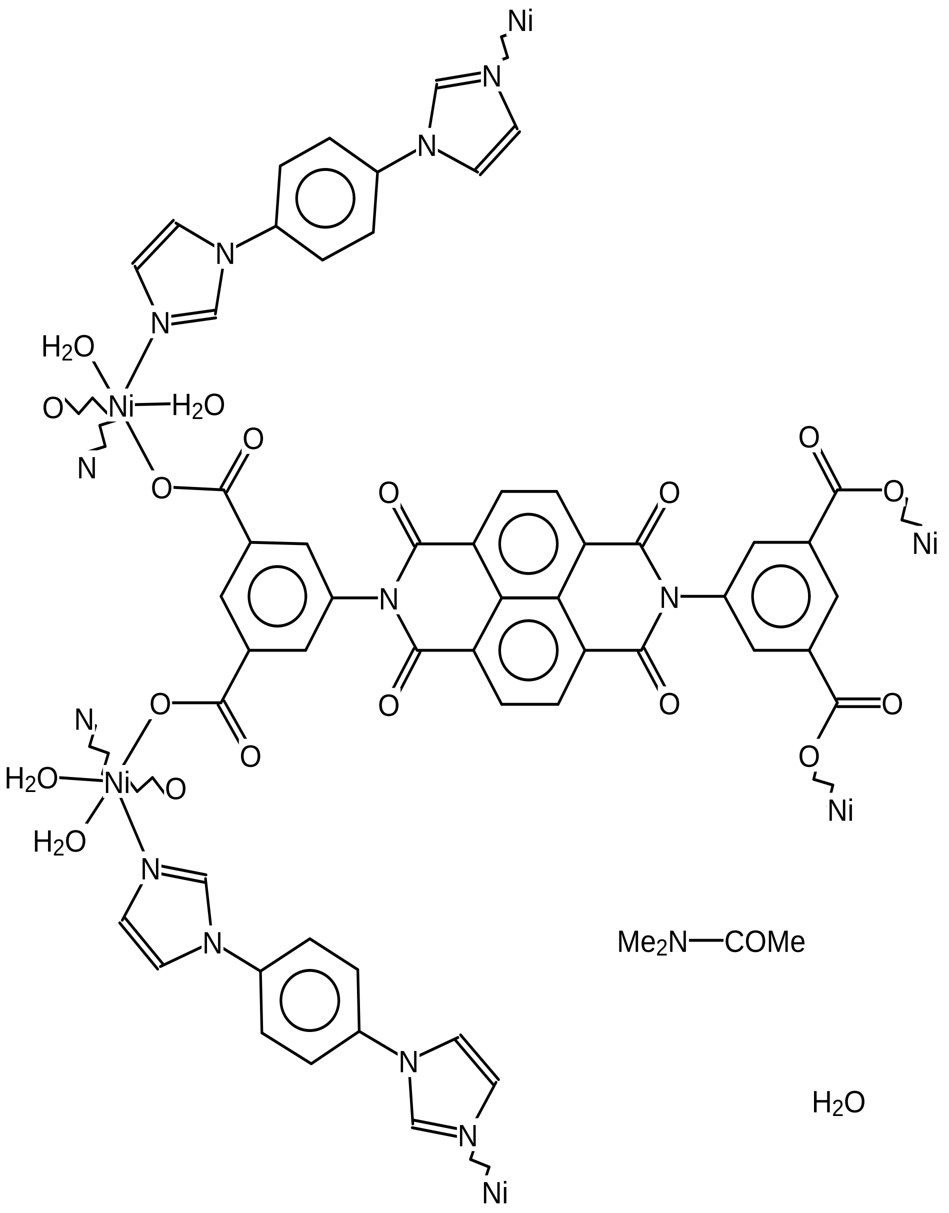

Search: search2 (Mon Jun 30 12:33:47 2025): Hit 93

ROVZIO01

Reference:	Li-Jun Zhai, Hong-Dao Li, Ling-Ling Gao, Jie Zhang, Yu-Lan Niu, Tuo-Ping Hu (2020) *Polyhedron* ,**180,**114417
Formula:	$(C_{54} H_{38} N_{10} Ni_2 O_{16})n,2(C_4 H_9 N_1 O_1),5(H_2 O_1)$
Compound Name:	catena-[(μ-5,5'-(1,3,6,8-tetraoxo-1,3,6,8-tetrahydrobenzo[lmn][3,8]phenanthroline-2,7-diyl)di(benzene-1,3-dicarboxylato))-bis(μ-1,1'-(1,4-phenylene)di(1H-imidazole))-di-nickel N,N-dimethylacetamide solvate pentahydrate]

Space Group:	P21/m	***Cell:***	***a*** 13.637(0)	***b*** 14.445(0)	***c*** 17.472(0)		
Space Group No.:	11	***(Å,°)***	α 90.00	β 96.75(0)	γ 90.00		

R-Factor (%):	7.13	***Temperature(K):***	293	***Density(g/cm³):***	1.423

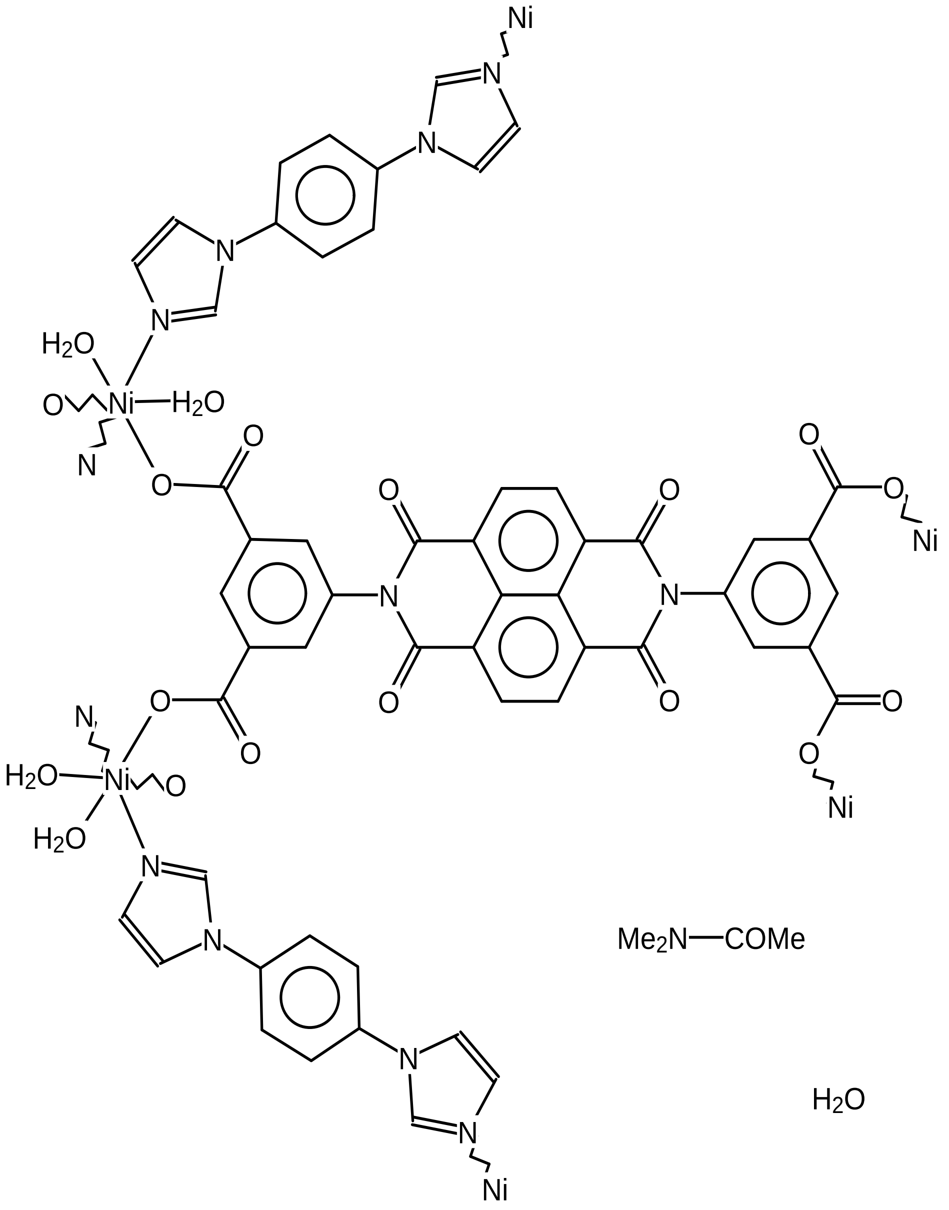

Search: search2 (Mon Jun 30 12:33:47 2025): Hit 94

SASDEZ

Reference: wen dai (2021)
CSD Communication(Private Communication) ,

Formula: $(C_{80}\ H_{68}\ Ca_4\ N_8\ O_{34})n$

Compound Name: catena-[bis(μ-5,5'-(1,3,6,8-tetraoxo-1,3,6,8-tetrahydrobenzo[lmn][3,8]phenanthroline-2,7-diyl)bis(benzene-1,3-dicarboxylato))-tetrakis(N,N-dimethylformamide)-bis(acetic acid)-bis(ethanol)-tetra-calcium(ii)]

Synonym: FJU-212

Space Group:	P-1	***Cell:***	***a***	10.169(0)	***b***	13.816(0)	***c***	14.486(0)
Space Group No.:	2	***(Å,°)***	α	87.53(0)	β	87.13(0)	γ	77.79(0)

R-Factor (%): 8.02 ***Temperature(K):*** 150 ***Density(g/cm³):*** 1.544

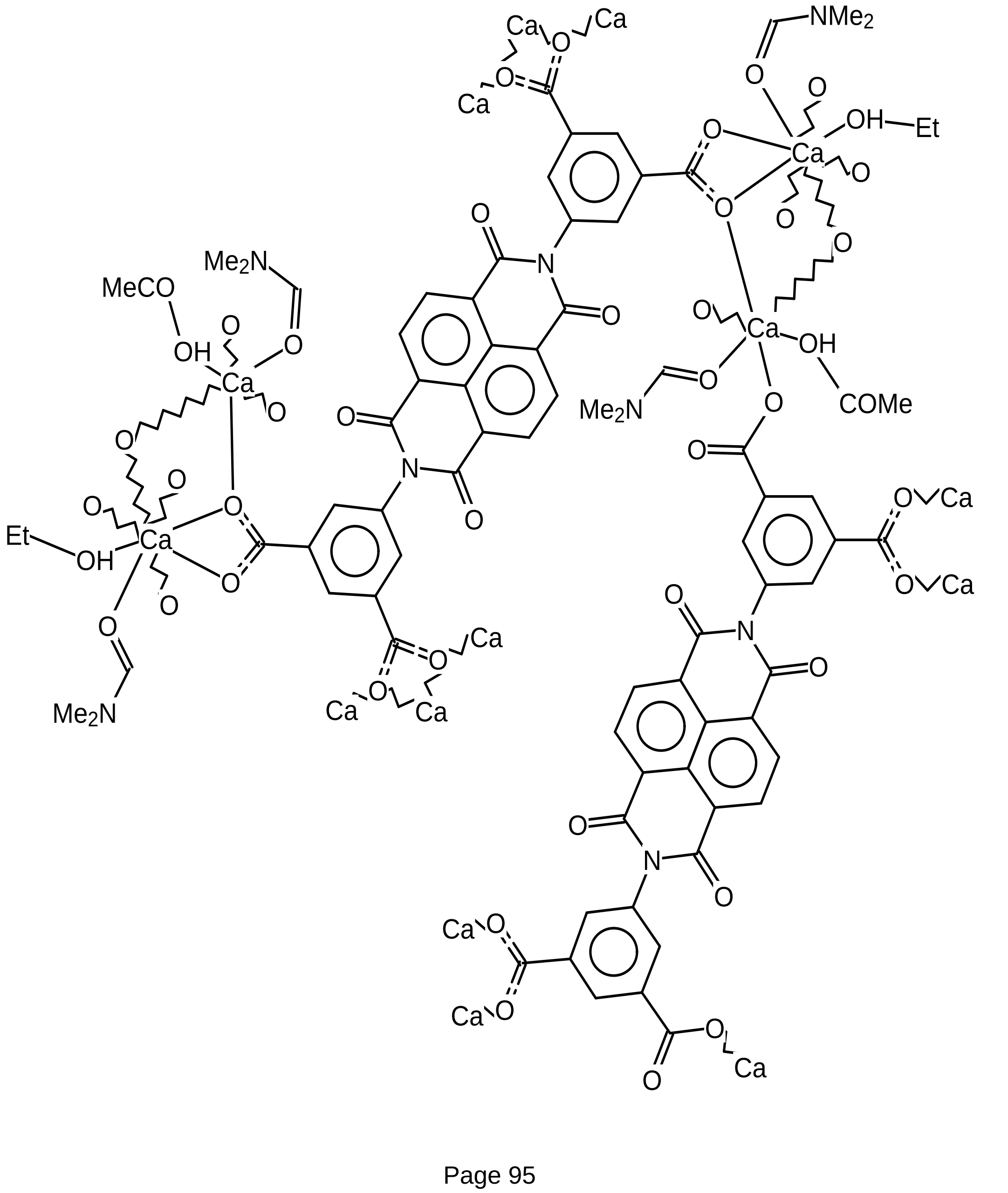

Search: search2 (Mon Jun 30 12:33:47 2025): Hit 95

SASDEZ01

Reference: Wen Dai, Wuji Wei, Zizhu Yao, Shengchang Xiang, Zhangjing Zhang (2022) *Inorg.Chem.Commun.* ,**141,**109497

Formula: $(C_{80} H_{68} Ca_4 N_8 O_{34})n$

Compound Name: catena-[bis(μ-5,5'-(1,3,6,8-tetraoxo-1,3,6,8-tetrahydrobenzo[lmn][3,8]phenanthroline-2,7-diyl)bis(benzene-1,3-dicarboxylato))-tetrakis(N,N-dimethylformamide)-bis(acetic acid)-bis(ethanol)-tetra-calcium(ii)]

Synonym: FJU-212

Space Group:	P-1	***Cell:***	***a***	10.169(0)	***b***	13.816(0)	***c***	14.486(0)
Space Group No.:	2	***(Å,°)***	α	87.53(0)	β	87.13(0)	γ	77.79(0)

R-Factor (%): 8.02 ***Temperature(K):*** 150 ***Density(g/cm³):*** 1.544

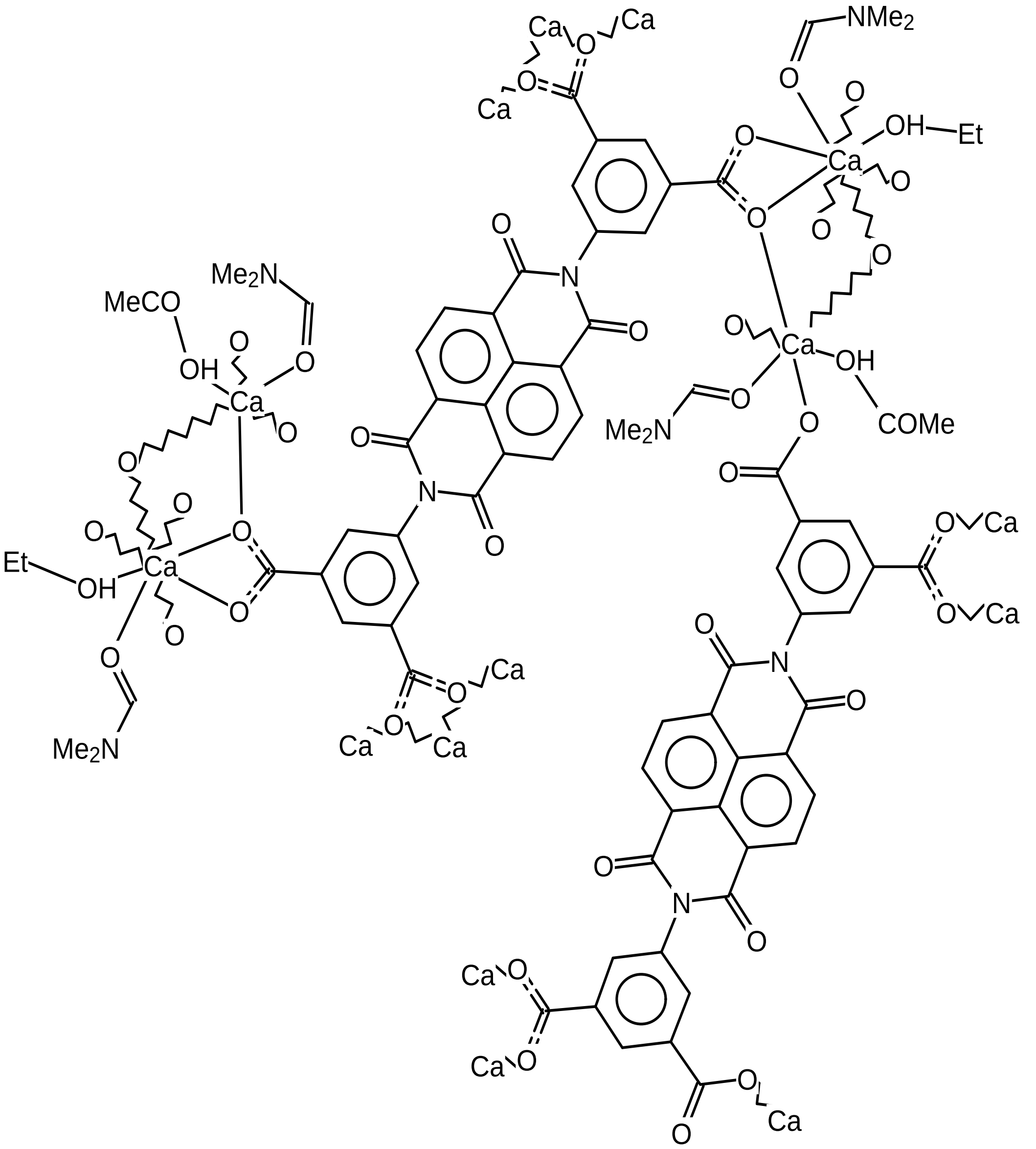

Search: search2 (Mon Jun 30 12:33:47 2025): Hit 96

TIHROU

Reference:	H.T.D.Nguyen, Y.B.N.Tran, H.N.Nguyen, T.C.Nguyen, F.Gandara, P.T.K.Nguyen (2018) *Inorg.Chem.* ,**57,**13772
Formula:	$(C_{90} H_{42} Eu_2 N_6 O_{40}{}^{2-})n,2(C_2 H_8 N_1{}^{1+}),2(H_2 O_1)$
Compound Name:	catena-[bis(dimethylammonium) bis(μ-3,3'-(1,3,6,8-tetraoxo-1,3,6,8-tetrahydrobenzo[lmn][3,8]phenanthroline-2,7-diyl)bis(5-carboxybenzoato))-(μ-5,5'-(1,3,6,8-tetraoxo-1,3,6,8-tetrahydrobenzo[lmn][3, 8]phenanthroline-2,7-diyl)bis(benzene-1,3-dicarboxylato))-tetraaqua-di-europium unknown solvate dihydrate]
Synonym:	MOF-591

Space Group:	P-1	***Cell:***	***a***	10.671(1)	***b***	18.167(1)	***c***	18.767(2)
Space Group No.:	2	***(Å,°)***	α	109.77(0)	β	102.20(0)	γ	97.90(0)

R-Factor (%): 8.71 ***Temperature(K):*** 100 ***Density(g/cm³):*** 1.161

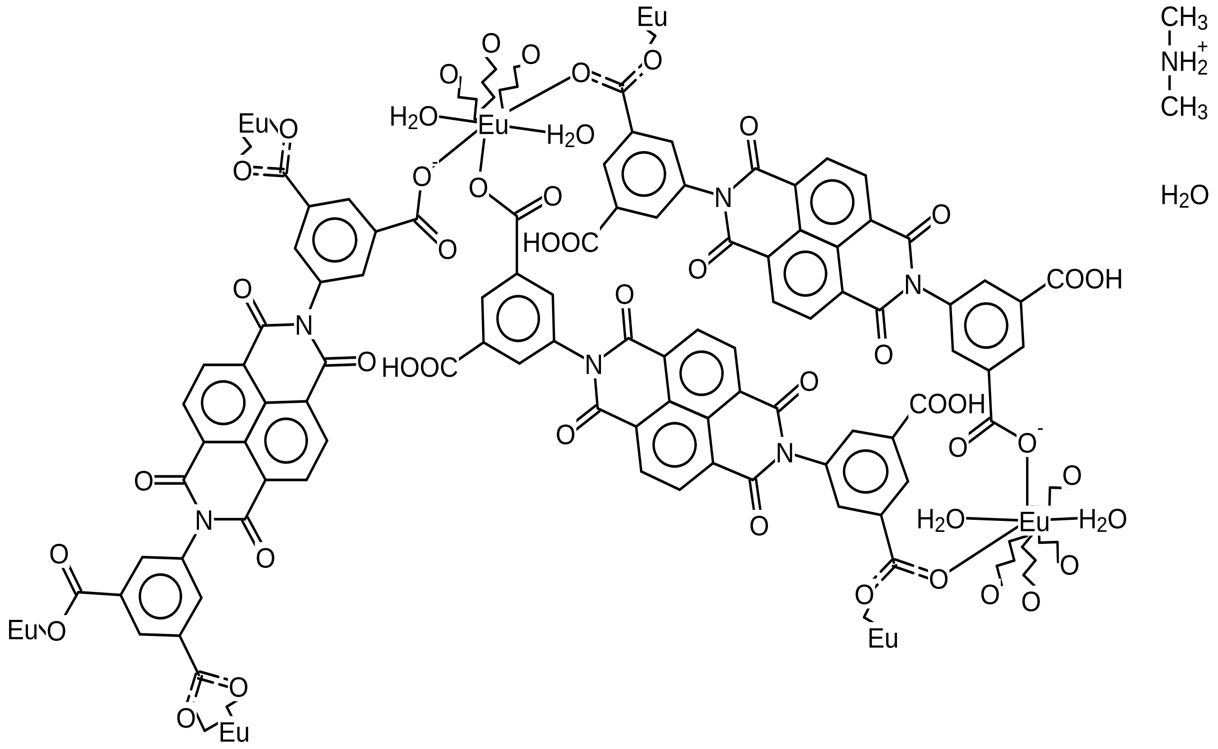

Search: search2 (Mon Jun 30 12:33:47 2025): Hit 97

TIHRUA

Reference:	H.T.D.Nguyen, Y.B.N.Tran, H.N.Nguyen, T.C.Nguyen, F.Gandara, P.T.K.Nguyen (2018) *Inorg.Chem.* ,**57,**13772
Formula:	$(C_{102} H_{66} N_{10} O_{42} Tb_2{}^{2-})n,2(C_2 H_8 N_1{}^{1+}),2(H_2 O_1)$
Compound Name:	catena-[bis(dimethylammonium) bis(μ-3,3'-(1,3,6,8-tetraoxo-1,3,6,8-tetrahydrobenzo[lmn][3,8]phenanthroline-2,7-diyl)bis(5-carboxybenzoato))-(μ- 5,5'-(1,3,6,8-tetraoxo-1,3,6,8-tetrahydrobenzo[lmn][3,8]phenanthroline-2,7-diyl)di(benzene-1,3-dicarboxylato))-diaqua-tetrakis(N,N-dimethylformamide)-di-terbium unknown solvate dihydrate]
Synonym:	MOF-592

Space Group:	P-1	***Cell:***	***a*** 10.657(0)	***b*** 18.130(0)	***c*** 19.501(0)		
Space Group No.:	2	***(Å,°)***	α 65.60(0)	β 74.96(0)	γ 81.32(0)		

R-Factor (%):	5.66	***Temperature(K):***	296	***Density(g/cm³):***	1.279

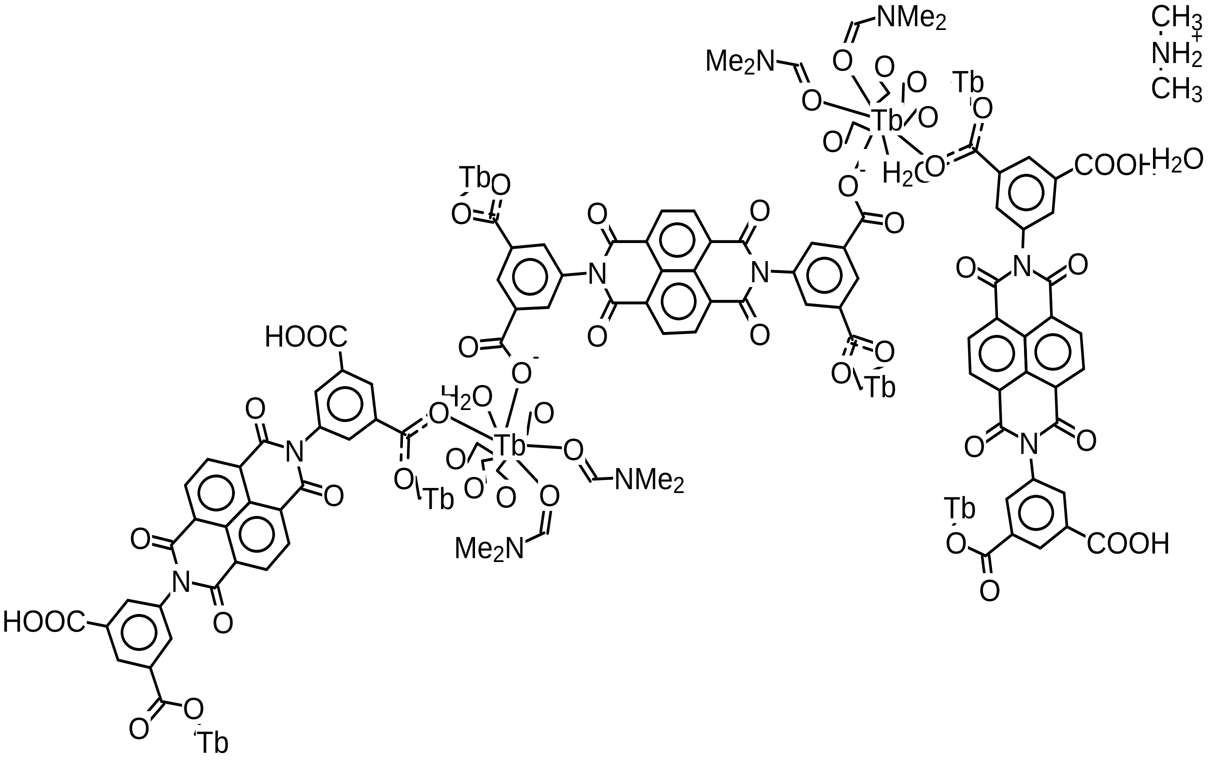

Search: search2 (Mon Jun 30 12:33:47 2025): Hit 98

TIHTUC

Reference: H.T.D.Nguyen, Y.B.N.Tran, H.N.Nguyen, T.C.Nguyen, F.Gandara, P.T.K.Nguyen (2018) *Inorg.Chem.* ,**57,**13772

Formula: $(C_{90} H_{62} N_6 Nd_4 O_{52})n$

Compound Name: catena-[tris(μ-5,5'-(1,3,6,8-tetraoxo-1,3,6,8-tetrahydrobenzo[lmn][3,8]phenanthroline-2,7-diyl)di(benzene-1,3-dicarboxylato))-hexadecaaqua-tetra-neodymium unknown solvate]

Synonym: MOF-590

Space Group:	P-1	***Cell:***	***a***	10.261(0)	***b***	13.000(1)	***c***	19.852(1)
Space Group No.:	2	***(Å,°)***	α	88.68(0)	β	82.49(0)	γ	73.64(0)

R-Factor (%): 6.03 ***Temperature(K):*** 100 ***Density(g/cm³):*** 1.738

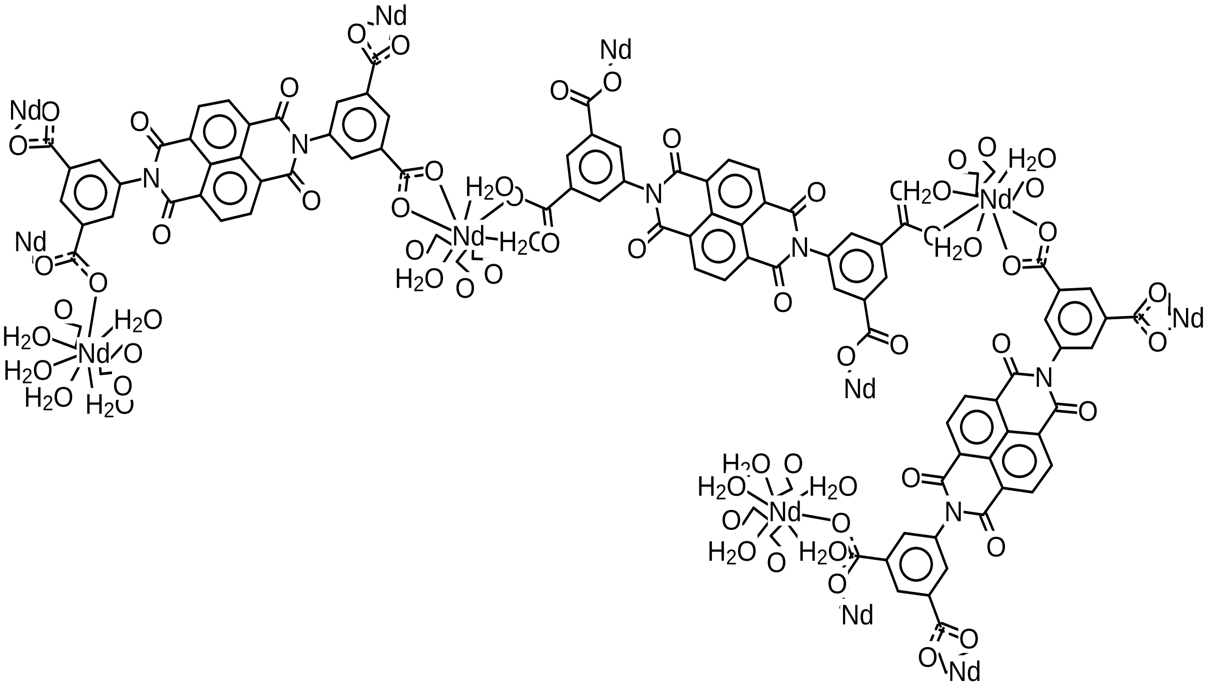

Search: search2 (Mon Jun 30 12:33:47 2025): Hit 99

TILTUH

Reference:	Kun Huang (2023)<br>*CSD Communication(Private Communication) ,*
Formula:	$(C_{54}\ H_{30}\ Cu_2\ N_{14}\ O_{12}\ S_2)n,H_2\ O_1$
Compound Name:	catena-[(μ-5,5'-(1,3,6,8-tetraoxo-1,3,6,8-tetrahydrobenzo[lmn][3,8]phenanthroline-2,7-diyl)bis(benzene-1,3-dicarboxylato))-bis(μ-4,7-bis(1H-imidazol-1-yl)-1,3-dihydro-2,1,3-benzothiadiazole)-di-copper(ii) monohydrate]

Space Group:	P-1	***Cell:***	***a*** 9.999(0)	***b*** 13.305(0)	***c*** 17.007(0)		
Space Group No.:	2	***(Å,°)***	α 95.57(0)	β 90.39(0)	γ 110.97(0)		
R-Factor (%):	5.71	***Temperature(K):***	100	***Density(g/cm³):***	1.009		

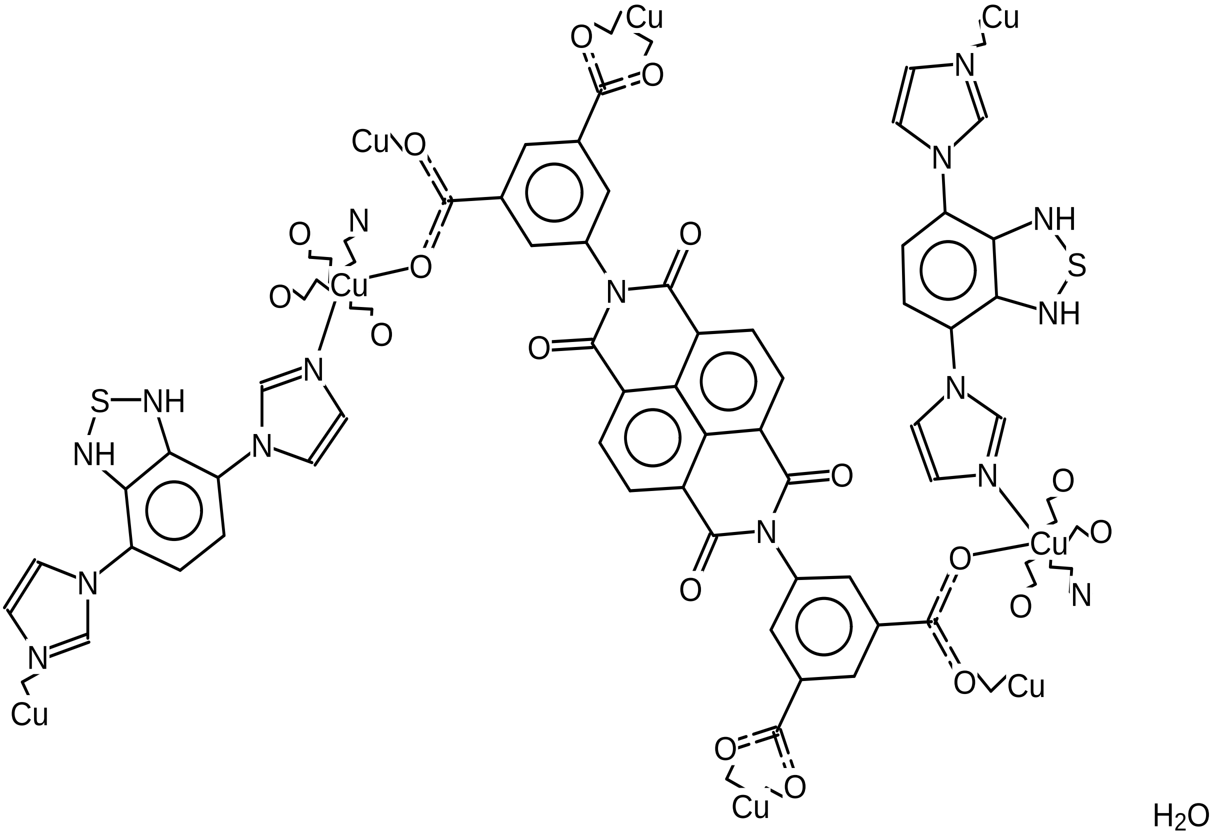

Search: search2 (Mon Jun 30 12:33:47 2025): Hit 100

UDIXIS

Reference:	Rong Huo, Chen Wang, Mei Yan Wang, Ming Yang Sun, Shan Jiang, Yong Heng Xing, Feng Ying Bai (2023) *Inorg.Chem.* ,**62,** 6661
Formula:	$(C_{46} H_{46} Eu_2 N_8 O_{22})n,2(C_4 H_9 N_1 O_1)$
Compound Name:	catena-((μ-5,5'-(1,3,6,8-tetraoxo-1,3,6,8-tetrahydrobenzo[lmn][3,8]phenanthroline-2,7-diyl)bis(benzene-1,3-dicarboxylato))-tetrakis(N,N-dimethylacetamide)-bis(nitrato)-di-europium N,N-dimethylacetamide solvate)

Space Group:	Pbcn	***Cell:***	***a***	10.924(0)	***b***	16.908(0)	***c***	67.617(3)
Space Group No.:	60	***(Å,°)***	α	90.00	β	90.00	γ	90.00

R-Factor (%):	5.52	***Temperature(K):***	303	***Density(g/cm³):***	1.639

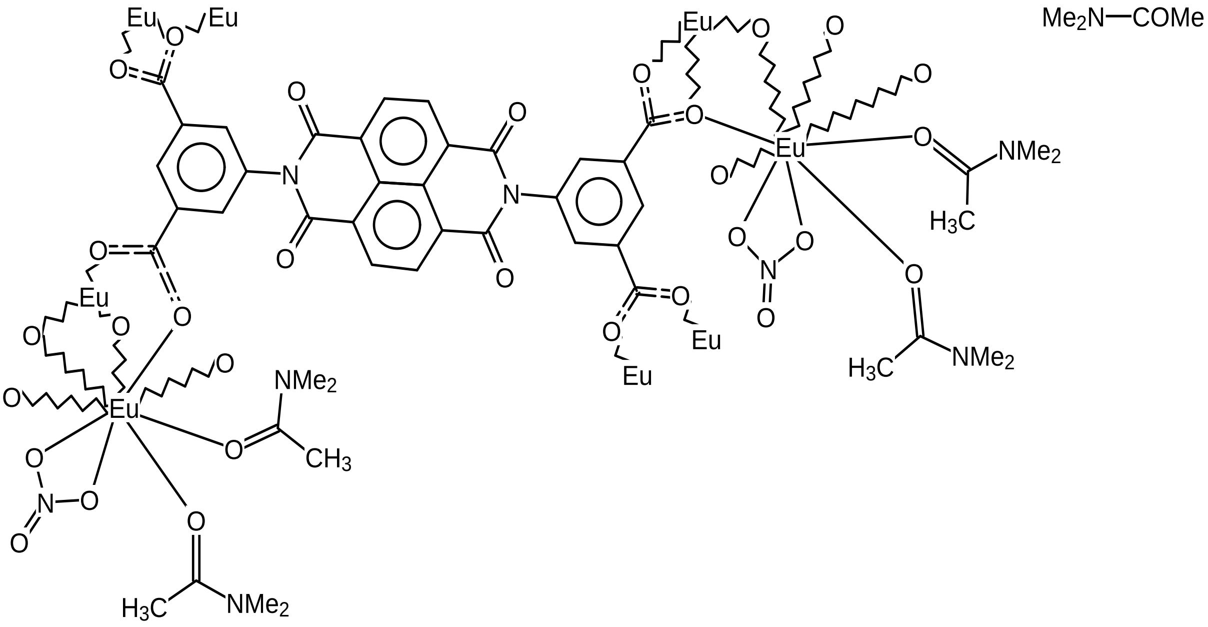

Search: search2 (Mon Jun 30 12:33:47 2025): Hit 101

VASGOP

Reference: Wen-Bin Li, Xiong-Hai Chen, Jia-Zhe Chen, Rong Huang, Jia-Wen Ye, Ling Chen, Hai-Ping Wang, Tao Yang, Liu-Yan Tang, Jie Bai, Zong-Wen Mo, Xiao-Ming Chen (2022)
ACS Applied Materials and Interfaces ,**14,**8458

Formula: $(C_{98}\ H_{56}\ Ca_{6}\ N_{8}\ O_{42})n,6(H_{2}\ O_{1})$

Compound Name: catena-[tris(μ-5,5'-(1,3,6,8-tetraoxo-1,3,6,8-tetrahydrobenzo[lmn][3,8]phenanthroline-2,7-diyl)bis(benzene-1,3-dicarboxylic acid))-bis(N,N-dimethylacetamide)-tetrakis(aqua)-hexa-calcium unknown solvate hexahydrate]

Space Group:	P-1	***Cell:***	***a*** 10.039(0)	***b*** 15.847(0)	***c*** 18.459(0)		
Space Group No.:	2	***(Å,°)***	α 73.21(0)	β 86.21(0)	γ 72.16(0)		
R-Factor (%):	4.05	***Temperature(K):***	150	***Density(g/cm³):***	1.469		

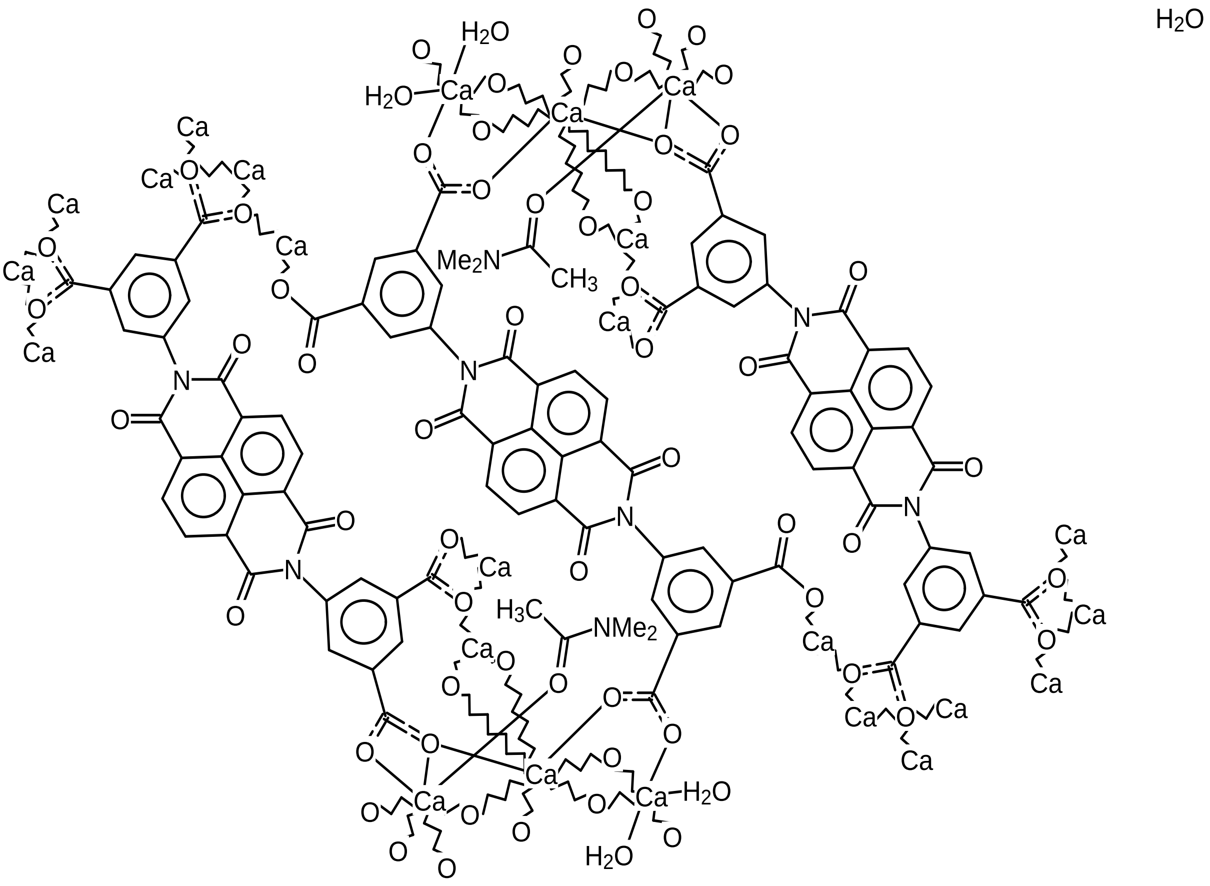

Search: search2 (Mon Jun 30 12:33:47 2025): Hit 102

VIXWIJ

Reference: A.J.Cairns, J.A.Perman, L.Wojtas, V.Ch.Kravtsov, M.H.Alkordi, M.Eddaoudi, M.J.Zaworotko (2008) *J.Am.Chem.Soc.* ,**130,** 1560

Formula: $(C_{93}\ H_{72}\ Co_6\ N_6\ O_{54})n,n(C_1\ H_4\ O_1)$,n$(H_2\ O_1)$

Compound Name: catena-(tris(μ_6-5,5'-(1,3,6,8-tetraoxo-1,3,6,8-tetrahydrobenzo[lmn][3,8]phenanthroline-2,7-diyl)dibenzene-1,3-dicarboxylato)-tris(μ_2-aqua)-dodeca-aqua-trimethanol-hexa-cobalt methanol solvate hydrate)

Space Group:	Pa-3	***Cell:***	***a***	39.288(2)	***b***	39.288(2)	***c***	39.288(2)
Space Group No.:	205	***(Å,°)***	α	90.00	β	90.00	γ	90.00

R-Factor (%): 12.96 ***Temperature(K):*** 100 ***Density(g/cm^3):*** 1.113

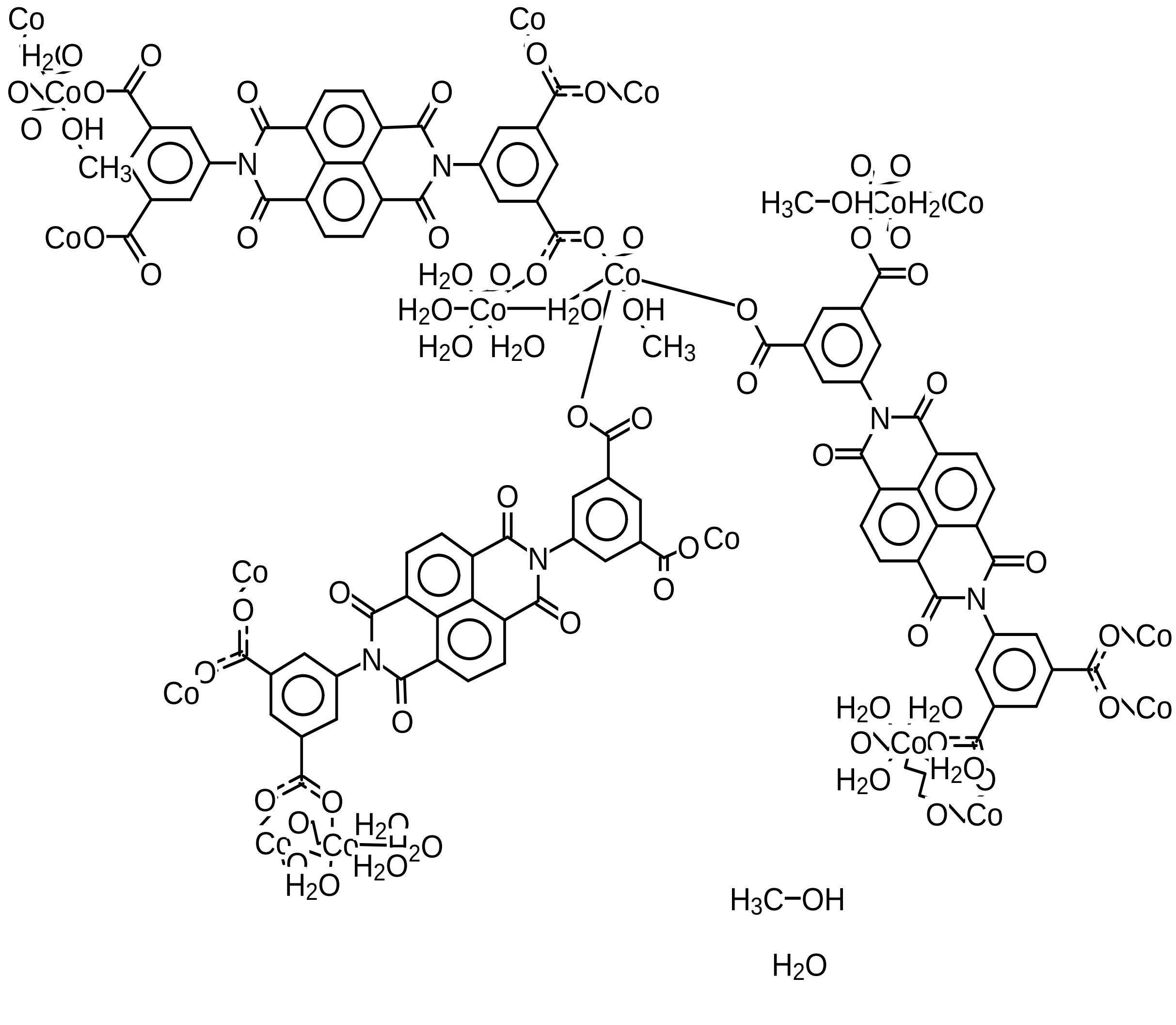

Search: search2 (Mon Jun 30 12:33:47 2025): Hit 103

WEYLUK

Reference:	Fangfang Wei, Yingxiang Ye, Wenhuan Huang, Quanjie Lin, Ziyin Li, Lizhen Liu, Shimin Chen, Zhangjing Zhang, Shengchang Xiang (2018) *Inorg.Chem.Commun.* ,**93,**105						
Formula:	$(C_{50} H_{30} N_6 O_{14} Zn_2)n$						
Compound Name:	catena-[bis(4,4'-bipyridine)-(5,5'-(1,3,6,8-tetraoxo-1,3,6,8-tetrahydrobenzo[lmn][3,8]phenanthroline-2,7-diyl)di(benzene-1,3-dicarboxylato))-diaqua-di-zinc(ii)]						
Synonym:	FJU-34						
Space Group:	P-1	***Cell:***	***a*** 9.397(0)	***b*** 9.531(0)	***c*** 14.572(0)		
Space Group No.:	2	***(Å,°)***	α 94.85(0)	β 95.06(0)	γ 110.44(0)		
R-Factor (%):	4.98	***Temperature(K):***	292	***Density(g/cm³):***	1.469		

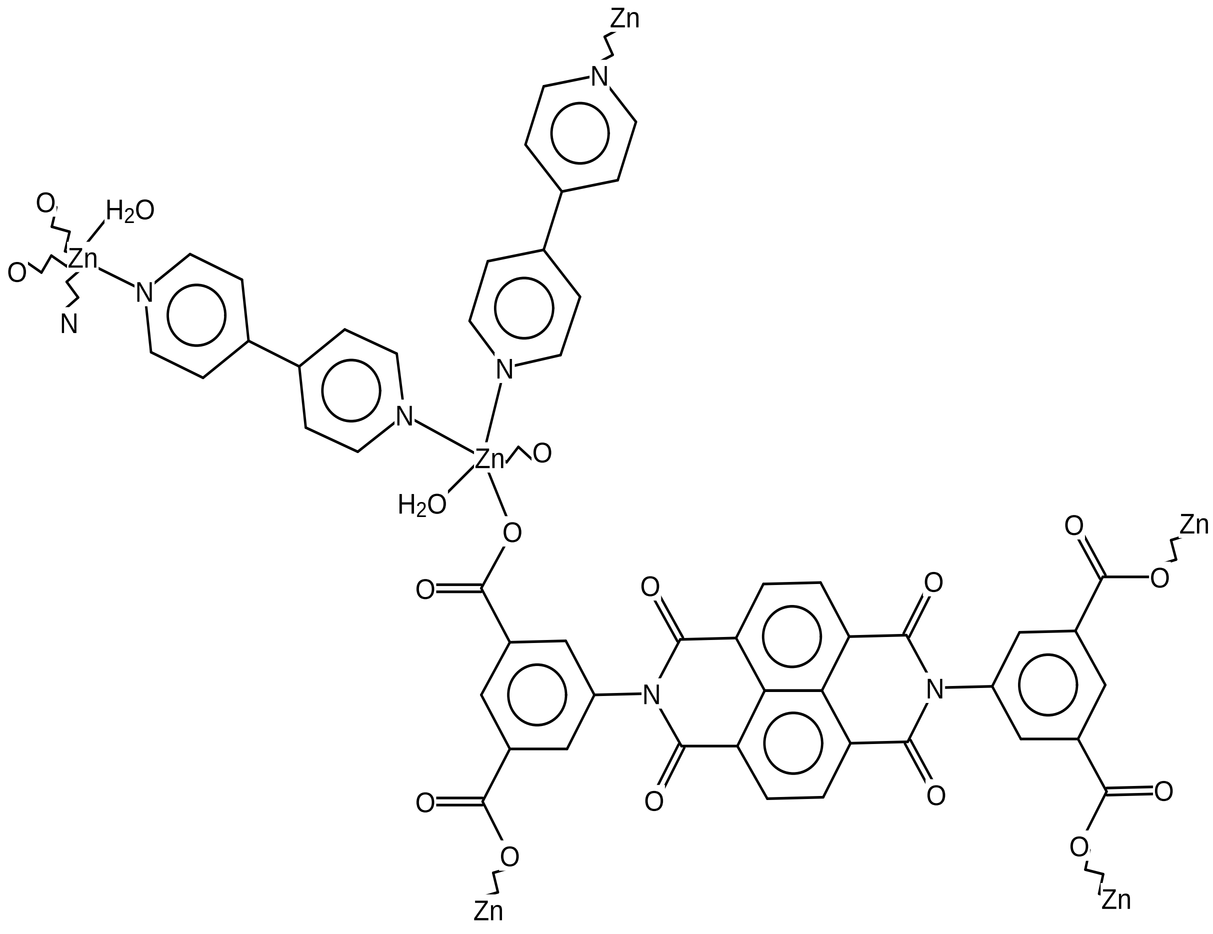

Search: search2 (Mon Jun 30 12:33:47 2025): Hit 104

XENKIM

Reference:	Lei Han, Lan Qin, Lanping Xu, Yan Zhou, Junliang Sun, Xiaodong Zou (2013) *Chem.Commun.* ,**49,**406
Formula:	$(C_{42}\ H_{38}\ Ca_2\ N_6\ O_{16})n,2n(C_3\ H_7\ N_1\ O_1)$
Compound Name:	catena-((μ_8-5,5'-(1,3,6,8-Tetraoxo-1,3,6,8-tetrahydrobenzo[lmn][3,8]phenanthroline-2,7-diyl)diisophthalato)-tetrakis(N,N-dimethylformamide)-di-calcium N,N-dimethylformamide solvate)

Space Group:	I41/a	***Cell:***	***a***	28.281(0)	***b***	28.281(0)	***c***	13.549(0)
Space Group No.:	88	***(Å,°)***	α	90.00	β	90.00	γ	90.00

R-Factor (%):	6.13	***Temperature(K):***	298	***Density(g/cm³):***	1.360

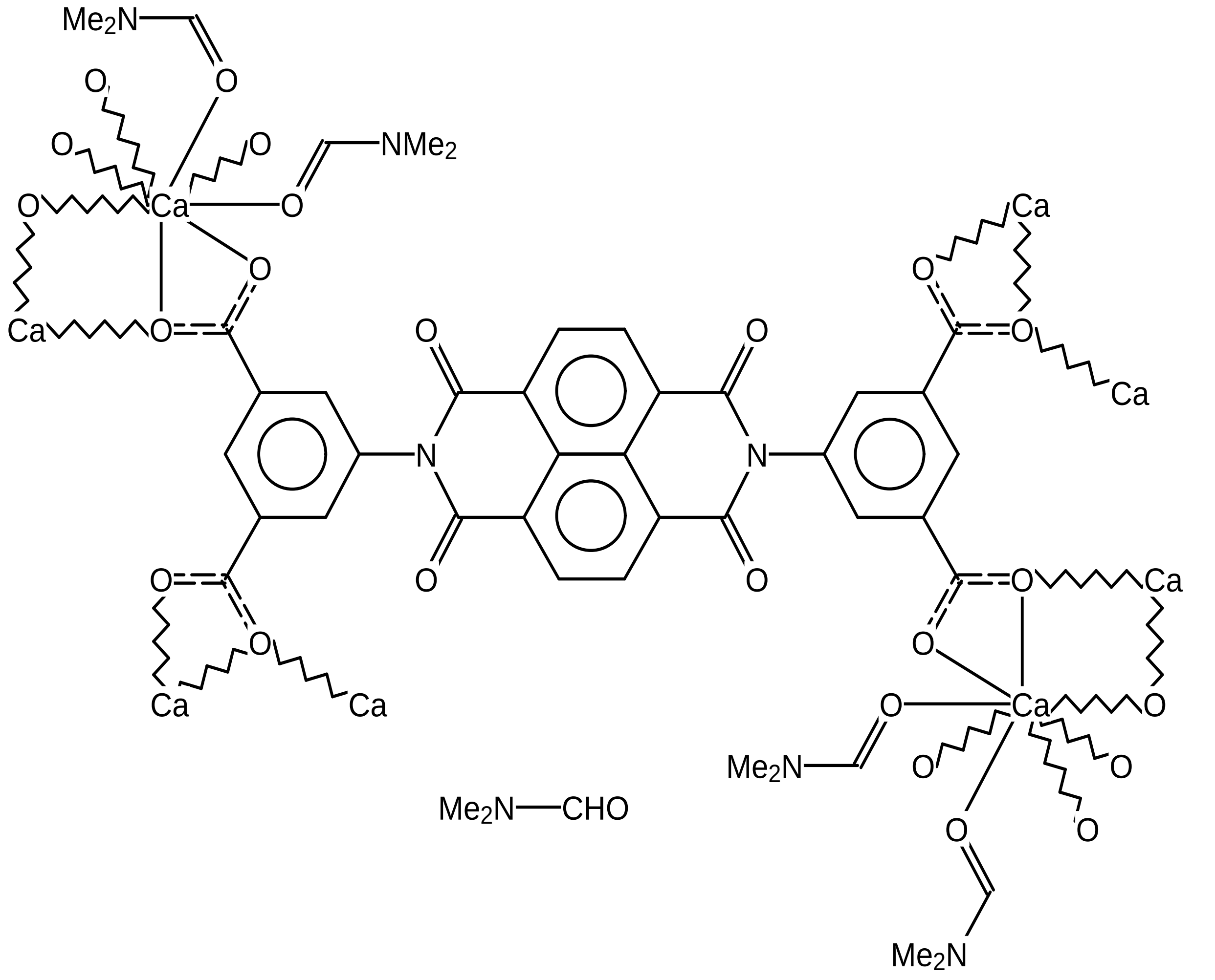

Search: search2 (Mon Jun 30 12:33:47 2025): Hit 105

XENKIM01

Reference:	Ming-Hua You, Meng-Hua Li, Hao-Hong Li, Yong Chen, Mei-Jin Lin (2019) *Dalton Trans.* ,**48,**17381
Formula:	$(C_{42} H_{38} Ca_2 N_6 O_{16})n,2(C_3 H_7 N_1 O_1)$
Compound Name:	catena-[(μ-5,5'-(1,3,6,8-tetraoxo-1,3,6,8-tetrahydrobenzo[lmn][3,8]phenanthroline-2,7-diyl)di(benzene-1,3-dicarboxylato))-tetrakis(N,N-dimethylformamide)-di-calcium(ii) N,N-dimethylformamide solvate]

Space Group:	I41/a	***Cell:***	***a***	28.159(1)	***b***	28.159(1)	***c***	13.456(1)
Space Group No.:	88	***(Å,°)***	α	90.00	β	90.00	γ	90.00

R-Factor (%):	7.02	***Temperature(K):***	293	***Density(g/cm^3):***	1.381

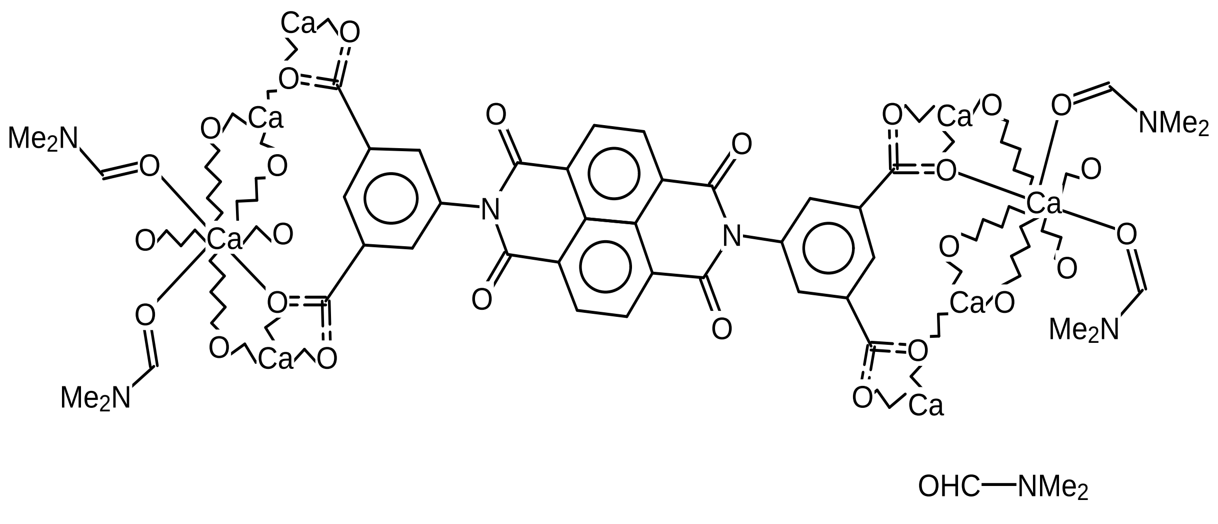

Search: search2 (Mon Jun 30 12:33:47 2025): Hit 106

YIWFAO

Reference: Sandeep Singh Dhankhar, N.Sharma, C.M.Nagaraja
(2019) *Inorg.Chem.Front.* ,**6,**1058

Formula: $(C_{42}\ H_{26}\ N_4\ O_{14}\ Zn_2)n,8(H_2\ O_1)$

Compound Name: catena-[(μ-4,4'-(ethane-1,2-diyl)dipyridine)-(μ-5,5'-(1,3,6,8-tetraoxo-1,3,6,8-tetrahydrobenzo[lmn][3,8]phenanthroline-2,7-diyl)di(benzene-1,3-dicarboxylato))-diaqua-di-zinc octahydrate]

Space Group:	P21/n	***Cell:***	***a***	4.821(5)	***b***	17.325(5)	***c***	27.459(5)
Space Group No.:	14	***(Å,°)***	α	90.00	β	91.62(0)	γ	90.00

R-Factor (%): 3.99 ***Temperature(K):*** 298 ***Density(g/cm³):*** 1.573

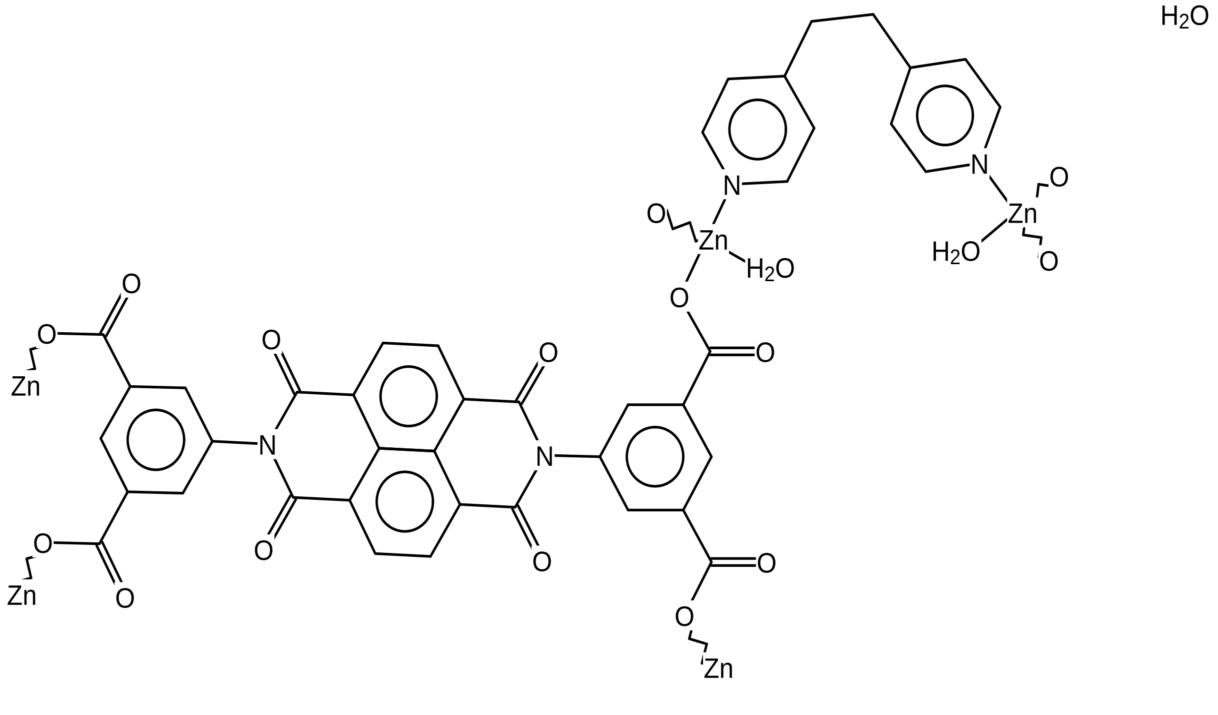

Search: search2 (Mon Jun 30 12:33:47 2025): Hit 107

YIWFES

Reference: Sandeep Singh Dhankhar, N.Sharma, C.M.Nagaraja
(2019) *Inorg.Chem.Front.* ,**6,**1058

Formula: $(C_{54} H_{30} N_6 O_{12} Zn_2)n,6(H_2 O_1)$

Compound Name: catena-[bis(μ-4,4'-(ethene-1,2-diyl)dipyridine)-(μ-5,5'-(1,3,6,8-tetraoxo-1,3,6,8-tetrahydrobenzo[lmn][3,8]phenanthroline-2,7-diyl)di(benzene-1,3-dicarboxylato))-di-zinc hexahydrate]

Space Group:	P2/c	***Cell:***	***a***	10.082(5)	***b***	13.637(5)	***c***	20.120(5)
Space Group No.:	13	***(Å,°)***	α	90.00	β	97.58(0)	γ	90.00

R-Factor (%): 7.17 ***Temperature(K):*** 298 ***Density(g/cm³):*** 1.446

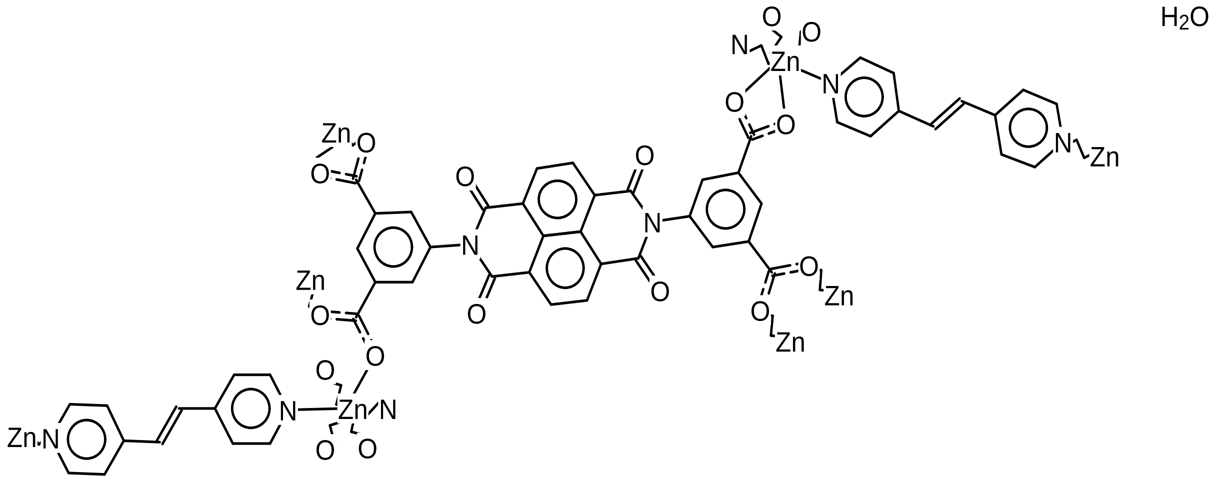

Supplement: Supplementary file 1 [file cg5c00992_si_001.pdf]
